# Study on Potential Differentially Expressed Genes in Idiopathic Pulmonary Fibrosis by Bioinformatics and Next-Generation Sequencing Data Analysis

**DOI:** 10.3390/biomedicines11123109

**Published:** 2023-11-21

**Authors:** Muttanagouda Giriyappagoudar, Basavaraj Vastrad, Rajeshwari Horakeri, Chanabasayya Vastrad

**Affiliations:** 1 Department of Radiation Oncology, Karnataka Institute of Medical Sciences (KIMS), Hubballi 580022, Karnataka, India; mgggoudar@gmail.com; 2 Department of Pharmaceutical Chemistry, K.L.E. Socitey’s College of Pharmacy, Gadag 582101, Karnataka, India; basavarajmv@gmail.com; 3 Department of Computer Science, Govt First Grade College, Hubballi 580032, Karnataka, India; rajeshwarigg@gmail.com; 4 Biostatistics and Bioinformatics, Chanabasava Nilaya, Bharthinagar, Dharwad 580001, Karnataka, India

**Keywords:** bioinformatics analysis, differentially expressed genes, MicroRNAs, transcription factors, idiopathic pulmonary fibrosis

## Abstract

Idiopathic pulmonary fibrosis (IPF) is a chronic progressive lung disease with reduced quality of life and earlier mortality, but its pathogenesis and key genes are still unclear. In this investigation, bioinformatics was used to deeply analyze the pathogenesis of IPF and related key genes, so as to investigate the potential molecular pathogenesis of IPF and provide guidance for clinical treatment. Next-generation sequencing dataset GSE213001 was obtained from Gene Expression Omnibus (GEO), and the differentially expressed genes (DEGs) were identified between IPF and normal control group. The DEGs between IPF and normal control group were screened with the DESeq2 package of R language. The Gene Ontology (GO) and REACTOME pathway enrichment analyses of the DEGs were performed. Using the g:Profiler, the function and pathway enrichment analyses of DEGs were performed. Then, a protein–protein interaction (PPI) network was constructed via the Integrated Interactions Database (IID) database. Cytoscape with Network Analyzer was used to identify the hub genes. miRNet and NetworkAnalyst databaseswereused to construct the targeted microRNAs (miRNAs), transcription factors (TFs), and small drug molecules. Finally, receiver operating characteristic (ROC) curve analysis was used to validate the hub genes. A total of 958 DEGs were screened out in this study, including 479 up regulated genes and 479 down regulated genes. Most of the DEGs were significantly enriched in response to stimulus, GPCR ligand binding, microtubule-based process, and defective GALNT3 causes HFTC. In combination with the results of the PPI network, miRNA-hub gene regulatory network and TF-hub gene regulatory network, hub genes including LRRK2, BMI1, EBP, MNDA, KBTBD7, KRT15, OTX1, TEKT4, SPAG8, and EFHC2 were selected. Cyclothiazide and rotigotinethe are predicted small drug molecules for IPF treatment. Our findings will contribute to identification of potential biomarkers and novel strategies for the treatment of IPF, and provide a novel strategy for clinical therapy.

## 1. Introduction

Lung fibrosis is a progressive, chronic, and irreversible fibrosing interstitial lung disease; it affects 2–9 people per 1,00,000 worldwide [1]. It is also known as idiopathic pulmonary fibrosis (IPF) [2].It is characterized by clinical symptoms of cough and dyspnea, declining pulmonary function with impaired gas exchange, and progressive lung scarring [3]. Debate on the good strategy for IPF management continues despite great advancement in treating IPF in recent decades. Extensive research has shown current therapeutic approaches in IPF included anti-inflammatory drugs, cytotoxic and immunosuppressive agents, immunomodulators, antifibrotic agents, and antioxidants targeting several crucial signaling pathways that predominantly regulate the inflammation.Genetic, aging, and environmental factors are thought to be the contributing factors to IPF [4]. However, IPF might also can be caused by many unknown causes, such as pulmonary hypertension [5], lung cancer [6], diabetes mellitus [7], dermatomyositis [8],polymyositis [9], systemic sclerosis [10],mixed connective tissue disease [11], systemic lupus erythematosus [12], rheumatoid arthritis [13],sarcoidosis [14], scleroderma [15], pneumonia [16], heart failure [17], obesity [18], viral respiratory diseases [19], gastroesophageal reflux disease [20], chronic obstructive pulmonary disease [21], and airway inflammation [22], which cannot be well solved by current drug treatment and IPF is still a complicated incurable pulmonary disease. Thus, it is necessary for us to utilize bioinformatics and next-generation sequencing (NGS) technology to explore the molecular pathogenesis or potential treatments of IPF.

The recent bioinformatics and NGS data analysis of specimens from sufferers and normal individuals enable us to investigate numerous diseases at diverse levels from somatic mutations and copy number variations to genomic expressions at the transcriptomic level [23,24]. Defining the molecular targets for diagnosis and re-examination is crucial for therapeutic action and prognostic outcome of IPF patients. Many researchers are committed to exploring new biomarkers for IPF. For example, studies have found that serum levels of p53 [25], TINF2 [26], ELMOD2 [27], TERT [28], and ABCA3 [29] are altered among IPF patients. Several investigations have described that significantsignaling pathways in IPF were identified such as TGF-β signaling pathway [30], Smad and STAT3 signaling pathways [31], p38 MAPK signaling pathway [32], Wnt/β-Catenin signaling pathway [33], and JAK-STAT signaling pathway [34]. Despite the increase in different potential biomarkers and pathways in IPF, such efforts have not yet yielded satisfactory results.In this regard, it is necessary to address the association of genes and signaling pathways in candidate genomes with IPF development.

Omics data areroutinely utilized to discover and validate new disease biomarkers.Potential and novel diagnostic biomarkers and therapeutic targets of IPF have been proposed in such integrative bioinformatics studies based on the identification of differentially expressed genes (DEGs). Here, NGS datasets (GSE213001), which includes gene expression data from IPF and normal control samples, were obtained from the Gene Expression Omnibus (GEO) [https://www.ncbi.nlm.nih.gov/geo/] (accessed on 23 July 2023) [35] database. Non-biased bioinformatics analyses, including identification of DEGs, gene ontology (GO) term enrichment analysis, REACTOME pathway enrichment analysis, protein–protein interaction (PPI) network analysis, module analysis, miRNA-hub gene regulatory network, TF-hub gene regulatory network, and protein–drug interaction network analysis were conducted, and the findings were further validated by receiver operating characteristic (ROC) curve analysis. The investigation probably revealed the pathogenic mechanism and potential therapeutic target of IPF.

## 2. Materials and Methods

### 2.1. Next-Generation Sequencing Data Source

**Figure 1:**
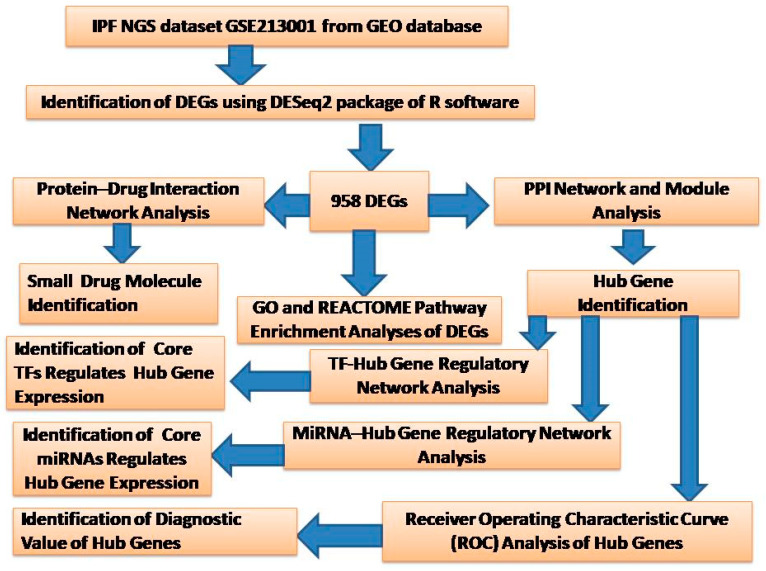
The overall design of the study. DEGs, differentially expressed genes; GO, Gene Ontology; PPI, protein–protein interaction; TF, transcription factor.

NGS data of human mRNA regardingIPF research (GSE213001) were obtained from the GEO database. There were 180 samples in GSE213001, including 98 IPF samples and 41 normal control samples without IPF. All samples were detected through the Illumina HiSeq 3000 (Homo sapiens) platform. The detail pipeline of this study was showed in Figure 1.

### 2.2. Identification of DEGs 

The DESeq2 package [36] of R language was utilized to screen DEGs. The false discovery rate (FDR) of the Benjamini–Hochberg (BH) method was applied to adjust *p*-values for multiple comparisons [37]. The significant differentially expressed cut-off was set as |logFC|  > 0.512 for up regulated genes and |logFC|  < −0.831 for down regulated genes, and adjusted *p* <  0.05. The volcano map and heatmap of the DEGs were respectively generated using the ggplot2 and gplot packages in R software (Version. 3.4.1 abd Version 3.1.3)

### 2.3. GO and Pathway Enrichment Analyses of DEGs

GO and REACTOME pathway enrichment analyses of DEGs were performed via g:Profiler (http://biit.cs.ut.ee/gprofiler/) (Accessed on 2 August 2023) [38]. The GO enrichment analysis (http://www.geneontology.org) (Accessed on 2 August 2023) [39] consists of biological processes (BP), cellular components (CC), and molecular functions (MF), which can be used to clarify the potential biological functions of the enriched genes. Pathway enrichment analysis can be used to identify the main biochemical metabolic pathways and signal transduction pathways involved in enriched genes. REACTOME (https://reactome.org/) (Accessed on 2 August 2023) [40] is a pathway database resource for understanding high-level biological functions and utilities. Gene count > 2 and *p* < 0.05 were set as the threshold.

### 2.4. Construction of the PPI Network and Module Analysis

To further analyze the impacts of DEGs on IPF, the PPI network was constructed among various DEGs. Also, the online software Integrated Interactions Database (IID) (http://iid.ophid.utoronto.ca/search_by_proteins/) (accessed on 26 July 2023) [41] was used to analyze the interactions of proteins encoded by DEGs. Then, the Cytoscape software (V3.10.0; http://cytoscape.org/) (accessed on 31 July 2023) [42] was utilized to visualize the PPI network. Hub genes were identified using Network Analyzer, a plug-in of Cytoscape software. Finally, the degree [43], betweenness [44], stress [45], and closeness [46] of each hub genes was obtained by analyzing the topological structure of the PPI network. Significant modules in the PPI network were identified by PEWCC [47], another plug-in of Cytoscape software.

### 2.5. Construction of the miRNA-Hub Gene Regulatory Network

MicroRNAs (miRNAs) can control gene expression by promoting or inhibiting mRNA degradation and translation. We therefore investigate miRNAs involved in regulatory mechanism and development process in IPF. The hub genes in PPI were selected as the promising targets for searching miRNA through the miRNet database (https://www.mirnet.ca/) (accessed on 4 August 2023), which is an experimentally validated miRNA–hub gene interactions database [48]. This database contains miRNA-hub gene regulatory network data from 14 disparate sources including TarBase, miRTarBase, miRecords, miRanda (S mansoni only), miR2Disease, HMDD, PhenomiR, SM2miR, PharmacomiR, EpimiR, starBase, TransmiR, ADmiRE, and TAM 2. The intersection of miRNAs and hub genes in IPF was used to construct the miRNAs–hub genes regulated network. The identified miRNA -hub gene regulatory network was visualized using the Cytoscape software [42].

### 2.6. Construction of the TF-Hub Gene Regulatory Network

Transcription factors (TFs) can control gene expression by promoting or inhibiting translation. We therefore investigate TFs involved in regulatory mechanism and development process in IPF. The hub genes in PPI were selected as the promising targets for searching TF through the NetworkAnalyst database (https://www.networkanalyst.ca/) (Accessed on 4 August 2023) [49]. This database contains TF–hub gene regulatory network data from JASPAR. The results of this process were arranged such that each entry was a specific TF–hub gene interaction associated with its source link. The intersection of TFs and hub genes in IPF was used to construct the TFs–hub genes regulated network. The identified TF–hub gene regulatory network was visualized using the Cytoscape software [42].

### 2.7. Construction of the Protein–Drug Interaction Network

In this investigation, prediction of protein–drug interactions or small molecules identification is one of the key parts. The DEGs were selected as the promising targets for searching small drug molecules through the NetworkAnalyst database (https://www.networkanalyst.ca/) (Accessed date 16 October 2023) [49]. This database contains protein–drug interaction network data from DrugBank. The results of this process were arranged such that each entry was a specific protein–drug interaction associated with its source link. The intersection of drug and DEGs in IPF was used to construct the protein–drug interaction network. The identified protein–drug interaction network was visualized using the Cytoscape software [42].

### 2.8. Receiver Operating Characteristic Curve (ROC) Analysis

ROC curve analyses to determine the specificity, sensitivity, likelihood ratios, positive predictive values, and negative predictive values for all possible thresholds of the ROC curve were performed using the R packages “pROC” [50]. The receiver operator characteristic curves were plotted and area under curve (AUC) was determined independently to assess the conduct of each model. The diagnostic values of the genes were predicted based on the ROC curve analysis. AUC  >  0.8 marked that the model had a good fitting effect.

## 3. Results 

### 3.1. Identification of DEGs

**Figure 2:**
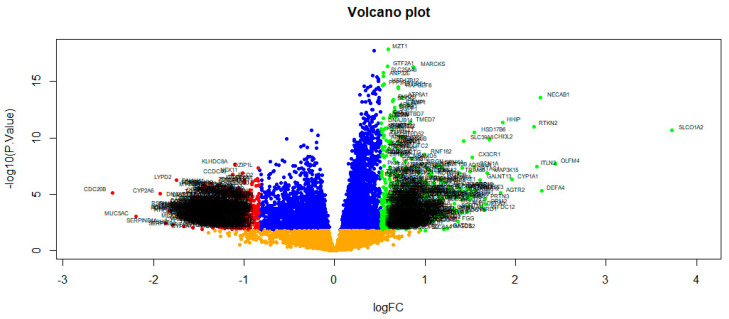
Volcano plot of DEGs between IPF samples and normal control samples. Green dot represented up regulated significant genes and red dot represented down regulated significant genes.

**Figure 3:**
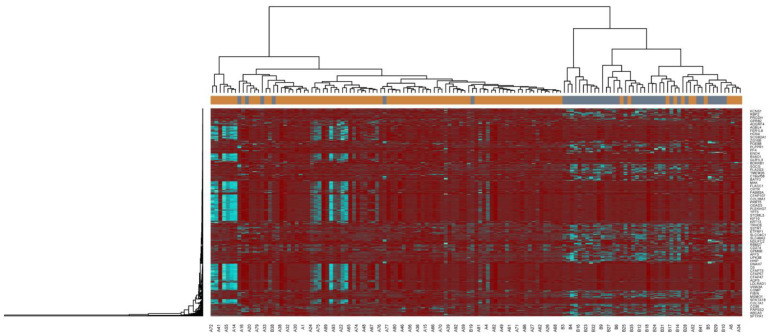
Heatmap of clustering analysis for IPF-related differentially-expressed genes. Legend on the top left indicate log fold change ingenes. (A1–A98 = IPF samples; B1–B 41 = Normal control samples).

The NGS dataset GSE213001 was downloaded from the GEO database, and total of 958 DEGs were screened between IPF and normal control groups with |logFC|  > 0.512 for up regulated genes, |logFC|  < −0.831 for down regulated genes and adjusted *p*  <  0.05, including 479 up regulated DEGs and 479 down regulated DEGs (Supplementary Table S1 and Figure 2). The heatmap of the DEGs has been shown in Figure 3.

### 3.2. GO and Pathway Enrichment Analyses of DEGs

Functional enrichment analyses of the GO terms and REACTOME pathway were performed for both up regulated and down regulated DEGs. To gain insight into the BP, CC, and MF of the DEGs products, we performed a GO enrichment analysis (Supplementary Table S2). In the BP group, the up regulated genes were mainly clustered in response to stimulus and biological regulation, and the down regulated genes were mainly clustered in microtubule-based process and plasma membrane bounded cell projection organization. For the CC group, the up regulated genes were primarily clustered in cell periphery and membrane. The down regulated genes were primarily clustered in cell projection and cytoplasm. The up regulated genes in the MF group were mostly clustered in signaling receptor binding and molecular transducer activity, and the down regulated genes were mostly clustered in tubulin binding and calcium ion binding. The top significantly enriched REACTOME pathways for the DEGs were also displayed by the g:Profiler online software (https://biit.cs.ut.ee/gprofiler/gost, accessed on 23 July 2023) and are presented in Supplementary Table S3. The up regulated genes were associated with GPCR ligand binding and class A/1 (Rhodopsin-like receptors), while the down regulated genes were involved in defective GALNT3 causes HFTC and Lewis blood group biosynthesis.

### 3.3. Construction of the PPI Network and Module Analysis

**Figure 4:**
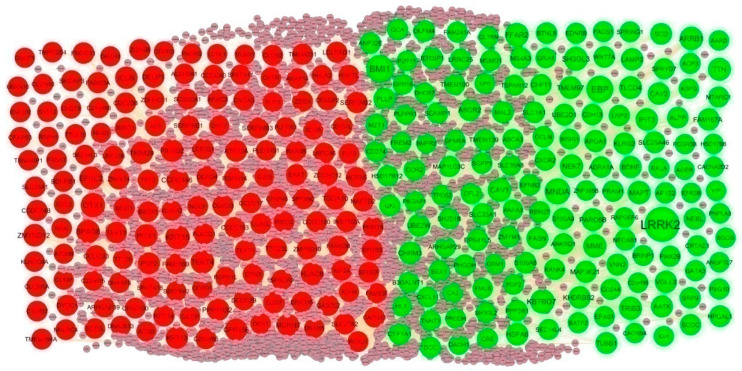
PPI network of DEGs. The PPI network contains 5557 nodes and 9632 edges; green color circle represents up regulated DEGs, and red color circle represents down regulated DEGs.

**Figure 5:**
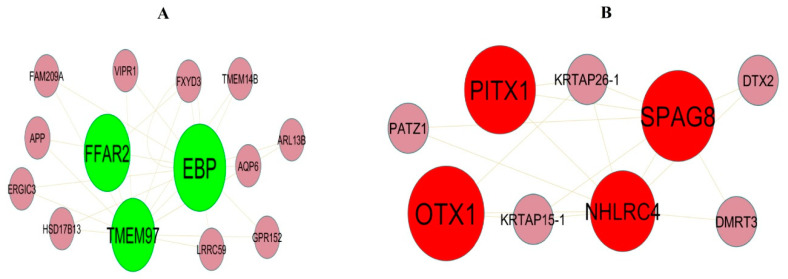
Modular analysis of DEGs. (**A**) Module 1 contains 14 nodes and 26 edges. (**B**) Module 2 contains 9 nodes and 17 edges. The color of each node represents DEGs (green represents up regulated DEGs, and red represents down regulated DEGs).

To investigate the molecular mechanism of IPF from a systematic perspective, a PPI network was constructed to explore the relationship between proteins. The PPI network was constructed by IID for DEGs. There were 5557 nodes and 9632 edges in the visualization network using the Cyctoscape (Figure 4). The genes with high node degree, betweenness, stress, and closeness are considered as hub genes, and include LRRK2, BMI1, EBP, MNDA, KBTBD7, KRT15, OTX1, TEKT4, SPAG8, and EFHC2, and are listed in Supplementary Table S4. According to the node degree of importance, we chose two significant modules from the PPI network complex for further analysis using Cytotype MCODE. Functional enrichment analysis showed that module 1 consisted of 14 nodes and 26 edges (Figure 5A), which are mainly associated with response to stimulus, metabolism of lipids and biological regulation, and that module 2 consisted of 9 nodes and 17 edges (Figure 5B), which are mainly associated with cytoplasm and cell projection. 

### 3.4. Construction of the miRNA-Hub Gene Regulatory Network

**Figure 6:**
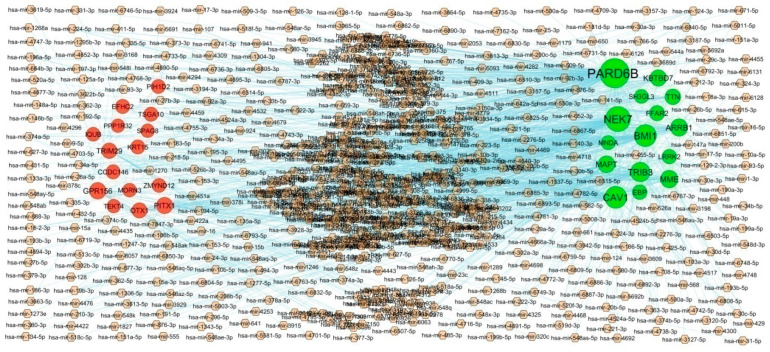
Interaction network between hub genes and targeted miRNAs. Hub genes are presented in green color (up regulated genes) and red color (down regulated genes) circles, whereas small miRNAs are shown in brown color diamond.

To explore the interactions between IPF related hub genes and miRNA, the miRNA-hub gene regulatory network containing 2299 nodes and 10,240 edges was constructed (Figure 6). Of all the nodes, 2023 nodes were miRNAs, while the other 276 nodes were hub genes. The 217 miRNAs (ex: hsa-mir-6830-5p) interacted with PARD6B; 156 miRNAs (ex: hsa-mir-362-3p) interacted with NEK7; 115 miRNAs (ex: hsa-mir-15b) interacted with BMI1; 115 miRNAs (ex: hsa-mir-766-5p) interacted with CAV1; 80 miRNAs (ex: hsa-mir-8057) interacted with TRIB3; 42 miRNAs (ex: hsa-mir-302a-3p) interacted with GPR156; 39 miRNAs (ex: hsa-mir-19b-3p) interacted with PITX1; 27 miRNAs (ex: hsa-mir-4524a-3p) interacted with TRIM29; 24 miRNAs (ex: hsa-mir-1537-5p) interacted with OTX1; 11 miRNAs (ex: hsa-mir-941) interacted with CCDC146 (Supplementary Table S5). Therefore, we speculate that miRNAs might function importantly in the molecular mechanism of IPF.

### 3.5. Construction of the TF-Hub Gene Regulatory Network

**Figure 7:**
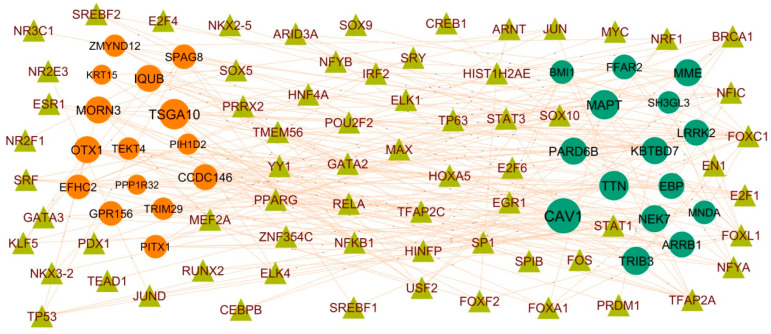
Interaction network between hub genes and targeted TFs. Hub genes are presented in green color (up regulated genes) and red color (down regulated genes) circles, whereas TFs are shown in olive color triangle.

To explore the interactions between IPF related hub genes and TF, the TF-hub gene regulatory network containing 358 nodes and 2169 edges was constructed (Figure 7). Of all the nodes, 79 nodes were TFs, while the other 279 nodes were hub genes. The 16 TFs (ex: NFYA) interacted with CAV1; 11 TFs (ex: SRF) interacted with TTN; 11 TFs (ex: TFAP2A) interacted with MAPT; 10 TFs (ex: EN1) interacted with TRIB3; 9 TFs (ex: ELK4) interacted with NEK7; 12 TFs (ex: JUN) interacted with IQUB; 9 TFs (ex: STAT1) interacted with MORN3; 9 TFs (ex: MEF2A) interacted with OTX1; 9 TFs (ex: TP53) interacted with CCDC146; 8 TFs (ex: NFKB1) interacted with EFHC2 (Supplementary Table S5). Therefore, we speculate that TFs might function importantly in the molecular mechanism of IPF.

### 3.6. Construction of the Protein–Drug Interaction Network

**Figure 8:**
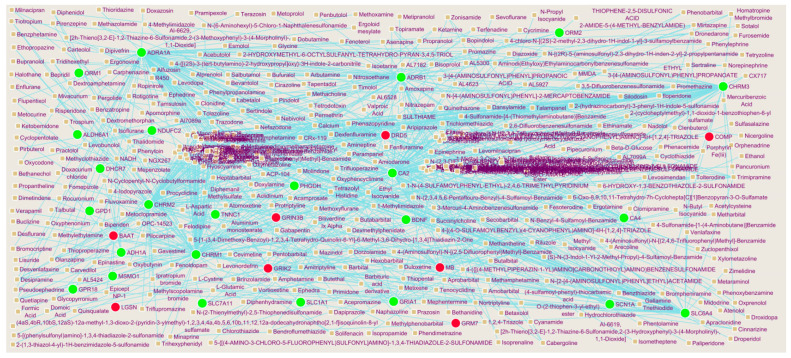
Interaction network between DEGs genes and small drug molecules. Hub genes are presented in green color (up regulated genes) and red color (down regulated genes) circles, whereas small drug molecules are shown in brown circles.

To explore the interactions between IPF related DEGs genes and drugs, the protein–drug interaction network containing 445 nodes and 671 edges was constructed (Figure 8). The 95 drug molecules (ex: Cyclothiazide) interacted with CA2; 89 drug molecules (ex: Tetryzoline) interacted with ADRA1A; 86 drug molecules(ex: Fesoterodine) interacted with CHRM1; 72 drug molecules(ex: Triflupromazine) interacted with CHRM2; 67 drugmolecules (ex: Methacholine) interacted with CHRM3; 26 drug molecules(ex: Rotigotine) interacted with DRD5; 21 drug molecules (ex: Hexobarbital) interacted with GRIK2; 15 drug molecules (ex: Atomoxetine) interacted with GRIN3B; 12 drug molecules (ex: Methylethylamine) interacted with MB; 1 drug molecule (ex: L-Glutamic Acid) interacted with LGSN (Supplementary Table S6).

### 3.7. Receiver Operating Characteristic Curve (ROC) Analysis

**Figure 9:**
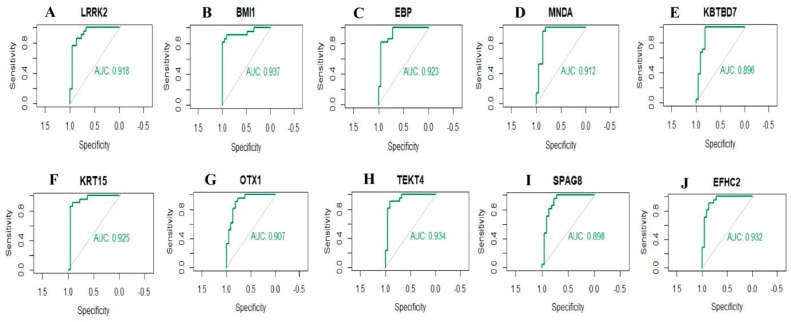
ROC curve analyses of hub genes. (**A**) LRRK2, (**B**) BMI1, (**C**) EBP, (**D**) MNDA, (**E**) KBTBD7, (**F**) KRT15, (**G**) OTX1, (**H**) TEKT4, (**I**) SPAG8, (**J**) EFHC2.

To identify new potential biomarkers for IPF, ROC curves of data derived from IPF and normal controls samples wereanalyzed using the R package. ROC curves were generated, and the area under the curves was used to compare the different hub genes. The results revealed hub genes with predicted AUC > 0.8, namely, LRRK2, BMI1, EBP, MNDA, KBTBD7, KRT15, OTX1, TEKT4, SPAG8, and EFHC2 (Figure 9). This analysis demonstrated that the ten selected hub genes had a diagnostic role in IPF.

## 4. Discussion

Despite advances in adjunctive pharmacotherapy of IPF, it is still the leading threat to human health in lung diseases [51]. Thus, successful screening techniques and accurate diagnosis remain the great challenges for decreasing the incidence of IPF. A bioinformatics and NGS data analysis is an ideal way to comprehensively investigate IPF.By performing DEGs analysis, 479 up regulated and 479 down regulated DEGs were successfully identified (|logFC|  > 0.512 for up regulated genes, |logFC|  < −0.831 for down regulated genes and adjust *p*-value < 0.05), respectively. It should be noted that there were some DEGs that were not shared among IPF networks, but have been proven to play indispensable roles in IPF in recent years. Involvement of highly significant DEGs including SLCO1A2 [52], OLFM4 [53], RTKN2 [54], CYP1A1 [55], and MUC5AC [56] plays a key role in rheumatoid arthritis development.Highly significant DEGs including OLFM4 [57], RTKN2 [58], CYP1A1 [59], MUC5AC [60], CYP2A6 [61], PCK1 [62], and PITX1 [63] have been reported to encourage the development of lung cancer. Recent studies have demonstrated that the OLFM4 [64] is associated with obesity. Highly significant DEGs including OLFM4 [65] and AGTR2 [66] are important in the development of viral respiratory diseases. Highly significant DEGs including CYP1A1 [67] and AGTR2 [68] might be associated with systemic sclerosis. The abnormal expression of CYP1A1 [67] might be related to the progression of systemic lupus erythematosus. The abnormal expression of CYP1A1 [69] and MUC5AC [70] contributes to the progression of pneumonia. Studies had shown that CYP1A1 [71] and AGTR2 [72] are master regulators that are activated in heart failure. Highly significant DEGs including CYP1A1 [73], HHIP (hedgehog interacting protein) [74], MUC5AC [75], and CYP2A6 [76] might be related to the pathophysiology of chronic obstructive pulmonary disease. Previous studies have reported that the CYP1A1 [77], MUC5AC [78], and ATP12A [79] expressions arerelated to the patients with airway inflammation. Studies have found that HHIP (hedgehog interacting protein) [80], MUC5AC [81], CYP2A6 [82], and PCK1 [83] can promote obesity. Highly significant DEGs including AGTR2 [68], MUC5AC [70], and ATP12A [84] are potential targets for IPF therapy. CYP1A1 [85], HHIP (hedgehog interacting protein) [86], AGTR2 [87], CYP2A6 [88], and PCK1 [83] are involved in growth and development of diabetes mellitus. Result suggests that these significant DEGs play a key role in the progression of IPF.

GO and REACTOME enrichment analyses were used to explore the molecular mechanisms of the enriched genes involved in the occurrence and development of IPF. Signaling pathways including GPCR ligand binding [89], neutrophil degranulation [90], immune system [91], metabolism of lipids [92], and signal transduction [93] play an important role in the IPF. Enriched genes including MAP3K15 [94], PRTN3 [95], CX3CR1 [96], AGRP(agouti related neuropeptide) [97], MPO (myeloperoxidase) [98], CD5L [99], S100A8 [100], NPR3 [101], VEGFD (vascular endothelial growth factor D) [102], CXCL11 [103], IL1A [104], CBS (cystathionine beta-synthase) [105], WNT7A [106], SCD (stearoyl-CoA desaturase) [107], LRP2 [108], SLC6A4 [109], BDNF (brain derived neurotrophic factor) [110], CXCL10 [111], ANGPTL7 [112], S100A9 [113], NPY1R [114], IL1B [115], GPIHBP1 [116], CYP1B1 [117], CD36 [118], MACROD2 [119], TRIB3 [120], SPX (spexin hormone) [121], PCSK9 [122], GPD1 [123], CDH13 [124], FFAR4 [125], FGF2 [126], FASN (fatty acid synthase) [127], DGAT2 [128], DACH1 [129], PNPLA3 [130], FGF9 [131], SLC7A11 [132], CLIC5 [133], VIP (vasoactive intestinal peptide) [134], SMAD6 [135], BMPR2 [136], APOA1 [137], INSIG1 [138], TLR3 [139], NLRP12 [140], ADRB1 [141], TLR8 [142], GATA3 [143], CCR2 [144], TLR7 [145], CCRL2 [146], BMPER (BMP binding endothelial regulator) [147], CAV1 [148], TFPI (tissue factor pathway inhibitor) [149], FADS1 [150], SUCNR1 [151], CADM2 [152], SLC19A3 [153], SGCG (sarcoglycan gamma) [154], ADH1B [155], NEGR1 [156], HSD17B12 [157], OXTR (oxytocin receptor) [158] and ANKK1 [159] play a key role in obesity. Enriched genes including MAP3K15 [94], CX3CR1 [160], S100A12 [161], PF4 [162], FFAR2 [163], MPO (myeloperoxidase) [98], HMGCS2 [164], F11 [165], S100A8 [100], GRIA1 [166], NPR3 [167], CXCL11 [103], CBS (cystathionine beta-synthase) [105], WNT7A [106], AQP4 [168], SCD (stearoyl-CoA desaturase) [169], SLC6A4 [170], CXCL10 [171], S100A9 [172], NPY1R [173], IL1B [174], CXCR1 [175], CXCR2 [176], GPIHBP1 [177], WNT3A [178], APOH (apolipoprotein H) [179], CHRM3 [180], CD36 [181], TRIB3 [182], PCSK9 [183], ACVR1C [184], GPD1 [123], FFAR4 [185], GPX3 [186], FGF2 [187], FASN (fatty acid synthase) [188], DGAT2 [189], DACH1 [190], PNPLA3 [191], FGF9 [192], SLC7A11 [193], VIP (vasoactive intestinal peptide) [194], KL (klotho) [195], UBE2D1 [196], APOA1 [197], RASGRF1 [198], LRRK2 [199], TLR3 [200], OCLN (occludin) [201], SLC22A3 [202], LIFR (LIF receptor subunit alpha) [203], TLR8 [142], GATA3 [204], CCR2 [205], NEK7 [206], CD274 [207], TLR7 [208], CCRL2 [146], EFNB2 [209], CAV1 [210], TRPC3 [211], DLL4 [212], ANXA3 [213], TFPI (tissue factor pathway inhibitor) [214], FADS1 [215], GPER1 [216], SUCNR1 [217], CADM2 [218], SLC19A3 [219], ADH1B [220], NEGR1 [156], HSD17B12 [157], KIF6 [221], UCN3 [222], ANKK1 [223], AQP5 [224], and HCN4 [225] are a key regulators of diabetes mellitus. Enriched genes includingPRTN3 [226], CX3CR1 [227], S100A12 [228], CSF2 [229], FGG (fibrinogen gamma chain) [230], LHX9 [231], MPO (myeloperoxidase) [232], F11 [233], S100A8 [234], CXCL11 [235], BPI (bactericidal permeability increasing protein) [236], BDNF (brain derived neurotrophic factor) [237], CXCL10 [238], S100A9 [239], IL1B [240], CXCR1 [241], CXCR2 [242], CYP1B1 [243], EDNRB (endothelin receptor type B) [244], CEBPA (CCAAT enhancer binding protein alpha) [245], CDH13 [246], GPX3 [247], FGF2 [248], SHH (sonic hedgehog signaling molecule) [249], VIP (vasoactive intestinal peptide) [250], KL (klotho) [251], SMAD6 [252], BMPR2 [253], APOA1 [254], TLR3 [255], GATA3 [256], CCR2 [257], CAV1 [258], TRPC3 [259], EPAS1 [260], SIGLEC14 [261], MAPK15 [262], DNAH5 [263], and AQP5 [264] were altered expressed in chronic obstructive pulmonary disease. Enriched genes includingDEFA3 [265], CX3CR1 [266], S100A12 [161], TUBB1 [267], ANKRD1 [268], ADRA1A [269], FGG (fibrinogen gamma chain) [270], AGER (advanced glycosylation end-product specific receptor) [271], PF4 [272], FFAR2 [273], MPO (myeloperoxidase) [274], CD5L [275], HMGCS2 [164], RXFP1 [276], F11 [277], S100A8 [278], PGLYRP1 [279], VEGFD (vascular endothelial growth factor D) [280], CHRM2 [281], CBS (cystathionine beta-synthase) [282], BPI (bactericidal permeability increasing protein) [283], LRP2 [284], BDNF (brain derived neurotrophic factor) [285], GCOM1 [286], CXCL10 [287], ANGPTL7 [288], PRODH (proline dehydrogenase 1) [289], P2RY1 [290], LRRN4 [291], S100A9 [292] CXCR1 [293], CXCR2 [293], GPIHBP1 [294], TNNT1 [295], WNT3A [296], BMI1 [297], CYP1B1 [298], FCN3 [299], TTN (titin) [300], STC1 [301], CD36 [302], MYZAP (myocardial zonula adherens protein) [303], TRIB3 [304], GPR18 [305], TNNC1 [306], SPX (spexin hormone) [121], SYNPO2L [307],PCSK9 [308], GPD1 [309], FFAR4 [310], GPX3 [311], FGF2 [187], ACKR4 [312], NDUFC2 [313], KBTBD7 [314], SHH (sonic hedgehog signaling molecule) [315], DACH1 [316], PNPLA3 [317], FGF9 [192], SLC7A11 [193], SGPP1 [318], VIP (vasoactive intestinal peptide) [319], KCNJ2 [320], KL (klotho) [321], SMAD6 [135], BMPR2 [322], APOA1 [323], CALCRL (calcitonin receptor like receptor) [324], INSIG1 [325], RASGRF1 [198],LRRK2 [326], TLR3 [327], ADRB1 [328], SLC22A3 [329], CA2 [330], SNX10 [331], LIFR (LIF receptor subunit alpha) [332], TLR8 [333], CMPK2 [334], GATA3 [335], RSPO2 [336], CCR2 [205], NEK7 [337], TLR7 [338], BEX1 [339], EFNB2 [340], CAV1 [341], ARRB1 [342], TRPC3 [343], CR1 [344], PEG10 [345], DLL4 [346], MEFV (MEFV innate immuity regulator, pyrin) [347], TFPI (tissue factor pathway inhibitor) [348], EPAS1 [349], FADS1 [215], DKK2 [350], CACNA2D2 [351], DPP6 [352], KCNA4 [353], PCDH17 [354], SUSD2 [355], PHACTR2 [356], DNAH9 [357], DNAH11 [358], CFAP45 [359], DNAH5 [360], FOXJ1 [361], MNS1 [299], KIF6 [221], DRD5 [362], UCN3 [363], OXTR (oxytocin receptor) [364], ANKK1 [365], and HCN4 [366] were associated with heart failure. Researchdemonstrated that enriched genes including PI3 [367], CX3CR1 [368], S100A12 [369], MPO (myeloperoxidase) [370], CD5L [371], S100A8 [372], CXCL11 [373], BPI (bactericidal permeability increasing protein) [374], AQP4 [375], BDNF (brain derived neurotrophic factor) [376], CXCL10 [377], CCL8 [378], S100A9 [379], IL1B [240], CXCR1 [380], CXCR2 [381], ABCA3 [382], GPR18 [383], VIP (vasoactive intestinal peptide) [384], KL (klotho) [385], TLR3 [386], NLRP12 [387], GATA3 [388], CCR2 [389], TLR7 [390], CAV1 [391], CR1 [392], DLL4 [393], and AQP5 [394] might be potential therapeutic targets for airway inflammation. Enriched genes includingCX3CR1 [395], S100A12 [396], MPO (myeloperoxidase) [397], RXFP1 [398], S100A8 [399], CXCL11 [373], CBS (cystathionine beta-synthase) [400], WNT7A [401], BDNF (brain derived neurotrophic factor) [402], CXCL10 [403], CCL8 [404], FCGR3B [405], S100A9 [406], IL1B [407], CXCR2 [408], WNT3A [409], BMI1 [410], STC1 [411], ABCA3 [412], CD36 [413], TRIB3 [414], GPX3 [415],FGF2 [416], FASN (fatty acid synthase) [417], SHH (sonic hedgehog signaling molecule) [418], DACH1 [419], FGF9 [420], SLC7A11 [421], VIP (vasoactive intestinal peptide) [422], KL (klotho) [423], BMPR2 [424], APOA1 [425], LRRK2 [426], TLR3 [427], GATA3 [428], RSPO2 [429], CCR2 [430], NEK7 [431], BMPER (BMP binding endothelial regulator) [432], CAV1 [433], CR1 [434], TFPI (tissue factor pathway inhibitor) [435], AP1S2 [436], FOXJ1 [437], AQP5 [438], MUC16 [439], and MUC4 [440] have been found in a IPF. Enriched genes including CX3CR1 [441], S100A12 [442], PF4 [443], MPO (myeloperoxidase) [444], WNT7A [445], SLC6A4 [446], BDNF (brain derived neurotrophic factor) [447], CXCL10 [448], NEK7 [449], CYP1B1 [450], ABCA3 [451], TRIB3 [452], PCSK9 [453], FGF2 [454], ACKR4 [455], FASN (fatty acid synthase) [456], VIP (vasoactive intestinal peptide) [457], KL (klotho) [458], BMPR2 [459], APOA1 [323], TLR3 [460], CCR2 [461], TLR7 [462], CAV1 [463], WWC2 [464], TFPI (tissue factor pathway inhibitor) [465], EPAS1 [466], and CCDC40 [467] were identified to be associated with pulmonary hypertension. A previous study reported that the enriched genes includingCX3CR1 [468], CSF2 [469], CLDN18 [470], TRIM58 [471], PF4 [472], FFAR2 [473], MPO (myeloperoxidase) [474], CD5L [475], SH3GL2 [476], ITGA2B [477], S100A8 [478], VEGFD (vascular endothelial growth factor D) [479], CXCL11 [480], IL1A [481], WNT7A [482], SSTR1 [483], AQP4 [484], SCD (stearoyl-CoA desaturase) [485], SLC6A4 [486], BDNF (brain derived neurotrophic factor) [487], CXCL10 [488], ODAM (odontogenic, ameloblast associated) [489], CASP5 [490], CCL8 [491], TMEM100 [492], S100A9 [493], IL1B [494], CXCR1 [495], CXCR2 [496], WNT3A [497], BMI1 [498], CYP1B1 [499], FCN3 [500], TTN (titin) [501], SHISA3 [502], AZGP1 [503], ABCA3 [504], CD36 [505], EDNRB (endothelin receptor type B) [506], BTNL9 [507], CEBPA (CCAAT enhancer binding protein alpha) [508], TRIB3 [509], TNNC1 [510], PCSK9 [511], P2RY13 [512], KITLG (KIT ligand) [513], CDH13 [514], GPX3 [515], FGF2 [416], FUT7 [516], FASN (fatty acid synthase) [517], NKD1 [518], FOXD1 [519], SLC1A1 [520], SHH (sonic hedgehog signaling molecule) [521], DACH1 [522], FGF9 [523], SLC7A11 [524], CLIC5 [525], MGAT3 [526], HSPA6 [527], TSPAN12 [528], SCAI (suppressor of cancer cell invasion) [529], VIP (vasoactive intestinal peptide) [530], SH3GL3 [531], KCNJ2 [532], KL (klotho) [533], UBE2D1 [534], SMAD6 [535], BMPR2 [536], APOA1 [537], TGFBR3 [538], RASGRF1 [539], LRRK2 [540], ATP8A2 [541], TLR3 [542], OCLN (occludin) [543], EMP2 [544], MNDA (myeloid cell nuclear differentiation antigen) [545], TLR8 [546], GATA3 [547], RSPO2 [548], CCR2 [549], EPB41L5 [550], CD274 [551], DDIAS (DNA damage induced apoptosis suppressor) [552], TLR7 [553], CCRL2 [554], BMPER (BMP binding endothelial regulator) [555], DUSP26 [556], CCBE1 [557], FZD8 [558], CAV1 [559], ARRB1 [560], CR1 [561], WWC2 [562], DLL4 [563], ANXA3 [564], EPAS1 [565], FADS1 [566], DKK2 [567], GPER1 [568], CADM2 [569], PARD6B [570], CACNA2D2 [571], ATP8A1 [572], PDZD2 [573], STXBP6 [574], ADH1A [575], GCA (grancalcin) [576], SUSD2 [577], EDIL3 [578], PHACTR2 [579], DNAH10 [580], CCDC65 [581], SPAG6 [582], MAPK15 [583], ENKUR (enkurin, TRPC channel interacting protein) [584], DNAH5 [585], PIERCE1 [586], TPPP3 [587], TTC21A [588], DLEC1 [589], SRCIN1 [590], PROM1 [591], AQP5 [592], SYT13 [593], TTC21A [594], SPTBN2 [595], MUC13 [596], MUC16 [597], and MUC4 [598] have been shown to be biomarkers of lung cancer. The altered expression of enriched genes including CX3CR1 [599], S100A12 [600], PF4 [601], MPO (myeloperoxidase) [602], RXFP1 [603], S100A8 [604], VEGFD (vascular endothelial growth factor D) [605], CXCL11 [606], IL1A [607], BPI (bactericidal permeability increasing protein) [608], SLC6A4 [609], CXCL10 [610], FCGR3B [611], S100A9 [612], IL1B [613], CXCR2 [614], CTNND2 [615], CD36 [616], PCSK9 [617], FGF2 [618], SHH (sonic hedgehog signaling molecule) [619], KL (klotho) [620], BMPR2 [621], TLR8 [622], GATA3 [623], CCR2 [624], TLR7 [625], CAV1 [626], TFPI (tissue factor pathway inhibitor) [627], and SPAG17 [628] have been proposed as novel biomarkers for systemic sclerosis. A previous study found that enriched genes including CX3CR1 [629], S100A12 [630], MPO (myeloperoxidase) [631], CD5L [632], F11 [633], S100A8 [634], BPI (bactericidal permeability increasing protein) [635], AQP4 [636], MME (membrane metalloendopeptidase) [637], BDNF (brain derived neurotrophic factor) [638], CXCL10 [639], RNASE2 [640], FCGR3B [641], S100A9 [642], GPIHBP1 [643], AFF3 [644], APOH (apolipoprotein H) [645], FCN3 [646], PCSK9 [308], LILRA2 [647], APOA1 [648], LRRK2 [649], CD244 [650], TLR3 [651], TLR8 [652], GATA3 [653], CCR2 [654], IFIT3 [655], NEK7 [656], TLR7 [657], CAV1 [658], CR1 [659], TFPI (tissue factor pathway inhibitor) [660], GPER1 [661], SIGLEC14 [662], FOXJ1 [663], GABRP (gamma-aminobutyric acid type A receptor subunit pi) [664], and TSGA10 [665] play a certain role in systemic lupus erythematosus.Altered expression of enriched genes including CX3CR1 [666], S100A12 [667], CSF2 [668], MPO (myeloperoxidase) [669], CD5L [670], F11 [671], S100A8 [672], PGLYRP1 [673], VEGFD (vascular endothelial growth factor D) [674], CXCL11 [373], BPI (bactericidal permeability increasing protein) [675], CXCL10 [676], S100A9 [677], CXCR1 [678], CXCR2 [679], ABCA3 [680], CD36 [681], SHH (sonic hedgehog signaling molecule) [682], TLR3 [683], CLEC4D [684], CCR2 [685], NEK7 [686], TLR7 [687], CCRL2 [688], and CAV1 [689] are associated with pneumonia progression. Enriched genes including CX3CR1 [666], CD177 [690], PF4 [691], FFAR2 [692], MPO (myeloperoxidase) [693], F11 [694], S100A8 [695], VEGFD (vascular endothelial growth factor D) [674], IL1A [696], BPI (bactericidal permeability increasing protein) [697], AQP4 [698], BDNF (brain derived neurotrophic factor) [699], CXCL10 [700], RNASE2 [701], FCGR3B [702], S100A9 [703], IL1B [704], CXCR2 [705], GPIHBP1 [294], CD36 [706], TRIB3 [707], PCSK9 [708], FGF2 [709], FASN (fatty acid synthase) [710], PNPLA3 [711], HSPA6 [712], VIP (vasoactive intestinal peptide) [713], TLR3 [683], ADRB1 [328], SPOCK2 [714], TLR8 [715], CCR2 [716], IFIT3 [717], NEK7 [718], TLR7 [687], EFNB2 [719], CAV1 [720], CR1 [721], and AQP5 [722] are positively correlated with the severity of viral respiratory diseases, suggesting theirpotential as a biomarkers for viral respiratory diseases. Previous study confirmed that enriched genes including CX3CR1 [723], S100A12 [724], CD177 [725], PF4 [726], MPO (myeloperoxidase) [727], CD5L [728], F11 [729], S100A8 [730], PGLYRP1 [731], GPR15 [732], BPI (bactericidal permeability increasing protein) [733], AQP4 [734], BDNF (brain derived neurotrophic factor) [735], CXCL10 [736], FCGR3B [737], S100A9 [738], IL1B [739], CXCR1 [740], CXCR2 [741], AFF3 [742], WNT3A [743], FCN3 [744], AZGP1 [745], CD36 [746], PCSK9 [747], GPX3 [748], FGF2 [749], SHH (sonic hedgehog signaling molecule) [750], SLC7A11 [751], VIP (vasoactive intestinal peptide) [752], KL (klotho) [753], APOA1 [754], RASGRF1 [755], CD244 [756], TLR3 [757], NLRP12 [758], SNX10 [759], TLR8 [760], GATA3 [761], CCR2 [762], TLR7 [763], CCRL2 [764], EFNB2 [765], FZD8 [766], CAV1 [767], CR1 [768], MEFV (MEFV innate immunity regulator, pyrin) [769], SUCNR1 [770], GCA (grancalcin) [771] and, FOXJ1 [663] were observed in rheumatoid arthritis. Enriched genes including S100A12 [772], MPO (myeloperoxidase) [773], S100A8 [774], CXCL10 [775], S100A9 [774], CD244 [775], CD244 [776],and TLR7 [777] have been linked to dermatomyositis. MPO (myeloperoxidase) [778] participated in the regulation of mixed connective tissue disease progression. Enriched genes including MPO (myeloperoxidase) [779], CXCL11 [780], IL1A [781], CXCL10 [782], FCGR3B [783], IL1B [784], TTN (titin) [300], BATF2 [785], VIP (vasoactive intestinal peptide) [786], TLR3 [787], CCR2 [788], TLR7 [789], and CR1 [790] wereclosely related to sarcoidosis. A study indicates that enriched genes including MPO (myeloperoxidase) [791], RXFP1 [792], CXCL11 [793], CTNND2 [615], BMPR2 [794], TLR3 [795], CCR2 [796], and CAV1 [797] are altered expressed in scleroderma. Enriched genes including CXCL11 [798] and CD244 [776] are considered potential biomarkers for polymyositis. Study demonstrated that enriched genes including IL1B [799], CXCR1 [800], FFAR4 [801], VIP (vasoactive intestinal peptide) [802], and GATA3 [803] have been identified as a key candidate genes in patients with gastroesophageal reflux disease. Therefore, it is necessary to perform GO term and pathway enrichment analysis in order to understand the interactions between DEGs and the associated biological processes. In this investigation, we identified some enriched genes for which the functions in IPF have not been completely characterized, suggesting their potential as biomarkers for this disease., There may be a relationship between these enriched genes in other diseases, includingdiabetes mellitus, obesity, chronic obstructive pulmonary disease, heart failure, airway inflammation, pulmonary hypertension, lung cancer, systemic sclerosis, systemic lupus erythematosus, pneumonia, viral respiratory diseases, rheumatoid arthritis, mixed connective tissue disease, sarcoidosis, polymyositis, and gastroesophageal reflux disease.

Hub genes in the molecular pathogenesis of IPF were identified by using the IID software of Cytoscape. Studies have shown that hub genes including LRRK2 [199] and FFAR2 [163] have been reported to be correlated with prognosis in a diabetes mellitus. Hub genes including LRRK2 [326], BMI1 [297], KBTBD7 [314], and FFAR2 [273] were associated with the risk of heart failure. Hub genes including LRRK2 [426] and BMI1 [410] are highly associated with IPF. Hub genes including LRRK2 [540], BMI1 [398], MNDA (myeloid cell nuclear differentiation antigen) [545], OTX1 [804], FFAR2 [473], and PITX1 [63] play a significant role in lung cancer progression. Hub gene LRRK2 [649] was identified as being associated with increased risk of systemic lupus erythematosus. A study has shown that hub gene FFAR2 [692] was significantly associated with viral respiratory diseases. There is no research showing that novel hub genes including EBP (EBP cholestenol delta-isomerase), KRT15, TEKT4, SPAG8, EFHC2, TMEM97, and NHLRC4 are related to IPF. This finding is consistent with our results. 

miRNA-hub gene regulatory network and TF-hub gene regulatory network can be regarded as key to the understanding of pathogenesis of IPF and might also lead to new therapeutic approaches. Studies have shown that biomarkers including PARD6B [570], BMI1 [498], CAV1 [559], TRIB3 [509], TTN (titin) [501], PITX1 [63],TRIM29 [805], OTX1 [804], NFYA (nuclear transcription factor Y subunit alpha) [806], TFAP2A [807], JUN (Jun proto-oncogene, AP-1 transcription factor subunit) [808], STAT1 [809], TP53 [810], and NFKB1 [811] might act as bifunctional mediators to lung cancer. Studies have shown that biomarkers including NEK7 [206], CAV1 [210], TRIB3 [182], STAT1 [812], TP53 [813], and NFKB1 [814] are closely related to the development of diabetes mellitus.Some papers reported that NEK7 [337], BMI1 [297], CAV1 [341], TRIB3 [304], TTN (titin) [300],hsa-mir-19b-3p [815], hsa-mir-941 [816], NFYA (nuclear transcription factor Y subunit alpha) [817], STAT1 [818], MEF2A [819], TP53 [820], and NFKB1 [821] have been revealed to be associated with heart failure. Previous studies have demonstrated that biomarkers including NEK7 [431], BMI1 [410], CAV1 [433], TRIB3 [414], SRF (serum response factor) [822], STAT1 [823], and TP53 [824] are believed to be related to the occurrence of IPF. Biomarkers including NEK7 [656], CAV1 [658], TSGA10 [665], STAT1 [825], TP53 [826], and NFKB1 [827] are reported to be associated with systemic lupus erythematosus. Biomarkers including NEK7 [686] and CAV1 [689] were associated with the risk of pneumonia. Biomarkers includingNEK7 [718], CAV1 [720], TRIB3 [707], has-mir-8057 [828], hsa-mir-1537-5p [829], STAT1 [830], and TP53 [831] are highly associated with viral respiratory diseases.Biomarkers including NEK7 [449], TRIB3 [452], CAV1 [463],NFYA (nuclear transcription factor Y subunit alpha) [817], STAT1 [832], and TP53 [833] played an important role in the pulmonary hypertension. Biomarkers including CAV1 [148], TRIB3 [120], STAT1 [834], TP53 [835], and NFKB1 [836] have been reported in obesity. Biomarkers including CAV1 [258], hsa-mir-19b-3p [837], SRF (serum response factor) [838], and TP53 [839] play an important role in the regulation of chronic obstructive pulmonary disease. Biomarkers including CAV1 [391], STAT1 [840], and NFKB1 [841] play a major regulatory role in the development of airway inflammation. CAV1 [626], TFAP2A [842],TP53 [843], and NFKB1 [844] are the most specific biomarkers for the detection systemic sclerosis. Biomarkers including CAV1 [767], hsa-mir-19b-3p [845], JUN (Jun proto-oncogene, AP-1 transcription factor subunit) [846], STAT1 [847], TP53 [848], and NFKB1 [849] were largely detected in rheumatoid arthritis. CAV1 [797] could be a potential biomarker for scleroderma. Biomarkers including TTN (titin) [300] and STAT1 [850] are widely involved in sarcoidosis. Biomarker STAT1 [851] was implicated in the pathology ofdermatomyositis. MAPT, GPR156, CCDC146, IQUB, MORN3, hsa-mir-6830-5p, hsa-mir-362-3p, hsa-mir-15b, hsa-mir-766-5p, hsa-mir-302a-3p, hsa-mir-4524a-3p, EN1, and ELK4 are all new biomarkers for the development of IPF and its complications. Our study showed that hub genes, miRNA, and TFs regulated in patients, although further research is needed to explore the underlying molecular mechanism.

To date, there is no effective drug for the treatment of IPF. Few investigations have indicated that some drugs, including pirfenidone and nintedanib, can improve the outcomes of IPF [852]. Herein, we predicted a set of drugs that could target the predicted protein–drug interaction network by using NetworkAnalyst database. Cyclothiazide, tetryzoline, fesoterodine, triflupromazine, methacholine, rotigotine, hexobarbital, atomoxetine, methylethylamine, and L-glutamic acid were the drugs with the highest number of target genes. The predicted genes might be efficient in the treatment of IPF, which needs further experimental screening and validation.

In conclusion, in the present study, we conducted a thorough bioinformatics analysis of DEGs by GSE213001 NGS data screening and identified several genes implicated in the development and progression of IPF. A total of 958 DEGs were identified, of which LRRK2, BMI1, EBP, MNDA, KBTBD7, KRT15, OTX1, TEKT4, SPAG8, and EFHC2 are probable hub genes of IPF. This investigation reveals a series of valuable genes for further investigation into the non-invasive diagnosis and targeted therapy of IPF. However, bioinformatics analyses merely indicate a general direction for further investigation. To confirm the functions of hub genes in IPF, molecular biology experiments are required.

## Data Availability

The datasets supporting the conclusions of this article are available in the GEO (Gene Expression Omnibus) (https://www.ncbi.nlm.nih.gov/geo/, accessed on 1 November 2023) repository. [(GSE213001) https://www.ncbi.nlm.nih.gov/geo/query/acc.cgi?acc=GSE213001, accessed on 1 November 2023].

## Supplementary Materials

The following supporting information can be downloaded at: https://www.mdpi.com/article/10.3390/biomedicines11123109/s1, Table S1 The statistical metrics for key differentially expressed genes (DEGs). Table S2 The enriched GO terms of the up and down regulated differentially expressed genes. Table S3 The enriched pathway terms of the up and down regulated differentially expressed. Table S4 Topology table for up and down regulated genes. Table S5 Candidate miRNA and TFs targeted by the hub genes. Table S6 Candidate drugs targeted by the hub genes.

## References

[1] Hutchinson J., Fogarty A., Hubbard R., McKeever T. (2015). Global incidence and mortality of idiopathic pulmonary fibrosis: A systematic review. Eur. Respir. J..

[2] Chanda D., Otoupalova E., Smith S.R., Volckaert T., De Langhe S.P., Thannickal V.J. (2019). Developmental pathways in the pathogenesis of lung fibrosis. Mol. Asp. Med..

[3] King T.E., Pardo A., Selman M. (2011). Idiopathic pulmonary fibrosis. Lancet.

[4] Pardo A., Selman M. (2021). The Interplay of the Genetic Architecture, Aging, and Environmental Factors in the Pathogenesis of Idiopathic Pulmonary Fibrosis. Am. J. Respir. Cell Mol. Biol..

[5] Robledo G.C., Hernández M.Y.J., Lucas S.A.G., Delgado F.R.C. (2022). Combined Pulmonary Fibrosis and Emphysema with Pulmonary Hypertension: Cases Report. Curr. Probl. Cardiol..

[6] Ballester B., Milara J., Cortijo J. (2019). Idiopathic Pulmonary Fibrosis and Lung Cancer: Mechanisms and Molecular Targets. Int. J. Mol. Sci..

[7] Bai L., Zhang L., Pan T., Wang W., Wang D., Turner C., Zhou X., He H. (2021). Idiopathic pulmonary fibrosis and diabetes mellitus: A meta-analysis and systematic review. Respir. Res..

[8] High W.A., Cohen J.B., Murphy B.A., Costner M.I. (2003). Fatal interstitial pulmonary fibrosis in anti-Jo-1-negative amyopathic dermatomyositis. J. Am. Acad. Dermatol..

[9] Weidensaul D., Imam T., Holyst M.M., King P.D., McMurray R.W. (1995). Polymyositis, pulmonary fibrosis, and hepatitis C. Arthritis Rheum..

[10] Mattoo H., Pillai S. (2021). Idiopathic pulmonary fibrosis and systemic sclerosis: Pathogenic mechanisms and therapeutic interventions. Cell. Mol. Life Sci..

[11] Gunnarsson R., El-Hage F., Aaløkken T.M., Reiseter S., Lund M.B., Garen T., Molberg Ø., Norwegian MCTD study group (2016). Associations between anti-Ro52 antibodies and lung fibrosis in mixed connective tissue disease. Rheumatology.

[12] Şenkal N., Kıyan E., Demir A.A., Yalçınkaya Y., Gül A., İnanç M., Öçal M.L., Esen B.A. (2022). Interstitial lung disease in patients with systemic lupus erythematosus: A cohort study. Turk. J. Med. Sci..

[13] Kadura S., Raghu G. (2021). Rheumatoid arthritis-interstitial lung disease: Manifestations and current concepts in pathogenesis and management. Eur. Respir. Rev..

[14] Patterson K.C., Strek M.E. (2013). Pulmonary fibrosis in sarcoidosis. Clinical features and outcomes. Ann. Am. Thorac. Soc..

[15] White B. (2002). Evaluation and management of pulmonary fibrosis in scleroderma. Curr. Rheumatol. Rep..

[16] Miądlikowska E., Rzepka-Wrona P., Miłkowska-Dymanowska J., Białas A.J., Piotrowski W.J. (2021). Review: Serum Biomarkers of Lung Fibrosis in Interstitial Pneumonia with Autoimmune Features-What Do We Already Know?. J. Clin. Med..

[17] Puukila S., Lawrence M.D., De Pasquale C.G., Bersten A.D., Bihari S., McEvoy-May J., Nemec-Bakk A., Dixon D.L. (2023). Monocyte chemotactic protein (MCP)-1 (CCL2) and its receptor (CCR2) are elevated in chronic heart failure facilitating lung monocyte infiltration and differentiation which may contribute to lung fibrosis. Cytokine.

[18] Guo X., Sunil C., Qian G. (2022). Obesity and the Development of Lung Fibrosis. Front. Pharmacol..

[19] Wendisch D., Dietrich O., Mari T., von Stillfried S., Ibarra I.L., Mittermaier M., Mache C., Chua R.L., Knoll R., Timm S. (2021). SARS-CoV-2 infection triggers profibrotic macrophage responses and lung fibrosis. Cell.

[20] Ruaro B., Pozzan R., Confalonieri P., Tavano S., Hughes M., Matucci Cerinic M., Baratella E., Zanatta E., Lerda S., Geri P. (2022). Gastroesophageal Reflux Disease in Idiopathic Pulmonary Fibrosis: Viewer or Actor? To Treat or Not to Treat?. Pharmaceuticals.

[21] Zhao H., Dennery P.A., Yao H. (2018). Metabolic reprogramming in the pathogenesis of chronic lung diseases, including BPD, COPD, and pulmonary fibrosis. Am. J. Physiol. Lung Cell. Mol. Physiol..

[22] Shin I.S., Lee M.Y., Jeon W.Y., Kim J.C., Shin H.K. (2013). Ojeok-san, a traditional Korean herbal medicine attenuates airway inflammation and pulmonary fibrosis induced by repeated ovalbumin challenge. J. Ethnopharmacol..

[23] Ganekal P., Vastrad B., Kavatagimath S., Vastrad C., Kotrashetti S. (2023). Bioinformatics and Next-Generation Data Analysis for Identification of Genes and Molecular Pathways Involved in Subjects with Diabetes and Obesity. Medicina.

[24] Alur V., Raju V., Vastrad B., Vastrad C., Kavatagimath S., Kotturshetti S. (2023). Bioinformatics Analysis of Next Generation Sequencing Data Identifies Molecular Biomarkers Associated with Type 2 Diabetes Mellitus. Clin. Med. Insights Endocrinol. Diabetes.

[25] Hojo S., Fujita J., Yamadori I., Kamei T., Yoshinouchi T., Ohtsuki Y., Okada H., Bandoh S., Yamaji Y., Takahara J. (1998). Heterogeneous point mutations of the p53 gene in pulmonary fibrosis. Eur. Respir. J..

[26] Du H., Guo Y., Ma D., Tang K., Cai D., Luo Y., Xie C. (2018). A case report of heterozygous TINF2 gene mutation associated with pulmonary fibrosis in a patient with dyskeratosis congenita. Medicine.

[27] Pulkkinen V., Bruce S., Rintahaka J., Hodgson U., Laitinen T., Alenius H., Kinnula V.L., Myllärniemi M., Matikainen S., Kere J. (2010). ELMOD2, a candidate gene for idiopathic pulmonary fibrosis, regulates antiviral responses. FASEB J..

[28] Mushiroda T., Wattanapokayakit S., Takahashi A., Nukiwa T., Kudoh S., Ogura T., Taniguchi H., Kubo M., Kamatani N., Nakamura Y. (2008). A genome-wide association study identifies an association of a common variant in TERT with susceptibility to idiopathic pulmonary fibrosis. J. Med. Genet..

[29] Campo I., Zorzetto M., Mariani F., Kadija Z., Morbini P., Dore R., Kaltenborn E., Frixel S., Zarbock R., Liebisch G. (2014). A large kindred of pulmonary fibrosis associated with a novel ABCA3 gene variant. Respir. Res..

[30] Kang H. (2017). Role of MicroRNAs in TGF-β Signaling Pathway-Mediated Pulmonary Fibrosis. Int. J. Mol. Sci..

[31] Zhang Y., Lu W., Zhang X., Lu J., Xu S., Chen S., Zhong Z., Zhou T., Wang Q., Chen J. (2019). Cryptotanshinone protects against pulmonary fibrosis through inhibiting Smad and STAT3 signaling pathways. Pharmacol. Res..

[32] Goda C., Balli D., Black M., Milewski D., Le T., Ustiyan V., Ren X., Kalinichenko V.V., Kalin T.V. (2020). Loss of FOXM1 in macrophages promotes pulmonary fibrosis by activating p38 MAPK signaling pathway. PLoS Genet..

[33] Wan X., Chen S., Li P., Zhao T., Xie S., Fang Y. (2022). Sinensetin protects against pulmonary fibrosis via inhibiting Wnt/β-Catenin signaling pathway. Tissue Cell..

[34] Li H., Wang Z., Zhang J., Wang Y., Yu C., Zhang J., Song X., Lv C. (2018). Feifukang ameliorates pulmonary fibrosis by inhibiting JAK-STAT signaling pathway. BMC Complement. Altern. Med..

[35] Clough E., Barrett T. (2016). The Gene Expression Omnibus Database. Methods Mol. Biol..

[36] Love M.I., Huber W., Anders S. (2014). Moderated estimation of fold change and dispersion for RNA-seq data with DESeq2. Genome Biol..

[37] Solari A., Goeman J.J. (2017). Minimally adaptive BH: A tiny but uniform improvement of the procedure of Benjamini and Hochberg. Biom. J..

[38] Reimand J., Kull M., Peterson H., Hansen J., Vilo J. (2007). g:Profiler—A web-based toolset for functional profiling of gene lists from large-scale experiments. Nucleic Acids Res..

[39] Thomas P.D. (2017). The Gene Ontology and the Meaning of Biological Function. Methods Mol. Biol..

[40] Fabregat A., Jupe S., Matthews L., Sidiropoulos K., Gillespie M., Garapati P., Haw R., Jassal B., Korninger F., May B. (2018). The Reactome Pathway Knowledgebase. Nucleic Acids Res..

[41] Pastrello C., Kotlyar M., Jurisica I. (2020). Informed Use of Protein-Protein Interaction Data: A Focus on the Integrated Interactions Database (IID). Methods Mol. Biol..

[42] Shannon P., Markiel A., Ozier O., Baliga N.S., Wang J.T., Ramage D., Amin N., Schwikowski B., Ideker T. (2003). Cytoscape: A software environment for integrated models of biomolecular interaction networks. Genome Res..

[43] Luo X., Guo L., Dai X.J., Wang Q., Zhu W., Miao X., Gong H. (2017). Abnormal intrinsic functional hubs in alcohol dependence: Evidence from a voxelwise degree centrality analysis. Neuropsychiatr. Dis. Treat..

[44] Li Y., Li W., Tan Y., Liu F., Cao Y., Lee K.Y. (2017). Hierarchical Decomposition for Betweenness Centrality Measure of Complex Networks. Sci. Rep..

[45] Gilbert M., Li Z., Wu X.N., Rohr L., Gombos S., Harter K., Schulze W.X. (2021). Comparison of path-based centrality measures in protein-protein interaction networks revealed proteins with phenotypic relevance during adaptation to changing nitrogen environments. J. Proteom..

[46] Li G., Li M., Wang J., Li Y., Pan Y. (2020). United Neighborhood Closeness Centrality and Orthology for Predicting Essential Proteins. IEEE/ACM Trans. Comput. Biol. Bioinform..

[47] Zaki N., Efimov D., Berengueres J. (2013). Protein complex detection using interaction reliability assessment and weighted clustering coefficient. BMC Bioinform..

[48] Fan Y., Xia J. (2018). miRNet-Functional Analysis and Visual Exploration of miRNA-Target Interactions in a Network Context. Methods Mol. Biol..

[49] Zhou G., Soufan O., Ewald J., Hancock R.E.W., Basu N., Xia J. (2019). NetworkAnalyst 3.0: A visual analytics platform for comprehensive gene expression profiling and meta-analysis. Nucleic Acids Res..

[50] Robin X., Turck N., Hainard A., Tiberti N., Lisacek F., Sanchez J.C., Müller M. (2011). pROC: An open-source package for R and S+ to analyze and compare ROC curves. BMC Bioinform..

[51] Thannickal V.J., Flaherty K.R., Martinez F.J., Lynch J.P. (2004). Idiopathic pulmonary fibrosis: Emerging concepts on pharmacotherapy. Expert Opin. Pharmacother..

[52] Wang J., Yin J., Li W., Xiao C., Han J., Zhou F. (2020). Association between SLCO1A2 genetic variation and methotrexate toxicity in human rheumatoid arthritis treatment. J. Biochem. Mol. Toxicol..

[53] Ren X., Geng M., Xu K., Lu C., Cheng Y., Kong L., Cai Y., Hou W., Lu Y., Aihaiti Y. (2021). Quantitative Proteomic Analysis of Synovial Tissue Reveals That Upregulated OLFM4 Aggravates Inflammation in Rheumatoid Arthritis. J. Proteome Res..

[54] Myouzen K., Kochi Y., Okada Y., Terao C., Suzuki A., Ikari K., Tsunoda T., Takahashi A., Kubo M., Taniguchi A. (2012). Functional variants in NFKBIE and RTKN2 involved in activation of the NF-κB pathway are associated with rheumatoid arthritis in Japanese. PLoS Genet..

[55] Yen J.H., Chen C.J., Tsai W.C., Lin C.H., Ou T.T., Hu C.J., Liu H.W. (2003). Manganese superoxide dismutase and cytochrome P450 1A1 genes polymorphisms in rheumatoid arthritis in Taiwan. Hum. Immunol..

[56] Volin M.V., Shahrara S., Haines G.K., Woods J.M., Koch A.E. (2008). Expression of mucin 3 and mucin 5AC in arthritic synovial tissue. Arthritis Rheum..

[57] Gao X.Z., Wang G.N., Zhao W.G., Han J., Diao C.Y., Wang X.H., Li S.L., Li W.C. (2019). Blocking OLFM4/HIF-1α axis alleviates hypoxia-induced invasion, epithelial-mesenchymal transition, and chemotherapy resistance in non-small-cell lung cancer. J. Cell. Physiol..

[58] Chen K., Ye C., Gao Z., Hu J., Chen C., Xiao R., Lu F., Wei K. (2023). Immune infiltration patterns and identification of new diagnostic biomarkers GDF10, NCKAP5, and RTKN2 in non-small cell lung cancer. Transl. Oncol..

[59] Shi X., Zhou S., Wang Z., Zhou Z., Wang Z. (2008). CYP1A1 and GSTM1 polymorphisms and lung cancer risk in Chinese populations: A meta-analysis. Lung Cancer.

[60] Lu L., Zeng Y., Yu Z., Chen S., Xie J., Rao B., Yang B., Qiu F., Lu J., Yang L. (2023). EIF4a3-regulated circRABL2B regulates cell stemness and drug sensitivity of lung cancer via YBX1-dependent downregulation of MUC5AC expression. Int. J. Biol. Sci..

[61] Park S.L., Murphy S.E., Wilkens L.R., Stram D.O., Hecht S.S., Le Marchand L. (2017). Association of CYP2A6 activity with lung cancer incidence in smokers: The multiethnic cohort study. PLoS ONE.

[62] Shao F., Bian X., Wang J., Xu D., Guo W., Jiang H., Zhao G., Zhu L., Wang S., Xing D. (2021). Prognostic Impact of PCK1 Protein Kinase Activity-Dependent Nuclear SREBP1 Activation in Non-Small-Cell Lung Carcinoma. Front. Oncol..

[63] Li W., Yang P., Zhong C., Shen X., Shi X., Li X. (2022). The circ-PITX1 promotes non-small cell lung cancer development via the miR-30e-5p/ITGA6 axis. Cell Cycle.

[64] Albuquerque D., Nóbrega C., Rodríguez-López R., Manco L. (2014). Association study of common polymorphisms in MSRA, TFAP2B, MC4R, NRXN3, PPARGC1A, TMEM18, SEC16B, HOXB5 and OLFM4 genes with obesity-related traits among Portuguese children. J. Hum. Genet..

[65] Brand H.K., Ahout I.M., de Ridder D., van Diepen A., Li Y., Zaalberg M., Andeweg A., Roeleveld N., de Groot R., Warris A. (2015). Olfactomedin 4 Serves as a Marker for Disease Severity in Pediatric Respiratory Syncytial Virus (RSV) Infection. PLoS ONE.

[66] Cui C., Huang C., Zhou W., Ji X., Zhang F., Wang L., Zhou Y., Cui Q. (2021). AGTR2, One Possible Novel Key Gene for the Entry of SARS-CoV-2 Into Human Cells. IEEE/ACM Trans. Comput. Biol. Bioinform..

[67] Von Schmiedeberg S., Fritsche E., Rönnau A.C., Specker C., Golka K., Richter-Hintz D., Schuppe H.C., Lehmann P., Ruzicka T., Esser C. (1999). Polymorphisms of the xenobiotic-metabolizing enzymes CYP1A1 and NAT-2 in systemic sclerosis and lupus erythematosus. Adv. Exp. Med. Biol..

[68] Parra E.R., Ruppert A.D., Capelozzi V.L. (2014). Angiotensin II type 1 and 2 receptors and lymphatic vessels modulate lung remodeling and fibrosis in systemic sclerosis and idiopathic pulmonary fibrosis. Clinics.

[69] Zhao J., Zhang W., Shen L., Yang X., Liu Y., Gai Z. (2017). Association of the ACE, GSTM1, IL-6, NOS3, and CYP1A1 polymorphisms with susceptibility of mycoplasma pneumoniae pneumonia in Chinese children. Medicine.

[70] Conti C., Montero-Fernandez A., Borg E., Osadolor T., Viola P., De Lauretis A., Stock C.J., Bonifazi M., Bonini M., Caramori G. (2016). Mucins MUC5B and MUC5AC in Distal Airways and Honeycomb Spaces: Comparison among Idiopathic Pulmonary Fibrosis/Usual Interstitial Pneumonia, Fibrotic Nonspecific Interstitial Pneumonitis, and Control Lungs. Am. J. Respir. Crit. Care Med..

[71] Zou J.G., Ma Y.T., Xie X., Yang Y.N., Pan S., Adi D., Liu F., Chen B.D. (2014). The association between CYP1A1 genetic polymorphisms and coronary artery disease in the Uygur and Han of China. Lipids Health Dis..

[72] Yap R.W.K., Shidoji Y., Yap W.S., Masaki M. (2017). Association and Interaction Effect of AGTR1 and AGTR2 Gene Polymorphisms with Dietary Pattern on Metabolic Risk Factors of Cardiovascular Disease in Malaysian Adults. Nutrients.

[73] Wang C.D., Chen N., Huang L., Wang J.R., Chen Z.Y., Jiang Y.M., He Y.Z., Ji Y.L. (2015). Impact of CYP1A1 Polymorphisms on Susceptibility to Chronic Obstructive Pulmonary Disease: A Meta-Analysis. Biomed. Res. Int..

[74] Wan E.S., Li Y., Lao T., Qiu W., Jiang Z., Mancini J.D., Owen C.A., Clish C., DeMeo D.L., Silverman E.K. (2017). Metabolomic profiling in a Hedgehog Interacting Protein (Hhip) murine model of chronic obstructive pulmonary disease. Sci. Rep..

[75] Liu W., Li J., Li T., Xie Y., Luo C. (2022). Reineckia carnea Alleviates the Production of Inflammatory Cytokines and MUC5AC in Rats with Chronic Obstructive Pulmonary Disease. Evid. Based Complement. Alternat. Med..

[76] Pezzuto A., Lionetto L., Ricci A., Simmaco M., Borro M. (2021). Inter-individual variation in CYP2A6 activity and chronic obstructive pulmonary disease in smokers: Perspectives for an early predictive marker. Biochim. Biophys. Acta Mol. Basis Dis..

[77] Cho S.H., Park S.Y., Lee E.J., Cho Y.H., Park H.S., Hong S.H., Kim W.J. (2015). Regulation of CYP1A1 and Inflammatory Cytokine by NCOA7 Isoform 4 in Response to Dioxin Induced Airway Inflammation. Tuberc. Respir. Dis..

[78] Wang X., Li Y., Luo D., Wang X., Zhang Y., Liu Z., Zhong N., Wu M., Li G. (2017). Lyn regulates mucus secretion and MUC5AC via the STAT6 signaling pathway during allergic airway inflammation. Sci. Rep..

[79] Lennox A.T., Coburn S.L., Leech J.A., Heidrich E.M., Kleyman T.R., Wenzel S.E., Pilewski J.M., Corcoran T.E., Myerburg M.M. (2018). ATP12A promotes mucus dysfunction during Type 2 airway inflammation. Sci. Rep..

[80] Wang H., Wang Y., Zhang H., Liang Z., Hu W., Qiu S., Li K., Zhang L., Dai H., Yang M. (2023). Hedgehog interacting protein as a circulating biomarker in women with obesity: A cross-sectional study and intervention studies. Ann. Med..

[81] Li T., Wang Y., Huang S., Tang H. (2022). The Regulation Mechanism of MUC5AC Secretion in Airway of Obese Asthma. Cell. Mol. Biol..

[82] Liu T., David S.P., Tyndale R.F., Wang H., Yu X.Q., Chen W., Zhou Q., Chen W.Q. (2012). Relationship between amounts of daily cigarette consumption and abdominal obesity moderated by CYP2A6 genotypes in Chinese male current smokers. Ann. Behav. Med..

[83] Beale E.G., Harvey B.J., Forest C. (2007). PCK1 and PCK2 as candidate diabetes and obesity genes. Cell Biochem. Biophys..

[84] Abdelgied M., Uhl K., Chen O.G., Schultz C., Tripp K., Peraino A.M., Paithankar S., Chen B., Tamae Kakazu M., Castillo Bahena A. (2023). Targeting ATP12A, a Nongastric Proton Pump α Subunit, for Idiopathic Pulmonary Fibrosis Treatment. Am. J. Respir. Cell Mol. Biol..

[85] Wang X.L., Greco M., Sim A.S., Duarte N., Wang J., Wilcken D.E. (2002). Effect of CYP1A1 MspI polymorphism on cigarette smoking related coronary artery disease and diabetes. Atherosclerosis.

[86] Lin A.C., Hung H.C., Chen Y.W., Cheng K.P., Li C.H., Lin C.H., Chang C.J., Wu H.T., Ou H.Y. (2019). Elevated Hedgehog-Interacting Protein Levels in Subjects with Prediabetes and Type 2 Diabetes. J. Clin. Med..

[87] Robillard S., Mercier C., Breton V., Paquin-Veillette J., Guay A., Lizotte F., Geraldes P. (2020). Ablation of angiotensin type 2 receptor prevents endothelial nitric oxide synthase glutathionylation and nitration in ischaemic abductor muscle of diabetic mice. Diabetes Vasc. Dis. Res..

[88] Liu T., Chen W.Q., David S.P., Tyndale R.F., Wang H., Chen Y.M., Yu X.Q., Chen W., Zhou Q., Ling W.H. (2011). Interaction between heavy smoking and CYP2A6 genotypes on type 2 diabetes and its possible pathways. Eur. J. Endocrinol..

[89] Haak A.J., Ducharme M.T., Diaz Espinosa A.M., Tschumperlin D.J. (2020). Targeting GPCR Signaling for Idiopathic Pulmonary Fibrosis Therapies. Trends Pharmacol. Sci..

[90] Borzì R.M., Grigolo B., Meliconi R., Fasano L., Sturani C., Fabbri M., Porstmann T., Facchini A. (1993). Elevated serum superoxide dismutase levels correlate with disease severity and neutrophil degranulation in idiopathic pulmonary fibrosis. Clin. Sci..

[91] Miles T., Hoyne G.F., Knight D.A., Fear M.W., Mutsaers S.E., Prêle C.M. (2020). The contribution of animal models to understanding the role of the immune system in human idiopathic pulmonary fibrosis. Clin. Transl. Immunol..

[92] Chen R.l., Dai J. (2023). Lipid metabolism in idiopathic pulmonary fibrosis: From pathogenesis to therapy. J. Mol. Med..

[93] Pechkovsky D.V., Prasse A., Kollert F., Engel K.M., Dentler J., Luttmann W., Friedrich K., Müller-Quernheim J., Zissel G. (2010). Alternatively activated alveolar macrophages in pulmonary fibrosis-mediator production and intracellular signal transduction. Clin. Immunol..

[94] Nag A., Dhindsa R.S., Mitchell J., Vasavda C., Harper A.R., Vitsios D., Ahnmark A., Bilican B., Madeyski-Bengtson K., Zarrouki B. (2022). Human genetics uncovers MAP3K15 as an obesity-independent therapeutic target for diabetes. Sci. Adv..

[95] Mirea A.M., Stienstra R., Kanneganti T.D., Tack C.J., Chavakis T., Toonen E.J.M., Joosten L.A.B. (2020). Mice Deficient in the IL-1β Activation Genes Prtn3, Elane, and Casp1 Are Protected Against the Development of Obesity-Induced NAFLD. Inflammation.

[96] Polyák A., Ferenczi S., Dénes A., Winkler Z., Kriszt R., Pintér-Kübler B., Kovács K.J. (2014). The fractalkine/Cx3CR1 system is implicated in the development of metabolic visceral adipose tissue inflammation in obesity. Brain Behav. Immun..

[97] Bäckberg M., Madjid N., Ogren S.O., Meister B. (2004). Down-regulated expression of agouti-related protein (AGRP) mRNA in the hypothalamic arcuate nucleus of hyperphagic and obese tub/tub mice. Mol. Brain Res..

[98] Qaddoumi M.G., Alanbaei M., Hammad M.M., Al Khairi I., Cherian P., Channanath A., Thanaraj T.A., Al-Mulla F., Abu-Farha M., Abubaker J. (2020). Investigating the Role of Myeloperoxidase and Angiopoietin-like Protein 6 in Obesity and Diabetes. Sci. Rep..

[99] Shoji S., Uchida K., Inoue G., Takata K., Mukai M., Aikawa J., Iwase D., Takano S., Sekiguchi H., Takaso M. (2021). Increase in CD5L expression in the synovial membrane of knee osteoarthritis patients with obesity. Cent. Eur. J. Immunol..

[100] Miyashita D., Inoue R., Tsuno T., Okuyama T., Kyohara M., Nakahashi-Oda C., Nishiyama K., Fukushima S., Inada Y., Togashi Y. (2022). Protective effects of S100A8 on sepsis mortality: Links to sepsis risk in obesity and diabetes. iScience.

[101] Guarino B.D., Dado C.D., Kumar A., Braza J., Harrington E.O., Klinger J.R. (2023). Deletion of the Npr3 gene increases severity of acute lung injury in obese mice. Pulm. Circ..

[102] Chakraborty A., Barajas S., Lammoglia G.M., Reyna A.J., Morley T.S., Johnson J.A., Scherer P.E., Rutkowski J.M. (2019). Vascular Endothelial Growth Factor-D (VEGF-D) Overexpression and Lymphatic Expansion in Murine Adipose Tissue Improves Metabolism in Obesity. Am. J. Pathol..

[103] Kochumon S., Madhoun A.A., Al-Rashed F., Azim R., Al-Ozairi E., Al-Mulla F., Ahmad R. (2020). Adipose tissue gene expression of CXCL10 and CXCL11 modulates inflammatory markers in obesity: Implications for metabolic inflammation and insulin resistance. Ther. Adv. Endocrinol. Metab..

[104] Maculewicz E., Antkowiak B., Antkowiak O., Borecka A., Mastalerz A., Leońska-Duniec A., Humińska-Lisowska K., Michałowska-Sawczyn M., Garbacz A., Lorenz K. (2022). The interactions between interleukin-1 family genes: IL1A, IL1B, IL1RN, and obesity parameters. BMC Genom..

[105] Zheng F., Han J., Lu H., Cui C., Yang J., Cui Q., Cai J., Zhou Y., Tang C., Xu G. (2018). Cystathionine beta synthase-hydrogen sulfide system in paraventricular nucleus reduced high fatty diet induced obesity and insulin resistance by brain-adipose axis. Biochim. Biophys. Acta Mol. Basis Dis..

[106] Wang W., Yan X., Lin Y., Ge H., Tan Q. (2018). Wnt7a promotes wound healing by regulation of angiogenesis and inflammation: Issues on diabetes and obesity. J. Dermatol. Sci..

[107] Poudyal H., Brown L. (2011). Stearoyl-CoA desaturase: A vital checkpoint in the development and progression of obesity. Endocr. Metab. Immune Disord. Drug Targets.

[108] Sandin E.S., Folberth J., Müller-Fielitz H., Pietrzik C.U., Herold E., Willnow T.E., Pfluger P.T., Nogueiras R., Prevot V., Krey T. (2021). LRP2 Involved in Leptin Transport over the Blood-Brain Barrier and Development of Obesity?. Int. J. Mol. Sci..

[109] Dias H., Muc M., Padez C., Manco L. (2016). Association of polymorphisms in 5-HTT (SLC6A4) and MAOA genes with measures of obesity in young adults of Portuguese origin. Arch. Physiol. Biochem..

[110] Sandrini L., Di Minno A., Amadio P., Ieraci A., Tremoli E., Barbieri S.S. (2018). Association between Obesity and Circulating Brain-Derived Neurotrophic Factor (BDNF) Levels: Systematic Review of Literature and Meta-Analysis. Int. J. Mol. Sci..

[111] Moreno B., Hueso L., Ortega R., Benito E., Martínez-Hervas S., Peiro M., Civera M., Sanz M.J., Piqueras L., Real J.T. (2022). Association of chemokines IP-10/CXCL10 and I-TAC/CXCL11 with insulin resistance and enhance leukocyte endothelial arrest in obesity. Microvasc. Res..

[112] Abu-Farha M., Cherian P., Al-Khairi I., Madhu D., Tiss A., Warsam S., Alhubail A., Sriraman D., Al-Refaei F., Abubaker J. (2017). Plasma and adipose tissue level of angiopoietin-like 7 (ANGPTL7) are increased in obesity and reduced after physical exercise. PLoS ONE.

[113] Pan X., Yang L., Wang S., Liu Y., Yue L., Chen S. (2023). Semaglutide ameliorates obesity-induced cardiac inflammation and oxidative stress mediated via reduction of neutrophil Cxcl2, S100a8, and S100a9 expression. Mol. Cell. Biochem..

[114] Feng X., Ding Y., Zhou M., Song N., Ding Y. (2022). Integrative Analysis of Exosomal miR-452 and miR-4713 Downregulating NPY1R for the Prevention of Childhood Obesity. Dis. Markers.

[115] de Moraes Rodrigues J., Souza de Lima D., Leal V.N.C., Bosco A.A., Sandrim V., Pontillo A. (2018). Gain-of-function SNPs in NLRP3 and IL1B genes confer protection against obesity and T2D: Undiscovered role of inflammasome genetics in metabolic ho-meostasis?. Endocrine.

[116] Aruga M., Tokita Y., Nakajima K., Kamachi K., Tanaka A. (2017). The effect of combined diet and exercise intervention on body weight and the serum GPIHBP1 concentration in overweight/obese middle-aged women. Clin. Chim. Acta.

[117] Rojas I.Y., Moyer B.J., Ringelberg C.S., Tomlinson C.R. (2020). Reversal of obesity and liver steatosis in mice via inhibition of aryl hydrocarbon receptor and altered gene expression of CYP1B1, PPARα, SCD1, and osteopontin. Int. J. Obes..

[118] Luo X., Li Y., Yang P., Chen Y., Wei L., Yu T., Xia J., Ruan X.Z., Zhao L., Chen Y. (2020). Obesity induces preadipocyte CD36 expression promoting inflammation via the disruption of lysosomal calcium homeostasis and lysosome function. eBioMedicine.

[119] Salas-Perez F., Assmann T.S., Ramos-Lopez O., Martínez J.A., Riezu-Boj J.I., Milagro F.I. (2023). Crosstalk between Gut Microbiota and Epigenetic Markers in Obesity Development: Relationship between Ruminococcus, BMI, and MACROD2/SEL1L2 Methylation. Nutrients.

[120] Lee S.K., Park C.Y., Kim J., Kim D., Choe H., Kim J.H., Hong J.P., Lee Y.J., Heo Y., Park H.S. (2022). TRIB3 Is Highly Expressed in the Adipose Tissue of Obese Patients and Is Associated with Insulin Resistance. J. Clin. Endocrinol. Metab..

[121] Kumar S., Mankowski R.T., Anton S.D., Babu Balagopal P. (2021). Novel insights on the role of spexin as a biomarker of obesity and related cardiometabolic disease. Int. J. Obes..

[122] Macchi C., Greco M.F., Favero C., Dioni L., Cantone L., Hoxha M., Vigna L., Solazzo G., Corsini A., Banach M. (2022). Associations Among PCSK9 Levels, Atherosclerosis-Derived Extracellular Vesicles, and Their miRNA Content in Adults with Obesity. Front. Cardiovasc. Med..

[123] Li N., Chang G., Xu Y., Ding Y., Li G., Yu T., Yao R., Li J., Shen Y., Wang X. (2017). Biallelic mutations in GPD1 gene in a Chinese boy mainly presented with obesity, insulin resistance, fatty liver, and short stature. Am. J. Med. Genet. A.

[124] Jo J., Sull J.W., Park E.J., Jee S.H. (2012). Effects of smoking and obesity on the association between CDH13 (rs3865188) and adiponectin among Korean men: The KARE study. Obesity.

[125] Peiris M., Aktar R., Reed D., Cibert-Goton V., Zdanaviciene A., Halder W., Robinow A., Corke S., Dogra H., Knowles C.H. (2022). Decoy bypass for appetite suppression in obese adults: Role of synergistic nutrient sensing receptors GPR84 and FFAR4 on colonic endocrine cells. Gut.

[126] Hao R.H., Guo Y., Dong S.S., Weng G.Z., Yan H., Zhu D.L., Chen X.F., Chen J.B., Yang T.L. (2016). Associations of Plasma FGF2 Levels and Polymorphisms in the FGF2 Gene with Obesity Phenotypes in Han Chinese Population. Sci. Rep..

[127] Sun D., Zhao T., Zhang Q., Wu M., Zhang Z. (2021). Fat mass and obesity-associated protein regulates lipogenesis via m6 A modification in fatty acid synthase mRNA. Cell Biol. Int..

[128] Yenilmez B., Wetoska N., Kelly M., Echeverria D., Min K., Lifshitz L., Alterman J.F., Hassler M.R., Hildebrand S., DiMarzio C. (2022). An RNAi therapeutic targeting hepatic DGAT2 in a genetically obese mouse model of nonalcoholic steatohepatitis. Mol. Ther..

[129] Ozcan L., Ghorpade D.S., Zheng Z., Cristina de Souza J., Chen K., Bessler M., Bagloo M., Schrope B., Pestell R., Tabas I. (2022). Hepatocyte DACH1 Is Increased in Obesity via Nuclear Exclusion of HDAC4 and Promotes Hepatic Insulin Resistance. Cell Rep..

[130] Marzuillo P., Di Sessa A., Guarino S., Capalbo D., Umano G.R., Pedullà M., La Manna A., Cirillo G., Miraglia Del Giudice E. (2019). Nonalcoholic fatty liver disease and eGFR levels could be linked by the PNPLA3 I148M polymorphism in children with obesity. Pediatr. Obes..

[131] Sun Y., Wang R., Zhao S., Li W., Liu W., Tang L., Wang Z., Wang W., Liu R., Ning G. (2019). FGF9 inhibits browning program of white adipocytes and associates with human obesity. J. Mol. Endocrinol..

[132] Li F., Hao S., Gao J., Jiang P. (2023). EGCG alleviates obesity-exacerbated lung cancer progression by STAT1/SLC7A11 pathway and gut microbiota. J. Nutr. Biochem..

[133] Bradford E.M., Miller M.L., Prasad V., Nieman M.L., Gawenis L.R., Berryman M., Lorenz J.N., Tso P., Shull G.E. (2010). CLIC5 mutant mice are resistant to diet-induced obesity and exhibit gastric hemorrhaging and increased susceptibility to torpor. Am. J. Physiol. Regul. Integr. Comp. Physiol..

[134] Atas U., Erin N., Tazegul G., Elpek G.O., Yildirim B. (2021). Changes in ghrelin, substance P and vasoactive intestinal peptide levels in the gastroduodenal mucosa of patients with morbid obesity. Neuropeptides.

[135] Niu H.M., Liu C.L. (2017). The aberrant expression of Smad6 and TGF-β in obesity linked cardiac disease. Eur. Rev. Med. Pharmacol. Sci..

[136] Schleinitz D., Klöting N., Böttcher Y., Wolf S., Dietrich K., Tönjes A., Breitfeld J., Enigk B., Halbritter J., Körner A. (2011). Genetic and evolutionary analyses of the human bone morphogenetic protein receptor 2 (BMPR2) in the pathophysiology of obesity. PLoS ONE.

[137] Lu M., Lu Q., Zhang Y., Tian G. (2011). ApoB/apoA1 is an effective predictor of coronary heart disease risk in overweight and obesity. J. Biomed. Res..

[138] Carobbio S., Hagen R.M., Lelliott C.J., Slawik M., Medina-Gomez G., Tan C.Y., Sicard A., Atherton H.J., Barbarroja N., Bjursell M. (2013). Adaptive changes of the Insig1/SREBP1/SCD1 set point help adipose tissue to cope with increased storage demands of obesity. Diabetes.

[139] Zhang G., Li R., Li W., Yang S., Sun Q., Yin H., Wang C., Hou B., Wang H., Yu L. (2021). Toll-like receptor 3 ablation prevented high-fat diet-induced obesity and metabolic disorder. J. Nutr. Biochem..

[140] Truax A.D., Chen L., Tam J.W., Cheng N., Guo H., Koblansky A.A., Chou W.C., Wilson J.E., Brickey W.J., Petrucelli A. (2018). The Inhibitory Innate Immune Sensor NLRP12 Maintains a Threshold against Obesity by Regulating Gut Microbiota Homeostasis. Cell Host Microbe.

[141] Aradillas-Garc C., Cruz M., Pérez-Luque E., Garay-Sevilla M.E., Malacara J.M., Aduna R., Peralta J., Burguete-García A., Alegría-Torres J.A. (2016). Obesity is associated with the Arg389Gly ADRB1 but not with the Trp64Arg ADRB3 polymorphism in children from San Luis PotosÍ and León, México. J. Biomed. Res..

[142] Ahmad R., Kochumon S., Thomas R., Atizado V., Sindhu S. (2016). Increased adipose tissue expression of TLR8 in obese individuals with or without type-2 diabetes: Significance in metabolic inflammation. J. Inflamm..

[143] El-Arabey A.A., Abdalla M. (2022). GATA3 as an immunomodulator in obesity-related metabolic dysfunction associated with fatty liver disease, insulin resistance, and type 2 diabetes. Chem. Biol. Interact..

[144] Lee S.J., Kang J.S., Kim H.M., Lee E.S., Lee J.H., Chung C.H., Lee E.Y. (2019). CCR2 knockout ameliorates obesity-induced kidney injury through inhibiting oxidative stress and ER stress. PLoS ONE.

[145] Englmeier L. (2020). A theory on SARS-COV-2 susceptibility: Reduced TLR7-activity as a mechanistic link between men, obese and elderly. J. Biol. Regul. Homeost. Agents.

[146] Xu M., Wang Y.M., Li W.Q., Huang C.L., Li J., Xie W.H., Zeng H.X., Tao L.F., Li X. (2020). Ccrl2 deficiency deteriorates obesity and insulin resistance through increasing adipose tissue macrophages infiltration. Genes Dis..

[147] Pérez L.M., de Lucas B., Gálvez B.G. (2021). BMPER is upregulated in obesity and seems to have a role in pericardial adipose stem cells. J. Cell. Physiol..

[148] Al Madhoun A., Kochumon S., Haddad D., Thomas R., Nizam R., Miranda L., Sindhu S., Bitar M.S., Ahmad R., Al-Mulla F. (2023). Adipose Tissue Caveolin-1 Upregulation in Obesity Involves TNF-α/NF-κB Mediated Signaling. Cells.

[149] Vambergue A., Rugeri L., Gaveriaux V., Devos P., Martin A., Fermon C., Fontaine P., Jude B. (2001). Factor VII, tissue factor pathway inhibitor, and monocyte tissue factor in diabetes mellitus: Influence of type of diabetes, obesity index, and age. Thromb. Res..

[150] Wang C., Murphy J., Delaney K.Z., Khor N., Morais J.A., Tsoukas M.A., Lowry D.E., Mutch D.M., Santosa S. (2021). Association between rs174537 FADS1 polymorphism and immune cell profiles in abdominal and femoral subcutaneous adipose tissue: An exploratory study in adults with obesity. Adipocyte.

[151] Keiran N., Ceperuelo-Mallafré V., Calvo E., Hernández-Alvarez M.I., Ejarque M., Núñez-Roa C., Horrillo D., Maymó-Masip E., Rodríguez M.M., Fradera R. (2019). SUCNR1 controls an anti-inflammatory program in macrophages to regulate the metabolic response to obesity. Nat. Immunol..

[152] Morris J., Bailey M.E.S., Baldassarre D., Cullen B., de Faire U., Ferguson A., Gigante B., Giral P., Goel A., Graham N. (2019). Genetic variation in CADM2 as a link between psychological traits and obesity. Sci. Rep..

[153] Pereira M.J., Andersson-Assarsson J.C., Jacobson P., Kamble P., Taube M., Sjöholm K., Carlsson L.M.S., Svensson P.A. (2021). Human adipose tissue gene expression of solute carrier family 19 member 3 (SLC19A3); relation to obesity and weight-loss. Obes. Sci. Pract..

[154] Kuhn T., Kaiser K., Lebek S., Altenhofen D., Knebel B., Herwig R., Rasche A., Pelligra A., Görigk S., Khuong J.M. (2022). Comparative genomic analyses of multiple backcross mouse populations suggest SGCG as a novel potential obesity-modifier gene. Hum. Mol. Genet..

[155] Morales L.D., Cromack D.T., Tripathy D., Fourcaudot M., Kumar S., Curran J.E., Carless M., Göring H.H.H., Hu S.L., Lopez-Alvarenga J.C. (2021). Further evidence supporting a potential role for ADH1B in obesity. Sci. Rep..

[156] Ng M.C., Tam C.H., So W.Y., Ho J.S., Chan A.W., Lee H.M., Wang Y., Lam V.K., Chan J.C., Ma R.C. (2010). Implication of genetic variants near NEGR1, SEC16B, TMEM18, ETV5/DGKG, GNPDA2, LIN7C/BDNF, MTCH2, BCDIN3D/FAIM2, SH2B1, FTO, MC4R, and KCTD15 with obesity and type 2 diabetes in 7705 Chinese. J. Clin. Endocrinol. Metab..

[157] Hachim M.Y., Aljaibeji H., Hamoudi R.A., Hachim I.Y., Elemam N.M., Mohammed A.K., Salehi A., Taneera J., Sulaiman N. (2020). An Integrative Phenotype-Genotype Approach Using Phenotypic Characteristics from the UAE National Diabetes Study Identifies HSD17B12 as a Candidate Gene for Obesity and Type 2 Diabetes. Genes.

[158] Çatli G., Acar S., Cingöz G., Rasulova K., Yarim A.K., Uzun H., Küme T., Kızıldağ S., Dündar B.N., Abacı A. (2021). Oxytocin receptor gene polymorphism and low serum oxytocin level are associated with hyperphagia and obesity in adolescents. Int. J. Obes..

[159] Aliasghari F., Nazm S.A., Yasari S., Mahdavi R., Bonyadi M. (2021). Associations of the ANKK1 and DRD2 gene polymorphisms with overweight, obesity and hedonic hunger among women from the Northwest of Iran. Eat. Weight Disord..

[160] Njerve I.U., Pettersen A.Å., Opstad T.B., Arnesen H., Seljeflot I. (2012). Fractalkine and its receptor (CX3CR1) in patients with stable coronary artery disease and diabetes mellitus. Metab. Syndr. Relat. Disord..

[161] Gellen B., Thorin-Trescases N., Thorin E., Gand E., Ragot S., Montaigne D., Pucheu Y., Mohammedi K., Gatault P., Potier L. (2022). Increased serum S100A12 levels are associated with higher risk of acute heart failure in patients with type 2 diabetes. ESC Heart Fail..

[162] Bayraktar M., Dündar S., Kirazli S., Teletar F. (1994). Platelet factor 4, beta-thromboglobulin and thrombospondin levels in type I diabetes mellitus patients. J. Int. Med. Res..

[163] Traisaeng S., Batsukh A., Chuang T.H., Herr D.R., Huang Y.F., Chimeddorj B., Huang C.M. (2020). Leuconostoc mesenteroides fermentation produces butyric acid and mediates Ffar2 to regulate blood glucose and insulin in type 1 diabetic mice. Sci. Rep..

[164] Shukla S.K., Liu W., Sikder K., Addya S., Sarkar A., Wei Y., Rafiq K. (2017). HMGCS2 is a key ketogenic enzyme potentially involved in type 1 diabetes with high cardiovascular risk. Sci. Rep..

[165] Paszek E., Polak M., Bryk-Wiązania A.H., Konieczyńska M., Undas A. (2023). Elevated plasma factor XI predicts cardiovascular events in patients with type 2 diabetes: A long-term observational study. Cardiovasc. Diabetol..

[166] Kochetova O.V., Avzaletdinova D.S., Korytina G.F., Morugova T.V., Mustafina O.E. (2020). The association between eating behavior and polymorphisms in GRIN2B, GRIK3, GRIA1 and GRIN1 genes in people with type 2 diabetes mellitus. Mol. Biol. Rep..

[167] Saulnier P.J., Roussel R., Halimi J.M., Lebrec J., Dardari D., Maimaitiming S., Guilloteau G., Prugnard X., Marechaud R., Ragot S. (2011). Impact of natriuretic peptide clearance receptor (NPR3) gene variants on blood pressure in type 2 diabetes. Diabetes Care.

[168] Li X., Zhu J., Zhong Y., Liu C., Yao M., Sun Y., Yao W., Ni X., Zhou F., Yao J. (2022). Targeting long noncoding RNA-AQP4-AS1 for the treatment of retinal neurovascular dysfunction in diabetes mellitus. eBioMedicine.

[169] Oballa R.M., Belair L., Black W.C., Bleasby K., Chan C.C., Desroches C., Du X., Gordon R., Guay J., Guiral S. (2011). Development of a liver-targeted stearoyl-CoA desaturase (SCD) inhibitor (MK-8245) to establish a therapeutic window for the treatment of diabetes and dyslipidemia. J. Med. Chem..

[170] Xiu L., Lin M., Liu W., Kong D., Liu Z., Zhang Y., Ouyang P., Liang Y., Zhong S., Chen C. (2015). Association of DRD3, COMT, and SLC6A4 Gene Polymorphisms with Type 2 Diabetes in Southern Chinese: A Hospital-Based Case-Control Study. Diabetes Technol. Ther..

[171] Gao X., Zhao S. (2020). miRNA-16-5p inhibits the apoptosis of high glucose-induced pancreatic β cells via targeting of CXCL10: Potential biomarkers in type 1 diabetes mellitus. Endokrynol. Pol..

[172] Cai Q., Zhu J., Cui X., Xia Y., Gao H., Wang X., Cheng M. (2022). S100A9 promotes inflammatory response in diabetic nonalcoholic fatty liver disease. Biochem. Biophys. Res. Commun..

[173] Choong Y.S., Lim Y.Y., Soong J.X., Savoo N., Guida C., Rhyman L., Ramracheya R., Ramasami P. (2021). Theoretical study of the interactions between peptide tyrosine tyrosine [PYY (1-36)], a newly identified modulator in type 2 diabetes pathophysiology, with receptors NPY1R and NPY4R. Hormones.

[174] Li J., Sun X., Luo S., Lin J., Xiao Y., Yu H., Huang G., Li X., Xie Z., Zhou Z. (2021). The Positivity Rate of IA-2A and ZnT8A in the Chinese Han Population with Type 1 Diabetes Mellitus: Association With rs1143627 and rs1143643 Polymorphisms in the IL1B Gene. Front. Pharmacol..

[175] Maffi P., Lundgren T., Tufveson G., Rafael E., Shaw J.A.M., Liew A., Saudek F., Witkowski P., Golab K., Bertuzzi F. (2020). Targeting CXCR1/2 Does Not Improve Insulin Secretion After Pancreatic Islet Transplantation: A Phase 3, Double-Blind, Randomized, Placebo-Controlled Trial in Type 1 Diabetes. Diabetes Care.

[176] Zhong X., Zhao X., Zhang L., Liu N., Shi S., Wang Y. (2022). Sodium hydrosulfide inhibiting endothelial cells injury and neutrophils activation via IL-8/CXCR2/ROS/NF-κB axis in type 1 diabetes mellitus rat. Biochem. Biophys. Res. Commun..

[177] Kurooka N., Eguchi J., Murakami K., Kamei S., Kikutsuji T., Sasaki S., Seki A., Yamaguchi S., Nojima I., Watanabe M. (2022). Circulating GPIHBP1 levels and microvascular complications in patients with type 2 diabetes: A cross-sectional study. J. Clin. Lipidol..

[178] Li J., Cai J., Liu L., Wu Y., Chen Y. (2022). Pulsed electromagnetic fields inhibit mandibular bone deterioration depending on the Wnt3a/β-catenin signaling activation in type 2 diabetic db/db mice. Sci. Rep..

[179] Castro A., Lázaro I., Selva D.M., Céspedes E., Girona J., NúriaPlana, Guardiola M., Cabré A., Simó R., Masana L. (2010). APOH is increased in the plasma and liver of type 2 diabetic patients with metabolic syndrome. Atherosclerosis.

[180] Guo Y., Traurig M., Ma L., Kobes S., Harper I., Infante A.M., Bogardus C., Baier L.J., Prochazka M. (2006). CHRM3 gene variation is associated with decreased acute insulin secretion and increased risk for early-onset type 2 diabetes in Pima Indians. Diabetes.

[181] Puchałowicz K., Rać M.E. (2020). The Multifunctionality of CD36 in Diabetes Mellitus and Its Complications-Update in Pathogenesis, Treatment and Monitoring. Cells.

[182] Lu G., Li J., Gao T., Liu Q., Chen O., Zhang X., Xiao M., Guo Y., Wang J., Tang Y. (2023). Integration of dietary nutrition and TRIB3 action into diabetes mellitus. Nutr. Rev..

[183] Hsu L.A., Teng M.S., Wu S., Chou H.H., Ko Y.L. (2022). Common and Rare PCSK9 Variants Associated with Low-Density Lipoprotein Cholesterol Levels and the Risk of Diabetes Mellitus: A Mendelian Randomization Study. Int. J. Mol. Sci..

[184] Emdin C.A., Khera A.V., Aragam K., Haas M., Chaffin M., Klarin D., Natarajan P., Bick A., Zekavat S.M., Nomura A. (2019). DNA Sequence Variation in ACVR1C Encoding the Activin Receptor-Like Kinase 7 Influences Body Fat Distribution and Protects Against Type 2 Diabetes. Diabetes.

[185] Carullo G., Mazzotta S., Vega-Holm M., Iglesias-Guerra F., Vega-Pérez J.M., Aiello F., Brizzi A. (2021). GPR120/FFAR4 Pharmacology: Focus on Agonists in Type 2 Diabetes Mellitus Drug Discovery. J. Med. Chem..

[186] Liu D., Liu L., Hu Z., Song Z., Wang Y., Chen Z. (2018). Evaluation of the oxidative stress-related genes ALOX5, ALOX5AP, GPX1, GPX3 and MPO for contribution to the risk of type 2 diabetes mellitus in the Han Chinese population. Diabetes Vasc. Dis. Res..

[187] Yeboah J., Sane D.C., Crouse J.R., Herrington D.M., Bowden D.W. (2007). Low plasma levels of FGF-2 and PDGF-BB are associated with cardiovascular events in type II diabetes mellitus (diabetes heart study). Dis. Markers.

[188] Menendez J.A., Vazquez-Martin A., Ortega F.J., Fernandez-Real J.M. (2009). Fatty acid synthase: Association with insulin resistance, type 2 diabetes, and cancer. Clin. Chem..

[189] Kantartzis K., Machicao F., Machann J., Schick F., Fritsche A., Häring H.U., Stefan N. (2009). The DGAT2 gene is a candidate for the dissociation between fatty liver and insulin resistance in humans. Clin. Sci..

[190] Cao A., Li J., Asadi M., Basgen J.M., Zhu B., Yi Z., Jiang S., Doke T., El Shamy O., Patel N. (2021). DACH1 protects podocytes from experimental diabetic injury and modulates PTIP-H3K4Me3 activity. J. Clin. Investig..

[191] Moon S., Chung G.E., Joo S.K., Park J.H., Chang M.S., Yoon J.W., Koo B.K., Kim W. (2022). A PNPLA3 Polymorphism Confers Lower Susceptibility to Incident Diabetes Mellitus in Subjects with Nonalcoholic Fatty Liver Disease. Clin. Gastroenterol. Hepatol..

[192] Singla D., Wang J. (2016). Fibroblast Growth Factor-9 Activates c-Kit Progenitor Cells and Enhances Angiogenesis in the Infarcted Diabetic Heart. Oxid. Med. Cell. Longev..

[193] Rong J., Li C., Zhang Q., Zheng G., Fan W., Pan Z., Shi S. (2023). Hydroxysafflor yellow A inhibits endothelial cell ferroptosis in diabetic atherosclerosis mice by regulating miR-429/SLC7A11. Pharm. Biol..

[194] Maher E., Bachoo M., Elabbady A.A., Polosa C., Bégin L.R., Collier B., Elhilali M.M., Hassouna M.M. (1996). Vasoactive intestinal peptide and impotence in experimental diabetes mellitus. Br. J. Urol..

[195] Słomiński B., Ryba-Stanisławowska M., Skrzypkowska M., Myśliwska J., Myśliwiec M. (2018). The KL-VS polymorphism of KLOTHO gene is protective against retinopathy incidence in patients with type 1 diabetes. Biochim. Biophys. Acta Mol. Basis Dis..

[196] Wang X., Wang L.T., Yu B. (2022). UBE2D1 and COX7C as Potential Biomarkers of Diabetes-Related Sepsis. Biomed. Res. Int..

[197] Zhu Y., Wang X., Wang W., Wang H., Zhang F. (2018). Expression and influence of pentraxin-3, HbAlc and ApoA1/ApoB in serum of patients with acute myocardial infarction combined with diabetes mellitus type 2. Exp. Ther. Med..

[198] Tsai T.H., Lin C.J., Chua S., Chung S.Y., Chen S.M., Lee C.H., Hang C.L. (2018). Deletion of RasGRF1 Attenuated Interstitial Fibrosis in Streptozotocin-Induced Diabetic Cardiomyopathy in Mice through Affecting Inflammation and Oxidative Stress. Int. J. Mol. Sci..

[199] Huang M.H., Liu Y.F., Nfor O.N., Hsu S.Y., Lin W.Y., Chang Y.S., Liaw Y.P. (2021). Interactive Association Between Intronic Polymorphism (rs10506151) of the LRRK2 Gene and Type 2 Diabetes on Neurodegenerative Diseases. Pharmgenomics Pers. Med..

[200] Zhou Z., Zeng C., Nie L., Huang S., Guo C., Xiao D., Han Y., Ye X., Ou M., Huang C. (2017). The effects of TLR3, TRIF and TRAF3 SNPs and interactions with environmental factors on type 2 diabetes mellitus and vascular complications in a Han Chinese population. Gene.

[201] Yu T., Lu X.J., Li J.Y., Shan T.D., Huang C.Z., Ouyang H., Yang H.S., Xu J.H., Zhong W., Xia Z.S. (2016). Overexpression of miR-429 impairs intestinal barrier function in diabetic mice by down-regulating occludin expression. Cell Tissue Res..

[202] Grbić E., Globočnik Petrovič M., Cilenšek I., Petrovič D. (2023). SLC22A3 rs2048327 Polymorphism Is Associated with Diabetic Retinopathy in Caucasians with Type 2 Diabetes Mellitus. Biomedicines.

[203] Toledo-Corral C.M., Banner L.R. (2012). Early changes of LIFR and gp130 in sciatic nerve and muscle of diabetic mice. Acta Histochem..

[204] Ozgur B.A., Cinar S.A., Coskunpinar E., Yilmaz A., Altunkanat D., Deniz G., Gurol A.O., Yilmaz M.T. (2023). The role of cytokines and T-bet, GATA3, ROR-γt, and FOXP3 transcription factors of T cell subsets in the natural clinical progression of Type 1 Diabetes. Immunol. Res..

[205] Tan X., Hu L., Shu Z., Chen L., Li X., Du M., Sun D., Mao X., Deng S., Huang K. (2019). Role of CCR2 in the Development of Streptozotocin-Treated Diabetic Cardiomyopathy. Diabetes.

[206] Cai H., Wang P., Zhang B., Dong X. (2020). Expression of the NEK7/NLRP3 inflammasome pathway in patients with diabetic lower extremity arterial disease. BMJ Open Diabetes Res. Care.

[207] Fang C., Huang Y., Pei Y., Zhang H.H., Chen X., Guo H., Li S., Ji X., Hu J. (2017). Genome-wide gene expression profiling reveals that CD274 is up-regulated new-onset type 1 diabetes mellitus. Acta Diabetol..

[208] Zhang Y., Lu Y., Gao Y., Liang X., Zhang R., Wang X., Zou X., Yang W. (2023). Effects of Aire on perforin expression in BMDCs via TLR7/8 and its therapeutic effect on type 1 diabetes. Int. Immunopharmacol..

[209] Broquères-You D., Leré-Déan C., Merkulova-Rainon T., Mantsounga C.S., Allanic D., Hainaud P., Contrères J.O., Wang Y., Vilar J., Virally M. (2012). Ephrin-B2-activated peripheral blood mononuclear cells from diabetic patients restore diabetes-induced impairment of postischemic neovascularization. Diabetes.

[210] Haddad D., Al Madhoun A., Nizam R., Al-Mulla F. (2020). Role of Caveolin-1 in Diabetes and Its Complications. Oxid. Med. Cell. Longev..

[211] Zbidi H., López J.J., Amor N.B., Bartegi A., Salido G.M., Rosado J.A. (2009). Enhanced expression of STIM1/Orai1 and TRPC3 in platelets from patients with type 2 diabetes mellitus. Blood Cells Mol. Dis..

[212] Shan T.D., Ouyang H., Yu T., Li J.Y., Huang C.Z., Yang H.S., Zhong W., Xia Z.S., Chen Q.K. (2016). iRNA-30e regulates abnormal differentiation of small intestinal epithelial cells in diabetic mice by downregulating Dll4 expression. Cell Prolif..

[213] Liu C., Liu Y., Yu Y., Zhao Y., Zhang D., Yu A. (2022). Identification of Up-Regulated ANXA3 Resulting in Fracture Non-Union in Patients with T2DM. Front. Endocrinol..

[214] Leurs P.B., van Oerle R., Wolffenbuttel B.H., Hamulyak K. (1997). Increased tissue factor pathway inhibitor (TFPI) and coagulation in patients with insulin-dependent diabetes mellitus. Thromb. Haemost..

[215] Li S.W., Wang J., Yang Y., Liu Z.J., Cheng L., Liu H.Y., Ma P., Luo W., Liu S.M. (2016). Polymorphisms in FADS1 and FADS2 alter plasma fatty acids and desaturase levels in type 2 diabetic patients with coronary artery disease. J. Transl. Med..

[216] Beyoğlu A., Kurutaş E.B., Karaküçük Y., Çömez A., Meşen A. (2022). Comparing the effects of serum GPER-1 and oxidant/antioxidant levels on retinopathy in patients with diabetes and healthy individuals: A pilot study. Arq. Bras. Oftalmol..

[217] Du B., Jia X., Tian W., Yan X., Wang N., Cai D., Li X., Zhang H., Jin M., Wu N. (2021). Associations of SUCNR1, GRK4, CAMK1D gene polymorphisms and the susceptibility of type 2 diabetes mellitus and essential hypertension in a northern Chinese Han population. J. Diabetes Complicat..

[218] Greenbaum L., Ravona-Springer R., Livny A., Shelly S., Sharvit-Ginon I., Ganmore I., Alkelai A., Heymann A., Schnaider Beeri M. (2019). The CADM2 gene is associated with processing speed performance—Evidence among elderly with type 2 diabetes. World J. Biol. Psychiatry.

[219] Porta M., Toppila I., Sandholm N., Hosseini S.M., Forsblom C., Hietala K., Borio L., Harjutsalo V., Klein B.E., Klein R. (2016). Variation in SLC19A3 and Protection from Microvascular Damage in Type 1 Diabetes. Diabetes.

[220] Yokoyama A., Mizukami T., Matsui T., Yokoyama T., Kimura M., Matsushita S., Higuchi S., Maruyama K. (2013). Genetic polymorphisms of alcohol dehydrogenase-1B and aldehyde dehydrogenase-2 and liver cirrhosis, chronic calcific pancreatitis, diabetes mellitus, and hypertension among Japanese alcoholic men. Alcohol Clin. Exp. Res..

[221] Wu G., Li G.B., Dai B., Zhang D.Q. (2014). Novel KIF6 polymorphism increases susceptibility to type 2 diabetes mellitus and coronary heart disease in Han Chinese men. J. Diabetes Res..

[222] Alarslan P., Unal Kocabas G., Demir I., Guler A., Bozkaya G., Aslanipour B., Calan M. (2020). Increased urocortin 3 levels are associated with the risk of having type 2 diabetes mellitus. J. Diabetes.

[223] Ramos-Lopez O., Mejia-Godoy R., Frías-Delgadillo K.J., Torres-Valadez R., Flores-García A., Sánchez-Enríquez S., Aguiar-García P., Martínez-López E., Zepeda-Carrillo E.A. (2019). Interactions between DRD2/ANKK1 TaqIA Polymorphism and Dietary Factors Influence Plasma Triglyceride Concentrations in Diabetic Patients from Western Mexico: A Cross-sectional Study. Nutrients.

[224] Gao C., Zhang W. (2019). Urinary AQP5 is independently associated with eGFR decline in patients with type 2 diabetes and nephropathy. Diabetes Res. Clin. Pract..

[225] Parveen S., Cheah P.H.S., Worthington L.P.I., Smither R.A., Munro M.L., Bussey C.T., Lamberts R.R., Jones P.P. (2023). Depressed HCN4 function in the type 2 diabetic sinoatrial node. Mol. Cell. Biochem..

[226] Crisford H., Sapey E., Stockley R.A. (2018). Proteinase 3; a potential target in chronic obstructive pulmonary disease and other chronic inflammatory diseases. Respir. Res..

[227] Zhang J., Patel J.M. (2010). Role of the CX3CL1-CX3CR1 axis in chronic inflammatory lung diseases. Int. J. Clin. Exp. Med..

[228] Lorenz E., Muhlebach M.S., Tessier P.A., Alexis N.E., Duncan Hite R., Seeds M.C., Peden D.B., Meredith W. (2008). Different expression ratio of S100A8/A9 and S100A12 in acute and chronic lung diseases. Respir. Med..

[229] He J.Q., Shumansky K., Connett J.E., Anthonisen N.R., Paré P.D., Sandford A.J. (2008). Association of genetic variations in the CSF2 and CSF3 genes with lung function in smoking-induced COPD. Eur. Respir. J..

[230] Zhang H., Li C., Song X., Cheng L., Liu Q., Zhang N., Wei L., Chung K., Adcock I.M., Ling C. (2021). Integrated analysis reveals lung fibrinogen gamma chain as a biomarker for chronic obstructive pulmonary disease. Ann. Transl. Med..

[231] Okutomo K., Fujino N., Yamada M., Saito T., Ono Y., Okada Y., Ichinose M., Sugiura H. (2022). Increased LHX9 expression in alveolar epithelial type 2 cells of patients with chronic obstructive pulmonary disease. Respir. Investig..

[232] Zhu A., Ge D., Zhang J., Teng Y., Yuan C., Huang M., Adcock I.M., Barnes P.J., Yao X. (2014). Sputum myeloperoxidase in chronic obstructive pulmonary disease. Eur. J. Med. Res..

[233] Jankowski M., Undas A., Kaczmarek P., Butenas S. (2011). Activated factor XI and tissue factor in chronic obstructive pulmonary disease: Links with inflammation and thrombin generation. Thromb. Res..

[234] Huang S.J., Ding Z.N., Xiang H.X., Fu L., Fei J. (2020). Association Between Serum S100A8/S100A9 Heterodimer and Pulmonary Function in Patients with Acute Exacerbation of Chronic Obstructive Pulmonary Disease. Lung.

[235] Porter J.C., Falzon M., Hall A. (2008). Polarized localization of epithelial CXCL11 in chronic obstructive pulmonary disease and mechanisms of T cell egression. J. Immunol..

[236] Chen C.Z., Ou C.Y., Wang R.H., Lee C.H., Lin C.C., Chang H.Y., Hsiue T.R. (2012). The role of bactericidal/permeability-increasing protein in men with chronic obstructive pulmonary disease. COPD.

[237] Papp C., Pak K., Erdei T., Juhasz B., Seres I., Szentpéteri A., Kardos L., Szilasi M., Gesztelyi R., Zsuga J. (2017). Alteration of the irisin-brain-derived neurotrophic factor axis contributes to disturbance of mood in COPD patients. Int. J. Chron. Obstruct. Pulmon. Dis..

[238] Jing H., Liu L., Zhou J., Yao H. (2018). Inhibition of C-X-C Motif Chemokine 10 (CXCL10) Protects Mice from Cigarette Smoke-Induced Chronic Obstructive Pulmonary Disease. Med. Sci. Monit..

[239] Kim R.Y., Sunkara K.P., Bracke K.R., Jarnicki A.G., Donovan C., Hsu A.C., Ieni A., Beckett E.L., Galvão I., Wijnant S. (2021). A microRNA-21-mediated SATB1/S100A9/NF-κB axis promotes chronic obstructive pulmonary disease pathogenesis. Sci. Transl. Med..

[240] Yi G., Liang M., Li M., Fang X., Liu J., Lai Y., Chen J., Yao W., Feng X., Hu L. (2018). A large lung gene expression study identifying IL1B as a novel player in airway inflammation in COPD airway epithelial cells. Inflamm. Res..

[241] Kaur M., Singh D. (2013). Neutrophil chemotaxis caused by chronic obstructive pulmonary disease alveolar macrophages: The role of CXCL8 and the receptors CXCR1/CXCR2. J. Pharmacol. Exp. Ther..

[242] Lazaar A.L., Miller B.E., Donald A.C., Keeley T., Ambery C., Russell J., Watz H., Tal-Singer R., for 205724 Investigators (2020). CXCR2 antagonist for patients with chronic obstructive pulmonary disease with chronic mucus hypersecretion: A phase 2b trial. Respir. Res..

[243] Kaur-Knudsen D., Bojesen S.E., Nordestgaard B.G. (2012). Cytochrome P450 1B1 and 2C9 genotypes and risk of ischemic vascular disease, cancer, and chronic obstructive pulmonary disease. Curr. Vasc. Pharmacol..

[244] Shi K., Chen X., Xie B., Yang S.S., Liu D., Dai G., Chen Q. (2018). Celastrol Alleviates Chronic Obstructive Pulmonary Disease by Inhibiting Cellular Inflammation Induced by Cigarette Smoke via the Ednrb/Kng1 Signaling Pathway. Front. Pharmacol..

[245] Didon L., Roos A.B., Elmberger G.P., Gonzalez F.J., Nord M. (2010). Lung-specific inactivation of CCAAT/enhancer binding protein alpha causes a pathological pattern characteristic of COPD. Eur. Respir. J..

[246] Yuan Y.M., Zhang J.L., Xu S.C., Ye R.S., Xu D., Zhang Y., Zhang Y.J., Chen Y.L., Liu Y.L., Su Z.G. (2016). Genetic variants of CDH13 determine the susceptibility to chronic obstructive pulmonary disease in a Chinese population. Acta Pharmacol. Sin..

[247] Reddy A.T., Lakshmi S.P., Banno A., Reddy R.C. (2018). Role of GPx3 in PPARγ-induced protection against COPD-associated oxidative stress. Free Radic. Biol. Med..

[248] Tan Y., Qiao Y., Chen Z., Liu J., Guo Y., Tran T., Tan K.S., Wang D.Y., Yan Y. (2020). FGF2, an Immunomodulatory Factor in Asthma and Chronic Obstructive Pulmonary Disease (COPD). Front. Cell Dev. Biol..

[249] Lahmar Z., Ahmed E., Fort A., Vachier I., Bourdin A., Bergougnoux A. (2022). Hedgehog pathway and its inhibitors in chronic obstructive pulmonary disease (COPD). Pharmacol. Ther..

[250] Cai J., Chen Q., Mehrabi Nasab E., Athari S.S. (2022). Immunomodulatory effect of N-acetyl-seryl-aspartyl-proline and vasoactive intestinal peptide on chronic obstructive pulmonary disease pathophysiology. Fundam. Clin. Pharmacol..

[251] Kureya Y., Kanazawa H., Ijiri N., Tochino Y., Watanabe T., Asai K., Hirata K. (2016). Down-Regulation of Soluble α-Klotho is Associated with Reduction in Serum Irisin Levels in Chronic Obstructive Pulmonary Disease. Lung.

[252] Springer J., Scholz F.R., Peiser C., Groneberg D.A., Fischer A. (2004). SMAD-signaling in chronic obstructive pulmonary disease: Transcriptional down-regulation of inhibitory SMAD 6 and 7 by cigarette smoke. Biol. Chem..

[253] Wang J., Zhang C., Zhang Z., Zheng Z., Sun D., Yang Q., Hadadi C., Li D., Xu X., Xiong M. (2016). A Functional Variant rs6435156C > T in BMPR2 is Associated with Increased Risk of Chronic Obstructive Pulmonary Disease (COPD) in Southern Chinese Population. eBioMedicine.

[254] Yazdani R., Marefati H., Shahesmaeili A., Nakhaei S., Bagheri A., Dastoorpoor M. (2018). Effect of Aerobic Exercises on Serum Levels of Apolipoprotein A1 and Apolipoprotein B, and Their Ratio in Patients with Chronic Obstructive Pulmonary Disease. Tanaffos.

[255] Calvén J., Yudina Y., Hallgren O., Westergren-Thorsson G., Davies D.E., Brandelius A., Uller L. (2012). Viral stimuli trigger exaggerated thymic stromal lymphopoietin expression by chronic obstructive pulmonary disease epithelium: Role of endosomal TLR3 and cytosolic RIG-I-like helicases. J. Innate Immun..

[256] He F., Wang N., Yu X., Zheng Y., Liu Q., Chen Q., Pu J., Li N., Zou W., Li B. (2022). GATA3/long noncoding RNA MHC-R regulates the immune activity of dendritic cells in chronic obstructive pulmonary disease induced by air pollution particulate matter. J. Hazard. Mater..

[257] Bai J., Song H., Cai C., Zhang M., Xu S., Tan J. (2012). The association of monocyte chemotactic protein-1 and CC chemokine receptor 2 gene variants with chronic obstructive pulmonary disease. DNA Cell Biol..

[258] Zhang T., Shang F., Ma Y., Xu Y., Sun W., Song H. (2023). Caveolin-1 Promotes the Imbalance of Th17/Treg in Chronic Obstructive Pulmonary Disease by Regulating Hsp70 Expression. Int. J. Chron. Obstruct. Pulmon. Dis..

[259] Shin S., Gombedza F.C., Awuah Boadi E., Yiu A.J., Roy S.K., Bandyopadhyay B.C. (2023). Reduction of TRPC1/TRPC3 mediated Ca^2+^-signaling protects oxidative stress-induced COPD. Cell. Signal..

[260] Yoo S., Takikawa S., Geraghty P., Argmann C., Campbell J., Lin L., Huang T., Tu Z., Foronjy R.F., Spira A. (2015). Integrative analysis of DNA methylation and gene expression data identifies EPAS1 as a key regulator of COPD. PLoS Genet..

[261] Angata T., Ishii T., Motegi T., Oka R., Taylor R.E., Soto P.C., Chang Y.C., Secundino I., Gao C.X., Ohtsubo K. (2013). Loss of Siglec-14 reduces the risk of chronic obstructive pulmonary disease exacerbation. Cell. Mol. Life Sci..

[262] Zhang M., Fang L., Zhou L., Molino A., Valentino M.R., Yang S., Zhang J., Li Y., Roth M. (2021). MAPK15-ULK1 signaling regulates mitophagy of airway epithelial cell in chronic obstructive pulmonary disease. Free Radic. Biol. Med..

[263] Lee J.H., McDonald M.L., Cho M.H., Wan E.S., Castaldi P.J., Hunninghake G.M., Marchetti N., Lynch D.A., Crapo J.D., Lomas D.A. (2014). DNAH5 is associated with total lung capacity in chronic obstructive pulmonary disease. Respir. Res..

[264] Hansel N.N., Sidhaye V., Rafaels N.M., Gao L., Gao P., Williams R., Connett J.E., Beaty T.H., Mathias R.A., Wise R.A. (2010). Aquaporin 5 polymorphisms and rate of lung function decline in chronic obstructive pulmonary disease. PLoS ONE.

[265] Maneerat Y., Prasongsukarn K., Benjathummarak S., Dechkhajorn W. (2017). PPBP and DEFA1/DEFA3 genes in hyperlipidaemia as feasible synergistic inflammatory biomarkers for coronary heart disease. Lipids Health Dis..

[266] Flamant M., Mougenot N., Balse E., Le Fèvre L., Atassi F., Gautier E.L., Le Goff W., Keck M., Nadaud S., Combadière C. (2021). Early activation of the cardiac CX3CL1/CX3CR1 axis delays β-adrenergic-induced heart failure. Sci. Rep..

[267] Freson K., De Vos R., Wittevrongel C., Thys C., Defoor J., Vanhees L., Vermylen J., Peerlinck K., Van Geet C. (2005). The TUBB1 Q43P functional polymorphism reduces the risk of cardiovascular disease in men by modulating platelet function and structure. Blood.

[268] Meng H., Du Z., Lu W., Wang Q., Sun X., Jiang Y., Wang Y., Li C., Tu P. (2021). Baoyuan decoction (BYD) attenuates cardiac hypertrophy through ANKRD1-ERK/GATA4 pathway in heart failure after acute myocardial infarction. Phytomedicine.

[269] He L., Huang C. (2017). MiR-19b and miR-16 cooperatively signaling target the regulator ADRA1A in Hypertensive heart disease. Biomed. Pharmacother..

[270] Lovely R.S., Yang Q., Massaro J.M., Wang J., D’Agostino R.B., O’Donnell C.J., Shannon J.D.H. (2011). Assessment of genetic determinants of the association of γ’ fibrinogen in relation to cardiovascular disease. Arterioscler. Thromb. Vasc. Biol..

[271] Li S., Hu D., Hu S., Sun Y., Zhang Y., Li H., Chen Y., Liu H., Cui G., Wang D.W. (2020). Association of rs2070600 in advanced glycosylation end-product specific receptor with prognosis of heart failure. ESC Heart Fail..

[272] Yamauchi K., Furui H., Taniguchi N., Sotobata I. (1986). Plasma beta-thromboglobulin and platelet factor 4 concentrations in patients with atrial fibrillation. Jpn. Heart J..

[273] Ruan J., Meng H., Wang X., Chen W., Tian X., Meng F. (2020). Low Expression of FFAR2 in Peripheral White Blood Cells May Be a Genetic Marker for Early Diagnosis of Acute Myocardial Infarction. Cardiol. Res. Pract..

[274] Janus S.E., Hajjari J., Chami T., Karnib M., Al-Kindi S.G., Rashid I. (2022). Myeloperoxidase is Independently Associated with Incident Heart Failure in Patients with Coronary Artery Disease and Kidney Disease. Curr. Probl. Cardiol..

[275] Agra-Bermejo R.M., Cacho-Antonio C., Rozados-Luis A., Couselo-Seijas M., Fernandez A.L., Martinez-Cereijo J.M., Bravo S.B., Gonzalez-Juanatey J.R., Eiras S. (2020). Macrophage Apoptosis Inhibitor, Was Identified in Epicardial Fat-Secretome and Regulated by Isoproterenol from Patients With Heart Failure. Front. Physiol..

[276] Wilhelmi T., Xu X., Tan X., Hulshoff M.S., Maamari S., Sossalla S., Zeisberg M., Zeisberg E.M. (2020). Serelaxin alleviates cardiac fibrosis through inhibiting endothelial-to-mesenchymal transition via RXFP1. Theranostics.

[277] Lova A., Pagán J., de la Morena G., Vázquez D.J., Cerezo-Manchado J.J., Bravo-Pérez C., Miñano A., Tomás A., Vicente V., Lozano M.L. (2023). Congenital factor XI deficiency and risk of heart failure in humans. J. Thromb. Haemost..

[278] Bai B., Xu Y., Chen H. (2023). Pathogenic roles of neutrophil-derived alarmins (S100A8/A9) in heart failure: From molecular mechanisms to therapeutic insights. Br. J. Pharmacol..

[279] Han Y., Hua S., Chen Y., Yang W., Zhao W., Huang F., Qiu Z., Yang C., Jiang J., Su X. (2021). irculating PGLYRP1 Levels as a Potential Biomarker for Coronary Artery Disease and Heart Failure. J. Cardiovasc. Pharmacol..

[280] Davidsson P., Eketjäll S., Eriksson N., Walentinsson A., Becker R.C., Cavallin A., Bogstedt A., Collén A., Held C., James S. (2023). ascular endothelial growth factor-D plasma levels and VEGFD genetic variants are independently associated with outcomes in patients with cardiovascular disease. Cardiovasc. Res..

[281] Zhang L., Hu A., Yuan H., Cui L., Miao G., Yang X., Wang L., Liu J., Liu X., Wang S. (2008). A missense mutation in the CHRM2 gene is associated with familial dilated cardiomyopathy. Circ. Res..

[282] Song X.M., Zheng X.Y., Zhu W.L., Huang L., Li Y. (2006). Relationship between polymorphism of cystathionine beta synthase gene and congenital heart disease in Chinese nuclear families. Biomed. Environ. Sci..

[283] Yu Y., Song G. (2020). Lipopolysaccharide-Binding Protein and Bactericidal/Permeability-Increasing Protein in Lipid Metabolism and Cardiovascular Diseases. Adv. Exp. Med. Biol..

[284] Theis J.L., Vogler G., Missinato M.A., Li X., Nielsen T., Zeng X.I., Martinez-Fernandez A., Walls S.M., Kervadec A., Kezos J.N. (2020). Patient-specific genomics and cross-species functional analysis implicate LRP2 in hypoplastic left heart syndrome. eLife.

[285] Cannavo A., Jun S., Rengo G., Marzano F., Agrimi J., Liccardo D., Elia A., Keceli G., Altobelli G.G., Marcucci L. (2023). β3AR-Dependent Brain-Derived Neurotrophic Factor (BDNF) Generation Limits Chronic Postischemic Heart Failure. Circ. Res..

[286] Heliö K., Mäyränpää M.I., Saarinen I., Ahonen S., Junnila H., Tommiska J., Weckström S., Holmström M., Toivonen M., Nikus K. (2021). GRINL1A Complex Transcription Unit Containing GCOM1, MYZAP, and POLR2M Genes Associates with Fully Penetrant Recessive Dilated Cardiomyopathy. Front. Genet..

[287] Altara R., Manca M., Hessel M.H., Gu Y., van Vark L.C., Akkerhuis K.M., Staessen J.A., Struijker-Boudier H.A., Booz G.W., Blankesteijn W.M. (2016). CXCL10 Is a Circulating Inflammatory Marker in Patients with Advanced Heart Failure: A Pilot Study. J. Cardiovasc. Transl. Res..

[288] Zhang C., He X., Zhao J., Cao Y., Liu J., Liang W., Zhou Y., Wang C., Xue R., Dong Y. (2020). Angiopoietin-Like Protein 7 and Short-Term Mortality in Acute Heart Failure. Cardiorenal. Med..

[289] Moreira J.B.N., Wohlwend M., Fenk S., Åmellem I., Flatberg A., Kraljevic J., Marinovic J., Ljubkovic M., Bjørkøy G., Wisløff U. (2019). Exercise Reveals Proline Dehydrogenase as a Potential Target in Heart Failure. Prog. Cardiovasc. Dis..

[290] Timur A.A., Murugesan G., Zhang L., Aung P.P., Barnard J., Wang Q.K., Gaussem P., Silverstein R.L., Bhatt D.L., Kottke-Marchant K. (2012). P2RY1 and P2RY12 polymorphisms and on-aspirin platelet reactivity in patients with coronary artery disease. Int. J. Lab. Hematol..

[291] Li R., Fang J., Huo B., Su Y.S., Wang J., Liu L.G., Hu M., Cheng C., Zheng P., Zhu X.H. (2017). Leucine-rich repeat neuronal protein 4 (LRRN4) potentially functions in dilated cardiomyopathy. Int. J. Clin. Exp. Pathol..

[292] Feng L., Li G., An J., Liu C., Zhu X., Xu Y., Gao Y., Li J., Liu J., Yan J. (2022). Exercise Training Protects Against Heart Failure Via Expansion of Myeloid-Derived Suppressor Cells Through Regulating IL-10/STAT3/S100A9 Pathway. Circ. Heart Fail..

[293] Dhayni K., Zibara K., Issa H., Kamel S., Bennis Y. (2022). Targeting CXCR1 and CXCR2 receptors in cardiovascular diseases. Pharmacol. Ther..

[294] Abdalla M., El-Arabey A.A., Gai Z. (2023). Interplay between LPL and GPIHBP1 in COVID-19 patients: A possible mechanism for post-recovery cardiomyopathy. Hum. Cell.

[295] Streff H., Bi W., Colón A.G., Adesina A.M., Miyake C.Y., Lalani S.R. (2019). Amish nemaline myopathy and dilated cardiomyopathy caused by a homozygous contiguous gene deletion of TNNT1 and TNNI3 in a Mennonite child. Eur. J. Med. Genet..

[296] Zhang Y., Zhang L., Fan X., Yang W., Yu B., Kou J., Li F. (2019). Captopril attenuates TAC-induced heart failure via inhibiting Wnt3a/β-catenin and Jak2/Stat3 pathways. Biomed. Pharmacother..

[297] Yang W., Wu Z., Yang K., Han Y., Chen Y., Zhao W., Huang F., Jin Y., Jin W. (2019). BMI1 promotes cardiac fibrosis in ischemia-induced heart failure via the PTEN-PI3K/Akt-mTOR signaling pathway. Am. J. Physiol. Heart Circ. Physiol..

[298] Mir R., Elfaki I., Jha C.K., Javid J., Babakr A.T., Banu S., Mir M.M., Jamwal D., Khullar N., Alzahrani K.J. (2021). Biological and Clinical Implications of TNF-α Promoter and CYP1B1 Gene Variations in Coronary Artery Disease Susceptibility. Cardiovasc. Hematol. Disord. Drug Targets.

[299] Zheng P.F., Liu F., Zheng Z.F., Pan H.W., Liu Z.Y. (2023). Identification MNS1, FRZB, OGN, LUM, SERP1NA3 and FCN3 as the potential immune-related key genes involved in ischaemic cardiomyopathy by random forest and nomogram. Aging.

[300] Virk Z.M., Richardson T.L., Nowatzke J.F., Ullah A., Pedrotty D.M., Shoemaker M.B., Kanagasundram A., Roden D.M., Stevenson W.G. (2023). Cardiac Sarcoidosis and a Likely Pathogenic TTN Variant in a Patient Presenting with Ventricular Tachycardia. JACC Case Rep..

[301] Sheikh-Hamad D., Bick R., Wu G.Y., Christensen B.M., Razeghi P., Poindexter B., Taegtmeyer H., Wamsley A., Padda R., Entman M. (2003). Stanniocalcin-1 is a naturally occurring L-channel inhibitor in cardiomyocytes: Relevance to human heart failure. Am. J. Physiol. Heart Circ. Physiol..

[302] Glatz J.F.C., Wang F., Nabben M., Luiken J.J.F.P. (2021). CD36 as a target for metabolic modulation therapy in cardiac disease. Expert Opin. Ther. Targets.

[303] Thorolfsdottir R.B., Sveinbjornsson G., Sulem P., Nielsen J.B., Jonsson S., Halldorsson G.H., Melsted P., Ivarsdottir E.V., Davidsson O.B., Kristjansson R.P. (2018). Coding variants in RPL3L and MYZAP increase risk of atrial fibrillation. Commun. Biol..

[304] Prudente S., Sesti G., Pandolfi A., Andreozzi F., Consoli A., Trischitta V. (2012). The mammalian tribbles homolog TRIB3, glucose homeostasis, and cardiovascular diseases. Endocr. Rev..

[305] Zhao M., Zheng Z., Yin Z., Zhang J., Qin J., Wan J., Wang M. (2023). Resolvin D2 and its receptor GPR18 in cardiovascular and metabolic diseases: A promising biomarker and therapeutic target. Pharmacol. Res..

[306] Hassoun R., Budde H., Mannherz H.G., Lódi M., Fujita-Becker S., Laser K.T., Gärtner A., Klingel K., Möhner D., Stehle R. (2021). De Novo Missense Mutations in TNNC1 and TNNI3 Causing Severe Infantile Cardiomyopathy Affect Myofilament Structure and Function and Are Modulated by Troponin Targeting Agents. Int. J. Mol. Sci..

[307] Clausen A.G., Vad O.B., Andersen J.H., Olesen M.S. (2021). Loss-of-Function Variants in the SYNPO2L Gene Are Associated with Atrial Fibrillation. Front. Cardiovasc. Med..

[308] Mok C.C., Ho L.Y., Chan K.L., Tse S.M., To C.H. (2023). Circulating Proprotein Convertase Subtilisin/Kexin Type 9 (PCSK9) is Associated with Disease Activity and Risk of Incident Cardiovascular Disease in Patients with Systemic Lupus Erythematosus. Inflammation.

[309] Wang C., Yin S., Wang Q., Jiang M., Li S., Zhen W., Duan Y., Gu H. (2022). miR-409-3p Regulated by GATA2 Promotes Cardiac Fibrosis through Targeting Gpd1. Oxid. Med. Cell. Longev..

[310] O’Connell T.D., Block R.C., Huang S.P., Shearer G.C. (2017). ω3-Polyunsaturated fatty acids for heart failure: Effects of dose on efficacy and novel signaling through free fatty acid receptor 4. J. Mol. Cell. Cardiol..

[311] Decharatchakul N., Settasatian C., Settasatian N., Komanasin N., Kukongviriyapan U., Intharapetch P., Senthong V., Sawanyawisuth K. (2020). Association of combined genetic variations in SOD3, GPX3, PON1, and GSTT1 with hypertension and severity of coronary artery disease. Heart Vessels.

[312] Zhang M., Zhang M., Zhou T., Liu M., Xia N., Gu M., Tang T., Nie S., Zhu Z., Lv B. (2021). Inhibition of fibroblast IL-6 production by ACKR4 deletion alleviates cardiac remodeling after myocardial infarction. Biochem. Biophys. Res. Commun..

[313] Gallo G., Forte M., Cotugno M., Marchitti S., Stanzione R., Tocci G., Bianchi F., Palmerio S., Scioli M., Frati G. (2023). Polymorphic variants at NDUFC2, encoding a mitochondrial complex I subunit, associate with cardiac hypertrophy in human hypertension. Mol. Med..

[314] Yuan C., Chen Z., Zhou Q. (2023). Crocin inhibits KBTBD7 to prevent excessive inflammation and cardiac dysfunction following myocardial infarction. Mol. Med. Rep..

[315] Peng R., Li B., Chen S., Shi Z., Yu L., Gao Y., Yang X., Lu L., Wang H. (2022). Deleterious Rare Mutations of GLI1 Dysregulate Sonic Hedgehog Signaling in Human Congenital Heart Disease. Front. Cardiovasc. Med..

[316] Raftrey B., Williams M., Rios Coronado P.E., Fan X., Chang A.H., Zhao M., Roth R., Trimm E., Racelis R., D’Amato G. (2021). Dach1 Extends Artery Networks and Protects Against Cardiac Injury. Circ. Res..

[317] Wu J.T., Liu S.S., Xie X.J., Liu Q., Xin Y.N., Xuan S.Y. (2020). Independent and joint correlation of PNPLA3 I148M and TM6SF2 E167K variants with the risk of coronary heart disease in patients with non-alcoholic fatty liver disease. Lipids Health Dis..

[318] Kumar D., Narang R., Saluja D., Srivastava K. (2022). Functional Association of miR-133b and miR-21 Through Novel Gene Targets ATG5, LRP6 and SGPP1 in Coronary Artery Disease. Mol. Diagn. Ther..

[319] Kupari M., Mikkola T.S., Turto H., Lommi J., Ylikorkala O. (2006). Vasoactive intestinal peptide—Release from the heart and response in heart failure due to left ventricular pressure overload. Eur. J. Heart Fail..

[320] Ortega A., Tarazón E., Roselló-Lletí E., Gil-Cayuela C., Lago F., González-Juanatey J.R., Cinca J., Jorge E., Martínez-Dolz L., Portolés M. (2015). Patients with Dilated Cardiomyopathy and Sustained Monomorphic Ventricular Tachycardia Show Up-Regulation of KCNN3 and KCNJ2 Genes and CACNG8-Linked Left Ventricular Dysfunction. PLoS ONE.

[321] Wang K., Li Z., Li Y., Liu X., Sun Y., Hong J., Ding Y., Zheng W., Qian L., Xu D. (2022). Cardioprotection of Klotho against myocardial infarction-induced heart failure through inducing autophagy. Mech. Ageing Dev..

[322] Li N., Zhu L., Zhu C., Zhou H., Zheng D., Xu G., Shi H., Gao J., Li A.J., Wang Z. (2021). BMPR2 promoter methylation and its expression in valvular heart disease complicated with pulmonary artery hypertension. Aging.

[323] Yu W., Dujiang X., Yi W., Guanwen D., Mengyu Z., Chang P., Aikai Z., Juan Z., Linlin Z., Hang Z. (2022). Apolipoprotein A1 is associated with pulmonary vascular resistance and adverse clinical outcomes in patients with pulmonary hypertension secondary to heart failure. Pulm. Circ..

[324] Totsune K., Takahashi K., Mackenzie H.S., Murakami O., Arihara Z., Sone M., Mouri T., Brenner B.M., Ito S. (2000). Increased gene expression of adrenomedullin and adrenomedullin-receptor complexes, receptor-activity modifying protein (RAMP)2 and calcitonin-receptor-like receptor (CRLR) in the hearts of rats with congestive heart failure. Clin. Sci..

[325] Liu X., Li Y., Wang L., Zhao Q., Lu X., Huang J., Fan Z., Gu D. (2008). The INSIG1 gene, not the INSIG2 gene, associated with coronary heart disease: TagSNPs and haplotype-based association study. The Beijing Atherosclerosis Study. Thromb. Haemost..

[326] Liu Y., Chen L., Gao L., Pei X., Tao Z., Xu Y., Li R. (2022). LRRK2 deficiency protects the heart against myocardial infarction injury in mice via the P53/HMGB1 pathway. Free Radic. Biol. Med..

[327] Tang X., Pan L., Zhao S., Dai F., Chao M., Jiang H., Li X., Lin Z., Huang Z., Meng G. (2020). SNO-MLP (S-Nitrosylation of Muscle LIM Protein) Facilitates Myocardial Hypertrophy Through TLR3 (Toll-Like Receptor 3)-Mediated RIP3 (Receptor-Interacting Protein Kinase 3) and NLRP3 (NOD-Like Receptor Pyrin Domain Containing 3) Inflammasome Activation. Circulation.

[328] Lv L., Li A., Jiang L., Zhang L. (2023). Deficiency of HTR4 and ADRB1 caused by SARS-CoV-2 spike may partially explain multiple COVID-19 related syndromes including depression, cognitive impairment, loss of appetite, heart failure, and hypertension. J. Infect..

[329] Wang L., Chen J., Zeng Y., Wei J., Jing J., Li G., Su L., Tang X., Wu T., Zhou L. (2016). Functional Variant in the SLC22A3-LPAL2-LPA Gene Cluster Contributes to the Severity of Coronary Artery Disease. Arterioscler. Thromb. Vasc. Biol..

[330] Su H., Hu K., Liu Z., Chen K., Xu J. (2021). Carbonic anhydrase 2 and 3 as risk biomarkers for dilated cardiomyopathy associated heart failure. Ann. Palliat. Med..

[331] Yao J., Hou J., Lv L., Song C., Zhang M., Wu Z. (2021). Does Decreased SNX10 Serve as a Novel Risk Factor in Atrial Fibrillation of the Valvular Heart Disease?—A Case-Control Study. Braz. J. Cardiovasc. Surg..

[332] López N., Varo N., Díez J., Fortuño M.A. (2007). Loss of myocardial LIF receptor in experimental heart failure reduces cardiotrophin-1 cytoprotection. A role for neurohumoral agonists?. Cardiovasc. Res..

[333] Satoh M., Akatsu T., Ishikawa Y., Minami Y., Takahashi Y., Nakamura M. (2007). Association between toll-like receptor 8 expression and adverse clinical outcomes in patients with enterovirus-associated dilated cardiomyopathy. Am. Heart J..

[334] Wu W., Bao W., Chen X., Lu Y., Fang J., Liu J., Peng S., Pi J., Tomlinson B., Chan P. (2023). Endothelial Gata6 deletion reduces monocyte recruitment and proinflammatory macrophage formation and attenuates atherosclerosis through Cmpk2-Nlrp3 pathways. Redox Biol..

[335] Yang M., Song L., Wang L., Yukht A., Ruther H., Li F., Qin M., Ghiasi H., Sharifi B.G., Shah P.K. (2018). Deficiency of GATA3-Positive Macrophages Improves Cardiac Function Following Myocardial Infarction or Pressure Overload Hypertrophy. J. Am. Coll. Cardiol..

[336] Singla B., Lin H.P., Chen A., Ahn W., Ghoshal P., Cherian-Shaw M., White J., Stansfield B.K., Csányi G. (2021). Role of R-spondin 2 in arterial lymphangiogenesis and atherosclerosis. Cardiovasc. Res..

[337] Li Y., Sun X., Liu X., Li J., Li X., Wang G., Liu Y., Lu X., Cui L., Shao M. (2022). P2X7R-NEK7-NLRP3 Inflammasome Activation: A Novel Therapeutic Pathway of Qishen Granule in the Treatment of Acute Myocardial Ischemia. J. Inflamm. Res..

[338] Hasham M.G., Baxan N., Stuckey D.J., Branca J., Perkins B., Dent O., Duffy T., Hameed T.S., Stella S.E., Bellahcene M. (2017). Systemic autoimmunity induced by the TLR7/8 agonist Resiquimod causes myocarditis and dilated cardiomyopathy in a new mouse model of autoimmune heart disease. Dis. Models Mech..

[339] Accornero F., Schips T.G., Petrosino J.M., Gu S.Q., Kanisicak O., van Berlo J.H., Molkentin J.D. (2017). BEX1 is an RNA-dependent mediator of cardiomyopathy. Nat. Commun..

[340] Su S.A., Yang D., Wu Y., Xie Y., Zhu W., Cai Z., Shen J., Fu Z., Wang Y., Jia L. (2017). EphrinB2 Regulates Cardiac Fibrosis Through Modulating the Interaction of Stat3 and TGF-β/Smad3 Signaling. Circ. Res..

[341] Wunderlich C., Schober K., Lange S.A., Drab M., Braun-Dullaeus R.C., Kasper M., Schwencke C., Schmeisser A., Strasser R.H. (2006). Disruption of caveolin-1 leads to enhanced nitrosative stress and severe systolic and diastolic heart failure. Biochem. Biophys. Res. Commun..

[342] Zhang F., Xu X., Hou J., Xiao H., Guo F., Li X., Yang H. (2023). Cardioprotective efficacy of Xin-shu-bao tablet in heart failure with reduced ejection fraction by modulating THBD/ARRB1/FGF1/STIM1 signaling. Biomed. Pharmacother..

[343] Tang S.G., Liu X.Y., Wang S.P., Wang H.H., Jovanović A., Tan W. (2019). Trimetazidine prevents diabetic cardiomyopathy by inhibiting Nox2/TRPC3-induced oxidative stress. J. Pharmacol. Sci..

[344] De Vries M.A., Trompet S., Mooijaart S.P., Smit R.A., Böhringer S., Castro Cabezas M., Jukema J.W. (2017). Complement receptor 1 gene polymorphisms are associated with cardiovascular risk. Atherosclerosis.

[345] Wen Z.Q., Li S.H., Shui X., Tang L.L., Zheng J.R., Chen L. (2019). LncRNA PEG10 aggravates cardiac hypertrophy through regulating HOXA9. Eur. Rev. Med. Pharmacol. Sci..

[346] You J., Wu J., Jiang G., Guo J., Wang S., Li L., Ge J., Zou Y. (2013). Olmesartan attenuates cardiac remodeling through DLL4/Notch1 pathway activation in pressure overload mice. J. Cardiovasc. Pharmacol..

[347] Hermansson C., Lundqvist A., Wasslavik C., Palmqvist L., Jeppsson A., Hultén L.M. (2013). Reduced expression of NLRP3 and MEFV in human ischemic heart tissue. Biochem. Biophys. Res. Commun..

[348] Falciani M., Gori A.M., Fedi S., Chiarugi L., Simonetti I., Dabizzi R.P., Prisco D., Pepe G., Abbate R., Gensini G.F. (1998). Elevated tissue factor and tissue factor pathway inhibitor circulating levels in ischaemic heart disease patients. Thromb. Haemost..

[349] Heaton M.P., Bassett A.S., Whitman K.J., Krafsur G.M., Lee S.I., Carlson J.M., Clark H.J., Smith H.R., Pelster M.C., Basnayake V. (2019). Evaluation of EPAS1 variants for association with bovine congestive heart failure. F1000Research.

[350] Li X.X., Mu B., Li X., Bie Z.D. (2022). circCELF1 Inhibits Myocardial Fibrosis by Regulating the Expression of DKK2 Through FTO/m6A and miR-636. J. Cardiovasc. Transl. Res..

[351] Zhang J., Wu L., Li Z., Fu G. (2017). miR-1231 exacerbates arrhythmia by targeting calciumchannel gene CACNA2D2 in myocardial infarction. Am. J. Transl. Res..

[352] Bergeman A.T., Hoeksema W.F., van der Ree M.H., Boersma L.V.A., Yap S.C., Verheul L.M., Hassink R.J., van der Crabben S.N., Volders P.G.A., van der Werf C. (2023). Outcomes in Dutch DPP6 risk haplotype for familial idiopathic ventricular fibrillation: A focused update. Neth. Heart J..

[353] Kang G.J., Xie A., Kim E., Dudley S.C. (2023). miR-448 regulates potassium voltage-gated channel subfamily A member 4 (KCNA4) in ischemia and heart failure. Heart Rhythm.

[354] Long Y., Wang L., Li Z. (2020). SP1-induced SNHG14 aggravates hypertrophic response in in vitro model of cardiac hypertrophy via up-regulation of PCDH17. J. Cell. Mol. Med..

[355] Bruikman C.S., Dalila N., van Capelleveen J.C., Kroon J., Peter J., Havik S.R., Willems M., Huisman L.C., de Boer O.J., Hovingh G.K. (2020). Genetic variants in SUSD2 are associated with the risk of ischemic heart disease. J. Clin. Lipidol..

[356] Majdalani P., Levitas A., Krymko H., Slanovic L., Braiman A., Hadad U., Dabsan S., Horev A., Zarivach R., Parvari R. (2023). A Missense Variation in PHACTR2 Associates with Impaired Actin Dynamics, Dilated Cardiomyopathy, and Left Ventricular Non-Compaction in Humans. Int. J. Mol. Sci..

[357] Chen W., Zhang Y., Shen L., Zhu J., Cai K., Lu Z., Zeng W., Zhao J., Zhou X. (2022). Biallelic DNAH9 mutations are identified in Chinese patients with defective left-right patterning and cilia-related complex congenital heart disease. Hum. Genet..

[358] Shen S.W., Yin R.X., Huang F., Wu J.Z., Cao X.L., Chen W.X. (2017). DNAH11 rs12670798 variant and G × E interactions on serum lipid levels, coronary heart disease, ischemic stroke and the lipid-lowering efficacy of atorvastatin. Int. J. Clin. Exp. Pathol..

[359] Deniz E., Pasha M., Guerra M.E., Viviano S., Ji W., Konstantino M., Jeffries L., Lakhani S.A., Medne L., Skraban C. (2023). CFAP45, a heterotaxy and congenital heart disease gene, affects cilia stability. Dev. Biol..

[360] Nöthe-Menchen T., Wallmeier J., Pennekamp P., Höben I.M., Olbrich H., Loges N.T., Raidt J., Dougherty G.W., Hjeij R., Dworniczak B. (2019). Randomization of Left-right Asymmetry and Congenital Heart Defects: The Role of DNAH5 in Humans and Mice. Circ. Genom. Precis. Med..

[361] Padua M.B., Helm B.M., Wells J.R., Smith A.M., Bellchambers H.M., Sridhar A., Ware S.M. (2023). Congenital heart defects caused by FOXJ1. Hum. Mol. Genet..

[362] Jiang X., Shao M., Liu X., Liu X., Zhang X., Wang Y., Yin K., Wang S., Hu Y., Jose P.A. (2021). Reversible Treatment of Pressure Overload-Induced Left Ventricular Hypertrophy through Drd5 Nucleic Acid Delivery Mediated by Functional Polyaminoglycoside. Adv. Sci..

[363] Stirrat C.G., Venkatasubramanian S., Pawade T., Mitchell A.J., Shah A.S., Lang N.N., Newby D.E. (2016). Cardiovascular effects of urocortin 2 and urocortin 3 in patients with chronic heart failure. Br. J. Clin. Pharmacol..

[364] Jacondino C.B., Borges C.A., Rosemberg L.S., da Silva I.G., da Luz Correa B., Valle Gottlieb M.G. (2019). Association of oxytocin levels and oxytocin receptor gene polymorphism (rs2254298) with cardiovascular risk factors in Brazilian elderly from Primary Health Care. Arch. Gerontol. Geriatr..

[365] Mayer O., Seidlerová J., Černá V., Kučerová A., Bruthans J., Vágovičová P., Vaněk J., Timoracká K., Wohlfahrt P., Filipovský J. (2015). The DRD2/ANKK1 Taq1A polymorphism is associated with smoking cessation failure in patients with coronary heart disease. Pers. Med..

[366] Paszkowska A., Piekutowska-Abramczuk D., Ciara E., Mirecka-Rola A., Brzezinska M., Wicher D., Kostrzewa G., Sarnecki J., Ziółkowska L. (2022). Clinical Presentation of Left Ventricular Noncompaction Cardiomyopathy and Bradycardia in Three Families Carrying HCN4 Pathogenic Variants. Genes.

[367] Tejera P., Wang Z., Zhai R., Su L., Sheu C.C., Taylor D.M., Chen F., Gong M.N., Thompson B.T., Christiani D.C. (2009). Genetic polymorphisms of peptidase inhibitor 3 (elafin) are associated with acute respiratory distress syndrome. Am. J. Respir. Cell Mol. Biol..

[368] Doggrell S.A. (2011). CX3CR1 as a target for airways inflammation. Expert Opin. Ther. Targets.

[369] Camoretti-Mercado B., Karrar E., Nuñez L., Bowman M.A. (2012). S100A12 and the Airway Smooth Muscle: Beyond Inflammation and Constriction. J. Allergy Ther..

[370] Haegens A., Heeringa P., van Suylen R.J., Steele C., Aratani Y., O’Donoghue R.J., Mutsaers S.E., Mossman B.T., Wouters E.F., Vernooy J.H. (2009). Myeloperoxidase deficiency attenuates lipopolysaccharide-induced acute lung inflammation and subsequent cytokine and chemokine production. J. Immunol..

[371] Weng D., Gao S., Shen H., Yao S., Huang Q., Zhang Y., Huang W., Wang Y., Zhang X., Yin Y. (2022). CD5L attenuates allergic airway inflammation by expanding CD11chigh alveolar macrophages and inhibiting NLRP3 inflammasome activation via HDAC2. Immunology.

[372] Van Crombruggen K., Vogl T., Pérez-Novo C., Holtappels G., Bachert C. (2016). Differential release and deposition of S100A8/A9 proteins in inflamed upper airway tissue. Eur. Respir. J..

[373] Kameda M., Otsuka M., Chiba H., Kuronuma K., Hasegawa T., Takahashi H., Takahashi H. (2020). CXCL9, CXCL10, and CXCL11; biomarkers of pulmonary inflammation associated with autoimmunity in patients with collagen vascular diseases-associated interstitial lung disease and interstitial pneumonia with autoimmune features. PLoS ONE.

[374] Harkin D.W., Barros D’Sa A.A.B., McCallion K., Hoper M., Halliday M.I., Campbell F.C. (2001). Bactericidal/permeability-increasing protein attenuates systemic inflammation and acute lung injury in porcine lower limb ischemia-reperfusion injury. Ann. Surg..

[375] Li Y., Lu H., Lv X., Tang Q., Li W., Zhu H., Long Y. (2018). Blockade of Aquaporin 4 Inhibits Irradiation-Induced Pulmonary Inflammation and Modulates Macrophage Polarization in Mice. Inflammation.

[376] Braun A., Lommatzsch M., Neuhaus-Steinmetz U., Quarcoo D., Glaab T., McGregor G.P., Fischer A., Renz H. (2004). Brain-derived neurotrophic factor (BDNF) contributes to neuronal dysfunction in a model of allergic airway inflammation. Br. J. Pharmacol..

[377] Dong S., Zhang X., He Y., Xu F., Li D., Xu W., Wang H., Yin Y., Cao J. (2013). Synergy of IL-27 and TNF-α in regulating CXCL10 expression in lung fibroblasts. Am. J. Respir. Cell Mol. Biol..

[378] Dulek D.E., Newcomb D.C., Goleniewska K., Cephus J., Zhou W., Reiss S., Toki S., Ye F., Zaynagetdinov R., Sherrill T.P. (2014). Allergic airway inflammation decreases lung bacterial burden following acute Klebsiella pneumoniae infection in a neutrophil- and CCL8-dependent manner. Infect. Immun..

[379] Palmer L.D., Maloney K.N., Boyd K.L., Goleniewska A.K., Toki S., Maxwell C.N., Chazin W.J., Peebles R.S., Newcomb D.C., Skaar E.P. (2019). The Innate Immune Protein S100A9 Protects from T-Helper Cell Type 2-mediated Allergic Airway Inflammation. Am. J. Respir. Cell Mol. Biol..

[380] Mattos M.S., Ferrero M.R., Kraemer L., Lopes G.A.O., Reis D.C., Cassali G.D., Oliveira F.M.S., Brandolini L., Allegretti M., Garcia C.C. (2020). CXCR1 and CXCR2 Inhibition by Ladarixin Improves Neutrophil-Dependent Airway Inflammation in Mice. Front. Immunol..

[381] Lazaar A.L., Sweeney L.E., MacDonald A.J., Alexis N.E., Chen C., Tal-Singer R. (2011). SB-656933, a novel CXCR2 selective antagonist, inhibits ex vivo neutrophil activation and ozone-induced airway inflammation in humans. Br. J. Clin. Pharmacol..

[382] Zhang S., Lu X., Fang X., Wang Z., Cheng S., Song J. (2022). Cigarette smoke extract combined with LPS reduces ABCA3 expression in chronic pulmonary inflammation may be related to PPARγ/P38 MAPK signaling pathway. Ecotoxicol. Environ. Saf..

[383] Yuan Y., Liao Q., Xue M., Shi Y., Rong L., Song Z., Tong Z., Zheng W., Zhu Q., Cui X. (2018). Shufeng Jiedu Capsules Alleviate Lipopolysaccharide-Induced Acute Lung Inflammatory Injury via Activation of GPR18 by Verbenalin. Cell. Physiol. Biochem..

[384] Onoue S., Misaka S., Aoki Y., Karaki S., Kuwahara A., Ohide A., Mizumoto T., Yamada S. (2010). Inhalable powder formulation of vasoactive intestinal peptide derivative, [R15,20,21, L17]-VIP-GRR, attenuated neutrophilic airway inflammation in cigarette smoke-exposed rats. Eur. J. Pharm. Sci..

[385] Krick S., Grabner A., Baumlin N., Yanucil C., Helton S., Grosche A., Sailland J., Geraghty P., Viera L., Russell D.W. (2018). Fibroblast growth factor 23 and Klotho contribute to airway inflammation. Eur. Respir. J..

[386] Jiang J.J., Chen S.M., Li H.Y., Xie Q.M., Yang Y.M. (2021). TLR3 inhibitor and tyrosine kinase inhibitor attenuate cigarette smoke/poly I:C-induced airway inflammation and remodeling by the EGFR/TLR3/MAPK signaling pathway. Eur. J. Pharmacol..

[387] Li M., Xue Y., Miao X., Ma P., Kong X., Jin Y., Li Y., Wang W., Zhang Q., Deng Q. (2023). NLRP12 attenuates ozone-induced pulmonary inflammation by regulating canonical NF-κB Pathway. Ecotoxicol. Environ. Saf..

[388] Lee C.C., Huang H.Y., Chiang B.L. (2008). Lentiviral-mediated GATA-3 RNAi decreases allergic airway inflammation and hyperresponsiveness. Mol. Ther..

[389] Pajulas A., Fu Y., Cheung C.C.L., Chu M., Cannon A., Alakhras N., Zhang J., Ulrich B.J., Nelson A.S., Zhou B. (2023). Interleukin-9 promotes mast cell progenitor proliferation and CCR2-dependent mast cell migration in allergic airway inflammation. Mucosal Immunol..

[390] Khan A.R., Amu S., Saunders S.P., Hams E., Blackshields G., Leonard M.O., Weaver C.T., Sparwasser T., Sheils O., Fallon P.G. (2015). Ligation of TLR7 on CD19(+) CD1d(hi) B cells suppresses allergic lung inflammation via regulatory T cells. Eur. J. Immunol..

[391] Li H.T., Ye C., Zhou M., Yang Y., Jin Q., Pan C.F. (2019). Moxifloxacin suppresses airway inflammation and modulates expression of caveolin-1 and flotillin-1 in airway smooth muscle cells of asthmatic rats. Ann. Transl. Med..

[392] Hamacher J., Sadallah S., Schifferli J.A., Villard J., Nicod L.P. (1998). Soluble complement receptor type 1 (CD35) in bronchoalveolar lavage of inflammatory lung diseases. Eur. Respir. J..

[393] Huang M.T., Chen Y.L., Lien C.I., Liu W.L., Hsu L.C., Yagita H., Chiang B.L. (2017). Notch Ligand DLL4 Alleviates Allergic Airway Inflammation via Induction of a Homeostatic Regulatory Pathway. Sci. Rep..

[394] Ijaz B., Shabbir A., Shahzad M., Mobashar A., Sharif M., Basheer M.I., Tareen R.B., Syed N.I. (2021). Amelioration of airway inflammation and pulmonary edema by Teucrium stocksianum via attenuation of pro-inflammatory cytokines and up-regulation of AQP1 and AQP5. Respir. Physiol. Neurobiol..

[395] Cai H., Chen S., Li X., Liu H., Zhang Y., Zhuang Q. (2022). The Combined Model of CX3CR1-Related Immune Infiltration Genes to Evaluate the Prognosis of Idiopathic Pulmonary Fibrosis. Front. Immunol..

[396] Li Y., He Y., Chen S., Wang Q., Yang Y., Shen D., Ma J., Wen Z., Ning S., Chen H. (2022). S100A12 as Biomarker of Disease Severity and Prognosis in Patients with Idiopathic Pulmonary Fibrosis. Front. Immunol..

[397] Watanabe T., Minezawa T., Hasegawa M., Goto Y., Okamura T., Sakakibara Y., Niwa Y., Kato A., Hayashi M., Isogai S. (2019). Prognosis of pulmonary fibrosis presenting with a usual interstitial pneumonia pattern on computed tomography in patients with myeloperoxidase anti-neutrophil cytoplasmic antibody-related nephritis: A retrospective single-center study. BMC Pulm. Med..

[398] Bahudhanapati H., Tan J., Dutta J.A., Strock S.B., Sembrat J., Àlvarez D., Rojas M., Jäger B., Prasse A., Zhang Y. (2019). MicroRNA-144-3p targets relaxin/insulin-like family peptide receptor 1 (RXFP1) expression in lung fibroblasts from patients with idiopathic pulmonary fibrosis. J. Biol. Chem..

[399] Arai N., Nakajima M., Matsuyama M., Matsumura S., Yazaki K., Sakai C., Nonaka M., Yanai H., Numata T., Yamamoto Y. (2023). Variations in S100A8/A12 Gene Expression Are Associated with the Efficacy of Nintedanib and Acute Exacerbation Development in Idiopathic Pulmonary Fibrosis Patients. Am. J. Respir. Cell Mol. Biol..

[400] Hamelet J., Maurin N., Fulchiron R., Delabar J.M., Janel N. (2007). Mice lacking cystathionine beta synthase have lung fibrosis and air space enlargement. Exp. Mol. Pathol..

[401] Huang G., Liang J., Huang K., Liu X., Taghavifar F., Yao C., Parimon T., Liu N., Dai K., Aziz A. (2023). Basal Cell-derived WNT7A Promotes Fibrogenesis at the Fibrotic Niche in Idiopathic Pulmonary Fibrosis. Am. J. Respir. Cell Mol. Biol..

[402] Cherubini E., Mariotta S., Scozzi D., Mancini R., Osman G., D’Ascanio M., Bruno P., Cardillo G., Ricci A. (2017). BDNF/TrkB axis activation promotes epithelial-mesenchymal transition in idiopathic pulmonary fibrosis. J. Transl. Med..

[403] Gui X., Qiu X., Tian Y., Xie M., Li H., Gao Y., Zhuang Y., Cao M., Ding H., Ding J. (2019). Prognostic value of IFN-γ, sCD163, CCL2 and CXCL10 involved in acute exacerbation of idiopathic pulmonary fibrosis. Int. Immunopharmacol..

[404] Lee J.U., Cheong H.S., Shim E.Y., Bae D.J., Chang H.S., Uh S.T., Kim Y.H., Park J.S., Lee B., Shin H.D. (2017). Gene profile of fibroblasts identify relation of CCL8 with idiopathic pulmonary fibrosis. Respir. Res..

[405] Bournazos S., Bournazou I., Murchison J.T., Wallace W.A., McFarlane P., Hirani N., Simpson A.J., Dransfield I., Hart S.P. (2011). Copy number variation of FCGR3B is associated with susceptibility to idiopathic pulmonary fibrosis. Respiration.

[406] Tanaka K., Enomoto N., Hozumi H., Isayama T., Naoi H., Aono Y., Katsumata M., Yasui H., Karayama M., Suzuki Y. (2021). Serum S100A8 and S100A9 as prognostic biomarkers in acute exacerbation of idiopathic pulmonary fibrosis. Respir. Investig..

[407] Barlo N.P., van Moorsel C.H., Korthagen N.M., Heron M., Rijkers G.T., Ruven H.J., van den Bosch J.M., Grutters J.C. (2011). Genetic variability in the IL1RN gene and the balance between interleukin (IL)-1 receptor agonist and IL-1β in idiopathic pulmonary fibrosis. Clin. Exp. Immunol..

[408] Russo R.C., Guabiraba R., Garcia C.C., Barcelos L.S., Roffê E., Souza A.L., Amaral F.A., Cisalpino D., Cassali G.D., Doni A. (2009). Role of the chemokine receptor CXCR2 in bleomycin-induced pulmonary inflammation and fibrosis. Am. J. Respir. Cell Mol. Biol..

[409] Gao F., Zhang Y., Yang Z., Wang M., Zhou Z., Zhang W., Ren Y., Han X., Wei M., Sun Z. (2020). Arctigenin Suppressed Epithelial-Mesenchymal Transition Through Wnt3a/β-Catenin Pathway in PQ-Induced Pulmonary Fibrosis. Front Pharmacol..

[410] Chen H., Chen H., Liang J., Gu X., Zhou J., Xie C., Lv X., Wang R., Li Q., Mao Z. (2020). TGF-β1/IL-11/MEK/ERK signaling mediates senescence-associated pulmonary fibrosis in a stress-induced premature senescence model of Bmi-1 deficiency. Exp. Mol. Med..

[411] Ohkouchi S., Kanehira M., Saigusa D., Ono M., Tazawa R., Terunuma H., Hirano T., Numakura T., Notsuda H., Inoue C. (2022). Metabolic and Epigenetic Regulation of SMAD7 by STC1 Ameliorates Lung Fibrosis. Am. J. Respir. Cell Mol. Biol..

[412] Klay D., Platenburg M.G.J.P., van Rijswijk R.H.N.A.J., Grutters J.C., van Moorsel C.H.M. (2020). ABCA3 mutations in adult pulmonary fibrosis patients: A case series and review of literature. Curr. Opin. Pulm. Med..

[413] Heinzelmann K., Lehmann M., Gerckens M., Noskovičová N., Frankenberger M., Lindner M., Hatz R., Behr J., Hilgendorff A., Königshoff M. (2018). Cell-surface phenotyping identifies CD36 and CD97 as novel markers of fibroblast quiescence in lung fibrosis. Am. J. Physiol. Lung Cell. Mol. Physiol..

[414] Lv X., Liu S., Liu C., Li Y., Zhang T., Qi J., Li K., Hua F., Cui B., Zhang X. (2023). TRIB3 promotes pulmonary fibrosis through inhibiting SLUG degradation by physically interacting with MDM2. Acta Pharm. Sin. B.

[415] Schamberger A.C., Schiller H.B., Fernandez I.E., Sterclova M., Heinzelmann K., Hennen E., Hatz R., Behr J., Vašáková M., Mann M. (2016). lutathione peroxidase 3 localizes to the epithelial lining fluid and the extracellular matrix in interstitial lung disease. Sci. Rep..

[416] Li L., Zhang S., Wei L., Wang Z., Ma W., Liu F., Qian Y. (2018). FGF2 and FGFR2 in patients with idiopathic pulmonary fibrosis and lung cancer. Oncol. Lett..

[417] Shin H., Park S., Hong J., Baek A.R., Lee J., Kim D.J., Jang A.S., Chin S.S., Jeong S.H., Park S.W. (2023). Overexpression of fatty acid synthase attenuates bleomycin induced lung fibrosis by restoring mitochondrial dysfunction in mice. Sci. Rep..

[418] Effendi W.I., Nagano T. (2021). The Hedgehog Signaling Pathway in Idiopathic Pulmonary Fibrosis: Resurrection Time. Int. J. Mol. Sci..

[419] Lu Y., Tang K., Wang S., Tian Z., Fan Y., Li B., Wang M., Zhao J., Xie J. (2023). Dach1 deficiency drives alveolar epithelium apoptosis in pulmonary fibrosis via modulating C-Jun/Bim activity. Transl. Res..

[420] Joannes A., Brayer S., Besnard V., Marchal-Sommé J., Jaillet M., Mordant P., Mal H., Borie R., Crestani B., Mailleux A.A. (2016). FGF9 and FGF18 in idiopathic pulmonary fibrosis promote survival and migration and inhibit myofibroblast differentiation of human lung fibroblasts in vitro. Am. J. Physiol. Lung Cell. Mol. Physiol..

[421] Liu Y., Tang A., Liu M., Xu C., Cao F., Yang C. (2023). Tuberostemonine may enhance the function of the SLC7A11/glutamate antiporter to restrain the ferroptosis to alleviate pulmonary fibrosis. J. Ethnopharmacol..

[422] Duan J.X., Guan X.X., Yang H.H., Mei W.X., Chen P., Tao J.H., Li Q., Zhou Y. (2021). Vasoactive intestinal peptide attenuates bleomycin-induced murine pulmonary fibrosis by inhibiting epithelial-mesenchymal transition: Restoring autophagy in alveolar epithelial cells. Int. Immunopharmacol..

[423] Barnes J.W., Duncan D., Helton S., Hutcheson S., Kurundkar D., Logsdon N.J., Locy M., Garth J., Denson R., Farver C. (2019). Role of fibroblast growth factor 23 and klotho cross talk in idiopathic pulmonary fibrosis. Am. J. Physiol. Lung Cell. Mol. Physiol..

[424] Yanagihara T., Tsubouchi K., Zhou Q., Chong M., Otsubo K., Isshiki T., Schupp J.C., Sato S., Scallan C., Upagupta C. (2023). Vascular-Parenchymal Cross-Talk Promotes Lung Fibrosis through BMPR2 Signaling. Am. J. Respir. Crit. Care Med..

[425] Wygrecka M., Alexopoulos I., Potaczek D.P., Schaefer L. (2023). Diverse functions of apolipoprotein A-I in lung fibrosis. Am. J. Physiol. Cell. Physiol..

[426] Tian Y., Lv J., Su Z., Wu T., Li X., Hu X., Zhang J., Wu L. (2021). LRRK2 plays essential roles in maintaining lung homeostasis and preventing the development of pulmonary fibrosis. Proc. Natl. Acad. Sci. USA.

[427] McElroy A.N., Invernizzi R., Laskowska J.W., O’Neill A., Doroudian M., Moghoofei M., Mostafaei S., Li F., Przybylski A.A., O’Dwyer D.N. (2022). Candidate Role for Toll-like Receptor 3 L412F Polymorphism and Infection in Acute Exacerbation of Idiopathic Pulmonary Fibrosis. Am. J. Respir. Crit. Care Med..

[428] Sun B., Xu S., Yan Y., Li Y., Li H., Zheng G., Dong T., Bai J. (2020). miR-205 Suppresses Pulmonary Fibrosis by Targeting GATA3 Through Inhibition of Endoplasmic Reticulum Stress. Curr. Pharm. Biotechnol..

[429] Munguía-Reyes A., Balderas-Martínez Y.I., Becerril C., Checa M., Ramírez R., Ortiz B., Meléndez-Zajgla J., Pardo A., Selman M. (2018). R-Spondin-2 Is Upregulated in Idiopathic Pulmonary Fibrosis and Affects Fibroblast Behavior. Am. J. Respir. Cell Mol. Biol..

[430] Brody S.L., Gunsten S.P., Luehmann H.P., Sultan D.H., Hoelscher M., Heo G.S., Pan J., Koenitzer J.R., Lee E.C., Huang T. (2021). Chemokine Receptor 2-targeted Molecular Imaging in Pulmonary Fibrosis. A Clinical Trial. Am. J. Respir. Crit. Care Med..

[431] Liu H., Gu C., Liu M., Liu G., Wang Y. (2020). NEK7 mediated assembly and activation of NLRP3 inflammasome downstream of potassium efflux in ventilator-induced lung injury. Biochem. Pharmacol..

[432] Huan C., Yang T., Liang J., Xie T., Cheng L., Liu N., Kurkciyan A., Monterrosa Mena J., Wang C., Dai H. (2015). Methylation-mediated BMPER expression in fibroblast activation in vitro and lung fibrosis in mice in vivo. Sci. Rep..

[433] Yin D., Qiu J., Hu S., Cheng L., Li H., Cheng X., Wang S., Lu J. (2022). CAV1 is a prognostic predictor for patients with idiopathic pulmonary fibrosis and lung cancer. J. Biosci..

[434] Zorzetto M., Ferrarotti I., Trisolini R., Lazzari Agli L., Scabini R., Novo M., De Silvestri A., Patelli M., Martinetti M., Cuccia M. (2003). Complement receptor 1 gene polymorphisms are associated with idiopathic pulmonary fibrosis. Am. J. Respir. Crit. Care Med..

[435] Kijiyama N., Ueno H., Sugimoto I., Sasaguri Y., Yatera K., Kido M., Gabazza E.C., Suzuki K., Hashimoto E., Takeya H. (2006). Intratracheal gene transfer of tissue factor pathway inhibitor attenuates pulmonary fibrosis. Biochem. Biophys. Res. Commun..

[436] Zhu L., Chen Y., Chen M., Wang W. (2021). Mechanism of miR-204-5p in exosomes derived from bronchoalveolar lavage fluid on the progression of pulmonary fibrosis via AP1S2. Ann. Transl. Med..

[437] Wijk S.C., Prabhala P., Löfdahl A., Nybom A., Lang S., Brunnström H., Bjermer L., Westergren-Thorsson G., Magnusson M. (2022). Ciliated (FOXJ1+) Cells Display Reduced Ferritin Light Chain in the Airways of Idiopathic Pulmonary Fibrosis Patients. Cells.

[438] Wang D., Deng B., Cheng L., Li J., Zhang J., Zhang X., Guo X., Yan T., Yue X., An Y. (2023). A novel and low-toxic peptide DR3penA alleviates pulmonary fibrosis by regulating the MAPK/miR-23b-5p/AQP5 signaling axis. Acta Pharm. Sin. B.

[439] Ballester B., Milara J., Montero P., Cortijo J. (2021). MUC16 Is Overexpressed in Idiopathic Pulmonary Fibrosis and Induces Fibrotic Responses Mediated by Transforming Growth Factor-β1 Canonical Pathway. Int. J. Mol. Sci..

[440] Milara J., Ballester B., Safont M.J., Artigues E., Escrivá J., Morcillo E., Cortijo J. (2021). MUC4 is overexpressed in idiopathic pulmonary fibrosis and collaborates with transforming growth factor β inducing fibrotic responses. Mucosal Immunol..

[441] Amsellem V., Abid S., Poupel L., Parpaleix A., Rodero M., Gary-Bobo G., Latiri M., Dubois-Rande J.L., Lipskaia L., Combadiere C. (2017). Roles for the CX3CL1/CX3CR1 and CCL2/CCR2 Chemokine Systems in Hypoxic Pulmonary Hypertension. Am. J. Respir. Cell Mol. Biol..

[442] Tzouvelekis A., Herazo-Maya J.D., Ryu C., Chu J.H., Zhang Y., Gibson K.F., Adonteng-Boateng P.K., Li Q., Pan H., Cherry B. (2018). S100A12 as a marker of worse cardiac output and mortality in pulmonary hypertension. Respirology.

[443] Kurrek M.M., Winkler M., Robinson D.R., Zapol W.M. (1995). Platelet factor 4 injection produces acute pulmonary hypertension in the awake lamb. Anesthesiology.

[444] Klinke A., Berghausen E., Friedrichs K., Molz S., Lau D., Remane L., Berlin M., Kaltwasser C., Adam M., Mehrkens D. (2018). Myeloperoxidase aggravates pulmonary arterial hypertension by activation of vascular Rho-kinase. JCI Insight.

[445] Chakraborty A., Nathan A., Orcholski M., Agarwal S., Shamskhou E.A., Auer N., Mitra A., Guardado E.S., Swaminathan G., Condon D.F. (2023). Wnt7a deficit is associated with dysfunctional angiogenesis in pulmonary arterial hypertension. Eur. Respir. J..

[446] Zhang F., Yang M., Xiao T., Hua Y., Chen Y., Xu S., Ni C. (2020). SLC6A4 gene L/S polymorphism and susceptibility to pulmonary arterial hypertension: A meta-analysis. J. Int. Med. Res..

[447] Schäfer K., Tello K., Pak O., Richter M., Gierhardt M., Kwapiszewska G., Veith C., Fink L., Gall H., Hecker M. (2023). Decreased plasma levels of the brain-derived neurotrophic factor correlate with right heart congestion in pulmonary arterial hypertension. ERJ Open Res..

[448] Hong C., Lu J., Chen R., Liu H., Chen H., Wu X., Guo W., Huang Z., Liao H. (2022). CXCL10 levels in diagnosis and improved hemodynamics in patients with chronic thromboembolic pulmonary hypertension undergoing balloon pulmonary angioplasty. Pulm. Circ..

[449] Li K., Li Y., Yu Y., Ding J., Huang H., Chu C., Hu L., Yu Y., Cao Y., Xu P. (2021). Bmi-1 alleviates adventitial fibroblast senescence by eliminating ROS in pulmonary hypertension. BMC Pulm. Med..

[450] Dempsie Y., MacRitchie N.A., White K., Morecroft I., Wright A.F., Nilsen M., Loughlin L., Mair K.M., MacLean M.R. (2013). Dexfenfluramine and the oestrogen-metabolizing enzyme CYP1B1 in the development of pulmonary arterial hypertension. Cardiovasc. Res..

[451] Ota C., Kimura M., Kure S. (2016). ABCA3 mutations led to pulmonary fibrosis and emphysema with pulmonary hypertension in an 8-year-old girl. Pediatr. Pulmonol..

[452] Cao X., Fang X., Guo M., Li X., He Y., Xie M., Xu Y., Liu X. (2021). TRB3 mediates vascular remodeling by activating the MAPK signaling pathway in hypoxic pulmonary hypertension. Respir. Res..

[453] Ye P., Jiang X.M., Qian W.C., Zhang J. (2023). Inhibition of PCSK9 Improves the Development of Pulmonary Arterial Hypertension Via Down-Regulating Notch3 Expression. Cardiovasc. Drugs Ther..

[454] Yoshida T., Nagaoka T., Nagata Y., Suzuki Y., Tsutsumi T., Kuriyama S., Watanabe J., Togo S., Takahashi F., Matsushita M. (2022). Periostin-related progression of different types of experimental pulmonary hypertension: A role for M2 macrophage and FGF-2 signalling. Respirology.

[455] Jiang C.Y., Wu L.W., Liu Y.W., Feng B., Ye L.C., Huang X., He Y.Y., Shen Y., Zhu Y.F., Zhou X.L. (2023). Identification of ACKR4 as an immune checkpoint in pulmonary arterial hypertension. Front. Immunol..

[456] Alsabeelah N., Kumar V. (2022). Protective Effect of Triclosan in Monocrotaline-Induced Pulmonary Arterial Hypertension: FASN Inhibition a Novel Approach. J. Pharm. Bioallied Sci..

[457] Leuchte H.H., Prechtl C., Callegari J., Meis T., Haziraj S., Bevec D., Behr J. (2015). Augmentation of the effects of vasoactive intestinal peptide aerosol on pulmonary hypertension via coapplication of a neutral endopeptidase 24.11 inhibitor. Am. J. Physiol. Lung Cell. Mol. Physiol..

[458] Batlahally S., Franklin A., Damianos A., Huang J., Chen P., Sharma M., Duara J., Keerthy D., Zambrano R., Shehadeh L.A. (2020). Soluble Klotho, a biomarker and therapeutic strategy to reduce bronchopulmonary dysplasia and pulmonary hypertension in preterm infants. Sci. Rep..

[459] Spiekerkoetter E., Tian X., Cai J., Hopper R.K., Sudheendra D., Li C.G., El-Bizri N., Sawada H., Haghighat R., Chan R. (2013). FK506 activates BMPR2, rescues endothelial dysfunction, and reverses pulmonary hypertension. J. Clin. Investig..

[460] Bhagwani A.R., Ali M., Piper B., Liu M., Hudson J., Kelly N., Bogamuwa S., Yang H., Londino J.D., Bednash J.S. (2023). A p53-TLR3 axis ameliorates pulmonary hypertension by inducing BMPR2 via IRF3. iScience.

[461] Abid S., Marcos E., Parpaleix A., Amsellem V., Breau M., Houssaini A., Vienney N., Lefevre M., Derumeaux G., Evans S. (2019). CCR2/CCR5-mediated macrophage-smooth muscle cell crosstalk in pulmonary hypertension. Eur. Respir. J..

[462] Yeh F.C., Chen C.N., Xie C.Y., Baxan N., Zhao L., Ashek A., Sabrin F., Lawrie A., Wilkins M., Zhao L. (2023). TLR7/8 activation induces autoimmune vasculopathy and causes severe pulmonary arterial hypertension. Eur. Respir. J..

[463] Liu B., Peng Y., Yi D., Machireddy N., Dong D., Ramirez K., Dai J., Vanderpool R., Zhu M.M., Dai Z. (2022). Endothelial PHD2 deficiency induces nitrative stress via suppression of caveolin-1 in pulmonary hypertension. Eur. Respir. J..

[464] Nakwan N., Kunhapan P., Chaiyasung T., Satproedprai N., Singkhamanan K., Mahasirimongkol S., Charalsawadi C. (2023). Genome-wide association study identifies WWC2 as a possible locus associated with persistent pulmonary hypertension of the newborn in the Thai population. Transl. Pediatr..

[465] White T.A., Witt T.A., Pan S., Mueske C.S., Kleppe L.S., Holroyd E.W., Champion H.C., Simari R.D. (2010). Tissue factor pathway inhibitor overexpression inhibits hypoxia-induced pulmonary hypertension. Am. J. Respir. Cell Mol. Biol..

[466] Wang N., Hua J., Fu Y., An J., Chen X., Wang C., Zheng Y., Wang F., Ji Y., Li Q. (2023). Updated perspective of EPAS1 and the role in pulmonary hypertension. Front. Cell Dev. Biol..

[467] Dai H.L., Wang D., Guang X.F., Zhang W.H. (2022). Pulmonary Hypertension in a Patient with Kartagener’s Syndrome and a Novel Homozygous Nonsense Mutation in CCDC40 Gene: A Case Report. Front. Med..

[468] Wu M., Jin M., Cao X., Qian K., Zhao L. (2022). RNA editing enzyme adenosine deaminases acting on RNA 1 deficiency increases the sensitivity of non-small cell lung cancer cells to anlotinib by regulating CX3CR1-fractalkine expression. Drug Dev. Res..

[469] Li H., Zhong R., He C., Tang C., Cui H., Li R., Liu Y., Lan S., Cheng Y. (2022). Colony-stimulating factor CSF2 mediates the phenotypic plasticity of small-cell lung cancer by regulating the p-STAT3/MYC pathway. Oncol. Rep..

[470] Liu J., Yang H., Yin D., Jia Y., Li S., Liu Y. (2022). Expression and prognostic analysis of CLDN18 and Claudin18.2 in lung adenocarcinoma. Pathol. Res. Pract..

[471] Shang R., Chen J., Gao Y., Chen J., Han G. (2023). TRIM58 Interacts with ZEB1 to Suppress NSCLC Tumor Malignancy by Promoting ZEB1 Protein Degradation via UPP. Dis. Markers..

[472] Struyf S., Burdick M.D., Peeters E., Van den Broeck K., Dillen C., Proost P., Van Damme J., Strieter R.M. (2007). Platelet factor-4 variant chemokine CXCL4L1 inhibits melanoma and lung carcinoma growth and metastasis by preventing angiogenesis. Cancer Res..

[473] Kim M.J., Kim J.Y., Shin J.H., Kang Y., Lee J.S., Son J., Jeong S.K., Kim D., Kim D.H., Chun E. (2023). FFAR2 antagonizes TLR2- and TLR3-induced lung cancer progression via the inhibition of AMPK-TAK1 signaling axis for the activation of NF-κB. Cell Biosci..

[474] Rymaszewski A.L., Tate E., Yimbesalu J.P., Gelman A.E., Jarzembowski J.A., Zhang H., Pritchard K.A., Vikis H.G. (2014). The role of neutrophil myeloperoxidase in models of lung tumor development. Cancers.

[475] Choi E.S., Faruque H.A., Kim J.H., Kim K.J., Choi J.E., Kim B.A., Kim B., Kim Y.J., Woo M.H., Park J.Y. (2021). CD5L as an Extracellular Vesicle-Derived Biomarker for Liquid Biopsy of Lung Cancer. Diagnostics.

[476] Dasgupta S., Jang J.S., Shao C., Mukhopadhyay N.D., Sokhi U.K., Das S.K., Brait M., Talbot C., Yung R.C., Begum S. (2013). SH3GL2 is frequently deleted in non-small cell lung cancer and downregulates tumor growth by modulating EGFR signaling. J. Mol. Med..

[477] Xing S., Zeng T., Xue N., He Y., Lai Y.Z., Li H.L., Huang Q., Chen S.L., Liu W.L. (2019). Development and Validation of Tumor-educated Blood Platelets Integrin Alpha 2b (ITGA2B) RNA for Diagnosis and Prognosis of Non-small-cell Lung Cancer through RNA-seq. Int. J. Biol. Sci..

[478] Wong S.W., McCarroll J., Hsu K., Geczy C.L., Tedla N. (2022). Intranasal Delivery of Recombinant S100A8 Protein Delays Lung Cancer Growth by Remodeling the Lung Immune Microenvironment. Front. Immunol..

[479] Sun L., Zhang Q., Li Y., Tang N., Qiu X. (2015). CCL21/CCR7 up-regulate vascular endothelial growth factor-D expression via ERK pathway in human non-small cell lung cancer cells. Int. J. Clin. Exp. Pathol..

[480] Kim H.J., Park J., Lee S.K., Kim K.R., Park K.K., Chung W.Y. (2015). Loss of RUNX3 expression promotes cancer-associated bone destruction by regulating CCL5, CCL19 and CXCL11 in non-small cell lung cancer. J. Pathol..

[481] Li Y., Lian H., Jia Q., Wan Y. (2015). Proteome screening of pleural effusions identifies IL1A as a diagnostic biomarker for non-small cell lung cancer. Biochem. Biophys. Res. Commun..

[482] Bikkavilli R.K., Avasarala S., Van Scoyk M., Arcaroli J., Brzezinski C., Zhang W., Edwards M.G., Rathinam M.K., Zhou T., Tauler J. (2015). Wnt7a is a novel inducer of β-catenin-independent tumor-suppressive cellular senescence in lung cancer. Oncogene.

[483] Fujita T., Yamaji Y., Sato M., Murao K., Takahara J. (1994). Gene expression of somatostatin receptor subtypes, SSTR1 and SSTR2, in human lung cancer cell lines. Life Sci..

[484] Jaskiewicz L., Hejne K., Szostak B., Osowiecka K., Skowronski M.T., Lepiarczyk E., Doboszynska A., Majewska M., Kordowitzki P., Skowronska A. (2022). Expression Profiles of AQP3 and AQP4 in Lung Adenocarcinoma Samples Generated via Bronchoscopic Biopsies. J. Clin. Med..

[485] Nashed M., Chisholm J.W., Igal R.A. (2012). Stearoyl-CoA desaturase activity modulates the activation of epidermal growth factor receptor in human lung cancer cells. Exp. Biol. Med.

[486] Tu Y., Yao S., Chen Q., Li W., Song Y., Zhang P. (2022). 5-Hydroxytryptamine activates a 5-HT/c-Myc/SLC6A4 signaling loop in non-small cell lung cancer. Biochim. Biophys. Acta Gen. Subj..

[487] Kimura S., Harada T., Ijichi K., Tanaka K., Liu R., Shibahara D., Kawano Y., Otsubo K., Yoneshima Y., Iwama E. (2018). Expression of brain-derived neurotrophic factor and its receptor TrkB is associated with poor prognosis and a malignant phenotype in small cell lung cancer. Lung Cancer.

[488] Wang C.L., Ho A.S., Chang C.C., Sie Z.L., Peng C.L., Chang J., Cheng C.C. (2023). Radiotherapy enhances CXCR3highCD8+ T cell activation through inducing IFNγ-mediated CXCL10 and ICAM-1 expression in lung cancer cells. Cancer Immunol. Immunother..

[489] Yuan D.F., Wang H.R., Wang Z.F., Liang G.H., Xing W.Q., Qin J.J. (2021). CircRNA CircZMYM4 inhibits the growth and metastasis of lung adenocarcinoma via the miR-587/ODAM pathway. Biochem. Biophys. Res. Commun..

[490] Ulybina Y.M., Kuligina E.S., Mitiushkina N.V., Rozanov M.E., Ivantsov A.O., Ponomariova D.N., Togo A.V., Levchenko E.V., Shutkin V.A., Brenister S.I. (2009). Coding polymorphisms in Casp5, Casp8 and DR4 genes may play a role in predisposition to lung cancer. Cancer Lett..

[491] Drosslerova M., Sterclova M., Taskova A., Hytych V., Richterova E., Bruzova M., Spunda M., Komarc M., Koziar Vasakova M. (2023). CCL2, CCL8, CXCL12 chemokines in resectable non-small cell lung cancer (NSCLC). Biomed. Pap. Med. Fac. Univ. Palacky Olomouc Czech. Repub..

[492] Wang Y., Ha M., Li M., Zhang L., Chen Y. (2022). Histone deacetylase 6-mediated downregulation of TMEM100 expedites the development and progression of non-small cell lung cancer. Hum. Cell.

[493] Biswas A.K., Han S., Tai Y., Ma W., Coker C., Quinn S.A., Shakri A.R., Zhong T.J., Scholze H., Lagos G.G. (2022). Targeting S100A9-ALDH1A1-Retinoic Acid Signaling to Suppress Brain Relapse in EGFR-Mutant Lung Cancer. Cancer Discov..

[494] Yin J., Wang C., Vogel U., Ma Y., Zhang Y., Wang H., Sun Z., Du S. (2023). Common variants of pro-inflammatory gene IL1B and interactions with PPP1R13L and POLR1G in relation to lung cancer among Northeast Chinese. Sci. Rep..

[495] Yang F., Zhang S., Meng Q., Zhou F., Pan B., Liu F., Yu Y. (2021). CXCR1 correlates to poor outcomes of EGFR-TKI against advanced non-small cell lung cancer by activating chemokine and JAK/STAT pathway. Pulm. Pharmacol. Ther..

[496] Wu X., Xia J., Wang Z., Xu Z., Liu K., Fu X., Deng H. (2022). Feiyanning downregulating CXCLs/CXCR2 axis to suppress TANs infiltration in the prevention of lung cancer metastasis. J. Ethnopharmacol..

[497] Song J.W., Zhu J., Wu X.X., Tu T., Huang J.Q., Chen G.Z., Liang L.Y., Zhou C.H., Xu X., Gong L.Y. (2021). GOLPH3/CKAP4 promotes metastasis and tumorigenicity by enhancing the secretion of exosomal WNT3A in non-small-cell lung cancer. Cell Death Dis..

[498] Shen H.T., Chien P.J., Chen S.H., Sheu G.T., Jan M.S., Wang B.Y., Chang W.W. (2020). BMI1-Mediated Pemetrexed Resistance in Non-Small Cell Lung Cancer Cells Is Associated with Increased SP1 Activation and Cancer Stemness. Cancers.

[499] Xie S., Tu Z., Xiong J., Kang G., Zhao L., Hu W., Tan H., Tembo K.M., Ding Q., Deng X. (2017). CXCR4 promotes cisplatin-resistance of non-small cell lung cancer in a CYP1B1-dependent manner. Oncol. Rep..

[500] Jang H., Jun Y., Kim S., Kim E., Jung Y., Park B.J., Lee J., Kim J., Lee S., Kim J. (2021). FCN3 functions as a tumor suppressor of lung adenocarcinoma through induction of endoplasmic reticulum stress. Cell Death Dis..

[501] Zou S., Ye J., Hu S., Wei Y., Xu J. (2022). Mutations in the TTN Gene are a Prognostic Factor for Patients with Lung Squamous Cell Carcinomas. Int. J. Gen. Med..

[502] Si J., Ma Y., Bi J.W., Xiong Y., Lv C., Li S., Wu N., Yang Y. (2019). Shisa3 brakes resistance to EGFR-TKIs in lung adenocarcinoma by suppressing cancer stem cell properties. J. Exp. Clin. Cancer Res..

[503] Tian B., Han X., Li G., Jiang H., Qi J., Li J., Tian Y., Wang C. (2020). A Long Intergenic Non-coding RNA, LINC01426, Promotes Cancer Progression via AZGP1 and Predicts Poor Prognosis in Patients with LUAD. Mol. Ther. Methods Clin. Dev..

[504] Overbeck T.R., Arnemann J., Waldmann-Beushausen R., Trümper L., Schöndube F.A., Reuter-Jessen K., Danner B.C. (2017). ABCA3 Phenotype in Non-Small Cell Lung Cancer Indicates Poor Outcome. Oncology.

[505] Sun Q., Zhang W., Wang L., Guo F., Song D., Zhang Q., Zhang D., Fan Y., Wang J. (2018). Hypermethylated CD36 gene affected the progression of lung cancer. Gene.

[506] Wei F., Ge Y., Li W., Wang X., Chen B. (2020). Role of endothelin receptor type B (EDNRB) in lung adenocarcinoma. Thorac. Cancer.

[507] Ma W., Liang J., Mo J., Zhang S., Hu N., Tian D., Chen Z. (2021). Butyrophilin-like 9 expression is associated with outcome in lung adenocarcinoma. BMC Cancer.

[508] Sato A., Yamada N., Ogawa Y., Ikegami M. (2013). CCAAT/enhancer-binding protein-α suppresses lung tumor development in mice through the p38α MAP kinase pathway. PLoS ONE.

[509] Yu J.J., Zhou D.D., Yang X.X., Cui B., Tan F.W., Wang J., Li K., Shang S., Zhang C., Lv X.X. (2020). TRIB3-EGFR interaction promotes lung cancer progression and defines a therapeutic target. Nat. Commun..

[510] Ye X., Xie G., Liu Z., Tang J., Cui M., Wang C., Guo C., Tang J. (2020). TNNC1 Reduced Gemcitabine Sensitivity of Nonsmall-Cell Lung Cancer by Increasing Autophagy. Med. Sci. Monit..

[511] Gao X., Yi L., Jiang C., Li S., Wang X., Yang B., Li W., Che N., Wang J., Zhang H. (2023). PCSK9 regulates the efficacy of immune checkpoint therapy in lung cancer. Front. Immunol..

[512] Lin J., Wu C., Ma D., Hu Q. (2021). Identification of P2RY13 as an immune-related prognostic biomarker in lung adenocarcinoma: A public database-based retrospective study. PeerJ.

[513] Salomonsson A., Jönsson M., Isaksson S., Karlsson A., Jönsson P., Gaber A., Bendahl P.O., Johansson L., Brunnström H., Jirström K. (2013). Histological specificity of alterations and expression of KIT and KITLG in non-small cell lung carcinoma. Genes Chromosomes Cancer.

[514] Wang Y., Zhang L., Yang J., Li B., Wang J. (2018). CDH13 promoter methylation regulates cisplatin resistance of non-small cell lung cancer cells. Oncol. Lett..

[515] Liu X., Chen Z., Wu Y., Gu F., Yan D., Yang L., Ma Q., Fu C. (2022). Circ_0078767 Inhibits the Progression of Non-Small-Cell Lung Cancer by Regulating the GPX3 Expression by Adsorbing miR-665. Int. J. Genom..

[516] Qiao R., Di F., Wang J., Wei Y., Xu T., Dai L., Gu W., Han B., Yang R. (2023). Identification of FUT7 hypomethylation as the blood biomarker in the prediction of early-stage lung cancer. J. Genet. Genom..

[517] Wang Q., Tian N., Zhang W., Lin Z., Shi F., Kong Y., Ren Y., Lyu J., Qin H., Liu H. (2022). Fatty Acid Synthase Mutations Predict Favorable Immune Checkpoint Inhibitor Outcome and Response in Melanoma and Non-Small Cell Lung Cancer Patients. Cancers.

[518] Zhang S., Wang Y., Dai S.D., Wang E.H. (2011). Down-regulation of NKD1 increases the invasive potential of non-small-cell lung cancer and correlates with a poor prognosis. BMC Cancer.

[519] Xie F., Li Y., Liang B. (2022). The Expression and Survival Significance of FOXD1 in Lung Squamous Cell Carcinoma: A Meta-Analysis, Immunohistochemistry Validation, and Bioinformatics Analysis. Biomed. Res. Int..

[520] Guo W., Li K., Sun B., Xu D., Tong L., Yin H., Liao Y., Song H., Wang T., Jing B. (2021). Dysregulated Glutamate Transporter SLC1A1 Propels Cystine Uptake via Xc- for Glutathione Synthesis in Lung Cancer. Cancer Res..

[521] Raz G., Allen K.E., Kingsley C., Cherni I., Arora S., Watanabe A., Lorenzo C.D., Edwards V.D.K., Sridhar S., Hostetter G. (2012). Hedgehog signaling pathway molecules and ALDH1A1 expression in early-stage non-small cell lung cancer. Lung Cancer.

[522] Yu J., Jiang P., Zhao K., Chen Z., Zuo T., Chen B. (2021). Role of DACH1 on Proliferation, Invasion, and Apoptosis in Human Lung Adenocarcinoma Cells. Curr. Mol. Med..

[523] Ishioka K., Yasuda H., Hamamoto J., Terai H., Emoto K., Kim T.J., Hirose S., Kamatani T., Mimaki S., Arai D. (2021). Upregulation of FGF9 in Lung Adenocarcinoma Transdifferentiation to Small Cell Lung Cancer. Cancer Res..

[524] Peng Y., Ouyang L., Zhou Y., Lai W., Chen Y., Wang Z., Yan B., Zhang Z., Zhou Y., Peng X. (2023). AhR Promotes the Development of Non-small cell lung cancer by Inducing SLC7A11-dependent Antioxidant Function. J. Cancer.

[525] Bian T., Zhang W., Wang F., Chu X., Pan X., Ruan J., Yu S., Liu L., Sun H., Qiu H. (2023). Identification of CLIC5 as a Prognostic Biomarker and Correlated Immunomodulator for Lung Adenocarcinoma. Comb. Chem. High Throughput Screen..

[526] Niu H., Qu A., Guan C. (2021). Suppression of MGAT3 expression and the epithelial-mesenchymal transition of lung cancer cells by miR-188-5p. Biomed. J..

[527] Wang L., Hou J., Wang J., Zhu Z., Zhang W., Zhang X., Shen H., Wang X. (2020). Regulatory roles of HSPA6 in Actinidia chinensis Planch. root extract (acRoots)-inhibited lung cancer proliferation. Clin. Transl. Med..

[528] Liang G., Meng W., Huang X., Zhu W., Yin C., Wang C., Fassan M., Yu Y., Kudo M., Xiao S. (2020). miR-196b-5p-mediated downregulation of TSPAN12 and GATA6 promotes tumor progression in non-small cell lung cancer. Proc. Natl. Acad. Sci. USA.

[529] Zhang Y., Qian K., Liu X., Zhao X., Zhao T., Lu G. (2022). Exosomal mir-625-3p derived from hypoxic lung cancer cells facilitates metastasis by targeting SCAI. Mol. Biol. Rep..

[530] Szilasi M., Buglyo A., Treszl A., Kiss L., Schally A.V., Halmos G. (2011). Gene expression of vasoactive intestinal peptide receptors in human lung cancer. Int. J. Oncol..

[531] Lin Z., Liu Z., Tan X., Li C. (2021). SH3GL3 functions as a potent tumor suppressor in lung cancer in a SH3 domain dependent manner. Biochem. Biophys. Res. Commun..

[532] Liu H., Huang J., Peng J., Wu X., Zhang Y., Zhu W., Guo L. (2015). Upregulation of the inwardly rectifying potassium channel Kir2.1 (KCNJ2) modulates multidrug resistance of small-cell lung cancer under the regulation of miR-7 and the Ras/MAPK pathway. Mol. Cancer.

[533] Chen B., Zhao H., Li M., She Q., Liu W., Zhang J., Zhao W., Huang S., Wu J. (2022). SHANK1 facilitates non-small cell lung cancer processes through modulating the ubiquitination of Klotho by interacting with MDM2. Cell Death Dis..

[534] Hou L., Li Y., Wang Y., Xu D., Cui H., Xu X., Cong Y., Yu C. (2018). UBE2D1 RNA Expression Was an Independent Unfavorable Prognostic Indicator in Lung Adenocarcinoma, but Not in Lung Squamous Cell Carcinoma. Dis. Markers.

[535] Pan S., Zhou G., Hu W., Pei H. (2020). SMAD-6, -7 and -9 are potential molecular biomarkers for the prognosis in human lung cancer. Oncol. Lett..

[536] Mondal A., NeMoyer R., Vora M., Napoli L., Syed Z., Langenfeld E., Jia D., Peng Y., Gilleran J., Roberge J. (2021). Bone morphogenetic protein receptor 2 inhibition destabilizes microtubules promoting the activation of lysosomes and cell death of lung cancer cells. Cell Commun. Signal..

[537] Ma J., Bai Y., Liu M., Jiao T., Chen Y., Yuan B., Liu B., Zeng L., Ming Z., Li W. (2022). Pretreatment HDL-C and ApoA1 are predictive biomarkers of progression-free survival in patients with EGFR mutated advanced non-small cell lung cancer treated with TKI. Thorac. Cancer.

[538] Liu C., Yang Z., Deng Z., Zhou Y., Gong Q., Zhao R., Chen T. (2018). Upregulated lncRNA ADAMTS9-AS2 suppresses progression of lung cancer through inhibition of miR-223-3p and promotion of TGFBR3. IUBMB Life.

[539] Zhu L.Y., Yuan J.B., Zhang L., He C.X., Lin X., Xu B., Jin G.H. (2022). Loss of MLL Induces Epigenetic Dysregulation of Rasgrf1 to Attenuate Kras-Driven Lung Tumorigenesis. Cancer Res..

[540] Jung K., Choi J.S., Koo B.M., Kim Y.J., Song J.Y., Sung M., Chang E.S., Noh K.W., An S., Lee M.S. (2021). TM4SF4 and LRRK2 Are Potential Therapeutic Targets in Lung and Breast Cancers through Outlier Analysis. Cancer Res. Treat..

[541] Wang X., Shi D., Zhao D., Hu D. (2020). Aberrant Methylation and Differential Expression of SLC2A1, TNS4, GAPDH, ATP8A2, and CASZ1 Are Associated with the Prognosis of Lung Adenocarcinoma. Biomed. Res. Int..

[542] Bianchi F., Alexiadis S., Camisaschi C., Truini M., Centonze G., Milione M., Balsari A., Tagliabue E., Sfondrini L. (2020). TLR3 Ex-pression Induces Apoptosis in Human Non-Small-Cell Lung Cancer. Int. J. Mol. Sci..

[543] Wang M., Liu Y., Qian X., Wei N., Tang Y., Yang J. (2018). Downregulation of occludin affects the proliferation, apoptosis and metastatic properties of human lung carcinoma. Oncol. Rep..

[544] Ma Y., Schröder D.C., Nenkov M., Rizwan M.N., Abubrig M., Sonnemann J., Murrieta-Coxca J.M., Morales-Prieto D.M., Westermann M., Gaßler N. (2021). Epithelial Membrane Protein 2 Suppresses Non-Small Cell Lung Cancer Cell Growth by Inhibition of MAPK Pathway. Int. J. Mol. Sci..

[545] Tang Z., Wang L., Bajinka O., Wu G., Tan Y. (2022). Abnormal Gene Expression Regulation Mechanism of Myeloid Cell Nuclear Differentiation Antigen in Lung Adenocarcinoma. Biology.

[546] Cherfils-Vicini J., Platonova S., Gillard M., Laurans L., Validire P., Caliandro R., Magdeleinat P., Mami-Chouaib F., Dieu-Nosjean M.C., Fridman W.H. (2010). Triggering of TLR7 and TLR8 expressed by human lung cancer cells induces cell survival and chemoresistance. J. Clin. Investig..

[547] Liu W., Zhang T., Guo L., Wang Y., Yang Y. (2018). Lysyl hydroxylases are transcription targets for GATA3 driving lung cancer cell metastasis. Sci. Rep..

[548] Xu Q., Xu Z. (2020). miR-196b-5p Promotes Proliferation, Migration and Invasion of Lung Adenocarcinoma Cells via Targeting RSPO2. Cancer Manag. Res..

[549] He M., Yu W., Chang C., Miyamoto H., Liu X., Jiang K., Yeh S. (2020). Estrogen receptor α promotes lung cancer cell invasion via increase of and cross-talk with infiltrated macrophages through the CCL2/CCR2/MMP9 and CXCL12/CXCR4 signaling pathways. Mol. Oncol..

[550] Wu D., Deng S., Li L., Liu T., Zhang T., Li J., Yu Y., Xu Y. (2021). TGF-β1-mediated exosomal lnc-MMP2-2 increases blood-brain barrier permeability via the miRNA-1207-5p/EPB41L5 axis to promote non-small cell lung cancer brain metastasis. Cell Death Dis..

[551] Murugesan K., Jin D.X., Comment L.A., Fabrizio D., Hegde P.S., Elvin J.A., Alexander B., Levy M.A., Frampton G.M., Montesion M. (2022). Association of CD274 (PD-L1) Copy Number Changes with Immune Checkpoint Inhibitor Clinical Benefit in Non-Squamous Non-Small Cell Lung Cancer. Oncologist.

[552] Im J.Y., Lee K.W., Won K.J., Kim B.K., Ban H.S., Yoon S.H., Lee Y.J., Kim Y.J., Song K.B., Won M. (2016). DNA damage-induced apoptosis suppressor (DDIAS), a novel target of NFATc1, is associated with cisplatin resistance in lung cancer. Biochim. Biophys. Acta.

[553] Zhou J., Xu Y., Wang G., Mei T., Yang H., Liu Y. (2022). The TLR7/8 agonist R848 optimizes host and tumor immunity to improve therapeutic efficacy in murine lung cancer. Int. J. Oncol..

[554] Sozio F., Schioppa T., Laffranchi M., Salvi V., Tamassia N., Bianchetto-Aguilera F.M., Tiberio L., Bonecchi R., Bosisio D., Parmentier M. (2023). CCRL2 Expression by Specialized Lung Capillary Endothelial Cells Controls NK-cell Homing in Lung Cancer. Cancer Immunol. Res..

[555] Huang W., Xu X., Liu M., Cui W., Peng G. (2020). Downregulation of Hsa_circ_0000735 Inhibits the Proliferation, Migration, Invasion, and Glycolysis in Non-small-cell Lung Cancer by Targeting miR-940/BMPER Axis. OncoTargets Ther..

[556] Cui Z., Li D., Zhao J., Chen K. (2022). Falnidamol and cisplatin combinational treatment inhibits non-small cell lung cancer (NSCLC) by targeting DUSP26-mediated signal pathways. Free Radic. Biol. Med..

[557] Li P., Cong Z., Qiang Y., Xiong L., Tang L., Zhang Y., Wu H., Yi J., Jing H., Li D. (2018). Clinical significance of CCBE1 expression in lung cancer. Mol. Med. Rep..

[558] Liu R., Chen Y., Shou T., Hu J., Qing C. (2019). miRNA-99b-5p targets FZD8 to inhibit non-small cell lung cancer proliferation, migration and invasion. OncoTargets Ther..

[559] Ali A., Levantini E., Fhu C.W., Teo J.T., Clohessy J.G., Goggi J.L., Wu C.S., Chen L., Chin T.M., Tenen D.G. (2019). CAV1—GLUT3 signaling is important for cellular energy and can be targeted by Atorvastatin in Non-Small Cell Lung Cancer. Theranostics.

[560] Shen H., Wang L., Zhang J., Dong W., Zhang T., Ni Y., Cao H., Wang K., Li Y., Wang Y. (2017). ARRB1 enhances the chemosensitivity of lung cancer through the mediation of DNA damage response. Oncol. Rep..

[561] Yu X., Rao J., Lin J., Zhang Z., Cao L., Zhang X. (2014). Tag SNPs in complement receptor-1 contribute to the susceptibility to non-small cell lung cancer. Mol. Cancer.

[562] Wang G., Zhou Y., Chen W., Yang Y., Ye J., Ou H., Wu H. (2020). miR-21-5p promotes lung adenocarcinoma cell proliferation, migration and invasion via targeting WWC2. Cancer Biomarkers.

[563] Li J., Han R., Li J., Zhai L., Xie X., Zhang J., Chen Y., Luo J., Wang S., Sun Z. (2021). Analysis of Molecular Mechanism of YiqiChutan Formula Regulating DLL4-Notch Signaling to Inhibit Angiogenesis in Lung Cancer. Biomed. Res. Int..

[564] Liu Q., Wang S., Pei G., Yang Y., Min X., Huang Y., Liu J. (2021). Impact Analysis of miR-1253 on Lung Cancer Progression Through Targeted Regulation of ANXA3. Cancer Manag. Res..

[565] Zhang M., Li J., Lin W., Qi L., Yao C., Zheng Z., Chen C., Duan S., Qi Y. (2022). EPAS1 Promoter Hypermethylation is a Diagnostic and Prognostic Biomarker for Non-Small Cell Lung Cancer. Genet. Test. Mol. Biomarkers.

[566] Wang D., Lin Y., Gao B., Yan S., Wu H., Li Y., Wu Q., Wei Y. (2016). Reduced Expression of FADS1 Predicts Worse Prognosis in Non-Small-Cell Lung Cancer. J. Cancer.

[567] Song W., Wu X., Cheng C., Li D., Chen J., Zhang W. (2023). ARHGAP9 knockdown promotes lung adenocarcinoma metastasis by activating Wnt/β-catenin signaling pathway via suppressing DKK2. Genomics.

[568] Li Z., Pan Y., Liu Q., Wang J., Liu C., Qu L., Li D. (2022). Role of GPER1 in the Mechanism of EGFR-TKIs Resistance in Lung Adenocarcinoma. Front. Oncol..

[569] Dai L., Zhao J., Yin J., Fu W., Chen G. (2020). Cell adhesion molecule 2 (CADM2) promotes brain metastasis by inducing epithelial-mesenchymal transition (EMT) in human non-small cell lung cancer. Ann. Transl. Med..

[570] Zhou Q., Dai J., Chen T., Dada L.A., Zhang X., Zhang W., DeCamp M.M., Winn R.A., Sznajder J.I., Zhou G. (2017). Downregulation of PKCζ/Pard3/Pard6b is responsible for lung adenocarcinoma cell EMT and invasion. Cell. Signal..

[571] Kang X., Kong F., Huang K., Li L., Li Z., Wang X., Zhang W., Wu X. (2019). LncRNA MIR210HG promotes proliferation and invasion of non-small cell lung cancer by upregulating methylation of CACNA2D2 promoter via binding to DNMT1. OncoTargets Ther..

[572] Li D., Xu T., Wang X., Ma X., Liu T., Wang Y., Jiang S. (2017). The role of ATP8A1 in non-small cell lung cancer. Int. J. Clin. Exp. Pathol..

[573] Tan T., Ma M., Xing S. (2023). Effect of circ_0000009 on lung adenocarcinoma progression by regulating PDZD2 in a ceRNA- and RBP- dependent manner. Gene.

[574] Wang W., Lin Y., Zhang G., Shi G., Jiang Y., Hu W., Zuo W. (2021). circ_0002346 Suppresses Non-Small-Cell Lung Cancer Progression Depending on the Regulation of the miR-582-3p/STXBP6 Axis. Int. J. Genom..

[575] Mao G., Mu Z., Wu D. (2021). Exosome-derived miR-2682-5p suppresses cell viability and migration by HDAC1-silence-mediated upregulation of ADH1A in non-small cell lung cancer. Hum. Exp. Toxicol..

[576] Zhai X., Wu Q., Pu D., Yin L., Wang W., Zhu D., Xu F. (2022). Case Report: A Novel Non-Reciprocal ALK Fusion: ALK-GCA and EML4-ALK Were Identified in Lung Adenocarcinoma, Which May Respond to Alectinib Adjuvant-Targeted Therapy. Front Oncol..

[577] Guo W., Shao F., Sun S., Song P., Guo L., Xue X., Zhang G., Zhang H., Gao Y., Qiu B. (2020). Loss of SUSD2 expression correlates with poor prognosis in patients with surgically resected lung adenocarcinoma. J. Cancer.

[578] Jeong D., Ban S., Oh S., Lee S.J., Park S.Y., Koh Y.W. (2017). Prognostic Significance of EDIL3 Expression and Correlation with Mesenchymal Phenotype and Microvessel Density in Lung Adenocarcinoma. Sci. Rep..

[579] Shi Q., Shi Q.N., Xu J.W., Wang H.Y., Li Y.J., Zhang X.X., Fu Y.H., Tian R.H., Jiang R., Liu C.C. (2022). rs9390123 and rs9399451 influence the DNA repair capacity of lung cancer by regulating PEX3 and PHACTR2-AS1 expression instead of PHACTR2. Oncol. Rep..

[580] Li M., Lin A., Luo P., Shen W., Xiao D., Gou L., Zhang J., Guo L. (2020). DNAH10 mutation correlates with cisplatin sensitivity and tumor mutation burden in small-cell lung cancer. Aging.

[581] Zhang Z., Xu P., Hu Z., Fu Z., Deng T., Deng X., Peng L., Xie Y., Long L., Zheng D. (2022). CCDC65, a Gene Knockout that leads to Early Death of Mice, acts as a potentially Novel Tumor Suppressor in Lung Adenocarcinoma. Int. J. Biol. Sci..

[582] Wu Q., Yan Y., Shi S., Qi Q., Han J. (2022). DNMT3b-mediated SPAG6 promoter hypermethylation affects lung squamous cell carcinoma development through the JAK/STAT pathway. Am. J. Transl. Res..

[583] Yu F.Y., Xu Q., Zhao X.Y., Mo H.Y., Zhong Q.H., Luo L., Lau A.T.Y., Xu Y.M. (2023). The Atypical MAP Kinase MAPK15 Is Required for Lung Adenocarcinoma Metastasis via Its Interaction with NF-κB p50 Subunit and Transcriptional Regulation of Prostaglandin E2 Receptor EP3 Subtype. Cancers.

[584] Ma Q., Lu Y., Lin J., Gu Y. (2019). ENKUR acts as a tumor suppressor in lung adenocarcinoma cells through PI3K/Akt and MAPK/ERK signaling pathways. J. Cancer.

[585] Li F., Fang Z., Zhang J., Li C., Liu H., Xia J., Zhu H., Guo C., Qin Z., Li F. (2016). Identification of TRA2B-DNAH5 fusion as a novel oncogenic driver in human lung squamous cell carcinoma. Cell Res..

[586] Roh J.I., Lee J., Sung Y.H., Oh J., Hyeon D.Y., Kim Y., Lee S., Devkota S., Kim H.J., Park B. (2020). mpaired AKT signaling and lung tumorigenesis by PIERCE1 ablation in KRAS-mutant non-small cell lung cancer. Oncogene.

[587] Li Y., Bai M., Xu Y., Zhao W., Liu N., Yu J. (2018). TPPP3 Promotes Cell Proliferation, Invasion and Tumor Metastasis via STAT3/Twist1 Pathway in Non-Small-Cell Lung Carcinoma. Cell. Physiol. Biochem..

[588] Wang W., Ren S., Wang Z., Zhang C., Huang J. (2020). Increased expression of TTC21A in lung adenocarcinoma infers favorable prognosis and high immune infiltrating level. Int. Immunopharmacol..

[589] Li X., Mao W., Guo D., Xu H. (2019). Clinicopathological Significance and Diagnostic Value of DLEC1 Hypermethylation in Lung Cancer: A Meta-analysis. J. Nippon. Med. Sch..

[590] Hong W., Yu S., Zhuang Y., Zhang Q., Wang J., Gao X. (2020). SRCIN1 Regulated by circCCDC66/miR-211 Is Upregulated and Promotes Cell Proliferation in Non-Small-Cell Lung Cancer. Biomed. Res. Int..

[591] Qiu Z.X., Zhao S., Mo X.M., Li W.M. (2015). Overexpression of PROM1 (CD133) confers poor prognosis in non-small cell lung cancer. Int. J. Clin. Exp. Pathol..

[592] Jaskiewicz L., Romaszko-Wojtowicz A., Doboszynska A., Skowronska A. (2023). The Role of Aquaporin 5 (AQP5) in Lung Adenocarcinoma: A Review Article. Cells.

[593] Zhang L., Fan B., Zheng Y., Lou Y., Cui Y., Wang K., Zhang T., Tan X. (2020). Identification SYT13 as a novel biomarker in lung adenocarcinoma. J. Cell. Biochem..

[594] Dmitriev A.A., Kashuba V.I., Haraldson K., Senchenko V.N., Pavlova T.V., Kudryavtseva A.V., Anedchenko E.A., Krasnov G.S., Pronina I.V., Loginov V.I. (2012). Genetic and epigenetic analysis of non-small cell lung cancer with NotI-microarrays. Epigenetics.

[595] Wu C., Dong B., Huang L., Liu Y., Ye G., Li S., Qi Y. (2021). SPTBN2, a New Biomarker of Lung Adenocarcinoma. Front. Oncol..

[596] Pang Y., Zhang Y., Zhang H.Y., Wang W.H., Jin G., Liu J.W., Zhu Z.J. (2022). MUC13 promotes lung cancer development and progression by activating ERK signaling. Oncol. Lett..

[597] Saad H.M., Tourky G.F., Al-Kuraishy H.M., Al-Gareeb A.I., Khattab A.M., Elmasry S.A., Alsayegh A.A., Hakami Z.H., Alsulimani A., Sabatier J.M. (2022). The Potential Role of MUC16 (CA125) Biomarker in Lung Cancer: A Magic Biomarker but with Adversity. Diagnostics.

[598] Yokoyama S., Higashi M., Tsutsumida H., Wakimoto J., Hamada T., Wiest E., Matsuo K., Kitazono I., Goto Y., Guo X. (2017). TET1-mediated DNA hypomethylation regulates the expression of MUC4 in lung cancer. Genes Cancer.

[599] Arai M., Ikawa Y., Chujo S., Hamaguchi Y., Ishida W., Shirasaki F., Hasegawa M., Mukaida N., Fujimoto M., Takehara K. (2013). Chemokine receptors CCR2 and CX3CR1 regulate skin fibrosis in the mouse model of cytokine-induced systemic sclerosis. J Dermatol. Sci..

[600] Omatsu J., Saigusa R., Miyagawa T., Fukui Y., Toyama S., Awaji K., Ikawa T., Norimatsu Y., Yoshizaki A., Sato S. (2021). Serum S100A12 levels: Possible association with skin sclerosis and interstitial lung disease in systemic sclerosis. Exp. Dermatol..

[601] Kowal-Bielecka O., Kowal K., Lewszuk A., Bodzenta-Lukaszyk A., Walecki J., Sierakowski S. (2005). Beta thromboglobulin and platelet factor 4 in bronchoalveolar lavage fluid of patients with systemic sclerosis. Ann. Rheum. Dis..

[602] Ruffatti A., Sinico R.A., Radice A., Ossi E., Cozzi F., Tonello M., Grypiotis P., Todesco S. (2002). Autoantibodies to proteinase 3 and myeloperoxidase in systemic sclerosis. J. Rheumatol..

[603] Corallo C., Pinto A.M., Renieri A., Cheleschi S., Fioravanti A., Cutolo M., Soldano S., Nuti R., Giordano N. (2019). Altered expression of RXFP1 receptor contributes to the inefficacy of relaxin-based anti-fibrotic treatments in systemic sclerosis. Clin. Exp. Rheumatol..

[604] Van Bon L., Cossu M., Loof A., Gohar F., Wittkowski H., Vonk M., Roth J., van den Berg W., van Heerde W., Broen J.C. (2014). Proteomic analysis of plasma identifies the Toll-like receptor agonists S100A8/A9 as a novel possible marker for systemic sclerosis phenotype. Ann. Rheum. Dis..

[605] Honda N., Jinnin M., Kajihara I., Makino T., Fukushima S., Ihn H. (2010). Impaired lymphangiogenesis due to excess vascular endothelial growth factor-D/Flt-4 signalling in the skin of patients with systemic sclerosis. Br. J. Dermatol..

[606] Crescioli C., Corinaldesi C., Riccieri V., Raparelli V., Vasile M., Del Galdo F., Valesini G., Lenzi A., Basili S., Antinozzi C. (2018). Association of circulating CXCL10 and CXCL11 with systemic sclerosis. Ann. Rheum. Dis..

[607] Kawaguchi Y., Tochimoto A., Ichikawa N., Harigai M., Hara M., Kotake S., Kitamura Y., Kamatani N. (2003). Association of IL1A gene polymorphisms with susceptibility to and severity of systemic sclerosis in the Japanese population. Arthritis Rheum..

[608] Khanna D., Aggarwal A., Bhakuni D.S., Dayal R., Misra R. (2003). Bactericidal/permeability-increasing protein and cathepsin G are the major antigenic targets of antineutrophil cytoplasmic autoantibodies in systemic sclerosis. J. Rheumatol..

[609] Wipff J., Bonnet P., Ruiz B., Dieude P., Avouac J., Tiev K., Hachulla E., Cracowski J.L., Diot E., Sibilia J. (2010). Association study of serotonin transporter gene (SLC6A4) in systemic sclerosis in European Caucasian populations. J. Rheumatol..

[610] Colasanti T., Stefanantoni K., Fantini C., Corinaldesi C., Vasile M., Marampon F., Di Luigi L., Antinozzi C., Sgrò P., Lenzi A. (2022). The Prostacyclin Analogue Iloprost Modulates CXCL10 in Systemic Sclerosis. Int. J. Mol. Sci..

[611] McKinney C., Broen J.C., Vonk M.C., Beretta L., Hesselstrand R., Hunzelmann N., Riemekasten G., Scorza R., Simeon C.P., Fonollosa V. (2012). Evidence that deletion at FCGR3B is a risk factor for systemic sclerosis. Genes Immun..

[612] Nikitorowicz-Buniak J., Shiwen X., Denton C.P., Abraham D., Stratton R. (2014). Abnormally differentiating keratinocytes in the epidermis of systemic sclerosis patients show enhanced secretion of CCN2 and S100A9. J. Investig. Dermatol..

[613] Mattuzzi S., Barbi S., Carletto A., Ravagnani V., Moore P.S., Bambara L.M., Scarpa A. (2007). Association of polymorphisms in the IL1B and IL2 genes with susceptibility and severity of systemic sclerosis. J. Rheumatol..

[614] Salim P.H., Jobim M., Bredemeier M., Chies J.A., Brenol J.C., Jobim L.F., Xavier R.M. (2012). Combined effects of CXCL8 and CXCR2 gene polymorphisms on susceptibility to systemic sclerosis. Cytokine.

[615] Stern E.P., Guerra S.G., Chinque H., Acquaah V., González-Serna D., Ponticos M., Martin J., Ong V.H., Khan K., Nihtyanova S.I. (2020). Analysis of Anti-RNA Polymerase III Antibody-positive Systemic Sclerosis and Altered GPATCH2L and CTNND2 Expression in Scleroderma Renal Crisis. J. Rheumatol..

[616] Bassyouni I.H., Gheita T.A., Talaat R.M. (2011). Clinical significance of serum levels of sCD36 in patients with systemic sclerosis: Preliminary data. Rheumatology.

[617] Ferraz-Amaro I., Delgado-Frías E., Hernández-Hernández V., Sánchez-Pérez H., de Armas-Rillo L., García-Dopico J.A., Díaz-González F. (2020). Proprotein convertase subtilisin/kexin type 9 in patients with systemic sclerosis. Clin. Exp. Rheumatol..

[618] İlgen U., Yayla M.E., Düzgün N. (2017). Low serum fibroblast growth factor 2 levels not accompanied by increased serum pentraxin 3 levels in patients with systemic sclerosis. Clin. Rheumatol..

[619] Horn A., Palumbo K., Cordazzo C., Dees C., Akhmetshina A., Tomcik M., Zerr P., Avouac J., Gusinde J., Zwerina J. (2012). Hedgehog signaling controls fibroblast activation and tissue fibrosis in systemic sclerosis. Arthritis Rheum..

[620] Talotta R., Rigamonti F., Letizia T., Bongiovanni S., Ditto M.C., Antivalle M., Santandrea S., Atzeni F., Vago T., Sarzi-Puttini P. (2019). Serum klotho concentrations inversely correlate with the severity of nailfold capillaroscopic patterns in patients with systemic sclerosis. Reumatismo.

[621] Xu B., Xu G., Yu Y., Lin J. (2021). The role of TGF-β or BMPR2 signaling pathway-related miRNA in pulmonary arterial hypertension and systemic sclerosis. Arthritis Res. Ther..

[622] Ah Kioon M.D., Tripodo C., Fernandez D., Kirou K.A., Spiera R.F., Crow M.K., Gordon J.K., Barrat F.J. (2018). Plasmacytoid dendritic cells promote systemic sclerosis with a key role for TLR8. Sci. Transl. Med..

[623] Baraut J., Farge D., Jean-Louis F., Masse I., Grigore E.I., Arruda L.C., Lamartine J., Verrecchia F., Michel L. (2015). Transforming growth factor-β increases interleukin-13 synthesis via GATA-3 transcription factor in T-lymphocytes from patients with systemic sclerosis. Arthritis Res. Ther..

[624] Carulli M.T., Ong V.H., Ponticos M., Shiwen X., Abraham D.J., Black C.M., Denton C.P. (2005). Chemokine receptor CCR2 ex-pression by systemic sclerosis fibroblasts: Evidence for autocrine regulation of myofibroblast differentiation. Arth. Rheum..

[625] Vreća M., Zeković A., Damjanov N., Andjelković M., Ugrin M., Pavlović S., Spasovski V. (2018). Expression of TLR7, TLR9, JAK2, and STAT3 genes in peripheral blood mononuclear cells from patients with systemic sclerosis. J. Appl. Genet..

[626] Lee R., Del Papa N., Introna M., Reese C.F., Zemskova M., Bonner M., Carmen-Lopez G., Helke K., Hoffman S., Tourkina E. (2019). Adipose-derived mesenchymal stromal/stem cells in systemic sclerosis: Alterations in function and beneficial effect on lung fibrosis are regulated by caveolin-1. J. Scleroderma Relat. Disord..

[627] Norimatsu Y., Miyagawa T., Fukui Y., Omatsu J., Toyama S., Awaji K., Ikawa T., Watanabe Y., Yoshizaki A., Sato S. (2021). Serum levels of tissue factor pathway inhibitor: Potential association with Raynaud’s phenomenon and telangiectasia in patients with systemic sclerosis. J. Dermatol..

[628] Sapao P., Roberson E.D.O., Shi B., Assassi S., Skaug B., Lee F., Naba A., Perez White B.E., Córdova-Fletes C., Tsou P.S. (2023). Reduced SPAG17 Expression in Systemic Sclerosis Triggers Myofibroblast Transition and Drives Fibrosis. J. Investig. Dermatol..

[629] Qiu F., Li Y., Zhu Y., Li G., Lei F., Zhang S., Luo L., Zhu J., Guo Y., Du B. (2021). CX3CR1 might be a promising predictor of systemic lupus erythematosus patients with pulmonary fibrosis. Scand. J. Immunol..

[630] Bobek D., Sestan M., Mijacika L., Kovacic N., Lukic I.K., Grcevic D., Jelusic M. (2023). Serum S100A12 levels in children with childhood-onset systemic lupus erythematosus, systemic juvenile arthritis, and systemic undefined recurrent fevers. Z. Rheumatol..

[631] Heidari Z., Mahmoudzadeh Sagheb H., Sheibak N. (2016). Immunohistochemical Expression of Myeloperoxidase in Placental Samples of Systematic Lupus Erythematosus Pregnancies. J. Fam. Reprod. Health.

[632] Lai X., Xiang Y., Zou L., Li Y., Zhang L. (2018). Elevation of serum CD5L concentration is correlated with disease activity in patients with systemic lupus erythematosus. Int. Immunopharmacol..

[633] Vercellotti G.M., Mosher D.F. (1982). Acquired factor XI deficiency in systemic lupus erythematosus. Thromb. Haemost..

[634] Kitagori K., Oku T., Wakabayashi M., Nakajima T., Nakashima R., Murakami K., Hirayama Y., Ishihama Y., Ohmura K., Morinobu A. (2023). Expression of S100A8 protein on B cells is associated with disease activity in patients with systemic lupus erythematosus. Arthritis Res. Ther..

[635] Chuang H.C., Chen M.H., Chen Y.M., Yang H.Y., Ciou Y.R., Hsueh C.H., Tsai C.Y., Tan T.H. (2021). BPI overexpression suppresses Treg differentiation and induces exosome-mediated inflammation in systemic lupus erythematosus. Theranostics.

[636] Asgari N., Jarius S., Laustrup H., Skejoe H.P., Lillevang S.T., Weinshenker B.G., Voss A. (2018). Aquaporin-4-autoimmunity in patients with systemic lupus erythematosus: A predominantly population-based study. Mult. Scler..

[637] Ding J., Li C., Shu K., Chen W., Cai C., Zhang X., Zhang W. (2023). Membrane metalloendopeptidase (MME) is positively correlated with systemic lupus erythematosus and may inhibit the occurrence of breast cancer. PLoS ONE.

[638] Shobeiri P., Maleki S., Amanollahi M., Habibzadeh A., Teixeira A.L., Rezaei N. (2023). Blood levels of brain-derived neurotrophic factor (BDNF) in systemic lupus erythematous (SLE): A systematic review and meta-analysis. Adv. Rheumatol..

[639] Zhang R., Li Y., Pan B., Li Y., Liu A., Li X. (2019). Increased expression of hub gene CXCL10 in peripheral blood mononuclear cells of patients with systemic lupus erythematosus. Exp. Ther. Med..

[640] Zhu Y., Tang X., Xu Y., Wu S., Liu W., Geng L., Ma X., Tsao B.P., Feng X., Sun L. (2022). RNASE2 Mediates Age-Associated B Cell Expansion Through Monocyte Derived IL-10 in Patients with Systemic Lupus Erythematosus. Front. Immunol..

[641] Chen J.Y., Wang C.M., Chang S.W., Cheng C.H., Wu Y.J., Lin J.C., Yang B., Ho H.H., Wu J. (2014). Association of FCGR3A and FCGR3B copy number variations with systemic lupus erythematosus and rheumatoid arthritis in Taiwanese patients. Arthritis Rheumatol..

[642] Lood C., Tydén H., Gullstrand B., Jönsen A., Källberg E., Mörgelin M., Kahn R., Gunnarsson I., Leanderson T., Ivars F. (2016). Platelet-Derived S100A8/A9 and Cardiovascular Disease in Systemic Lupus Erythematosus. Arthritis Rheumatol..

[643] Kunitsu T., Harada-Shiba M., Sato T., Nonomura K., Kimura T., Miyashita K., Nakajima K., Murakami M. (2022). Development of hypertriglyceridemia due to GPIHBP1 autoantibodies prior to clinical diagnosis of systemic lupus erythematosus in a 14-year-old girl. Allergol. Int..

[644] Cen H., Leng R.X., Wang W., Zhou M., Feng C.C., Chen G.M., Li R., Pan H.F., Li X.P., Ye D.Q. (2012). Association of AFF1 rs340630 and AFF3 rs10865035 polymorphisms with systemic lupus erythematosus in a Chinese population. Immunogenetics.

[645] Wajda A., Sowińska A., Haładyj E., Stypińska B., Nałęcz-Janik J., Jagodziński P.P., Majewski D., Olesińska M., Paradowska-Gorycka A. (2021). Tissue factor and human apolipoprotein H genetic variants and pro-inflammatory cytokines in systemic lupus erythematosus patients. Clin. Exp. Rheumatol..

[646] Troldborg A., Steffensen R., Trendelenburg M., Hauser T., Winther K.G., Hansen A.G., Stengaard-Pedersen K., Voss A., Thiel S. (2019). Ficolin-3 Deficiency Is Associated with Disease and an Increased Risk of Systemic Lupus Erythematosus. J. Clin. Immunol..

[647] Mamegano K., Kuroki K., Miyashita R., Kusaoi M., Kobayashi S., Matsuta K., Maenaka K., Colonna M., Ozaki S., Hashimoto H. (2008). Association of LILRA2 (ILT1, LIR7) splice site polymorphism with systemic lupus erythematosus and microscopic polyangiitis. Genes Immun..

[648] Abd-Elmawla M.A., Fawzy M.W., Rizk S.M., Shaheen A.A. (2018). Role of long non-coding RNAs expression (ANRIL, NOS3-AS, and APOA1-AS) in development of atherosclerosis in Egyptian systemic lupus erythematosus patients. Clin. Rheumatol..

[649] Zhang M., Yao C., Cai J., Liu S., Liu X.N., Chen Y., Wang S., Ji P., Pan M., Kang Z. (2019). LRRK2 is involved in the pathogenesis of system lupus erythematosus through promoting pathogenic antibody production. J. Transl. Med..

[650] Malaer J.D., Marrufo A.M., Mathew P.A. (2019). 2B4 (CD244, SLAMF4) and CS1 (CD319, SLAMF7) in systemic lupus erythematosus and cancer. Clin. Immunol..

[651] Enevold C., Kjær L., Nielsen C.H., Voss A., Jacobsen R.S., Hermansen M.L., Redder L., Oturai A.B., Jensen P.E., Bendtzen K. (2014). Genetic polymorphisms of dsRNA ligating pattern recognition receptors TLR3, MDA5, and RIG-I. Association with systemic lupus erythematosus and clinical phenotypes. Rheumatol. Int..

[652] Young N.A., Valiente G.R., Hampton J.M., Wu L.C., Burd C.J., Willis W.L., Bruss M., Steigelman H., Gotsatsenko M., Amici S.A. (2017). Estrogen-regulated STAT1 activation promotes TLR8 expression to facilitate signaling via microRNA-21 in systemic lupus erythematosus. Clin. Immunol..

[653] Kato H., Perl A. (2021). Double-Edged Sword: Interleukin-2 Promotes T Regulatory Cell Differentiation but Also Expands Interleukin-13- and Interferon-γ-Producing CD8+ T Cells via STAT6-GATA-3 Axis in Systemic Lupus Erythematosus. Front. Immunol..

[654] Pan Q., Feng Y., Peng Y., Zhou H., Deng Z., Li L., Han H., Lin J., Shi L., Wang S. (2017). Basophil Recruitment to Skin Lesions of Patients with Systemic Lupus Erythematosus Mediated by CCR1 and CCR2. Cell. Physiol. Biochem..

[655] Wang J., Dai M., Cui Y., Hou G., Deng J., Gao X., Liao Z., Liu Y., Meng Y., Wu L. (2018). Association of Abnormal Elevations in IFIT3 With Overactive Cyclic GMP-AMP Synthase/Stimulator of Interferon Genes Signaling in Human Systemic Lupus Erythematosus Monocytes. Arthritis Rheumatol..

[656] Ma Z.Z., Sun H.S., Lv J.C., Guo L., Yang Q.R. (2018). Expression and clinical significance of the NEK7-NLRP3 inflammasome signaling pathway in patients with systemic lupus erythematosus. J. Inflamm..

[657] Mauro D., Manou-Stathopoulou S., Rivellese F., Sciacca E., Goldmann K., Tsang V., Lucey-Clayton I., Pagani S., Alam F., Pyne D. (2023). UBE2L3 regulates TLR7-induced B cell autoreactivity in Systemic Lupus Erythematosus. J. Autoimmun..

[658] Spies C.M., Schaumann D.H., Berki T., Mayer K., Jakstadt M., Huscher D., Wunder C., Burmester G.R., Radbruch A., Lauster R. (2006). Membrane glucocorticoid receptors are down regulated by glucocorticoids in patients with systemic lupus erythematosus and use a caveolin-1-independent expression pathway. Ann. Rheum. Dis..

[659] Nath S.K., Harley J.B., Lee Y.H. (2005). Polymorphisms of complement receptor 1 and interleukin-10 genes and systemic lupus erythematosus: A meta-analysis. Hum. Genet..

[660] Ertenli I., Kiraz S., Celik I.C., Haznedaroglu C., Erman M., Calgüneri M., Kirazli S. (2001). Changes in the concentration and distribution of tissue factor pathway inhibitor in Behçet’s disease and systemic lupus erythematosus: Effect on the prethrombotic state. Ann. Rheum. Dis..

[661] Zhang X., Qian H., Chen Y., Wu Y., Sun Y., He Y., Chen S., Shi G., Liu Y. (2023). Autoantibodies targeting to GPER1 promote monocyte cytokines production and inflammation in systemic lupus erythematosus. Signal Transduct. Target. Ther..

[662] Thornhill S.I., Mak A., Lee B., Lee H.Y., Poidinger M., Connolly J.E., Fairhurst A.M. (2017). Monocyte Siglec-14 expression is upregulated in patients with systemic lupus erythematosus and correlates with lupus disease activity. Rheumatology.

[663] Li C.S., Zhang Q., Lim M.K., Sheen D.H., Shim S.C., Kim J.Y., Lee S.S., Yun K.J., Moon H.B., Chung H.T. (2007). Association of FOXJ1 polymorphisms with systemic lupus erythematosus and rheumatoid arthritis in Korean population. Exp. Mol. Med..

[664] Kim H.S., Jin E.H., Mo J.S., Shim H., Lee S.S., Chae S.C. (2015). The Association of the GABRP Polymorphisms with Systemic Lupus Erythematosus. J. Immunol. Res..

[665] Smith C.J., Oscarson M., Rönnblom L., Alimohammadi M., Perheentupa J., Husebye E.S., Gustafsson J., Nordmark G., Meloni A., Crock P.A. (2011). TSGA10—A target for autoantibodies in autoimmune polyendocrine syndrome type 1 and systemic lupus erythematosus. Scand. J. Immunol..

[666] Runyan C.E., Welch L.C., Lecuona E., Shigemura M., Amarelle L., Abdala-Valencia H., Joshi N., Lu Z., Nam K., Markov N.S. (2020). Impaired phagocytic function in CX3CR1+ tissue-resident skeletal muscle macrophages prevents muscle recovery after influenza A virus-induced pneumonia in old mice. Aging Cell.

[667] Jiang X., Huang C.M., Feng C.M., Xu Z., Fu L., Wang X.M. (2022). Corrigendum: Associations of Serum S100A12 With Severity and Prognosis in Patients with Community-Acquired Pneumonia: A Prospective Cohort Study. Front. Immunol..

[668] Arao Y., Stumpo D.J., Hoenerhoff M.J., Tighe R.M., Yu Y.R., Sutton D., Kashyap A., Beerman I., Blackshear P.J. (2023). Lethal eosinophilic crystalline pneumonia in mice expressing a stabilized Csf2 mRNA. FASEB J..

[669] Bando M., Homma S., Harigai M. (2022). MPO-ANCA positive interstitial pneumonia: Current knowledge and future perspectives. Sarcoidosis Vasc. Diffuse Lung Dis..

[670] Chen T., Duan J., Li M., Wu X., Cao J. (2020). Assessment of serum CD5L as a biomarker to distinguish etiology and predict mortality in adults with pneumonia. J. Infect..

[671] Salomon O., Preis M., Abu Shtaya A., Kotler A., Stein N., Saliba W. (2018). Factor XI deficiency is not associated with an increased risk of pneumonia and pneumonia-related mortality. Haemophilia.

[672] Xie S., Wang J., Tuo W., Zhuang S., Cai Q., Yao C., Han F., Zhu H., Xiang Y., Yuan C. (2023). Serum level of S100A8/A9 as a biomarker for establishing the diagnosis and severity of community-acquired pneumonia in children. Front. Cell. Infect. Microbiol..

[673] Jia Y., Ren S., Song L., Wang S., Han W., Li J., Yu Y., Ma B. (2023). PGLYRP1-mIgG2a-Fc inhibits macrophage activation via AKT/NF-κB signaling and protects against fatal lung injury during bacterial infection. iScience.

[674] Carneiro A.S., Mafort T.T., Lopes A.J. (2021). A 34-Year-Old Woman from Brazil with Pulmonary Lymphangioleiomyomatosis Diagnosed by Raised Serum Vascular Endothelial Growth Factor-D (VEGF-D) Levels and Lung Cysts on Computed Tomography Imaging Presenting with COVID-19 Pneumonia. Am. J. Case Rep..

[675] Steiner P., Otth M., Casaulta C., Aebi C. (2009). Autoantibodies against bactericidal/permeability-increasing protein (BPI) in children with acute pneumonia. FEMS Immunol. Med. Microbiol..

[676] Li M., Chen Y., Li H., Yang D., Zhou Y., Chen Z., Zhang Y. (2021). Serum CXCL10/IP-10 may be a potential biomarker for severe Mycoplasma pneumoniae pneumonia in children. BMC Infect. Dis..

[677] Liu H.Y., Xiang H.X., Xiang Y., Xu Z., Feng C.M., Fei J., Fu L., Zhao H. (2021). The associations of serum S100A9 with the severity and prognosis in patients with community-acquired pneumonia: A prospective cohort study. BMC Infect. Dis..

[678] Wei J., Peng J., Wang B., Qu H., Wang S., Faisal A., Cheng J.W., Gordon J.R., Li F. (2013). CXCR1/CXCR2 antagonism is effective in pulmonary defense against Klebsiella pneumoniae infection. Biomed. Res. Int..

[679] Eisele N.A., Lee-Lewis H., Besch-Williford C., Brown C.R., Anderson D.M. (2011). Chemokine receptor CXCR2 mediates bacterial clearance rather than neutrophil recruitment in a murine model of pneumonic plague. Am. J. Pathol..

[680] Young L.R., Nogee L.M., Barnett B., Panos R.J., Colby T.V., Deutsch G.H. (2008). Usual interstitial pneumonia in an adolescent with ABCA3 mutations. Chest.

[681] Sharif O., Matt U., Saluzzo S., Lakovits K., Haslinger I., Furtner T., Doninger B., Knapp S. (2013). The scavenger receptor CD36 downmodulates the early inflammatory response while enhancing bacterial phagocytosis during pneumococcal pneumonia. J. Immunol..

[682] Coon D.R., Roberts D.J., Loscertales M., Kradin R. (2006). Differential epithelial expression of SHH and FOXF1 in usual and nonspecific interstitial pneumonia. Exp. Mol. Pathol..

[683] Iwata-Yoshikawa N., Nagata N., Takaki H., Matsumoto M., Suzuki T., Hasegawa H., Seya T. (2022). Prophylactic Vaccine Targeting TLR3 on Dendritic Cells Ameliorates Eosinophilic Pneumonia in a Mouse SARS-CoV Infection Model. Immunohorizons.

[684] Steichen A.L., Binstock B.J., Mishra B.B., Sharma J. (2013). C-type lectin receptor Clec4d plays a protective role in resolution of Gram-negative pneumonia. J. Leukoc. Biol..

[685] Cao M., Liu H., Dong Y., Liu W., Yu Z., Wang Q., Wang Q., Liang Z., Li Y., Ren H. (2021). Mesenchymal stem cells alleviate idiopathic pneumonia syndrome by modulating T cell function through CCR2-CCL2 axis. Stem Cell Res. Ther..

[686] Wang X., Zhao Y., Wang D., Liu C., Qi Z., Tang H., Liu Y., Zhang S., Cui Y., Li Y. (2023). ALK-JNK signaling promotes NLRP3 inflammasome activation and pyroptosis via NEK7 during Streptococcus pneumoniae infection. Mol. Immunol..

[687] Abolhassani H., Vosughimotlagh A., Asano T., Landegren N., Boisson B., Delavari S., Bastard P., Aranda-Guillén M., Wang Y., Zuo F. (2022). X-Linked TLR7 Deficiency Underlies Critical COVID-19 Pneumonia in a Male Patient with Ataxia-Telangiectasia. J. Clin. Immunol..

[688] An P., Li R., Wang J.M., Yoshimura T., Takahashi M., Samudralal R., O’Brien S.J., Phair J., Goedert J.J., Kirk G.D. (2011). Role of exonic variation in chemokine receptor genes on AIDS: CCRL2 F167Y association with pneumocystis pneumonia. PLoS Genet..

[689] Guo Q., Shen N., Yuan K., Li J., Wu H., Zeng Y., Fox J., Bansal A.K., Singh B.B., Gao H. (2012). Caveolin-1 plays a critical role in host immunity against Klebsiella pneumoniae by regulating STAT5 and Akt activity. Eur. J. Immunol..

[690] Lévy Y., Wiedemann A., Hejblum B.P., Durand M., Lefebvre C., Surénaud M., Lacabaratz C., Perreau M., Foucat E., Déchenaud M. (2021). CD177, a specific marker of neutrophil activation, is associated with coronavirus disease 2019 severity and death. iScience.

[691] Favaloro E.J., Pasalic L., Lippi G. (2022). Antibodies against Platelet Factor 4 and Their Associated Pathologies: From HIT/HITT to Spontaneous HIT-Like Syndrome, to COVID-19, to VITT/TTS. Antibodies.

[692] Wang G., Jiang L., Wang J., Zhang J., Kong F., Li Q., Yan Y., Huang S., Zhao Y., Liang L. (2020). The G Protein-Coupled Receptor FFAR2 Promotes Internalization during Influenza A Virus Entry. J. Virol..

[693] Goud P.T., Bai D., Abu-Soud H.M. (2021). A Multiple-Hit Hypothesis Involving Reactive Oxygen Species and Myeloperoxidase Explains Clinical Deterioration and Fatality in COVID-19. Int. J. Biol. Sci..

[694] Andreani G., Uscello L., Montaruli B., Briozzo A., Vitale F., Tricarico M., Arnaldi L., Marengo S., Norbiato C. (2020). Acquired Factor XI Deficiency during SARS-CoV-2 Infection: Not Only Thrombosis. TH Open.

[695] Mellett L., Khader S.A. (2022). S100A8/A9 in COVID-19 pathogenesis: Impact on clinical outcomes. Cytokine Growth Factor Rev..

[696] Liu Y., Li S., Zhang G., Nie G., Meng Z., Mao D., Chen C., Chen X., Zhou B., Zeng G. (2013). Genetic variants in IL1A and IL1B contribute to the susceptibility to 2009 pandemic H1N1 influenza A virus. BMC Immunol..

[697] Pinkenburg O., Meyer T., Bannert N., Norley S., Bolte K., Czudai-Matwich V., Herold S., Gessner A., Schnare M. (2016). The Human Antimicrobial Protein Bactericidal/Permeability-Increasing Protein (BPI) Inhibits the Infectivity of Influenza A Virus. PLoS ONE.

[698] Creed M.A., Ballesteros E., Greenfield L.J., Imitola J. (2020). Mild COVID-19 infection despite chronic B cell depletion in a patient with aquaporin-4-positive neuromyelitis optica spectrum disorder. Mult. Scler. Relat. Disord..

[699] Asgarzadeh A., Fouladi N., Asghariazar V., Sarabi S.F., Khiavi H.A., Mahmoudi M., Safarzadeh E. (2022). Serum Brain-Derived Neurotrophic Factor (BDNF) in COVID-19 Patients and its Association with the COVID-19 Manifestations. J. Mol. Neurosci..

[700] Aboagye J.O., Yew C.W., Ng O.W., Monteil V.M., Mirazimi A., Tan Y.J. (2018). Overexpression of the nucleocapsid protein of Middle East respiratory syndrome coronavirus up-regulates CXCL10. Biosci. Rep..

[701] Domachowske J.B., Dyer K.D., Bonville C.A., Rosenberg H.F. (1998). Recombinant human eosinophil-derived neurotoxin/RNase 2 functions as an effective antiviral agent against respiratory syncytial virus. J. Infect. Dis..

[702] Nassir N., Tambi R., Bankapur A., Al Heialy S., Karuvantevida N., Khansaheb H.H., Zehra B., Begum G., Hameid R.A., Ahmed A. (2021). Single-cell transcriptome identifies FCGR3B upregulated subtype of alveolar macrophages in patients with critical COVID-19. iScience.

[703] Oguariri R.M., Brann T.W., Adelsberger J.W., Chen Q., Goswami S., Mele A.R., Imamichi T. (2022). Short Communication: S100A8 and S100A9, Biomarkers of SARS-CoV-2 Infection and Other Diseases, Suppress HIV Replication in Primary Macrophages. AIDS Res. Hum. Retroviruses.

[704] García-Ramírez R.A., Ramírez-Venegas A., Quintana-Carrillo R., Camarena Á.E., Falfán-Valencia R., Mejía-Aranguré J.M. (2015). TNF, IL6, and IL1B Polymorphisms Are Associated with Severe Influenza A (H1N1) Virus Infection in the Mexican Population. PLoS ONE.

[705] Koenig L.M., Boehmer D.F.R., Metzger P., Schnurr M., Endres S., Rothenfusser S. (2020). Blocking inflammation on the way: Rationale for CXCR2 antagonists for the treatment of COVID-19. J. Exp. Med..

[706] Tang Z., Xu Y., Tan Y., Shi H., Jin P., Li Y., Teng J., Liu H., Pan H., Hu Q. (2023). CD36 mediates SARS-CoV-2-envelope-protein-induced platelet activation and thrombosis. Nat. Commun..

[707] De Moraes D., Paiva B.V.B., Cury S.S., Ludwig R.G., Junior J.P.A., Mori M.A.D.S., Carvalho R.F. (2021). Prediction of SARS-CoV Interaction with Host Proteins during Lung Aging Reveals a Potential Role for TRIB3 in COVID-19. Aging Dis..

[708] Navarese E.P., Podhajski P., Gurbel P.A., Grzelakowska K., Ruscio E., Tantry U., Magielski P., Kubica A., Niezgoda P., Adamski P. (2023). PCSK9 Inhibition During the Inflammatory Stage of SARS-CoV-2 Infection. J. Am. Coll. Cardiol..

[709] Meini S., Giani T., Tascini C. (2020). Intussusceptive angiogenesis in Covid-19: Hypothesis on the significance and focus on the possible role of FGF2. Mol. Biol. Rep..

[710] Chu J., Xing C., Du Y., Duan T., Liu S., Zhang P., Cheng C., Henley J., Liu X., Qian C. (2021). Pharmacological inhibition of fatty acid synthesis blocks SARS-CoV-2 replication. Nat. Metab..

[711] Grimaudo S., Amodio E., Pipitone R.M., Maida C.M., Pizzo S., Prestileo T., Tramuto F., Sardina D., Vitale F., Casuccio A. (2021). PNPLA3 and TLL-1 Polymorphisms as Potential Predictors of Disease Severity in Patients With COVID-19. Front. Cell Dev. Biol..

[712] Chi L., Shan Y., Cui Z. (2022). N-Acetyl-L-Cysteine Protects Airway Epithelial Cells during Respiratory Syncytial Virus Infection against Mucin Synthesis, Oxidative Stress, and Inflammatory Response and Inhibits HSPA6 Expression. Anal. Cell. Pathol..

[713] Youssef J.G., Bitar M.Z., Zahiruddin F., Al-Saadi M., Elshawwaf M., Yau S., Goodarzi A., Javitt J.C. (2022). Brief Report: Rapid Clinical Recovery from Critical Coronavirus Disease 2019 With Respiratory Failure in a Pregnant Patient Treated with IV Vasoactive Intestinal Peptide. Crit. Care Explor..

[714] Ahn N., Kim W.J., Kim N., Park H.W., Lee S.W., Yoo J.Y. (2019). The Interferon-Inducible Proteoglycan Testican-2/SPOCK2 Functions as a Protective Barrier against Virus Infection of Lung Epithelial Cells. J. Virol..

[715] Khalifa A.E., Ghoneim A.I. (2021). Potential value of pharmacological agents acting on toll-like receptor (TLR) 7 and/or TLR8 in COVID-19. Curr. Res. Pharmacol. Drug Discov..

[716] Ranjbar M., Rahimi A., Baghernejadan Z., Ghorbani A., Khorramdelazad H. (2022). Role of CCL2/CCR2 axis in the pathogenesis of COVID-19 and possible Treatments: All options on the Table. Int. Immunopharmacol..

[717] Schindewolf C., Lokugamage K., Vu M.N., Johnson B.A., Scharton D., Plante J.A., Kalveram B., Crocquet-Valdes P.A., Sotcheff S., Jaworski E. (2023). SARS-CoV-2 Uses Nonstructural Protein 16 To Evade Restriction by IFIT1 and IFIT3. J. Virol..

[718] Boal-Carvalho I., Mazel-Sanchez B., Silva F., Garnier L., Yildiz S., Bonifacio J.P., Niu C., Williams N., Francois P., Schwerk N. (2020). Influenza A viruses limit NLRP3-NEK7-complex formation and pyroptosis in human macrophages. EMBO Rep..

[719] Shirato K., Ujike M., Kawase M., Matsuyama S. (2015). Identification of CCL2, RARRES2 and EFNB2 as host cell factors that influence the multistep replication of respiratory syncytial virus. Virus Res..

[720] Szczepanski A., Owczarek K., Milewska A., Baster Z., Rajfur Z., Mitchell J.A., Pyrc K. (2018). Canine respiratory coronavirus employs caveolin-1-mediated pathway for internalization to HRT-18G cells. Vet. Res..

[721] Kisserli A., Schneider N., Audonnet S., Tabary T., Goury A., Cousson J., Mahmoudi R., Bani-Sadr F., Kanagaratnam L., Jolly D. (2021). Acquired decrease of the C3b/C4b receptor (CR1, CD35) and increased C4d deposits on erythrocytes from ICU COVID-19 patients. Immunobiology.

[722] Towne J.E., Harrod K.S., Krane C.M., Menon A.G. (2000). Decreased expression of aquaporin (AQP)1 and AQP5 in mouse lung after acute viral infection. Am. J. Respir. Cell Mol. Biol..

[723] Sato M., Ohtsuka K., Takahashi R., Wakabayashi K., Odai T., Isozaki T., Yajima N., Miwa Y., Kasama T. (2011). Involvement of CX3CL1/CX3CR1 axis in etanercept therapy for patients with active rheumatoid arthritis. Open Access Rheumatol..

[724] Roszkowski L., Jaszczyk B., Plebańczyk M., Ciechomska M. (2022). S100A8 and S100A12 Proteins as Biomarkers of High Disease Activity in Patients with Rheumatoid Arthritis That Can Be Regulated by Epigenetic Drugs. Int. J. Mol. Sci..

[725] Kaundal U., Khullar A., Leishangthem B., Jain S., Dhooria A., Saikia B., Dhir V. (2021). The effect of methotrexate on neutrophil reactive oxygen species and CD177 expression in rheumatoid arthritis. Clin. Exp. Rheumatol..

[726] Nguyen M.V.C., Baillet A., Romand X., Trocmé C., Courtier A., Marotte H., Thomas T., Soubrier M., Miossec P., Tébib J. (2019). Prealbumin, platelet factor 4 and S100A12 combination at baseline predicts good response to TNF alpha inhibitors in rheumatoid arthritis. Jt. Bone Spine.

[727] Wang W., Jian Z., Guo J., Ning X. (2014). Increased levels of serum myeloperoxidase in patients with active rheumatoid arthritis. Life Sci..

[728] Wu X.N., Gao Z.W., Yang L., Zhang J., Liu C., Zhang H.Z., Dong K. (2023). CD5L aggravates rheumatoid arthritis progression via promoting synovial fibroblasts proliferation and activity. Clin. Exp. Immunol..

[729] Blatt P.M., Yount W.J., Utsinger P.D., Korn J.H., Hadler N.M., Roberts H.R. (1977). Factor XI deficiency, juvenile rheumatoid arthritis and systemic lupus erythematosus. Report of the first case. Am. J. Med..

[730] Inciarte-Mundo J., Frade-Sosa B., Sanmartí R. (2022). From bench to bedside: Calprotectin (S100A8/S100A9) as a biomarker in rheumatoid arthritis. Front. Immunol..

[731] Luo Q., Li X., Zhang L., Yao F., Deng Z., Qing C., Su R., Xu J., Guo Y., Huang Z. (2019). Serum PGLYRP-1 is a highly discriminatory biomarker for the diagnosis of rheumatoid arthritis. Mol. Med. Rep..

[732] Cartwright A., Schmutz C., Askari A., Kuiper J.H., Middleton J. (2014). Orphan receptor GPR15/BOB is up-regulated in rheumatoid arthritis. Cytokine.

[733] Wada Y., Kuroda T., Murasawa A., Nakano M., Narita I. (2010). Anti-neutrophil cytoplasmic autoantibodies against bactericidal/permeability-increasing protein in patients with rheumatoid arthritis and their correlation with bronchial involvement. Mod. Rheumatol..

[734] Cai L., Lei C., Li R., Chen W.N., Hu C.M., Chen X.Y., Li C.M. (2017). Overexpression of aquaporin 4 in articular chondrocytes exacerbates the severity of adjuvant-induced arthritis in rats: An in vivo and in vitro study. J. Inflamm..

[735] Lai N.S., Yu H.C., Tseng H.Y., Hsu C.W., Huang H.B., Lu M.C. (2021). Increased Serum Levels of Brain-Derived Neurotrophic Factor Contribute to Inflammatory Responses in Patients with Rheumatoid Arthritis. Int. J. Mol. Sci..

[736] Pandya J.M., Lundell A.C., Andersson K., Nordström I., Theander E., Rudin A. (2017). Blood chemokine profile in untreated early rheumatoid arthritis: CXCL10 as a disease activity marker. Arthritis Res. Ther..

[737] Mahmoud I., Moalla M., Ben Tekaya A., Bouden S., Rouached L., Tekaya R., Saidane O., Gorji Y., Elleuch M., Laatar A. (2023). Impact of FCGR2A R131H, FCGR3A F158V and FCGR3B NA1/NA2 polymorphisms on response to Fc-containing TNF inhibitors in Tunisian rheumatoid arthritis patients. Drug Metab. Pers. Ther..

[738] Obry A., Lequerré T., Hardouin J., Boyer O., Fardellone P., Philippe P., Le Loët X., Cosette P., Vittecoq O. (2014). Identification of S100A9 as biomarker of responsiveness to the methotrexate/etanercept combination in rheumatoid arthritis using a proteomic approach. PLoS ONE.

[739] Rong H., He X., Wang L., Bai M., Jin T., Wang Y., Yang W., He Y., Yuan D. (2020). Association between IL1B polymorphisms and the risk of rheumatoid arthritis. Int. Immunopharmacol..

[740] Min S.H., Wang Y., Gonsiorek W., Anilkumar G., Kozlowski J., Lundell D., Fine J.S., Grant E.P. (2010). Pharmacological targeting reveals distinct roles for CXCR2/CXCR1 and CCR2 in a mouse model of arthritis. Biochem. Biophys. Res. Commun..

[741] Wang W., Deng Z., Liu G., Yang J., Zhou W., Zhang C., Shen W., Zhang Y. (2021). Platelet-derived extracellular vesicles promote the migration and invasion of rheumatoid arthritis fibroblast-like synoviocytes via CXCR2 signaling. Exp. Ther. Med..

[742] Ali Y., Khan S., Chen Y., Farooqi N., Islam Z.U., Akhtar M., Aamir, Aman A., Shah A.A., Jamal M. (2021). Association of AFF3 Gene Polymorphism rs10865035 with Rheumatoid Arthritis: A Population-Based Case-Control Study on a Pakistani Cohort. Genet. Res..

[743] Wu J., Fan W., Ma L., Geng X. (2018). miR-708-5p promotes fibroblast-like synoviocytes’ cell apoptosis and ameliorates rheumatoid arthritis by the inhibition of Wnt3a/β-catenin pathway. Drug Des. Dev. Ther..

[744] Gil-Quiñones S.R., Gutierrez-Castañeda L., Larios-Salazar L., Mejia-Mesa S., Motta A., Tovar-Parra D. (2022). Effect of Polymorphisms in the FCN1, FCN2, and FCN3 Genes on the Susceptibility to Develop Rheumatoid Arthritis: A Systematic Review. Int. J. Rheumatol..

[745] Na H.S., Kwon J.E., Lee S.H., Jhun J., Kim S.M., Kim S.Y., Kim E.K., Jung K., Park S.H., Cho M.L. (2017). Th17 and IL-17 Cause Acceleration of Inflammation and Fat Loss by Inducing α2-Glycoprotein 1 (AZGP1) in Rheumatoid Arthritis with High-Fat Diet. Am. J. Pathol..

[746] Gómez-Bañuelos E., Martín-Márquez B.T., Martínez-García E.A., Figueroa-Sanchez M., Nuñez-Atahualpa L., Rocha-Muñoz A.D., Sánchez-Hernández P.E., Navarro-Hernandez R.E., Madrigal-Ruiz P.M., Saldaña-Millan A.A. (2014). ow levels of CD36 in peripheral blood monocytes in subclinical atherosclerosis in rheumatoid arthritis: A cross-sectional study in a Mexican population. Biomed. Res. Int..

[747] Meng Y., Zheng X., Zhang Z., Geng H., Li X. (2023). Circulating PCSK9 relates to aggravated disease activity, Th17/Treg imbalance, and predicts treatment outcome of conventional synthetic DMARDs in rheumatoid arthritis patients. Ir. J. Med. Sci..

[748] Chen T., Zhou Z., Peng M., Hu H., Sun R., Xu J., Zhu C., Li Y., Zhang Q., Luo Y. (2023). Glutathione peroxidase 3 is a novel clinical diagnostic biomarker and potential therapeutic target for neutrophils in rheumatoid arthritis. Arthritis Res. Ther..

[749] Zhao S., Wang Y., Hou L., Wang Y., Xu N., Zhang N. (2020). Pentraxin 3 inhibits fibroblast growth factor 2 induced osteoclastogenesis in rheumatoid arthritis. Biomed. Pharmacother..

[750] Liu F., Feng X.X., Zhu S.L., Huang H.Y., Chen Y.D., Pan Y.F., June R.R., Zheng S.G., Huang J.L. (2018). Sonic Hedgehog Signaling Pathway Mediates Proliferation and Migration of Fibroblast-Like Synoviocytes in Rheumatoid Arthritis via MAPK/ERK Signaling Pathway. Front. Immunol..

[751] Lee H.R., Yoo S.J., Kim J., Kang S.W. (2023). LKB1 Regulates Inflammation of Fibroblast-like Synoviocytes from Patients with Rheumatoid Arthritis via AMPK-Dependent SLC7A11-NOX4-ROS Signaling. Cells.

[752] Villanueva-Romero R., Gutiérrez-Cañas I., Carrión M., Pérez-García S., Seoane I.V., Martínez C., Gomariz R.P., Juarranz Y. (2018). The Anti-Inflammatory Mediator, Vasoactive Intestinal Peptide, Modulates the Differentiation and Function of Th Subsets in Rheumatoid Arthritis. J. Immunol. Res..

[753] Ercan Z., Deniz G., Yentur S.B., Arikan F.B., Karatas A., Alkan G., Koca S.S. (2023). Effects of acute aerobic exercise on cytokines, klotho, irisin, and vascular endothelial growth factor responses in rheumatoid arthritis patients. Ir. J. Med. Sci..

[754] Öhman M., Öhman M.L., Wållberg-Jonsson S. (2014). The apoB/apoA1 ratio predicts future cardiovascular events in patients with rheumatoid arthritis. Scand. J. Rheumatol..

[755] Abreu J.R., de Launay D., Sanders M.E., Grabiec A.M., van de Sande M.G., Tak P.P., Reedquist K.A. (2009). The Ras guanine nucleotide exchange factor RasGRF1 promotes matrix metalloproteinase-3 production in rheumatoid arthritis synovial tissue. Arthritis Res. Ther..

[756] Suzuki A., Yamada R., Kochi Y., Sawada T., Okada Y., Matsuda K., Kamatani Y., Mori M., Shimane K., Hirabayashi Y. (2008). Functional SNPs in CD244 increase the risk of rheumatoid arthritis in a Japanese population. Nat. Genet..

[757] Laska M.J., Hansen B., Troldborg A., Lorenzen T., Stengaard-Pedersen K., Junker P., Nexø B.A., Lindegaard H.M. (2014). A non-synonymous single-nucleotide polymorphism in the gene encoding Toll-like Receptor 3 (TLR3) is associated with sero-negative rheumatoid arthritis (RA) in a Danish population. BMC Res. Notes.

[758] Zhang X., Nan H., Guo J., Liu J. (2021). NLRP12 reduces proliferation and inflammation of rheumatoid arthritis fibroblast-like synoviocytes by regulating the NF-κB and MAPK pathways. Eur. Cytokine Netw..

[759] Sun Y., Guo Y., Chang L., Zhang J. (2023). Long noncoding RNA H19 synergizes with STAT1 to regulate SNX10 in rheumatoid arthritis. Mol. Immunol..

[760] Sacre S., Lo A., Gregory B., Stephens M., Chamberlain G., Stott P., Brennan F. (2016). Oligodeoxynucleotide inhibition of Toll-like receptors 3, 7, 8, and 9 suppresses cytokine production in a human rheumatoid arthritis model. Eur. J. Immunol..

[761] Khadem Azarian S., Jafarnezhad-Ansariha F., Nazeri S., Azizi G., Aghazadeh Z., Hosseinzadeh E., Mirshafiey A. (2020). Effects of guluronic acid, as a new NSAID with immunomodulatory properties on IL-17, RORγt, IL-4 and GATA-3 gene expression in rheumatoid arthritis patients. Immunopharmacol. Immunotoxicol..

[762] Chen W., Fang Y., Wang H., Tan X., Zhu X., Xu Z., Jiang H., Wu X., Hong W., Wang X. (2023). Role of chemokine receptor 2 in rheumatoid arthritis: A research update. Int. Immunopharmacol..

[763] Ramos-González E.J., Bastian Y., Castañeda-Delgado J.E., Zapata-Zúñiga M., Gómez-Moreno M., Castillo-Ortiz J.D., Ramos-Remus C., Enciso-Moreno J.A. (2022). Overexpression of TLR7 and TLR9 Occurs Before Onset Symptoms in First-Degree Relatives of Rheumatoid Arthritis Patients. Arch. Med. Res..

[764] Galligan C.L., Matsuyama W., Matsukawa A., Mizuta H., Hodge D.R., Howard O.M., Yoshimura T. (2004). Up-regulated expression and activation of the orphan chemokine receptor, CCRL2, in rheumatoid arthritis. Arthritis Rheum..

[765] Hu Y., Wang X., Wu Y., Jin W., Cheng B., Fang X., Martel-Pelletier J., Kapoor M., Peng J., Qi S. (2015). Role of EFNB1 and EFNB2 in Mouse Collagen-Induced Arthritis and Human Rheumatoid Arthritis. Arthritis Rheumatol..

[766] Miao C.G., Shi W.J., Xiong Y.Y., Yu H., Zhang X.L., Qin M.S., Du C.L., Song T.W., Li J. (2015). miR-375 regulates the canonical Wnt pathway through FZD8 silencing in arthritis synovial fibroblasts. Immunol. Lett..

[767] Li S., Jin Z., Lu X. (2017). MicroRNA-192 suppresses cell proliferation and induces apoptosis in human rheumatoid arthritis fibroblast-like synoviocytes by downregulating caveolin 1. Mol. Cell. Biochem..

[768] Anand D., Kumar U., Kanjilal M., Kaur S., Das N. (2014). Leucocyte complement receptor 1 (CR1/CD35) transcript and its correlation with the clinical disease activity in rheumatoid arthritis patients. Clin. Exp. Immunol..

[769] Koca S.S., Etem E.O., Isik B., Yuce H., Ozgen M., Dag M.S., Isik A. (2010). Prevalence and significance of MEFV gene mutations in a cohort of patients with rheumatoid arthritis. Jt. Bone Spine.

[770] Chen H., Pan T., Liu P., Wang P., Xu S. (2019). Baihu Jia Guizhi Decoction Improves Rheumatoid Arthritis Inflammation by Regulating Succinate/SUCNR1 Metabolic Signaling Pathway. Evid. Based Complement. Altern. Med..

[771] Martínez A., Varadé J., Lamas J.R., Fernández-Arquero M., Jover J.A., de la Concha E.G., Fernández-Gutiérrez B., Urcelay E. (2008). Association of the IFIH1-GCA-KCNH7 chromosomal region with rheumatoid arthritis. Ann. Rheum. Dis..

[772] Lou Y., Zheng Y., Fan B., Zhang L., Zhu F., Wang X., Chen Z., Tan X. (2020). Serum S100A12 levels are correlated with clinical severity in patients with dermatomyositis-associated interstitial lung disease. J. Int. Med. Res..

[773] Kawai H., Kitagawa W., Suzuki N., Maeda K., Suzuki K., Miura N., Morita H., Banno S., Yamamura M., Imai H. (2011). Myeloperoxidase-antineutrophil cytoplasmic antibody-related crescentic glomerulonephritis after treatment for clinically amyopathic dermatomyositis: A coincidental combination or not?. Clin. Exp. Nephrol..

[774] Lou Y., Zheng Y., Fan B., Zhang L., Zhu F., Wang X., Chen Z., Tan X., Wei Q. (2020). Serum levels of interleukins and S100A8/A9 correlate with clinical severity in patients with dermatomyositis-associated interstitial lung disease. BMC Pulm. Med..

[775] Wienke J., Bellutti Enders F., Lim J., Mertens J.S., van den Hoogen L.L., Wijngaarde C.A., Yeo J.G., Meyer A., Otten H.G., Fritsch-Stork R.D.E. (2019). Galectin-9 and CXCL10 as Biomarkers for Disease Activity in Juvenile Dermatomyositis: A Longitudinal Cohort Study and Multicohort Validation. Arthritis Rheumatol..

[776] Ye J., Liu Q., Fu Q., Li B., Huang J., Zeng G. (2023). Tim-3, PD-1, CD244 and Foxp3 Positive T Cells’ Relation to the Prognosis of Dermatomyositis and Polymyositis Patients. J. Coll. Physicians Surg. Pak..

[777] Meyer A., Alsaleh G., Heuschling C., Gottenberg J.E., Georgel P., Geny B., Bahram S., Sibilia J. (2016). Dermatomyositis flare on imiquimod therapy highlights a crucial role of aberrant TLR7 signalling. RMD Open.

[778] Murakami T., Endo S., Moriki T., Doi T., Matsumoto Y. (2011). Mixed connective tissue disease developing into MPO-ANCA-positive polyangiitis. Intern. Med..

[779] Rothkrantz-Kos S., Drent M., Rutgers A., Heeringa P., De J., van Dieijen-Visser M.P., Cohen Tervaert J.W. (2003). Relationship between myeloperoxidase promotor polymorphism and disease severity in sarcoidosis. Eur. J. Intern. Med..

[780] Arger N.K., Ho M., Woodruff P.G., Koth L.L. (2019). Serum CXCL11 correlates with pulmonary outcomes and disease burden in sarcoidosis. Respir. Med..

[781] Grutters J.C., Sato H., Pantelidis P., Ruven H.J., McGrath D.S., Wells A.U., van den Bosch J.M., Welsh K.I., du Bois R.M. (2003). Analysis of IL6 and IL1A gene polymorphisms in UK and Dutch patients with sarcoidosis. Sarcoidosis Vasc. Diffuse Lung Dis..

[782] Arger N.K., Ho M.E., Allen I.E., Benn B.S., Woodruff P.G., Koth L.L. (2020). CXCL9 and CXCL10 are differentially associated with systemic organ involvement and pulmonary disease severity in sarcoidosis. Respir. Med..

[783] Typiak M., Rębała K., Dudziak M., Słomiński J.M., Dubaniewicz A. (2016). Polymorphism of FCGR2A, FCGR2C, and FCGR3B Genes in the Pathogenesis of Sarcoidosis. Adv. Exp. Med. Biol..

[784] Talreja J., Talwar H., Bauerfeld C., Grossman L.I., Zhang K., Tranchida P., Samavati L. (2019). HIF-1α regulates IL-1β and IL-17 in sarcoidosis. eLife..

[785] He J., Li X., Zhou J., Hu R. (2022). BATF2 and PDK4 as diagnostic molecular markers of sarcoidosis and their relationship with immune infiltration. Ann. Transl. Med..

[786] Prasse A., Zissel G., Lützen N., Schupp J., Schmiedlin R., Gonzalez-Rey E., Rensing-Ehl A., Bacher G., Cavalli V., Bevec D. (2010). Inhaled vasoactive intestinal peptide exerts immunoregulatory effects in sarcoidosis. Am. J. Respir. Crit. Care Med..

[787] Cooke G., Kamal I., Strengert M., Hams E., Mawhinney L., Tynan A., O’Reilly C., O’Dwyer D.N., Kunkel S.L., Knaus U.G. (2018). Toll-like receptor 3 L412F polymorphism promotes a persistent clinical phenotype in pulmonary sarcoidosis. QJM.

[788] Spagnolo P., Renzoni E.A., Wells A.U., Sato H., Grutters J.C., Sestini P., Abdallah A., Gramiccioni E., Ruven H.J., du Bois R.M. (2003). C-C chemokine receptor 2 and sarcoidosis: Association with Lofgren’s syndrome. Am. J. Respir. Crit. Care Med..

[789] Bordignon M., Bargagli E., Agostini C., Cinetto F., Baldo V., Alaibac M., Rottoli P. (2013). TLR7 Gln11Leu single nucleotide polymorphism in patients with sarcoidosis. Sarcoidosis Vasc. Diffuse Lung Dis..

[790] Zorzetto M., Bombieri C., Ferrarotti I., Medaglia S., Agostini C., Tinelli C., Malerba G., Carrabino N., Beretta A., Casali L. (2002). Complement receptor 1 gene polymorphisms in sarcoidosis. Am. J. Respir. Cell Mol. Biol..

[791] Omote A., Muramatsu M., Sugimoto Y., Hosono S., Murakami R., Tanaka H., Watanabe Y., Sano H., Kato K. (1997). Myeloperoxidase-specific anti-neutrophil cytoplasmic autoantibodies—Related scleroderma renal crisis treated with double-filtration plasmapheresis. Intern. Med..

[792] Giordano N., Volpi N., Franci D., Corallo C., Fioravanti A., Papakostas P., Montella A., Biagioli M., Fimiani M., Grasso G. (2012). Expression of RXFP1 in skin of scleroderma patients and control subjects. Scand. J. Rheumatol..

[793] Antinozzi C., Sgrò P., Marampon F., Caporossi D., Del Galdo F., Dimauro I., Di Luigi L. (2021). Sildenafil Counteracts the In Vitro Activation of CXCL-9, CXCL-10 and CXCL-11/CXCR3 Axis Induced by Reactive Oxygen Species in Scleroderma Fibroblasts. Biology.

[794] Morse J., Barst R., Horn E., Cuervo N., Deng Z., Knowles J. (2002). Pulmonary hypertension in scleroderma spectrum of disease: Lack of bone morphogenetic protein receptor 2 mutations. J. Rheumatol..

[795] Agarwal S.K., Wu M., Livingston C.K., Parks D.H., Mayes M.D., Arnett F.C., Tan F.K. (2011). Toll-like receptor 3 upregulation by type I interferon in healthy and scleroderma dermal fibroblasts. Arthritis Res. Ther..

[796] Ishikawa M., Yamamoto T. (2021). Antifibrogenic effects of C-C chemokine receptor type 2 antagonist in a bleomycin-induced scleroderma model. Exp. Dermatol..

[797] Liakouli V., Elies J., El-Sherbiny Y.M., Scarcia M., Grant G., Abignano G., Derrett-Smith E.C., Esteves F., Cipriani P., Emery P. (2018). Scleroderma fibroblasts suppress angiogenesis via TGF-β/caveolin-1 dependent secretion of pigment epithelium-derived factor. Ann. Rheum. Dis..

[798] Yan W., Chen C., Chen H. (2017). Estrogen Downregulates miR-21 Expression and Induces Inflammatory Infiltration of Macrophages in Polymyositis: Role of CXCL10. Mol. Neurobiol..

[799] Chourasia D., Achyut B.R., Tripathi S., Mittal B., Mittal R.D., Ghoshal U.C. (2009). Genotypic and functional roles of IL-1B and IL-1RN on the risk of gastroesophageal reflux disease: The presence of IL-1B-511*T/IL-1RN*1 (T1) haplotype may protect against the disease. Am. J. Gastroenterol..

[800] Isomoto H., Nishi Y., Kohno S. (2005). CXC receptor 1 is overexpressed in endoscopy-negative gastroesophageal reflux disease. Scand. J. Gastroenterol..

[801] Fabisiak A., Bartoszek A., Talar M., Binienda A., Dziedziczak K., Krajewska J.B., Mosińska P., Niewinna K., Tarasiuk A., Mokrowiecka A. (2020). Expression of FFAR3 and FFAR4 Is Increased in Gastroesophageal Reflux Disease. J. Clin. Med..

[802] Kassim S.K., El Touny M., El Guinaidy M., El Moghni M.A., El Mohsen A.A. (2002). Serum nitrates and vasoactive intestinal peptide in patients with gastroesophageal reflux disease. Clin. Biochem..

[803] Wright B.L., Nguyen N., Shim K.P., Masterson J.C., Jacobsen E.A., Ochkur S.I., Lee J.J., Furuta G.T. (2018). Increased GATA-3 and T-bet expression in eosinophilic esophagitis versus gastroesophageal reflux disease. J. Allergy Clin. Immunol..

[804] Lu Y. (2021). miR-223-5p Suppresses OTX1 to Mediate Malignant Progression of Lung Squamous Cell Carcinoma Cells. Comput. Math. Methods Med..

[805] Liu D., Ren H., Wen G., Xia P. (2023). Nicotine up-regulates SLC7A5 expression depending on TRIM29 in non-small cell lung cancer. Genes Dis..

[806] Wang W., Wang J., Liu S., Ren Y., Wang J., Liu S., Cui W., Jia L., Tang X., Yang J. (2022). An EHMT2/NFYA-ALDH2 signaling axis modulates the RAF pathway to regulate paclitaxel resistance in lung cancer. Mol. Cancer.

[807] Zhao J., Lan G. (2023). TFAP2A activates HMGA1 to promote glycolysis and lung adenocarcinoma progression. Pathol. Res. Pract..

[808] Cui R., Jiang N., Zhang M., Du S., Ou H., Ge R., Ma D., Zhang J. (2021). AMOTL2 inhibits JUN Thr239 dephosphorylation by binding PPP2R2A to suppress the proliferation in non-small cell lung cancer cells. Biochim. Biophys. Acta Mol. Cell Res..

[809] Zhao L., Zhang W., Luan F., Chen X., Wu H., He Q., Weng Q., Ding L., Yang B. (2023). Butein suppresses PD-L1 expression via downregulating STAT1 in non-small cell lung cancer. Biomed. Pharmacother..

[810] Assoun S., Theou-Anton N., Nguenang M., Cazes A., Danel C., Abbar B., Pluvy J., Gounant V., Khalil A., Namour C. (2019). Association of TP53 mutations with response and longer survival under immune checkpoint inhibitors in advanced non-small-cell lung cancer. Lung Cancer.

[811] Pereira E.E.B., Modesto A.A.C., Fernandes B.M., Burbano R.M.R., Assumpção P.P., Fernandes M.R., Guerreiro J.F., Santos S.E.B.D., Santos N.P.C.D. (2023). Association between Polymorphism of Genes IL-1A, NFKB1, PAR1, TP53, and UCP2 and Susceptibility to Non-Small Cell Lung Cancer in the Brazilian Amazon. Genes.

[812] Cao B., Liu M., Zhao Y., Gong C. (2022). Chronic oral mucocutaneous candidiasis, recurrent respiratory infection, hepatosplenomegaly, and autoimmune diabetes mellitus: A case report of a gain-of-function mutation of STAT1 in a Chinese boy. Front. Pediatr..

[813] Haghnazari L., Sabzi R. (2021). Relationship between TP53 and interleukin-6 gene variants and the risk of types 1 and 2 diabetes mellitus development in the Kermanshah province. J. Med. Life.

[814] Raza W., Guo J., Qadir M.I., Bai B., Muhammad S.A. (2022). qPCR Analysis Reveals Association of Differential Expression of SRR, NFKB1, and PDE4B Genes with Type 2 Diabetes Mellitus. Front. Endocrinol..

[815] Parvan R., Hosseinpour M., Moradi Y., Devaux Y., Cataliotti A., da Silva G.J.J. (2022). Diagnostic performance of microRNAs in the detection of heart failure with reduced or preserved ejection fraction: A systematic review and meta-analysis. Eur. J. Heart Fail..

[816] Bai R., Yang Q., Xi R., Li L., Shi D., Chen K. (2017). miR-941 as a promising biomarker for acute coronary syndrome. BMC Cardiovasc. Disord..

[817] Satoh T., Wang L., Espinosa-Diez C., Wang B., Hahn S.A., Noda K., Rochon E.R., Dent M.R., Levine A.R., Baust J.J. (2021). Metabolic Syndrome Mediates ROS-miR-193b-NFYA-Dependent Downregulation of Soluble Guanylate Cyclase and Contributes to Exercise-Induced Pulmonary Hypertension in Heart Failure with Preserved Ejection Fraction. Circulation.

[818] Wu L., Archacki S.R., Zhang T., Wang Q.K. (2007). Induction of high STAT1 expression in transgenic mice with LQTS and heart failure. Biochem. Biophys. Res. Commun..

[819] Qiao Q., Zhao C.M., Yang C.X., Gu J.N., Guo Y.H., Zhang M., Li R.G., Qiu X.B., Xu Y.J., Yang Y.Q. (2020). Detection and functional characterization of a novel MEF2A variation responsible for familial dilated cardiomyopathy. Clin. Chem. Lab. Med..

[820] Sano S., Wang Y., Ogawa H., Horitani K., Sano M., Polizio A.H., Kour A., Yura Y., Doviak H., Walsh K. (2021). TP53-mediated therapy-related clonal hematopoiesis contributes to doxorubicin-induced cardiomyopathy by augmenting a neutrophil-mediated cytotoxic response. JCI Insight.

[821] Luo J.Y., Liu F., Zhang T., Tian T., Luo F., Li X.M., Yang Y.N. (2022). Association of NFKB1 gene rs28362491 mutation with the occurrence of major adverse cardiovascular events. BMC Cardiovasc. Disord..

[822] Bernau K., Leet J.P., Bruhn E.M., Tubbs A.J., Zhu T., Sandbo N. (2021). Expression of serum response factor in the lung mesenchyme is essential for development of pulmonary fibrosis. Am. J. Physiol. Lung Cell. Mol. Physiol..

[823] Xiao T., Ren S., Bao J., Gao D., Sun R., Gu X., Gao J., Chen S., Jin J., Wei L. (2023). Vorapaxar proven to be a promising candidate for pulmonary fibrosis by intervening in the PAR1/JAK2/STAT1/3 signaling pathway-an experimental in vitro and vivo study. Eur. J. Pharmacol..

[824] Demopoulos K., Arvanitis D.A., Vassilakis D.A., Siafakas N.M., Spandidos D.A. (2002). MYCL1, FHIT, SPARC, p16(INK4) and TP53 genes associated to lung cancer in idiopathic pulmonary fibrosis. J. Cell. Mol. Med..

[825] Goropevšek A., Gorenjak M., Gradišnik S., Dai K., Holc I., Hojs R., Krajnc I., Pahor A., Avčin T. (2017). Increased Levels of STAT1 Protein in Blood CD4 T Cells from Systemic Lupus Erythematosus Patients Are Associated with Perturbed Homeostasis of Activated CD45RA-FOXP3hi Regulatory Subset and Follow-Up Disease Severity. J. Interferon Cytokine Res..

[826] Macedo J.M.B., Silva A.L., Pinto A.C., Landeira L.F.L., Portari E.A., Santos-Rebouças C.B., Klumb E.M. (2023). TP53 and p21 (CDKN1A) polymorphisms and the risk of systemic lupus erythematosus. Adv. Rheumatol..

[827] Nascimento D.Q., da Silva I.I.F.G., Lima C.A.D., Cavalcanti A.S., Roberti L.R., Queiroz R.G.P., Ferriani V.P.L., Crovella S., Carvalho L.M., Sandrin-Garcia P. (2022). Expression of the miR-9-5p, miR-125b-5p and its target gene NFKB1 and TRAF6 in childhood-onset systemic lupus erythematosus (cSLE). Autoimmunity.

[828] Aslani M., Mortazavi-Jahromi S.S., Mirshafiey A. (2021). Cytokine storm in the pathophysiology of COVID-19: Possible functional disturbances of miRNAs. Int. Immunopharmacol..

[829] Goud V.R., Chakraborty R., Chakraborty A., Lavudi K., Patnaik S., Sharma S., Patnaik S. (2022). A bioinformatic approach of targeting SARS-CoV-2 replication by silencing a conserved alternative reserve of the orf8 gene using host miRNAs. Comput. Biol. Med..

[830] Zhang Y., Mao D., Keeler S.P., Wang X., Wu K., Gerovac B.J., Shornick L.L., Agapov E.V., Holtzman M.J. (2019). Respiratory Enterovirus (like Parainfluenza Virus) Can Cause Chronic Lung Disease if Protection by Airway Epithelial STAT1 Is Lost. J. Immunol..

[831] Harford J.B., Kim S.S., Pirollo K.F., Chang E.H. (2022). TP53 Gene Therapy as a Potential Treatment for Patients with COVID-19. Viruses.

[832] Jandl K., Marsh L.M., Mutgan A.C., Crnkovic S., Valzano F., Zabini D., Hoffmann J., Foris V., Gschwandtner E., Klepetko W. (2022). Impairment of the NKT-STAT1-CXCL9 Axis Contributes to Vessel Fibrosis in Pulmonary Hypertension Caused by Lung Fibrosis. Am. J. Respir. Crit. Care Med..

[833] Hennigs J.K., Cao A., Li C.G., Shi M., Mienert J., Miyagawa K., Körbelin J., Marciano D.P., Chen P.I., Roughley M. (2021). PPARγ-p53-Mediated Vasculoregenerative Program to Reverse Pulmonary Hypertension. Circ. Res..

[834] Cox A.R., Chernis N., Bader D.A., Saha P.K., Masschelin P.M., Felix J.B., Sharp R., Lian Z., Putluri V., Rajapakshe K. (2020). STAT1 Dissociates Adipose Tissue Inflammation From Insulin Sensitivity in Obesity. Diabetes.

[835] Montazeri-Najafabady N., Dabbaghmanesh M.H., Nasimi N., Sohrabi Z., Chatrabnous N. (2021). The association between TP53 rs1625895 polymorphism and the risk of sarcopenic obesity in Iranian older adults: A case-control study. BMC Musculoskelet. Disord..

[836] Yenmis G., Soydas T., Arkan H., Tasan E., Kanigur Sultuybek G. (2018). Genetic Variation in NFKB1 Gene Influences Liver Enzyme Levels in Morbidly Obese Women. Arch. Iran. Med..

[837] Bersimbaev R., Aripova A., Bulgakova O., Kussainova A., Akparova A., Izzotti A. (2021). The Plasma Levels of hsa-miR-19b-3p, hsa-miR-125b-5p, and hsamiR- 320c in Patients with Asthma, COPD and Asthma-COPD Overlap Syndrome (ACOS). Microrna.

[838] Lewis A., Riddoch-Contreras J., Natanek S.A., Donaldson A., Man W.D., Moxham J., Hopkinson N.S., Polkey M.I., Kemp P.R. (2012). Downregulation of the serum response factor/miR-1 axis in the quadriceps of patients with COPD. Thorax.

[839] Gu C., Li Y., Liu J., Ying X., Liu Y., Yan J., Chen C., Zhou H., Cao L., Ma Y. (2017). LncRNA-mediated SIRT1/FoxO3a and SIRT1/p53 signaling pathways regulate type II alveolar epithelial cell senescence in patients with chronic obstructive pulmonary disease. Mol. Med. Rep..

[840] Lewis B.W., Jackson D., Amici S.A., Walum J., Guessas M., Guessas S., Coneglio E., Boda A.V., Guerau-de-Arellano M., Grayson M.H. (2021). Corticosteroid insensitivity persists in the absence of STAT1 signaling in severe allergic airway inflammation. Am. J. Physiol. Lung Cell. Mol. Physiol..

[841] Zhao J., Pu J., Hao B., Huang L., Chen J., Hong W., Zhou Y., Li B., Ran P. (2020). LncRNA RP11-86H7.1 promotes airway inflammation induced by TRAPM2.5 by acting as a ceRNA of miRNA-9-5p to regulate NFKB1 in HBECS. Sci. Rep..

[842] Malaab M., Renaud L., Takamura N., Zimmerman K.D., da Silveira W.A., Ramos P.S., Haddad S., Peters-Golden M., Penke L.R., Wolf B. (2022). Antifibrotic factor KLF4 is repressed by the miR-10/TFAP2A/TBX5 axis in dermal fibroblasts: Insights from twins discordant for systemic sclerosis. Ann. Rheum. Dis..

[843] Zmorzyński S., Wojcierowska-Litwin M., Kowal M., Michalska-Jakubus M., Styk W., Filip A.A., Walecka I., Krasowska D. (2020). NOTCH3 T6746C and TP53 P72R Polymorphisms Are Associated with the Susceptibility to Diffuse Cutaneous Systemic Sclerosis. Biomed. Res. Int..

[844] Liu C., Yan S., Chen H., Wu Z., Li L., Cheng L., Li H., Li Y. (2021). Association of GTF2I, NFKB1, and TYK2 Regional Polymorphisms with Systemic Sclerosis in a Chinese Han Population. Front. Immunol..

[845] Ding D., Zhang Q., Zeng F.J., Cai M.X., Gan Y., Dong X.J. (2023). Mechanism of Gentisic Acid on Rheumatoid Arthritis Based on miR-19b-3p/RAF1 Axis. Chin. J. Integr. Med..

[846] Huber R., Augsten S., Kirsten H., Zell R., Stelzner A., Thude H., Eidner T., Stuhlmüller B., Ahnert P., Kinne R.W. (2020). Identification of New, Functionally Relevant Mutations in the Coding Regions of the Human Fos and Jun Proto-Oncogenes in Rheumatoid Arthritis Synovial Tissue. Life.

[847] Tucci G., Garufi C., Pacella I., Zagaglioni M., Pinzon Grimaldos A., Ceccarelli F., Conti F., Spinelli F.R., Piconese S. (2022). Baricitinib therapy response in rheumatoid arthritis patients associates to STAT1 phosphorylation in monocytes. Front. Immunol..

[848] Gansmo L.B., Lie B.A., Mæhlen M.T., Vatten L., Romundstad P., Hveem K., Lønning P.E., Knappskog S. (2021). Polymorphisms in the TP53-MDM2-MDM4-axis in patients with rheumatoid arthritis. Gene.

[849] Elkhawaga S.Y., Gomaa M.H., Elsayed M.M., Ebeed A.A. (2021). NFKB1 promoter–94 insertion/deletion ATTG polymorphism (rs28362491) is associated with severity and disease progression of rheumatoid arthritis through interleukin-6 levels modulation in Egyptian patients. Clin Rheumatol..

[850] Rosenbaum J.T., Pasadhika S., Crouser E.D., Choi D., Harrington C.A., Lewis J.A., Austin C.R., Diebel T.N., Vance E.E., Braziel R.M. (2009). Hypothesis: Sarcoidosis is a STAT1-mediated disease. Clin. Immunol..

[851] Gallardo E., de Andrés I., Illa I. (2001). Cathepsins are upregulated by IFN-gamma/STAT1 in human muscle culture: A possible active factor in dermatomyositis. J. Neuropathol. Exp. Neurol..

[852] Sgalla G., Iovene B., Calvello M., Ori M., Varone F., Richeldi L. (2018). Idiopathic pulmonary fibrosis: Pathogenesis and management. Respir. Res..

