# Supplementary material for: Study on Potential Differentially Expressed Genes in Idiopathic Pulmonary Fibrosis by Bioinformatics and Next-Generation Sequencing Data Analysis
Source: Biomedicines. 2023 Nov 21;11(12):3109. doi: 10.3390/biomedicines11123109 (PMC10740779; doi:10.3390/biomedicines11123109)
Supplement: Supplementary file 1 [file biomedicines-11-03109-s001.zip › biomedicines-2653971-supplementary.pdf]

Table S1 The statistical metrics for key differentially expressed genes (DEGs).

Gene Symbol	logFC	pValue	adj.P.Val	tvalue	Regulation	Gene Name
SLCO1A2	3.726667	2.10E-11	3.29E-09	6.699037	Up	solute carrier organic anion transporter family member 1A2
OLFM4	2.443401	2.03E-08	8.58E-07	5.609154	Up	olfactomedin 4
DEFA4	2.288422	5.11E-06	8.17E-05	4.560317	Up	defensin alpha 4
NECAB1	2.277329	2.62E-14	1.41E-11	7.615939	Up	N-terminal EF-hand calcium binding protein 1
ITLN2	2.233514	3.34E-08	1.27E-06	5.522634	Up	intelectin 2
RTKN2	2.202055	9.80E-12	1.82E-09	6.809355	Up	rhotekin 2
CYP1A1	1.958708	4.84E-07	1.18E-05	5.032612	Up	cytochrome P450 family 1 subfamily A member 1
HHIP	1.861403	4.50E-12	9.54E-10	6.920567	Up	hedgehog interacting protein
AGTR2	1.838389	7.27E-06	0.000109	4.485482	Up	angiotensin II receptor type 2
CHI3L2	1.70625	1.37E-10	1.45E-08	6.419584	Up	chitinase 3 like 2
MAP3K15	1.68578	1.27E-07	3.94E-06	5.282487	Up	mitogen-activated protein kinase kinase kinase 15
ORM2	1.670216	8.37E-05	0.000773	3.933642	Up	orosomucoid 2
PRTN3	1.66482	2.87E-05	0.000321	4.183191	Up	proteinase 3
WFDC12	1.653406	0.000256	0.001849	3.656259	Up	WAP four-disulfide core domain 12
DEFA3	1.623825	0.00012	0.001035	3.846044	Up	defensin alpha 3
GALNT13	1.609443	5.25E-07	1.27E-05	5.016917	Up	polypeptide N-acetylgalactosaminyltransferase 13
RNASE3	1.551794	4.13E-06	6.91E-05	4.604856	Up	ribonuclease A family member 3
SCN1A	1.545567	3.63E-08	1.35E-06	5.508046	Up	sodium voltage-gated channel alpha subunit 1
HSD17B6	1.544643	3.23E-11	4.62E-09	6.635692	Up	hydroxysteroid 17-beta dehydrogenase 6
PI3	1.53004	1.67E-05	0.000209	4.304671	Up	peptidase inhibitor 3
CX3CR1	1.520273	5.68E-09	2.92E-07	5.826041	Up	C-X3-C motif chemokine receptor 1
S100A12	1.511955	5.94E-06	9.20E-05	4.528661	Up	S100 calcium binding protein A12
TUBB1	1.501628	1.23E-05	0.000164	4.372072	Up	tubulin beta 1 class VI
CRTAC1	1.498618	9.52E-08	3.10E-06	5.335693	Up	cartilage acidic protein 1
PROK2	1.49785	3.02E-06	5.40E-05	4.66956	Up	prokineticin 2
CSF2	1.487492	2.80E-05	0.000314	4.189412	Up	colony stimulating factor 2
ALPP	1.479287	0.000107	0.000941	3.874387	Up	alkaline phosphatase, placental
CD177	1.469469	1.92E-05	0.000232	4.273978	Up	CD177 molecule
ANKRD1	1.466629	0.000406	0.002657	3.536161	Up	ankyrin repeat domain 1
CADM2	1.460814	4.48E-06	7.41E-05	4.587788	Up	cell adhesion molecule 2
AGRP	1.459043	2.50E-06	4.63E-05	4.707972	Up	agouti related neuropeptide
SLC39A8	1.428325	1.79E-10	1.75E-08	6.378507	Up	solute carrier family 39 member 8
ADRA1A	1.412386	4.55E-08	1.65E-06	5.468233	Up	adrenoceptor alpha 1A
MS4A3	1.407997	2.99E-05	0.000331	4.174463	Up	membrane spanning 4-domains A3
MS4A15	1.407407	5.67E-06	8.89E-05	4.538403	Up	membrane spanning 4-domains A15
ADCY8	1.403596	0.000467	0.00296	3.498719	Up	adenylate cyclase 8
GGTLC1	1.398003	3.88E-06	6.61E-05	4.617923	Up	gamma-glutamyltransferase light chain 1
TRIM58	1.378761	1.32E-07	4.02E-06	5.276636	Up	tripartite motif containing 58
FGG	1.367873	0.002424	0.010844	3.032716	Up	fibrinogen gamma chain
ZDHHC19	1.367468	2.35E-06	4.41E-05	4.720461	Up	zinc finger DHHC-type palmitoyltransferase 19

AGER	1.35608	1.29E-05	0.00017	4.362054	Up	advanced glycosylation end-product specific receptor
LHX9	1.348205	0.000346	0.002357	3.578126	Up	LIM homeobox 9
PF4	1.34113	0.000361	0.002434	3.566921	Up	platelet factor 4
FFAR2	1.340923	2.23E-05	0.000261	4.24086	Up	free fatty acid receptor 2
SERTM1	1.326803	0.000187	0.001436	3.736505	Up	serine rich and transmembrane domain containing 1
MGAM2	1.325326	6.78E-05	0.000651	3.983855	Up	maltase-glucoamylase 2 (putative)
ORM1	1.305666	0.000245	0.001782	3.667932	Up	orosomucoid 1
GP1BA	1.298681	3.29E-06	5.78E-05	4.651848	Up	glycoprotein Ib platelet subunit alpha
MPO	1.272553	1.98E-06	3.85E-05	4.755188	Up	myeloperoxidase
CD5L	1.268611	1.46E-05	0.000188	4.333999	Up	CD5 molecule like
SLCO4C1	1.252787	3.60E-06	6.23E-05	4.633043	Up	solute carrier organic anion transporter family member 4C1
GATD3	1.246426	0.011314	0.036418	2.532863	Up	glutamine amidotransferase class 1 domain containing 3
SH3GL2	1.235838	1.59E-05	0.000201	4.315327	Up	SH3 domain containing GRB2 like 2, endophilin A1
ADGRE1	1.233669	9.10E-07	2.00E-05	4.91025	Up	adhesion G protein-coupled receptor E1
RS1	1.223036	0.000866	0.004794	3.330696	Up	retinoschisin 1
HMGCS2	1.206207	0.012077	0.038444	2.509883	Up	3-hydroxy-3-methylglutaryl-CoA synthase 2
SLC5A9	1.189442	5.23E-05	0.000522	4.045058	Up	solute carrier family 5 member 9
RXFP1	1.188223	1.01E-06	2.19E-05	4.88954	Up	relaxin family peptide receptor 1
ITGA2B	1.183783	3.83E-07	9.67E-06	5.077144	Up	integrin subunit alpha 2b
SYT15B	1.183499	4.03E-08	1.49E-06	5.489494	Up	synaptotagmin 15B
F11	1.175516	0.00059	0.003553	3.436074	Up	coagulation factor XI
S100A8	1.175054	2.05E-05	0.000245	4.25894	Up	S100 calcium binding protein A8
SFTPA2	1.174217	3.72E-05	0.000399	4.123997	Up	surfactant protein A2
CHRM1	1.168264	0.000602	0.003611	3.430529	Up	cholinergic receptor muscarinic 1
FAM167A	1.165267	5.47E-07	1.29E-05	5.008966	Up	family with sequence similarity 167 member A
PGLYRP1	1.158301	0.000848	0.004718	3.33673	Up	peptidoglycan recognition protein 1
BRINP1	1.157452	3.24E-05	0.000355	4.155749	Up	BMP/retinoic acid inducible neural specific 1
SNX22	1.157308	8.71E-06	0.000126	4.446869	Up	sorting nexin 22
ZBED2	1.155994	1.64E-05	0.000205	4.309611	Up	zinc finger BED-type containing 2
GPM6A	1.149957	2.76E-08	1.09E-06	5.556184	Up	glycoprotein M6A
GRIA1	1.141825	1.19E-05	0.00016	4.378714	Up	glutamate ionotropic receptor AMPA type subunit 1
NPR3	1.121107	3.51E-08	1.32E-06	5.513641	Up	natriuretic peptide receptor 3
RBP2	1.120952	0.000784	0.004435	3.358291	Up	retinol binding protein 2
MUCL3	1.111579	0.001469	0.00731	3.180675	Up	mucin like 3
VEGFD	1.096482	1.01E-06	2.18E-05	4.890571	Up	vascular endothelial growth factor D
CHRM2	1.096233	0.001935	0.009123	3.100067	Up	cholinergic receptor muscarinic 2
MPIG6B	1.086753	2.76E-06	5.04E-05	4.688238	Up	megakaryocyte and platelet inhibitory receptor G6b
CXCL11	1.085868	0.00068	0.003959	3.397455	Up	C-X-C motif chemokine ligand 11
LAMP3	1.085091	3.73E-05	0.000399	4.123524	Up	lysosomal associated membrane protein 3
GPR15	1.080741	0.002209	0.010136	3.06053	Up	G protein-coupled receptor 15
SHISA2	1.073802	1.31E-07	4.01E-06	5.277387	Up	shisa family member 2
KCTD16	1.070257	2.33E-07	6.30E-06	5.17049	Up	potassium channel tetramerization domain containing 16
KLRG2	1.069203	0.000321	0.002218	3.597377	Up	killer cell lectin like receptor G2

IL1A	1.064859	0.00019	0.001456	3.732112	Up	interleukin 1 alpha
LILRA1	1.053918	1.80E-07	5.17E-06	5.218657	Up	leukocyte immunoglobulin like receptor A1
ND6	1.049081	7.88E-08	2.63E-06	5.3698	Up	NADH dehydrogenase subunit 6
CBS	1.048011	3.71E-05	0.000398	4.125074	Up	cystathionine beta-synthase
CLDN18	1.047537	0.002946	0.012634	2.973298	Up	claudin 18
WNT7A	1.046829	9.19E-06	0.00013	4.435451	Up	Wnt family member 7A
NPFFR1	1.04173	5.36E-06	8.47E-05	4.550296	Up	neuropeptide FF receptor 1
NFE2	1.035791	1.64E-05	0.000205	4.308769	Up	nuclear factor, erythroid 2
SFTPA1	1.033603	0.000353	0.00239	3.57294	Up	surfactant protein A1
BPI	1.031272	0.000371	0.002471	3.560063	Up	bactericidal permeability increasing protein
BTNL8	1.030299	0.000103	0.000911	3.883508	Up	butyrophilin like 8
SSTR1	1.027973	4.49E-06	7.41E-05	4.587376	Up	somatostatin receptor 1
PRKG2	1.027838	1.25E-06	2.63E-05	4.847774	Up	protein kinase cGMP-dependent 2
AQP4	1.022337	1.03E-05	0.000142	4.410583	Up	aquaporin 4
USP44	1.022328	5.09E-08	1.82E-06	5.448026	Up	ubiquitin specific peptidase 44
PLA2G4E	1.014528	0.003251	0.013626	2.942972	Up	phospholipase A2 group IVE
FOLR3	1.013648	0.000452	0.00289	3.507673	Up	folate receptor gamma
SCD	1.01282	1.52E-05	0.000194	4.326413	Up	stearoyl-CoA desaturase
SLC19A3	1.010289	7.10E-07	1.62E-05	4.958671	Up	solute carrier family 19 member 3
LRP2	1.006373	0.000129	0.001095	3.828211	Up	LDL receptor related protein 2
TRIM71	1.005932	1.09E-05	0.000149	4.39922	Up	tripartite motif containing 71
SLC6A4	1.005045	0.015722	0.04737	2.415306	Up	solute carrier family 6 member 4
MME	1.003156	4.50E-06	7.41E-05	4.586762	Up	membrane metalloendopeptidase
BDNF	1.001912	9.01E-06	0.000128	4.439743	Up	brain derived neurotrophic factor
ZNF385B	0.995591	1.91E-05	0.000232	4.274887	Up	zinc finger protein 385B
RNF182	0.995496	3.10E-09	1.81E-07	5.926288	Up	ring finger protein 182
MYRF	0.974509	0.000297	0.002093	3.61779	Up	myelin regulatory factor
SEC14L4	0.974443	0.000178	0.001392	3.747686	Up	SEC14 like lipid binding 4
NRGN	0.964971	2.20E-08	9.13E-07	5.595451	Up	neurogranin
GCOM1	0.964481	1.87E-05	0.000227	4.280406	Up	GCOM1, MYZAP-POLR2M combined locus
GFI1B	0.96123	0.000201	0.001523	3.718067	Up	growth factor independent 1B transcriptional repressor
PEBP4	0.959036	0.000586	0.003539	3.437835	Up	phosphatidylethanolamine binding protein 4
LRRN3	0.956791	6.85E-07	1.57E-05	4.965673	Up	leucine rich repeat neuronal 3
CXCL10	0.956734	0.001413	0.007093	3.191992	Up	C-X-C motif chemokine ligand 10
ODAM	0.953708	0.003364	0.014006	2.932395	Up	odontogenic, ameloblast associated
CPB2	0.948363	0.014321	0.044012	2.449109	Up	carboxypeptidase B2
RPH3A	0.945528	0.001046	0.005552	3.277886	Up	rabphilin 3A
KCNS1	0.937449	0.000977	0.005265	3.297088	Up	potassium voltage-gated channel modifier subfamily S member 1
ANGPTL7	0.93254	0.000471	0.002977	3.496537	Up	angiopoietin like 7
ASPG	0.93217	0.001952	0.009174	3.097393	Up	asparaginase
TMEM97	0.931267	1.29E-07	3.98E-06	5.279651	Up	transmembrane protein 97
PTCRA	0.929636	8.68E-05	0.000797	3.924732	Up	pre T cell antigen receptor alpha
CASP5	0.928596	0.000171	0.001348	3.759014	Up	caspase 5

RIMKLA	0.924486	5.09E-05	0.00051	4.051534	Up	ribosomal modification protein rimK like family member A
LGI3	0.917561	0.003541	0.014568	2.916379	Up	leucine rich repeat LGI family member 3
CCL8	0.906363	0.000972	0.005248	3.298642	Up	C-C motif chemokine ligand 8
PRODH	0.901718	0.006419	0.023233	2.725598	Up	proline dehydrogenase 1
CLTRN	0.90112	8.72E-06	0.000126	4.446726	Up	collectrin, amino acid transport regulator
TMEM100	0.897363	0.000682	0.003969	3.396709	Up	transmembrane protein 100
P2RY1	0.897328	3.98E-08	1.48E-06	5.491945	Up	purinergic receptor P2Y1
RNASE2	0.896564	0.000158	0.001275	3.777498	Up	ribonuclease A family member 2
CSPG5	0.896384	0.00025	0.001813	3.662563	Up	chondroitin sulfate proteoglycan 5
LRRTM2	0.892735	9.00E-05	0.000822	3.916088	Up	leucine rich repeat transmembrane neuronal 2
GPRIN2	0.884966	0.000122	0.001046	3.842564	Up	G protein regulated inducer of neurite outgrowth 2
LRRN4	0.882641	0.0008	0.004506	3.352731	Up	leucine rich repeat neuronal 4
VEPH1	0.881795	0.000354	0.002394	3.572108	Up	ventricular zone expressed PH domain containing 1
SIGLEC11	0.879986	7.36E-06	0.00011	4.483037	Up	sialic acid binding Ig like lectin 11
FCGR3B	0.87501	0.000743	0.004256	3.373345	Up	Fc gamma receptor IIIb
S100A9	0.874918	0.000228	0.001691	3.685291	Up	S100 calcium binding protein A9
NPY1R	0.874152	0.000165	0.001315	3.766824	Up	neuropeptide Y receptor Y1
MARCKS	0.872521	5.59E-17	2.10E-13	8.373601	Up	myristoylated alanine rich protein kinase C substrate
MSMO1	0.867924	6.55E-07	1.51E-05	4.974255	Up	methylsterol monooxygenase 1
IL1B	0.867653	0.001451	0.007253	3.184213	Up	interleukin 1 beta
CXCR1	0.858859	0.00228	0.010385	3.051141	Up	C-X-C motif chemokine receptor 1
PLPPR1	0.856766	0.000128	0.00109	3.829712	Up	phospholipid phosphatase related 1
CXCR2	0.854949	0.000829	0.004647	3.342863	Up	C-X-C motif chemokine receptor 2
GPIHBP1	0.853387	0.000977	0.005265	3.297183	Up	glycosylphosphatidylinositol anchored high density lipoprotein binding protein 1
SAMD5	0.852839	7.39E-09	3.68E-07	5.781753	Up	sterile alpha motif domain containing 5
AFF3	0.851292	2.04E-05	0.000244	4.260257	Up	ALF transcription elongation factor 3
PCDH9	0.847357	2.07E-06	3.98E-05	4.746011	Up	protocadherin 9
TNNT1	0.844116	0.005172	0.019606	2.796128	Up	troponin T1, slow skeletal type
WNT3A	0.837416	0.000623	0.003703	3.421259	Up	Wnt family member 3A
PARD6B	0.834916	4.50E-06	7.41E-05	4.58678	Up	par-6 family cell polarity regulator beta
TMED7	0.834798	4.69E-12	9.73E-10	6.914562	Up	transmembrane p24 trafficking protein 7
SP5	0.833924	0.000174	0.001364	3.754474	Up	Sp5 transcription factor
CA4	0.830295	0.008543	0.029084	2.62983	Up	carbonic anhydrase 4
APOH	0.827213	0.009119	0.030575	2.607569	Up	apolipoprotein H
SGCG	0.826508	0.000163	0.001296	3.771067	Up	sarcoglycan gamma
FZD5	0.826035	1.52E-06	3.11E-05	4.808676	Up	frizzled class receptor 5
CHRM3	0.820024	9.85E-06	0.000138	4.420411	Up	cholinergic receptor muscarinic 3
ACADL	0.81846	0.000177	0.001382	3.750386	Up	acyl-CoA dehydrogenase long chain
CTNND2	0.816462	0.000811	0.00456	3.349034	Up	catenin delta 2
BMI1	0.812293	1.17E-13	4.40E-11	7.420272	Up	BMI1 proto-oncogene, polycomb ring finger
PCARE	0.810876	0.003483	0.014384	2.921528	Up	photoreceptor cilium actin regulator
PRSS21	0.803212	0.000806	0.004536	3.350688	Up	serine protease 21

CYP1B1	0.798853	3.85E-05	0.000409	4.116276	Up	cytochrome P450 family 1 subfamily B member 1
FCN3	0.798379	0.00769	0.026769	2.665369	Up	ficolin 3
TTN	0.797279	0.001445	0.00723	3.185423	Up	titin
NIM1K	0.795122	1.40E-05	0.000182	4.343551	Up	NIM1 serine/threonine protein kinase
SHISA3	0.794638	0.000456	0.002911	3.505181	Up	shisa family member 3
AZGP1	0.794084	0.012721	0.039943	2.491478	Up	alpha-2-glycoprotein 1, zinc-binding
RIPPLY3	0.791581	0.002202	0.010113	3.061555	Up	ripply transcriptional repressor 3
GPAM	0.790433	4.27E-08	1.57E-06	5.479205	Up	glycerol-3-phosphate acyltransferase, mitochondrial
STC1	0.789493	0.003952	0.015837	2.881995	Up	stanniocalcin 1
ABCA3	0.789374	0.002934	0.012599	2.974596	Up	ATP binding cassette subfamily A member 3
ST8SIA6	0.789286	1.01E-05	0.00014	4.415074	Up	ST8 alpha-N-acetyl-neuraminide alpha-2,8-sialyltransferase 6
ELOVL3	0.788495	9.47E-07	2.06E-05	4.902332	Up	ELOVL fatty acid elongase 3
GBP4	0.788416	7.56E-06	0.000112	4.477412	Up	guanylate binding protein 4
CD36	0.788298	5.85E-06	9.09E-05	4.531857	Up	CD36 molecule
HMGCS1	0.783582	5.48E-08	1.92E-06	5.435071	Up	3-hydroxy-3-methylglutaryl-CoA synthase 1
CACNA2D3	0.781809	5.27E-06	8.38E-05	4.553623	Up	calcium voltage-gated channel auxiliary subunit alpha2delta 3
CACNA2D2	0.781549	0.001999	0.009341	3.090379	Up	calcium voltage-gated channel auxiliary subunit alpha2delta 2
VSIG10	0.78052	1.26E-08	5.79E-07	5.691425	Up	V-set and immunoglobulin domain containing 10
EDNRB	0.778024	1.06E-07	3.40E-06	5.315695	Up	endothelin receptor type B
MYZAP	0.77616	0.000496	0.003094	3.482787	Up	myocardial zonula adherens protein
MGAM	0.775981	0.009242	0.030894	2.602952	Up	maltase-glucoamylase
MACROD2	0.775908	8.75E-05	0.000801	3.922996	Up	mono-ADP ribosylhydrolase 2
PLA2G3	0.773985	0.002128	0.009845	3.071696	Up	phospholipase A2 group III
BTNL9	0.772766	0.001294	0.006586	3.217415	Up	butyrophilin like 9
CEBPA	0.771134	2.38E-06	4.44E-05	4.717987	Up	CCAAT enhancer binding protein alpha
C2orf88	0.770338	5.14E-06	8.20E-05	4.558987	Up	chromosome 2 open reading frame 88
TRIB3	0.767008	7.54E-06	0.000112	4.477863	Up	tribbles pseudokinase 3
GPR18	0.765001	0.000238	0.001745	3.674611	Up	G protein-coupled receptor 18
SLC46A2	0.76484	0.005188	0.019659	2.795092	Up	solute carrier family 46 member 2
CNTN6	0.76271	0.001003	0.005367	3.289799	Up	contactin 6
FMO5	0.758959	0.001646	0.00801	3.147692	Up	flavin containing dimethylaniline monoxygenase 5
TNNC1	0.755531	0.002398	0.010762	3.035904	Up	troponin C1, slow skeletal and cardiac type
ANGPT4	0.751626	0.000467	0.002958	3.499095	Up	angiopoietin 4
FAM167B	0.750142	1.85E-05	0.000226	4.281845	Up	family with sequence similarity 167 member B
SPX	0.748505	3.79E-05	0.000404	4.120018	Up	spexin hormone
MAP3K21	0.746867	2.10E-07	5.83E-06	5.190158	Up	mitogen-activated protein kinase kinase kinase 21
SYNPO2L	0.746552	0.00651	0.023479	2.720908	Up	synaptopodin 2 like
ATP8A1	0.744997	2.61E-14	1.41E-11	7.616369	Up	ATPase phospholipid transporting 8A1
CCDC141	0.744633	0.000609	0.003639	3.427564	Up	coiled-coil domain containing 141
TPD52	0.741541	8.42E-11	9.90E-09	6.492983	Up	tumor protein D52
PCSK9	0.740276	0.005099	0.019383	2.800715	Up	proprotein convertase subtilisin/kexin type 9
ACVR1C	0.740103	0.000204	0.001541	3.713882	Up	activin A receptor type 1C
SYT15	0.739694	2.43E-05	0.00028	4.221096	Up	synaptotagmin 15

TDRD9	0.737613	0.000126	0.001075	3.834021	Up	tudor domain containing 9
RNF128	0.736962	9.84E-05	0.000878	3.894482	Up	ring finger protein 128
SH2D1B	0.736718	0.002137	0.00987	3.070481	Up	SH2 domain containing 1B
PRTG	0.734237	3.47E-09	2.01E-07	5.907804	Up	protogenin
P2RY13	0.727834	5.32E-06	8.44E-05	4.551785	Up	purinergic receptor P2Y13
IER3IP1	0.726911	1.44E-13	5.27E-11	7.393027	Up	immediate early response 3 interacting protein 1
CDKL2	0.724664	0.000348	0.002365	3.576733	Up	cyclin dependent kinase like 2
KITLG	0.72459	3.87E-10	3.27E-08	6.259261	Up	KIT ligand
GPD1	0.724563	0.005534	0.020646	2.77419	Up	glycerol-3-phosphate dehydrogenase 1
CDH13	0.722869	7.64E-07	1.72E-05	4.944381	Up	cadherin 13
BATF2	0.718971	7.44E-05	0.000703	3.961684	Up	basic leucine zipper ATF-like transcription factor 2
DPP6	0.714059	0.010784	0.034952	2.549635	Up	dipeptidyl peptidase like 6
ADARB2	0.713528	0.00297	0.012715	2.970817	Up	adenosine deaminase RNA specific B2 (inactive)
FFAR4	0.71313	0.003619	0.014805	2.909633	Up	free fatty acid receptor 4
PTPRQ	0.712554	0.000478	0.003008	3.493019	Up	protein tyrosine phosphatase receptor type Q
FIBIN	0.712085	6.50E-05	0.000628	3.994042	Up	fin bud initiation factor homolog
GPX3	0.712059	6.30E-05	0.000611	4.001106	Up	glutathione peroxidase 3
AATK	0.711857	0.000127	0.001082	3.831912	Up	apoptosis associated tyrosine kinase
FCGR1BP	0.708182	0.000147	0.001207	3.795893	Up	Fc gamma receptor Ib, pseudogene
RAPGEF6	0.705717	4.23E-15	3.54E-12	7.847881	Up	Rap guanine nucleotide exchange factor 6
TLCD4	0.704769	2.18E-10	2.09E-08	6.34799	Up	TLC domain containing 4
ETFRF1	0.704233	3.11E-15	3.12E-12	7.886485	Up	electron transfer flavoprotein regulatory factor 1
LTB	0.702138	0.00169	0.008171	3.139995	Up	lymphotoxin beta
LILRA2	0.701663	2.86E-05	0.00032	4.184232	Up	leukocyte immunoglobulin like receptor A2
FGF2	0.700257	1.32E-06	2.77E-05	4.836257	Up	fibroblast growth factor 2
PDZD2	0.699065	1.23E-06	2.60E-05	4.851409	Up	PDZ domain containing 2
ACKR4	0.697971	0.000147	0.001207	3.796077	Up	atypical chemokine receptor 4
CSF3R	0.697927	0.001087	0.005734	3.266949	Up	colony stimulating factor 3 receptor
MMP25	0.697396	0.005779	0.021418	2.760054	Up	matrix metallopeptidase 25
NDUFC2	0.696935	8.92E-10	6.31E-08	6.127583	Up	NADH:ubiquinone oxidoreductase subunit C2
MAP1LC3C	0.694668	0.004675	0.018091	2.828617	Up	microtubule associated protein 1 light chain 3 gamma
SLC26A8	0.693861	0.009029	0.030348	2.610965	Up	solute carrier family 26 member 8
TSPAN13	0.692795	2.27E-07	6.16E-06	5.175726	Up	tetraspanin 13
P3H2	0.692383	0.000102	0.000907	3.88485	Up	prolyl 3-hydroxylase 2
FUT7	0.690073	0.000843	0.004698	3.338436	Up	fucosyltransferase 7
PLAG1	0.689541	6.10E-07	1.42E-05	4.988095	Up	PLAG1 zinc finger
FASN	0.687752	0.00086	0.004769	3.33271	Up	fatty acid synthase
UPK3B	0.687654	0.010296	0.033683	2.565716	Up	uroplakin 3B
PIK3AP1	0.687184	3.00E-06	5.37E-05	4.670853	Up	phosphoinositide-3-kinase adaptor protein 1
TMEM26	0.686086	0.00388	0.015604	2.887745	Up	transmembrane protein 26
NKD1	0.682722	5.61E-05	0.000553	4.028723	Up	NKD inhibitor of WNT signaling pathway 1
ADH1B	0.682617	2.13E-07	5.87E-06	5.187407	Up	alcohol dehydrogenase 1B (class I), beta polypeptide
STXBP6	0.681277	0.002791	0.012137	2.989864	Up	syntaxin binding protein 6

KBTBD7	0.681189	1.30E-12	3.50E-10	7.094075	Up	kelch repeat and BTB domain containing 7
GPR160	0.680953	1.59E-08	7.05E-07	5.651493	Up	G protein-coupled receptor 160
FOXD1	0.680558	0.003459	0.014294	2.923738	Up	forkhead box D1
SOWAHD	0.678898	0.000356	0.002406	3.570679	Up	sosondowah ankyrin repeat domain family member D
SLC1A1	0.677852	0.000109	0.000958	3.869126	Up	solute carrier family 1 member 1
RXRG	0.677851	0.000506	0.003136	3.47759	Up	retinoid X receptor gamma
LILRA5	0.676704	0.000599	0.003593	3.432024	Up	leukocyte immunoglobulin like receptor A5
SHH	0.676703	0.001211	0.006227	3.236268	Up	sonic hedgehog signaling molecule
DGAT2	0.673676	0.002014	0.009407	3.08811	Up	diacylglycerol O-acyltransferase 2
DACH1	0.673529	1.03E-07	3.32E-06	5.320711	Up	dachshund family transcription factor 1
PNPLA3	0.671915	0.003055	0.012999	2.962189	Up	patatin like phospholipase domain containing 3
ARL5A	0.664439	2.01E-13	7.21E-11	7.348126	Up	ADP ribosylation factor like GTPase 5A
SRP9	0.664421	3.48E-13	1.17E-10	7.27437	Up	signal recognition particle 9
ACOXL	0.662442	0.00456	0.017719	2.836576	Up	acyl-CoA oxidase like
FGF9	0.661507	1.32E-05	0.000173	4.357535	Up	fibroblast growth factor 9
HMSD	0.661023	0.001177	0.006083	3.244458	Up	histocompatibility minor serpin domain containing
SLC7A11	0.660826	0.004977	0.019021	2.808517	Up	solute carrier family 7 member 11
EFR3B	0.660393	1.56E-05	0.000198	4.3196	Up	EFR3 homolog B
CLIC5	0.659958	0.000435	0.0028	3.517923	Up	chloride intracellular channel 5
SGPP1	0.65873	5.24E-13	1.68E-10	7.219008	Up	sphingosine-1-phosphate phosphatase 1
CACNB4	0.657264	7.08E-09	3.55E-07	5.788901	Up	calcium voltage-gated channel auxiliary subunit beta 4
MGAT3	0.655583	1.09E-05	0.000149	4.398573	Up	beta-1,4-mannosyl-glycoprotein 4-beta-N-acetylglucosaminyltransferase
CCDC68	0.654563	3.54E-05	0.000384	4.135296	Up	coiled-coil domain containing 68
MTARC1	0.654334	2.76E-06	5.05E-05	4.68761	Up	mitochondrial amidoxime reducing component 1
OLFML2A	0.653244	2.88E-06	5.22E-05	4.679083	Up	olfactomedin like 2A
PHGDH	0.650395	4.83E-05	0.000491	4.063913	Up	phosphoglycerate dehydrogenase
HSPA6	0.648825	0.002429	0.010867	3.031996	Up	heat shock protein family A (Hsp70) member 6
TSPAN12	0.6488	1.40E-05	0.000182	4.343794	Up	tetraspanin 12
SCAI	0.64777	1.34E-12	3.55E-10	7.089697	Up	suppressor of cancer cell invasion
KCNA4	0.647232	0.006881	0.024538	2.702528	Up	potassium voltage-gated channel subfamily A member 4
PI4K2B	0.646869	4.29E-14	2.16E-11	7.551801	Up	phosphatidylinositol 4-kinase type 2 beta
HOXD1	0.645662	0.001216	0.00625	3.235109	Up	homeobox D1
FHDC1	0.644069	0.000209	0.001571	3.70805	Up	FH2 domain containing 1
PCDH17	0.642583	0.000256	0.001849	3.656389	Up	protocadherin 17
VIP	0.642256	0.000144	0.001191	3.800489	Up	vasoactive intestinal peptide
IGSF6	0.641868	0.000127	0.00108	3.832308	Up	immunoglobulin superfamily member 6
GMFB	0.641224	1.77E-11	2.83E-09	6.723955	Up	glia maturation factor beta
PLLP	0.640686	0.00124	0.006335	3.229481	Up	plasmolipin
SCOC	0.639466	6.04E-14	2.66E-11	7.507132	Up	short coiled-coil protein
SH3GL3	0.638209	0.006549	0.023575	2.718952	Up	SH3 domain containing GRB2 like 3, endophilin A3
SDR16C5	0.636948	0.013863	0.042871	2.4608	Up	short chain dehydrogenase/reductase family 16C member 5
KLRF1	0.636534	0.000879	0.004837	3.326795	Up	killer cell lectin like receptor F1

KCNJ2	0.636167	8.40E-06	0.000122	4.454662	Up	potassium inwardly rectifying channel subfamily J member 2
TRHDE	0.635749	2.98E-05	0.000331	4.175363	Up	thyrotropin releasing hormone degrading enzyme
LRRC25	0.635206	1.85E-05	0.000226	4.281738	Up	leucine rich repeat containing 25
ADGRE3	0.631847	0.001262	0.006434	3.224479	Up	adhesion G protein-coupled receptor E3
KL	0.630848	0.000407	0.002665	3.535256	Up	klotho
UBE2D1	0.630474	3.51E-10	3.04E-08	6.27431	Up	ubiquitin conjugating enzyme E2 D1
ADH1A	0.629388	0.000114	0.000992	3.858538	Up	alcohol dehydrogenase 1A (class I), alpha polypeptide
SMAD6	0.629134	0.005888	0.021718	2.753979	Up	SMAD family member 6
ANKRD29	0.626968	4.86E-05	0.000493	4.062302	Up	ankyrin repeat domain 29
ELF5	0.626428	0.008205	0.028168	2.643534	Up	E74 like ETS transcription factor 5
KHDRBS2	0.626063	0.001111	0.005819	3.260824	Up	KH RNA binding domain containing, signal transduction associated 2
BMPR2	0.626031	1.11E-07	3.52E-06	5.307754	Up	bone morphogenetic protein receptor type 2
GCA	0.624975	9.23E-07	2.03E-05	4.907454	Up	grancalcin
AARD	0.624493	0.009499	0.031527	2.593551	Up	alanine and arginine rich domain containing protein
APOA1	0.62282	0.002119	0.009809	3.073079	Up	apolipoprotein A1
CCDC85A	0.620791	0.000687	0.003993	3.394686	Up	coiled-coil domain containing 85A
QKI	0.620615	9.91E-13	2.82E-10	7.131688	Up	QKI, KH domain containing RNA binding
NEGR1	0.619599	9.24E-05	0.000838	3.909811	Up	neuronal growth regulator 1
CALCRL	0.618234	2.85E-05	0.00032	4.184983	Up	calcitonin receptor like receptor
TGFBR3	0.617324	1.66E-06	3.34E-05	4.790577	Up	transforming growth factor beta receptor 3
SPRYD7	0.616844	1.80E-10	1.75E-08	6.377367	Up	SPRY domain containing 7
INSIG1	0.615911	8.48E-06	0.000123	4.452736	Up	insulin induced gene 1
RASGRF1	0.615844	0.003343	0.013942	2.934259	Up	Ras protein specific guanine nucleotide releasing factor 1
LRRK2	0.615661	0.001849	0.008785	3.113535	Up	leucine rich repeat kinase 2
NEBL	0.613599	5.20E-07	1.26E-05	5.018905	Up	nebulette
FAM241A	0.613362	4.39E-08	1.60E-06	5.474461	Up	family with sequence similarity 241 member A
HDHD2	0.613187	3.63E-10	3.12E-08	6.269246	Up	haloacid dehalogenase like hydrolase domain containing 2
ATP8A2	0.613091	0.000466	0.002954	3.499556	Up	ATPase phospholipid transporting 8A2
CD244	0.612948	0.000252	0.001829	3.659732	Up	CD244 molecule
TLR3	0.612335	1.62E-12	4.06E-10	7.064096	Up	toll like receptor 3
CAV2	0.607636	2.21E-07	6.04E-06	5.18093	Up	caveolin 2
HLX	0.607112	2.03E-06	3.92E-05	4.750713	Up	H2.0 like homeobox
DLEU7	0.606691	0.000446	0.002861	3.511159	Up	deleted in lymphocytic leukemia 7
CLEC4D	0.60665	0.016819	0.049809	2.390646	Up	C-type lectin domain family 4 member D
KCNMB4	0.60614	0.000532	0.003268	3.463952	Up	potassium calcium-activated channel subfamily M regulatory beta subunit 4
NLRP12	0.605152	0.002123	0.009821	3.072504	Up	NLR family pyrin domain containing 12
ADRB1	0.60511	0.009167	0.030705	2.605774	Up	adrenoceptor beta 1
OCLN	0.604663	0.000883	0.004857	3.32527	Up	occludin
VNN2	0.604537	0.004963	0.018991	2.809422	Up	vanin 2
TNFSF10	0.604197	0.000206	0.001551	3.711848	Up	TNF superfamily member 10
SPOCK2	0.603771	0.002486	0.01107	3.025061	Up	SPARC (osteonectin), cwcv and kazal like domains proteoglycan 2
SUSD2	0.602142	0.002493	0.011091	3.02423	Up	sushi domain containing 2

SLC22A3	0.601034	0.000226	0.001682	3.687598	Up	solute carrier family 22 member 3
SPIN4	0.600324	9.51E-10	6.66E-08	6.117428	Up	spindlin family member 4
CA2	0.600226	0.001853	0.0088	3.112755	Up	carbonic anhydrase 2
GCSAML	0.599908	0.000171	0.001349	3.75825	Up	germinal center associated signaling and motility like
TMEM170B	0.598208	2.27E-10	2.15E-08	6.341856	Up	transmembrane protein 170B
PAPSS2	0.597686	1.53E-05	0.000195	4.324223	Up	3'-phosphoadenosine 5'-phosphosulfate synthase 2
ST6GALNAC5	0.596594	4.33E-05	0.000448	4.088898	Up	ST6 N-acetylgalactosaminide alpha-2,6-sialyltransferase 5
EMP2	0.596575	0.000932	0.005066	3.310423	Up	epithelial membrane protein 2
GPM6B	0.596078	8.34E-05	0.000772	3.934332	Up	glycoprotein M6B
C10orf105	0.595753	0.001694	0.00818	3.139286	Up	chromosome 10 open reading frame 105
SNX10	0.594894	1.03E-05	0.000142	4.41179	Up	sorting nexin 10
LIFR	0.593395	0.000161	0.001287	3.774152	Up	LIF receptor subunit alpha
MZT1	0.593244	1.43E-18	1.44E-14	8.795224	Up	mitotic spindle organizing protein 1
PHACTR1	0.593018	8.94E-05	0.000818	3.917611	Up	phosphatase and actin regulator 1
HPN	0.59227	0.011953	0.038127	2.513526	Up	hepsin
RIMS4	0.590851	0.012128	0.038554	2.508402	Up	regulating synaptic membrane exocytosis 4
MNDA	0.589645	0.000304	0.002124	3.61208	Up	myeloid cell nuclear differentiation antigen
SLFNL1	0.587914	0.002757	0.012043	2.993569	Up	schlafen like 1
LILRB1	0.586426	0.000582	0.003516	3.440078	Up	leukocyte immunoglobulin like receptor B1
TLR8	0.585815	0.001524	0.007534	3.17001	Up	toll like receptor 8
CMPK2	0.585172	3.86E-05	0.000409	4.115815	Up	cytidine/uridine monophosphate kinase 2
GTF2A1	0.585001	4.50E-17	2.10E-13	8.399151	Up	general transcription factor IIA subunit 1
GATA3	0.584657	1.40E-05	0.000182	4.343827	Up	GATA binding protein 3
RNASE4	0.583629	0.000611	0.003646	3.426732	Up	ribonuclease A family member 4
DCXR	0.581767	0.000136	0.001136	3.815885	Up	dicarbonyl and L-xylulose reductase
RSPO2	0.581175	0.003664	0.014946	2.905734	Up	R-spondin 2
CCR2	0.579284	0.005379	0.020184	2.7834	Up	C-C motif chemokine receptor 2
IFIT3	0.579066	0.000243	0.001775	3.669268	Up	interferon induced protein with tetratricopeptide repeats 3
SFTA2	0.578857	0.016817	0.049809	2.390687	Up	surfactant associated 2
SEC14L6	0.578757	0.002014	0.009407	3.088189	Up	SEC14 like lipid binding 6
NEK7	0.578617	1.49E-11	2.55E-09	6.74864	Up	NIMA related kinase 7
TMEM41B	0.577043	1.34E-07	4.07E-06	5.272886	Up	transmembrane protein 41B
BEND4	0.57309	0.004364	0.017055	2.850564	Up	BEN domain containing 4
NKAPL	0.572662	3.23E-06	5.71E-05	4.655432	Up	NFKB activating protein like
EPB41L5	0.572631	4.51E-06	7.41E-05	4.586592	Up	erythrocyte membrane protein band 4.1 like 5
AP1S2	0.572308	1.68E-10	1.71E-08	6.388058	Up	adaptor related protein complex 1 subunit sigma 2
DIPK2A	0.572184	3.76E-09	2.12E-07	5.894403	Up	divergent protein kinase domain 2A
MARCHF1	0.571421	4.18E-06	6.98E-05	4.602393	Up	membrane associated ring-CH-type finger 1
KCTD14	0.571341	0.001482	0.007365	3.178195	Up	potassium channel tetramerization domain containing 14
VGLL3	0.571145	0.000174	0.001367	3.753726	Up	vestigial like family member 3
CD274	0.570647	0.000935	0.005083	3.309381	Up	CD274 molecule
SPRING1	0.570626	9.22E-06	0.000131	4.43474	Up	SREBF pathway regulator in golgi 1
ALDH6A1	0.568506	1.54E-09	1.00E-07	6.03992	Up	aldehyde dehydrogenase 6 family member A1

B3GALT2	0.567137	0.0036	0.014744	2.911272	Up	beta-1,3-galactosyltransferase 2
DDIAS	0.566491	5.06E-05	0.000508	4.053038	Up	DNA damage induced apoptosis suppressor
STARD4	0.565099	1.06E-06	2.27E-05	4.880904	Up	StAR related lipid transfer domain containing 4
GUCY1A2	0.563988	0.000793	0.004475	3.355179	Up	guanylate cyclase 1 soluble subunit alpha 2
CHPT1	0.563345	1.45E-11	2.51E-09	6.752944	Up	choline phosphotransferase 1
TLR7	0.562992	0.001032	0.005492	3.281558	Up	toll like receptor 7
MFSD2A	0.562216	0.015201	0.046189	2.42755	Up	major facilitator superfamily domain containing 2A
RALA	0.562144	4.12E-06	6.91E-05	4.605427	Up	RAS like proto-oncogene A
DCC	0.559864	0.009224	0.030846	2.60364	Up	DCC netrin 1 receptor
CCRL2	0.559776	0.000221	0.001645	3.694255	Up	C-C motif chemokine receptor like 2
PRAM1	0.559391	0.000509	0.00315	3.475976	Up	PML-RARA regulated adaptor molecule 1
TMEM47	0.559081	1.57E-05	0.000199	4.318306	Up	transmembrane protein 47
SIGLEC14	0.558047	0.002271	0.010365	3.052288	Up	sialic acid binding Ig like lectin 14
BEX1	0.557362	0.00419	0.016571	2.863505	Up	brain expressed X-linked 1
MAL2	0.556666	0.000456	0.002911	3.505149	Up	mal, T cell differentiation protein 2
BMPER	0.556641	3.31E-05	0.000361	4.150888	Up	BMP binding endothelial regulator
ARHGAP29	0.555352	5.44E-07	1.29E-05	5.010112	Up	Rho GTPase activating protein 29
SCAMP1	0.554475	5.70E-10	4.36E-08	6.198384	Up	secretory carrier membrane protein 1
TMEM139	0.553966	0.011346	0.036506	2.531867	Up	transmembrane protein 139
PDP2	0.552511	4.71E-07	1.16E-05	5.037684	Up	pyruvate dehydrogenase phosphatase catalytic subunit 2
DUSP26	0.55159	0.001678	0.008127	3.141919	Up	dual specificity phosphatase 26
ZMYM1	0.550816	5.89E-11	7.16E-09	6.54647	Up	zinc finger MYM-type containing 1
UPB1	0.550478	0.013577	0.042165	2.468259	Up	beta-ureidopropionase 1
KANK4	0.550172	0.007273	0.025625	2.684059	Up	KN motif and ankyrin repeat domains 4
SMCO3	0.549942	0.00845	0.02886	2.633539	Up	single-pass membrane protein with coiled-coil domains 3
ACP3	0.549624	0.001393	0.007011	3.19603	Up	acid phosphatase 3
EFNB2	0.54929	2.77E-05	0.000312	4.191807	Up	ephrin B2
CCBE1	0.548101	0.00059	0.003551	3.436371	Up	collagen and calcium binding EGF domains 1
PDE8B	0.547797	0.000263	0.001892	3.649284	Up	phosphodiesterase 8B
ZBTB41	0.547533	5.54E-09	2.87E-07	5.830228	Up	zinc finger and BTB domain containing 41
HPCAL1	0.547012	4.84E-05	0.000492	4.063307	Up	hippocalcin like 1
PKIA	0.546874	2.98E-08	1.16E-06	5.542764	Up	cAMP-dependent protein kinase inhibitor alpha
HSD17B12	0.545849	1.63E-15	2.23E-12	7.966727	Up	hydroxysteroid 17-beta dehydrogenase 12
ANKRD22	0.545776	0.005242	0.019788	2.791769	Up	ankyrin repeat domain 22
FZD8	0.545717	1.33E-05	0.000175	4.354308	Up	frizzled class receptor 8
CAV1	0.545385	1.30E-05	0.000172	4.359406	Up	caveolin 1
C1orf167	0.544974	0.003796	0.015339	2.894656	Up	chromosome 1 open reading frame 167
ACSS2	0.543889	2.86E-05	0.00032	4.184137	Up	acyl-CoA synthetase short chain family member 2
DHCR7	0.543582	0.001483	0.007368	3.177912	Up	7-dehydrocholesterol reductase
SP6	0.543313	0.010387	0.033899	2.562678	Up	Sp6 transcription factor
ANP32E	0.542858	3.64E-16	7.83E-13	8.150004	Up	acidic nuclear phosphoprotein 32 family member E
CASD1	0.542753	9.59E-11	1.10E-08	6.473351	Up	CAS1 domain containing 1
ST20	0.541365	0.00043	0.002783	3.520756	Up	suppressor of tumorigenicity 20

UBE2W	0.540435	1.97E-12	4.63E-10	7.036862	Up	ubiquitin conjugating enzyme E2 W
FREM2	0.540052	0.010923	0.035359	2.54514	Up	FRAS1 related extracellular matrix 2
SLC25A46	0.539395	1.70E-16	5.11E-13	8.241772	Up	solute carrier family 25 member 46
SC5D	0.539203	1.77E-11	2.83E-09	6.723974	Up	sterol-C5-desaturase
ID4	0.538984	0.000302	0.002117	3.613301	Up	inhibitor of DNA binding 4
ARRB1	0.537974	8.85E-06	0.000127	4.443454	Up	arrestin beta 1
CLCN4	0.537838	2.41E-06	4.49E-05	4.715224	Up	chloride voltage-gated channel 4
TRPC3	0.53746	0.002454	0.01095	3.028973	Up	transient receptor potential cation channel subfamily C member 3
CR1	0.537417	0.008051	0.027755	2.649923	Up	complement C3b/C4b receptor 1 (Knops blood group)
GLT1D1	0.537203	0.013284	0.041423	2.476075	Up	glycosyltransferase 1 domain containing 1
WWC2	0.536541	4.47E-05	0.000461	4.081995	Up	WW and C2 domain containing 2
B3GALNT1	0.536032	0.0001	0.000891	3.88977	Up	beta-1,3-N-acetylgalactosaminyltransferase 1 (globoside blood group)
GPR82	0.53494	0.008485	0.028952	2.632148	Up	G protein-coupled receptor 82
ZBTB6	0.533771	1.14E-09	7.77E-08	6.088452	Up	zinc finger and BTB domain containing 6
ERVMER34-1	0.532895	0.000462	0.002935	3.501656	Up	endogenous retrovirus group MER34 member 1, envelope
HECW2	0.532689	0.014359	0.044119	2.448161	Up	HECT, C2 and WW domain containing E3 ubiquitin protein ligase 2
PEG10	0.531923	0.006459	0.023346	2.723532	Up	paternally expressed 10
ANOS1	0.53174	0.000843	0.004699	3.338126	Up	anosmin 1
PPP3R1	0.530836	2.47E-15	2.80E-12	7.914945	Up	protein phosphatase 3 regulatory subunit B, alpha
DLL4	0.529304	0.00067	0.003915	3.401581	Up	delta like canonical Notch ligand 4
CFL2	0.529034	3.05E-09	1.79E-07	5.928999	Up	cofilin 2
MEFV	0.528895	0.010878	0.035226	2.546605	Up	MEFV innate immuity regulator, pyrin
TMC2	0.527636	0.009997	0.032918	2.575942	Up	transmembrane channel like 2
ANXA3	0.527469	0.005872	0.021672	2.754838	Up	annexin A3
TFPI	0.527239	1.69E-07	4.90E-06	5.230895	Up	tissue factor pathway inhibitor
MYLK4	0.527064	0.000588	0.003547	3.437147	Up	myosin light chain kinase family member 4
MAPT	0.527029	0.002179	0.010021	3.064653	Up	microtubule associated protein tau
ACAT2	0.52679	5.87E-05	0.000574	4.017866	Up	acetyl-CoA acetyltransferase 2
C1GALT1	0.526288	3.28E-11	4.62E-09	6.633558	Up	core 1 synthase, glycoprotein-N-acetylgalactosamine 3-beta-galactosyltransferase 1
ASGR2	0.525767	0.009556	0.031683	2.591481	Up	asialoglycoprotein receptor 2
C19orf38	0.525456	0.001171	0.006059	3.245911	Up	chromosome 19 open reading frame 38
SLC33A1	0.524917	5.87E-10	4.44E-08	6.193931	Up	solute carrier family 33 member 1
EDIL3	0.524859	5.00E-05	0.000505	4.055521	Up	EGF like repeats and discoidin domains 3
MATN3	0.524406	0.006459	0.023346	2.723514	Up	matrilin 3
EBP	0.524406	0.002161	0.009953	3.067148	Up	EBP cholestenol delta-isomerase
C21orf91	0.524032	3.64E-09	2.08E-07	5.899624	Up	chromosome 21 open reading frame 91
SLITRK2	0.523462	0.014717	0.045018	2.439267	Up	SLIT and NTRK like family member 2
CXADR	0.523136	0.000903	0.004937	3.319175	Up	CXADR Ig-like cell adhesion molecule
IRX1	0.522985	0.008309	0.028482	2.639239	Up	iroquois homeobox 1
EPAS1	0.522235	0.000563	0.003424	3.448637	Up	endothelial PAS domain protein 1
ADGRG2	0.522134	0.001746	0.008397	3.130394	Up	adhesion G protein-coupled receptor G2
RBM27	0.52167	3.55E-09	2.05E-07	5.903944	Up	RNA binding motif protein 27

IDI1	0.521166	3.05E-07	7.98E-06	5.120342	Up	isopentenyl-diphosphate delta isomerase 1
RNF144B	0.5209	9.46E-08	3.09E-06	5.336847	Up	ring finger protein 144B
TBX3	0.520278	7.70E-05	0.000723	3.953524	Up	T-box transcription factor 3
IRX2	0.519862	0.003849	0.015513	2.890261	Up	iroquois homeobox 2
FADS1	0.519046	2.67E-05	0.000303	4.199613	Up	fatty acid desaturase 1
GIMAP4	0.517909	1.94E-07	5.43E-06	5.205286	Up	GTPase, IMAP family member 4
DKK2	0.516481	0.006694	0.024018	2.711671	Up	dickkopf WNT signaling pathway inhibitor 2
DNAJB14	0.51623	4.92E-12	9.75E-10	6.907878	Up	DnaJ heat shock protein family (Hsp40) member B14
PHACTR2	0.516147	1.67E-11	2.74E-09	6.732116	Up	phosphatase and actin regulator 2
PXMP4	0.515393	0.00028	0.001987	3.633488	Up	peroxisomal membrane protein 4
GPER1	0.515095	0.006923	0.024657	2.700507	Up	G protein-coupled estrogen receptor 1
SUCNR1	0.514658	0.001614	0.007884	3.153283	Up	succinate receptor 1
PCLAF	0.51345	0.005203	0.019684	2.794197	Up	PCNA clamp associated factor
CDC20B	-2.45054	7.11E-06	0.000108	-4.49029	Down	cell division cycle 20B
MUC5AC	-2.19179	0.000911	0.004971	-3.31675	Down	mucin 5AC, oligomeric mucus/gel-forming
CYP2A6	-1.92571	8.42E-06	0.000122	-4.45413	Down	cytochrome P450 family 2 subfamily A member 6
SERPINB11	-1.86724	0.004146	0.016433	-2.86683	Down	serpin family B member 11
PCK1	-1.82117	0.000302	0.002114	-3.61393	Down	phosphoenolpyruvate carboxykinase 1
BTG4	-1.80784	0.000269	0.001923	-3.64357	Down	BTG anti-proliferation factor 4
MSMB	-1.78547	0.005298	0.019954	-2.78833	Down	microseminoprotein beta
LYPD2	-1.74807	5.39E-07	1.28E-05	-5.01174	Down	LY6/PLAUR domain containing 2
PITX1	-1.73123	0.000148	0.001213	-3.79403	Down	paired like homeodomain 1
ATP12A	-1.7033	0.000434	0.0028	-3.51833	Down	ATPase H+/K+ transporting non-gastric alpha2 subunit
SERPINB3	-1.66423	0.007278	0.02563	-2.68384	Down	serpin family B member 3
FRMPD2	-1.66279	0.00032	0.002212	-3.59869	Down	FERM and PDZ domain containing 2
FER1L6	-1.62163	0.000198	0.001507	-3.72107	Down	fer-1 like family member 6
RSPH10B2	-1.6209	0.000122	0.001051	-3.84112	Down	radial spoke head 10 homolog B2
MCIDAS	-1.59413	0.001536	0.007584	-3.16773	Down	multiciliate differentiation and DNA synthesis associated cell cycle protein
LRRC74B	-1.58345	0.000224	0.001666	-3.69019	Down	leucine rich repeat containing 74B
CCNO	-1.57511	7.78E-05	0.000729	-3.95109	Down	cyclin O
CYP2F1	-1.56943	0.002997	0.012805	-2.96804	Down	cytochrome P450 family 2 subfamily F member 1
KRT14	-1.56546	0.008895	0.029992	-2.61607	Down	keratin 14
GSTA2	-1.56532	0.00216	0.009953	-3.06725	Down	glutathione S-transferase alpha 2
MMP10	-1.56288	0.000348	0.002366	-3.57649	Down	matrix metallopeptidase 10
DEUP1	-1.56273	0.000147	0.001207	-3.79615	Down	deuterosome assembly protein 1
DRC1	-1.54327	0.000186	0.001436	-3.73667	Down	dynein regulatory complex subunit 1
FBXW10	-1.53831	0.000179	0.001397	-3.74627	Down	F-box and WD repeat domain containing 10
DNAAF1	-1.538	1.91E-05	0.000231	-4.27558	Down	dynein axonemal assembly factor 1
DNAI1	-1.53788	0.000288	0.002037	-3.62592	Down	dynein axonemal intermediate chain 1
CCDC190	-1.52967	0.000629	0.003727	-3.41874	Down	coiled-coil domain containing 190
CFAP299	-1.51757	0.000871	0.004807	-3.32913	Down	cilia and flagella associated protein 299
DNAH3	-1.51576	0.000476	0.003004	-3.49366	Down	dynein axonemal heavy chain 3

EPPIN	-1.51207	0.001943	0.009141	-3.09884	Down	epididymal peptidase inhibitor
DNAH10	-1.49046	7.18E-05	0.000681	-3.97035	Down	dynein axonemal heavy chain 10
CFAP100	-1.48643	0.000481	0.003027	-3.491	Down	cilia and flagella associated protein 100
CFAP157	-1.48478	0.000181	0.001405	-3.74453	Down	cilia and flagella associated protein 157
CRABP1	-1.48438	0.000295	0.002076	-3.62005	Down	cellular retinoic acid binding protein 1
SEC14L3	-1.48299	0.000159	0.001281	-3.77619	Down	SEC14 like lipid binding 3
KIAA2012	-1.482	0.000814	0.004573	-3.34805	Down	KIAA2012
MUC13	-1.47501	0.000448	0.002869	-3.5101	Down	mucin 13, cell surface associated
CFAP52	-1.47438	0.000374	0.002486	-3.55781	Down	cilia and flagella associated protein 52
DCDC2B	-1.47015	0.00077	0.00437	-3.36344	Down	doublecortin domain containing 2B
CYP2A13	-1.46618	0.013335	0.041539	-2.47471	Down	cytochrome P450 family 2 subfamily A member 13
HOATZ	-1.45943	0.001111	0.005819	-3.26081	Down	HOATZ cilia and flagella associated protein
MS4A8	-1.45835	0.001088	0.005739	-3.26664	Down	membrane spanning 4-domains A8
CDHR4	-1.45622	0.000217	0.001624	-3.69827	Down	cadherin related family member 4
NME9	-1.4507	0.000138	0.001148	-3.81261	Down	NME/NM23 family member 9
BEST4	-1.44768	1.63E-06	3.29E-05	-4.79482	Down	bestrophin 4
CROCC2	-1.44683	2.06E-05	0.000246	-4.25839	Down	ciliary rootlet coiled-coil, rootletin family member 2
C11orf16	-1.44035	0.001031	0.005487	-3.28191	Down	chromosome 11 open reading frame 16
ALOX15	-1.43719	0.000256	0.001851	-3.65579	Down	arachidonate 15-lipoxygenase
LMNTD1	-1.43668	0.001573	0.007718	-3.16092	Down	lamin tail domain containing 1
DRD5	-1.43419	0.000173	0.001363	-3.75484	Down	dopamine receptor D5
DNAH9	-1.4312	0.000456	0.002911	-3.50508	Down	dynein axonemal heavy chain 9
HYDIN	-1.42919	0.000326	0.002243	-3.59387	Down	HYDIN axonemal central pair apparatus protein
DRC3	-1.42869	1.72E-06	3.43E-05	-4.78429	Down	dynein regulatory complex subunit 3
CFAP57	-1.42646	6.87E-05	0.000657	-3.98087	Down	cilia and flagella associated protein 57
CFAP74	-1.42588	0.00016	0.001283	-3.77507	Down	cilia and flagella associated protein 74
AKAP14	-1.4216	0.001315	0.006677	-3.21268	Down	A-kinase anchoring protein 14
TEKT2	-1.41712	0.000197	0.001502	-3.72225	Down	tektin 2
MUC16	-1.41705	0.002105	0.009755	-3.07498	Down	mucin 16, cell surface associated
TEKT1	-1.41461	0.001166	0.006046	-3.24713	Down	tektin 1
CFAP46	-1.41418	9.25E-05	0.000838	-3.90959	Down	cilia and flagella associated protein 46
CFAP65	-1.41029	0.000568	0.003447	-3.44657	Down	cilia and flagella associated protein 65
SPAG17	-1.40988	0.000242	0.001768	-3.67064	Down	sperm associated antigen 17
C7orf57	-1.4069	0.000251	0.001818	-3.66158	Down	chromosome 7 open reading frame 57
DNAJB13	-1.40688	3.71E-05	0.000398	-4.12498	Down	DnaJ heat shock protein family (Hsp40) member B13
CCNA1	-1.40251	2.12E-05	0.000251	-4.25187	Down	cyclin A1
SLC23A1	-1.39999	0.000596	0.00358	-3.43333	Down	solute carrier family 23 member 1
VWA3B	-1.39961	0.000985	0.005296	-3.29466	Down	von Willebrand factor A domain containing 3B
C1orf87	-1.39865	0.000364	0.002445	-3.56502	Down	chromosome 1 open reading frame 87
CAPSL	-1.39804	0.001974	0.00925	-3.09411	Down	calcyphosine like
CCDC33	-1.39757	0.002031	0.009463	-3.08566	Down	coiled-coil domain containing 33
STOML3	-1.39531	0.001925	0.009086	-3.10164	Down	stomatin like 3
CFAP53	-1.39272	5.10E-05	0.000511	-4.051	Down	cilia and flagella associated protein 53

ARMC3	-1.39223	0.000975	0.005262	-3.29751	Down	armadillo repeat containing 3
LDLRAD1	-1.39168	0.001236	0.00632	-3.23055	Down	low density lipoprotein receptor class A domain containing 1
SIAH3	-1.3916	0.000331	0.002275	-3.58974	Down	siah E3 ubiquitin protein ligase family member 3
DNAH12	-1.3911	0.000455	0.002906	-3.50599	Down	dynein axonemal heavy chain 12
CCDC65	-1.39073	4.40E-05	0.000455	-4.08516	Down	coiled-coil domain containing 65
ODAD1	-1.39061	0.000116	0.001004	-3.85487	Down	outer dynein arm docking complex subunit 1
MAP3K19	-1.39039	0.002261	0.010329	-3.05363	Down	mitogen-activated protein kinase kinase kinase 19
ABHD12B	-1.38167	0.000919	0.005009	-3.31429	Down	abhydrolase domain containing 12B
PRR29	-1.37458	0.00013	0.001101	-3.82653	Down	proline rich 29
TTLL9	-1.3741	1.77E-05	0.000218	-4.29175	Down	tubulin tyrosine ligase like 9
MARCHF10	-1.37183	0.000111	0.000972	-3.86458	Down	membrane associated ring-CH-type finger 10
RSPH14	-1.37091	0.000386	0.002554	-3.54952	Down	radial spoke head 14 homolog
CFAP107	-1.36945	0.002814	0.012205	-2.98736	Down	cilia and flagella associated protein 107
DNAH11	-1.36912	1.20E-06	2.56E-05	-4.85515	Down	dynein axonemal heavy chain 11
EPGN	-1.36659	0.000978	0.005268	-3.29672	Down	epithelial mitogen
PLEKHS1	-1.36261	0.003129	0.013232	-2.95482	Down	pleckstrin homology domain containing S1
CCDC60	-1.36131	0.002295	0.010434	-3.0492	Down	coiled-coil domain containing 60
MB	-1.36073	0.001172	0.006064	-3.24555	Down	myoglobin
LRRC43	-1.35643	5.40E-05	0.000536	-4.03746	Down	leucine rich repeat containing 43
CFAP43	-1.35602	0.000327	0.00225	-3.59299	Down	cilia and flagella associated protein 43
AGBL2	-1.3542	2.39E-05	0.000276	-4.22534	Down	AGBL carboxypeptidase 2
C22orf15	-1.34631	0.000127	0.001082	-3.83159	Down	chromosome 22 open reading frame 15
DLEC1	-1.34525	5.07E-05	0.000509	-4.05253	Down	DLEC1 cilia and flagella associated protein
DYDC1	-1.34524	0.008968	0.030192	-2.61325	Down	DPY30 domain containing 1
DYDC2	-1.34405	0.001805	0.00862	-3.12054	Down	DPY30 domain containing 2
ADGB	-1.34217	0.000675	0.003934	-3.39965	Down	androglobin
TMEM212	-1.33979	0.00466	0.018046	-2.82965	Down	transmembrane protein 212
ACSBG1	-1.33763	0.000931	0.005064	-3.31068	Down	acyl-CoA synthetase bubblegum family member 1
MROH9	-1.33754	0.001509	0.007477	-3.17291	Down	maestro heat like repeat family member 9
PZP	-1.33684	2.07E-05	0.000246	-4.25731	Down	PZP alpha-2-macroglobulin like
ANKRD66	-1.33574	0.003085	0.013091	-2.95915	Down	ankyrin repeat domain 66
ABCA13	-1.33509	0.000478	0.003008	-3.49296	Down	ATP binding cassette subfamily A member 13
ANKUB1	-1.3333	0.002675	0.011754	-3.00286	Down	ankyrin repeat and ubiquitin domain containing 1
BDKRB1	-1.33284	0.000232	0.001712	-3.68116	Down	bradykinin receptor B1
SPAG6	-1.3304	0.001799	0.008601	-3.12148	Down	sperm associated antigen 6
RSPH1	-1.3303	0.000694	0.004022	-3.3919	Down	radial spoke head component 1
MYCBPAP	-1.32909	2.41E-06	4.49E-05	-4.71546	Down	MYCBP associated protein
VWA3A	-1.32629	3.59E-05	0.000389	-4.13216	Down	von Willebrand factor A domain containing 3A
GABRP	-1.32552	7.53E-05	0.000709	-3.95886	Down	gamma-aminobutyric acid type A receptor subunit pi
CFAP47	-1.32283	0.001507	0.007469	-3.17341	Down	cilia and flagella associated protein 47
C6orf118	-1.3228	0.002416	0.01082	-3.03365	Down	chromosome 6 open reading frame 118
CCDC17	-1.32279	8.52E-05	0.000785	-3.92928	Down	coiled-coil domain containing 17
FAM81B	-1.31212	0.000499	0.003103	-3.48156	Down	family with sequence similarity 81 member B

RSPH10B	-1.31186	0.004757	0.018327	-2.82307	Down	radial spoke head 10 homolog B
SPEF1	-1.31104	0.000973	0.005253	-3.29821	Down	sperm flagellar 1
SPATA4	-1.30741	0.001023	0.005452	-3.28412	Down	spermatogenesis associated 4
OTX1	-1.30674	0.000497	0.003097	-3.4822	Down	orthodenticle homeobox 1
DRC7	-1.30622	0.001482	0.007365	-3.17812	Down	dynein regulatory complex subunit 7
RIBC1	-1.30608	4.80E-06	7.74E-05	-4.57353	Down	RIB43A domain with coiled-coils 1
ERICH5	-1.30256	0.000149	0.001217	-3.79204	Down	glutamate rich 5
DNAH2	-1.30031	0.000169	0.001337	-3.76169	Down	dynein axonemal heavy chain 2
WDR38	-1.29892	0.003196	0.013448	-2.94821	Down	WD repeat domain 38
GJB7	-1.29822	0.002715	0.011902	-2.99824	Down	gap junction protein beta 7
KRT13	-1.29734	0.000118	0.001023	-3.84955	Down	keratin 13
LRRC46	-1.29681	0.000656	0.003851	-3.40716	Down	leucine rich repeat containing 46
RP1	-1.29477	0.000189	0.001454	-3.73272	Down	RP1 axonemal microtubule associated
CIBAR2	-1.29428	0.001191	0.006144	-3.24095	Down	CBY1 interacting BAR domain containing 2
DNAAF6	-1.29411	0.006765	0.024209	-2.70818	Down	dynein axonemal assembly factor 6
DNAI3	-1.29292	3.65E-05	0.000393	-4.1289	Down	dynein axonemal intermediate chain 3
NWD1	-1.29283	0.001982	0.009273	-3.0929	Down	NACHT and WD repeat domain containing 1
RIBC2	-1.29249	4.48E-05	0.000462	-4.08122	Down	RIB43A domain with coiled-coils 2
EFCAB12	-1.29229	1.96E-05	0.000235	-4.26974	Down	EF-hand calcium binding domain 12
GAS2L2	-1.29167	0.000446	0.002861	-3.51141	Down	growth arrest specific 2 like 2
TTC29	-1.29102	0.001492	0.007403	-3.17626	Down	tetratricopeptide repeat domain 29
DNAI2	-1.29057	0.000874	0.004816	-3.32833	Down	dynein axonemal intermediate chain 2
ECT2L	-1.28912	0.002408	0.010794	-3.03462	Down	epithelial cell transforming 2 like
TTLL10	-1.2891	0.000215	0.001608	-3.70097	Down	tubulin tyrosine ligase like 10
CCDC187	-1.28712	0.002132	0.009854	-3.07126	Down	coiled-coil domain containing 187
ERICH3	-1.28616	0.002309	0.010469	-3.04734	Down	glutamate rich 3
EFHC2	-1.28458	1.47E-06	3.02E-05	-4.81538	Down	EF-hand domain containing 2
FABP6	-1.28364	0.00152	0.007517	-3.17076	Down	fatty acid binding protein 6
TEKT4	-1.28364	0.003662	0.014943	-2.90589	Down	tektin 4
C2orf50	-1.28341	0.00013	0.001103	-3.8257	Down	chromosome 2 open reading frame 50
STMND1	-1.28245	0.003482	0.014384	-2.9216	Down	stathmin domain containing 1
FAM166B	-1.28221	0.000493	0.003081	-3.48439	Down	family with sequence similarity 166 member B
ZBBX	-1.28184	0.002548	0.011283	-3.01759	Down	zinc finger B-box domain containing
MORN5	-1.28089	0.004492	0.017481	-2.84138	Down	MORN repeat containing 5
FAM83A	-1.2803	0.002422	0.010838	-3.03297	Down	family with sequence similarity 83 member A
NEK10	-1.27895	0.000178	0.001391	-3.74866	Down	NIMA related kinase 10
CNGA4	-1.27882	0.000572	0.003464	-3.4445	Down	cyclic nucleotide gated channel subunit alpha 4
MDH1B	-1.27823	5.12E-05	0.000512	-4.0499	Down	malate dehydrogenase 1B
CFAP141	-1.27565	0.00277	0.012071	-2.99217	Down	cilia and flagella associated protein 141
DNAAF8	-1.2744	0.000129	0.001095	-3.82832	Down	dynein axonemal assembly factor 8
DCDC1	-1.27316	0.000707	0.00408	-3.38688	Down	doublecortin domain containing 1
CDHR3	-1.27308	0.000227	0.001688	-3.68637	Down	cadherin related family member 3
SCGB1A1	-1.27268	0.003213	0.013501	-2.94662	Down	secretoglobin family 1A member 1

DNAH7	-1.27175	8.50E-05	0.000784	-3.92975	Down	dynein axonemal heavy chain 7
CFAP45	-1.27137	7.93E-05	0.000741	-3.94648	Down	cilia and flagella associated protein 45
PSCA	-1.27123	0.008256	0.028325	-2.64143	Down	prostate stem cell antigen
USP6	-1.27031	0.000455	0.002906	-3.50588	Down	ubiquitin specific peptidase 6
APOBEC4	-1.27016	0.006425	0.023245	-2.72527	Down	apolipoprotein B mRNA editing enzyme catalytic polypeptide like 4
SPAG8	-1.26988	8.03E-06	0.000117	-4.46431	Down	sperm associated antigen 8
AGBL4	-1.26707	0.000249	0.001808	-3.66355	Down	AGBL carboxypeptidase 4
WDR93	-1.26668	0.000582	0.003517	-3.4399	Down	WD repeat domain 93
LRRC71	-1.26666	0.000148	0.001211	-3.79497	Down	leucine rich repeat containing 71
CFAP70	-1.26539	6.49E-06	9.94E-05	-4.50988	Down	cilia and flagella associated protein 70
FAM216B	-1.26428	0.001632	0.007955	-3.15018	Down	family with sequence similarity 216 member B
TTC16	-1.26272	7.16E-06	0.000108	-4.48889	Down	tetratricopeptide repeat domain 16
DNAH6	-1.26032	0.000161	0.00129	-3.77332	Down	dynein axonemal heavy chain 6
CCDC78	-1.25798	0.000571	0.003463	-3.44482	Down	coiled-coil domain containing 78
UBXN10	-1.25719	5.46E-06	8.61E-05	-4.54634	Down	UBX domain protein 10
GCNT3	-1.25692	6.43E-06	9.87E-05	-4.51177	Down	glucosaminyl (N-acetyl) transferase 3, mucin type
C20orf85	-1.2563	0.005489	0.020503	-2.77685	Down	chromosome 20 open reading frame 85
EDN2	-1.25625	0.000162	0.001294	-3.77207	Down	endothelin 2
CFAP61	-1.25619	0.000714	0.004108	-3.3841	Down	cilia and flagella associated protein 61
ANKFN1	-1.25432	8.12E-05	0.000754	-3.94087	Down	ankyrin repeat and fibronectin type III domain containing 1
CFAP126	-1.25128	0.002919	0.012556	-2.97612	Down	cilia and flagella associated protein 126
ZMYND10	-1.24945	0.000411	0.002684	-3.53271	Down	zinc finger MYND-type containing 10
HHATL	-1.2478	0.000479	0.003016	-3.49204	Down	hedgehog acyltransferase like
TSPAN1	-1.24767	0.000416	0.00271	-3.52965	Down	tetraspanin 1
DNAI7	-1.24575	0.00014	0.001165	-3.80789	Down	dynein axonemal intermediate chain 7
ODAD2	-1.24463	0.000751	0.004289	-3.37038	Down	outer dynein arm docking complex subunit 2
TSNAXIP1	-1.2444	1.97E-06	3.84E-05	-4.7567	Down	translin associated factor X interacting protein 1
TCTE1	-1.24393	0.001099	0.005778	-3.26384	Down	t-complex-associated-testis-expressed 1
CWH43	-1.24198	0.009091	0.030497	-2.6086	Down	cell wall biogenesis 43 C-terminal homolog
CFAP251	-1.23807	3.61E-05	0.00039	-4.13124	Down	cilia and flagella associated protein 251
SRD5A2	-1.2364	0.003639	0.014872	-2.90788	Down	steroid 5 alpha-reductase 2
FAM183A	-1.23568	0.004204	0.016612	-2.86247	Down	family with sequence similarity 183 member A
ZNF474	-1.23323	0.000243	0.001775	-3.66941	Down	zinc finger protein 474
ENO4	-1.23284	2.30E-05	0.000268	-4.23333	Down	enolase 4
ODAD3	-1.23187	0.000116	0.001008	-3.8538	Down	outer dynein arm docking complex subunit 3
GRIN3B	-1.23008	0.000979	0.00527	-3.29645	Down	glutamate ionotropic receptor NMDA type subunit 3B
PRMT8	-1.22706	1.24E-05	0.000165	-4.36969	Down	protein arginine methyltransferase 8
MAPK15	-1.22543	5.48E-05	0.000542	-4.03425	Down	mitogen-activated protein kinase 15
RSPH4A	-1.22495	0.000346	0.002357	-3.57791	Down	radial spoke head component 4A
DYNLT4	-1.22382	0.001851	0.008793	-3.1132	Down	dynein light chain Tctex-type 4
CHST9	-1.22362	0.008836	0.029834	-2.61833	Down	carbohydrate sulfotransferase 9
B3GALT5	-1.22351	0.001658	0.008047	-3.14559	Down	beta-1,3-galactosyltransferase 5
CATSPERD	-1.22284	0.005532	0.020643	-2.77431	Down	cation channel sperm associated auxiliary subunit delta

CLDN2	-1.22151	0.000289	0.00204	-3.62539	Down	claudin 2
MMP3	-1.21893	0.002787	0.012127	-2.99031	Down	matrix metallopeptidase 3
FBXO15	-1.21725	0.00011	0.000963	-3.86761	Down	F-box protein 15
FANK1	-1.21713	4.60E-06	7.52E-05	-4.58221	Down	fibronectin type III and ankyrin repeat domains 1
CFAP20DC	-1.21592	8.34E-06	0.000121	-4.45625	Down	CFAP20 domain containing
PTPRT	-1.21543	0.001025	0.00546	-3.28359	Down	protein tyrosine phosphatase receptor type T
ERN2	-1.2141	0.008276	0.028388	-2.6406	Down	endoplasmic reticulum to nucleus signaling 2
UCN3	-1.21302	0.002498	0.011111	-3.02357	Down	urocortin 3
CCDC74A	-1.2121	2.11E-06	4.02E-05	-4.74279	Down	coiled-coil domain containing 74A
PIFO	-1.2117	7.11E-05	0.000676	-3.9726	Down	primary cilia formation
C4orf47	-1.21169	3.94E-05	0.000416	-4.1107	Down	chromosome 4 open reading frame 47
SLC5A5	-1.21148	0.011615	0.037229	-2.52364	Down	solute carrier family 5 member 5
FUT6	-1.21098	0.001976	0.009252	-3.09385	Down	fucosyltransferase 6
DNAAF3	-1.21041	0.000819	0.004595	-3.34632	Down	dynein axonemal assembly factor 3
FAM227A	-1.20805	4.98E-06	7.99E-05	-4.56579	Down	family with sequence similarity 227 member A
ENKUR	-1.20729	0.002356	0.010623	-3.04127	Down	enkurin, TRPC channel interacting protein
UCN2	-1.20719	0.000531	0.003263	-3.46449	Down	urocortin 2
CFAP210	-1.20648	0.000229	0.001692	-3.68471	Down	cilia and flagella associated protein 210
TOGARAM2	-1.20565	1.10E-05	0.00015	-4.39719	Down	TOG array regulator of axonemal microtubules 2
ROPN1L	-1.20441	0.000491	0.003075	-3.48548	Down	rhophilin associated tail protein 1 like
IQCD	-1.20391	1.99E-05	0.000239	-4.26605	Down	IQ motif containing D
SCGB2A1	-1.20224	0.003315	0.01385	-2.9369	Down	secretoglobin family 2A member 1
C5orf49	-1.20221	0.001575	0.007725	-3.16053	Down	chromosome 5 open reading frame 49
WDR49	-1.20137	7.45E-05	0.000703	-3.96139	Down	WD repeat domain 49
DIO1	-1.19999	0.00133	0.006745	-3.2095	Down	iodothyronine deiodinase 1
CLXN	-1.1998	0.001452	0.007253	-3.18418	Down	calaxin
ODAD4	-1.19926	5.32E-05	0.000529	-4.0412	Down	outer dynein arm docking complex subunit 4
DYNLRB2	-1.19841	0.000843	0.004698	-3.33828	Down	dynein light chain roadblock-type 2
MAT1A	-1.19807	0.000202	0.001533	-3.71628	Down	methionine adenosyltransferase 1A
DAW1	-1.1971	0.000236	0.001729	-3.67752	Down	dynein assembly factor with WD repeats 1
RSPH9	-1.19318	0.000572	0.003465	-3.44432	Down	radial spoke head component 9
C2orf73	-1.19263	0.001074	0.005675	-3.27027	Down	chromosome 2 open reading frame 73
CCDC74B	-1.19213	0.000263	0.00189	-3.64962	Down	coiled-coil domain containing 74B
CATIP	-1.19199	0.000662	0.003876	-3.40465	Down	ciliogenesis associated TTC17 interacting protein
MEIG1	-1.19089	0.000534	0.003273	-3.46332	Down	meiosis/spermiogenesis associated 1
C9orf24	-1.18449	0.003995	0.015963	-2.87858	Down	chromosome 9 open reading frame 24
SPATA17	-1.18378	0.000318	0.002201	-3.60012	Down	spermatogenesis associated 17
C22orf23	-1.18097	0.000609	0.003639	-3.42752	Down	chromosome 22 open reading frame 23
EYA1	-1.17966	0.002377	0.010695	-3.03852	Down	EYA transcriptional coactivator and phosphatase 1
SAXO2	-1.17876	0.001244	0.006352	-3.22855	Down	stabilizer of axonemal microtubules 2
SLC9C2	-1.17675	0.000687	0.003993	-3.39472	Down	solute carrier family 9 member C2 (putative)
SERPINB2	-1.17644	0.00043	0.002783	-3.52097	Down	serpin family B member 2
CFAP58	-1.17636	1.17E-05	0.000158	-4.38251	Down	cilia and flagella associated protein 58

CFAP276	-1.17603	0.006634	0.023824	-2.71467	Down	cilia and flagella associated protein 276
HHLA2	-1.17311	0.000367	0.002457	-3.56305	Down	HERV-H LTR-associating 2
SPINK1	-1.17164	0.000126	0.001075	-3.83393	Down	serine peptidase inhibitor Kazal type 1
TP53AIP1	-1.17143	0.009439	0.03137	-2.59574	Down	tumor protein p53 regulated apoptosis inducing protein 1
CFAP73	-1.16955	0.005474	0.020459	-2.77772	Down	cilia and flagella associated protein 73
FHAD1	-1.16916	1.18E-05	0.000159	-4.38079	Down	forkhead associated phosphopeptide binding domain 1
DOC2A	-1.16838	9.22E-05	0.000837	-3.91017	Down	double C2 domain alpha
AK8	-1.16742	4.22E-05	0.00044	-4.09531	Down	adenylate kinase 8
CFAP77	-1.16694	0.008641	0.029367	-2.62592	Down	cilia and flagella associated protein 77
TEKT3	-1.16139	5.50E-06	8.64E-05	-4.54481	Down	tektin 3
PADI1	-1.15872	0.006604	0.023739	-2.71618	Down	peptidyl arginine deiminase 1
TEX26	-1.15645	0.004312	0.016899	-2.85438	Down	testis expressed 26
ADH6	-1.15524	0.006078	0.022267	-2.74355	Down	alcohol dehydrogenase 6 (class V)
SYT8	-1.15473	0.000413	0.002692	-3.53166	Down	synaptotagmin 8
STOX1	-1.1533	5.01E-05	0.000505	-4.05514	Down	storkhead box 1
CCDC181	-1.15284	9.16E-05	0.000832	-3.91177	Down	coiled-coil domain containing 181
CCDC113	-1.15014	0.000178	0.001392	-3.7482	Down	coiled-coil domain containing 113
B4GALNT2	-1.14954	0.004071	0.016182	-2.8726	Down	beta-1,4-N-acetyl-galactosaminyltransferase 2
ADGRF4	-1.14779	9.28E-06	0.000131	-4.43339	Down	adhesion G protein-coupled receptor F4
CERKL	-1.14748	0.00367	0.01496	-2.9052	Down	ceramide kinase like
PRR15	-1.14658	7.68E-05	0.000722	-3.95427	Down	proline rich 15
DTHD1	-1.14631	0.000279	0.001984	-3.63412	Down	death domain containing 1
CACNG6	-1.14249	0.005269	0.019879	-2.79012	Down	calcium voltage-gated channel auxiliary subunit gamma 6
TMEM59L	-1.13978	2.30E-06	4.32E-05	-4.72507	Down	transmembrane protein 59 like
C6	-1.13956	0.000394	0.002594	-3.54394	Down	complement C6
HABP2	-1.13756	1.02E-05	0.000141	-4.41367	Down	hyaluronan binding protein 2
CRISP2	-1.13533	0.012356	0.039083	-2.50181	Down	cysteine rich secretory protein 2
EFCAB10	-1.13448	0.003792	0.015326	-2.895	Down	EF-hand calcium binding domain 10
IQUB	-1.13375	5.00E-05	0.000505	-4.05547	Down	IQ motif and ubiquitin domain containing
KIF19	-1.13353	0.000501	0.003116	-3.48005	Down	kinesin family member 19
DKK1	-1.13148	3.05E-05	0.000337	-4.16992	Down	dickkopf WNT signaling pathway inhibitor 1
PACRG	-1.13086	0.000308	0.002144	-3.6088	Down	parkin coregulated
TMPRSS4	-1.128	0.006734	0.024116	-2.7097	Down	transmembrane serine protease 4
GSTA1	-1.12601	0.001897	0.008968	-3.10598	Down	glutathione S-transferase alpha 1
CNGA3	-1.12501	0.001433	0.007188	-3.18795	Down	cyclic nucleotide gated channel subunit alpha 3
TTC23L	-1.12483	0.00011	0.000966	-3.86674	Down	tetratricopeptide repeat domain 23 like
FAM166C	-1.12401	0.005399	0.020246	-2.78224	Down	family with sequence similarity 166 member C
CCDC40	-1.12323	1.87E-07	5.31E-06	-5.21164	Down	coiled-coil domain containing 40
ABCA12	-1.12219	0.005197	0.019675	-2.79459	Down	ATP binding cassette subfamily A member 12
CD164L2	-1.11777	0.000871	0.004806	-3.32929	Down	CD164 molecule like 2
CNTD1	-1.11691	0.00068	0.003959	-3.39754	Down	cyclin N-terminal domain containing 1
SNTN	-1.11613	0.009289	0.030996	-2.60124	Down	sentan, cilia apical structure protein
TP73	-1.1153	0.000772	0.004383	-3.36248	Down	tumor protein p73

EFHB	-1.11404	0.000506	0.003136	-3.47762	Down	EF-hand domain family member B
DNAH5	-1.11302	0.000316	0.002187	-3.60203	Down	dynein axonemal heavy chain 5
FOXJ1	-1.10527	0.001938	0.009125	-3.09964	Down	forkhead box J1
FAM83F	-1.1039	0.001099	0.005778	-3.2639	Down	family with sequence similarity 83 member F
KLHDC8A	-1.09928	2.24E-08	9.22E-07	-5.59257	Down	kelch domain containing 8A
DQX1	-1.09807	0.000133	0.001123	-3.82003	Down	DEAQ-box RNA dependent ATPase 1
EFCAB6	-1.09785	1.73E-05	0.000215	-4.29657	Down	EF-hand calcium binding domain 6
DNAI4	-1.09759	5.79E-05	0.000567	-4.0211	Down	dynein axonemal intermediate chain 4
BCAS1	-1.09707	3.88E-05	0.00041	-4.11475	Down	brain enriched myelin associated protein 1
KCNH3	-1.09646	0.000154	0.001245	-3.78533	Down	potassium voltage-gated channel subfamily H member 3
HRK	-1.09406	0.000449	0.002876	-3.50922	Down	harakiri, BCL2 interacting protein
KRT15	-1.0888	0.004994	0.019065	-2.80744	Down	keratin 15
CFAP95	-1.08817	0.011679	0.037394	-2.52171	Down	cilia and flagella associated protein 95
GDA	-1.088	0.001904	0.008999	-3.10477	Down	guanine deaminase
CCDC170	-1.08763	0.000115	0.001002	-3.85579	Down	coiled-coil domain containing 170
NEK5	-1.08554	0.00117	0.006055	-3.24617	Down	NIMA related kinase 5
SRCIN1	-1.08486	3.27E-05	0.000357	-4.15403	Down	SRC kinase signaling inhibitor 1
PLPPR3	-1.08439	0.000116	0.001004	-3.85492	Down	phospholipid phosphatase related 3
FLACC1	-1.08245	1.30E-05	0.000172	-4.35932	Down	flagellum associated containing coiled-coil domains 1
SLC44A4	-1.0824	7.81E-05	0.000731	-3.95013	Down	solute carrier family 44 member 4
CAPN13	-1.07803	0.000176	0.001379	-3.7511	Down	calpain 13
RIIAD1	-1.07622	0.001656	0.008044	-3.14581	Down	regulatory subunit of type II PKA R-subunit domain containing 1
PROM1	-1.07323	0.007456	0.026105	-2.67575	Down	prominin 1
DAPL1	-1.07169	0.000781	0.004423	-3.35935	Down	death associated protein like 1
STK33	-1.06761	3.21E-06	5.67E-05	-4.65706	Down	serine/threonine kinase 33
ZG16B	-1.06641	0.000223	0.001663	-3.69092	Down	zymogen granule protein 16B
PLEKHG7	-1.06558	0.004696	0.018155	-2.82716	Down	pleckstrin homology and RhoGEF domain containing G7
ST6GALNAC1	-1.06379	9.70E-05	0.000869	-3.89793	Down	ST6 N-acetylgalactosaminide alpha-2,6-sialyltransferase 1
PIERCE1	-1.06323	0.000714	0.004108	-3.38432	Down	piercer of microtubule wall 1
STRA6	-1.0627	0.005113	0.019417	-2.79981	Down	signaling receptor and transporter of retinol STRA6
ATG9B	-1.05961	2.82E-06	5.13E-05	-4.6837	Down	autophagy related 9B
IGFL2	-1.05744	0.010801	0.035002	-2.54906	Down	IGF like family member 2
CFAP54	-1.05536	0.000301	0.002114	-3.61409	Down	cilia and flagella associated protein 54
ADGRA1	-1.05248	0.010302	0.033689	-2.56551	Down	adhesion G protein-coupled receptor A1
IL5RA	-1.04946	0.000141	0.00117	-3.80676	Down	interleukin 5 receptor subunit alpha
PLCH2	-1.04808	0.00037	0.00247	-3.5607	Down	phospholipase C eta 2
CCDC146	-1.04496	5.75E-06	8.96E-05	-4.53555	Down	coiled-coil domain containing 146
ESPN	-1.04207	0.002398	0.010762	-3.03592	Down	espin
MS4A6E	-1.04063	0.000612	0.003653	-3.4261	Down	membrane spanning 4-domains A6E
AK7	-1.0404	0.000369	0.00247	-3.5612	Down	adenylate kinase 7
TRIM29	-1.03912	0.003545	0.014579	-2.91606	Down	tripartite motif containing 29
SLC27A2	-1.03893	0.000163	0.001296	-3.77111	Down	solute carrier family 27 member 2
FNDC11	-1.03681	1.84E-05	0.000225	-4.28338	Down	fibronectin type III domain containing 11

LRRC10B	-1.03593	0.001746	0.008397	-3.13028	Down	leucine rich repeat containing 10B
PIH1D2	-1.03507	9.85E-05	0.000878	-3.89437	Down	PIH1 domain containing 2
MYEOV	-1.03486	4.34E-06	7.22E-05	-4.5943	Down	myeloma overexpressed
C21orf58	-1.03222	7.77E-06	0.000115	-4.47142	Down	chromosome 21 open reading frame 58
MUC4	-1.03108	0.004543	0.017662	-2.83778	Down	mucin 4, cell surface associated
AKR1B10	-1.02869	0.010298	0.033683	-2.56565	Down	aldo-keto reductase family 1 member B10
NGEF	-1.02805	0.000166	0.00132	-3.76571	Down	neuronal guanine nucleotide exchange factor
FMO6P	-1.02723	0.00093	0.005063	-3.31082	Down	flavin containing dimethylaniline monoxygenase 6, pseudogene
LRRC73	-1.02529	0.000748	0.004279	-3.3713	Down	leucine rich repeat containing 73
MKRN2OS	-1.02094	0.000149	0.001216	-3.7925	Down	MKRN2 opposite strand
MNS1	-1.02025	9.44E-06	0.000133	-4.42967	Down	meiosis specific nuclear structural 1
WNK2	-1.02004	7.49E-05	0.000706	-3.96016	Down	WNK lysine deficient protein kinase 2
MAP7D2	-1.01976	1.42E-05	0.000183	-4.34095	Down	MAP7 domain containing 2
TFF3	-1.01964	1.17E-05	0.000158	-4.38226	Down	trefoil factor 3
SMKR1	-1.01824	0.000989	0.005309	-3.2936	Down	small lysine rich protein 1
FUT2	-1.01568	0.005212	0.019714	-2.79362	Down	fucosyltransferase 2
NEK11	-1.01463	1.26E-07	3.92E-06	-5.28403	Down	NIMA related kinase 11
GBP6	-1.01023	0.002269	0.010363	-3.05252	Down	guanylate binding protein family member 6
AWAT2	-1.0091	0.009876	0.032584	-2.58015	Down	acyl-CoA wax alcohol acyltransferase 2
LGSN	-1.00862	0.009519	0.031578	-2.59285	Down	lengsin, lens protein with glutamine synthetase domain
LRGUK	-1.00809	2.45E-05	0.000282	-4.2191	Down	leucine rich repeats and guanylate kinase domain containing
OXTR	-1.00701	0.00111	0.005819	-3.26095	Down	oxytocin receptor
BBOX1	-1.00514	0.006213	0.022665	-2.7363	Down	gamma-butyrobetaine hydroxylase 1
CRLF1	-1.00066	9.46E-07	2.06E-05	-4.9025	Down	cytokine receptor like factor 1
PLPP2	-0.99867	0.000111	0.000968	-3.86577	Down	phospholipid phosphatase 2
FAM83B	-0.99858	0.010338	0.033762	-2.56431	Down	family with sequence similarity 83 member B
TTLL6	-0.99754	0.000662	0.003874	-3.40497	Down	tubulin tyrosine ligase like 6
CCL24	-0.99685	0.000854	0.00474	-3.33472	Down	C-C motif chemokine ligand 24
KIF6	-0.99482	6.42E-05	0.000621	-3.99672	Down	kinesin family member 6
KIF24	-0.99098	6.44E-06	9.88E-05	-4.51136	Down	kinesin family member 24
KPNA7	-0.99077	0.000135	0.00113	-3.81773	Down	karyopherin subunit alpha 7
IQCG	-0.98968	5.78E-07	1.36E-05	-4.99827	Down	IQ motif containing G
HCAR1	-0.988	0.002418	0.010826	-3.03339	Down	hydroxycarboxylic acid receptor 1
GJB3	-0.98636	0.001062	0.005621	-3.27361	Down	gap junction protein beta 3
MMP19	-0.98401	1.40E-05	0.000182	-4.34371	Down	matrix metallopeptidase 19
GRIK2	-0.98255	0.000761	0.004331	-3.36676	Down	glutamate ionotropic receptor kainate type subunit 2
CFAP161	-0.97917	0.000312	0.00217	-3.60487	Down	cilia and flagella associated protein 161
LRRIQ1	-0.97829	0.007501	0.026226	-2.67373	Down	leucine rich repeats and IQ motif containing 1
B3GNT3	-0.97384	0.002348	0.010595	-3.04224	Down	UDP-GlcNAc:betaGal beta-1,3-N-acetylglucosaminyltransferase 3
SERPINI2	-0.97241	0.000695	0.004023	-3.39173	Down	serpin family I member 2
SLC52A1	-0.97049	0.001305	0.006633	-3.21498	Down	solute carrier family 52 member 1
TPPP3	-0.96586	2.22E-05	0.000261	-4.24107	Down	tubulin polymerization promoting protein family member 3
PPIL6	-0.96304	0.000556	0.003384	-3.45232	Down	peptidylprolyl isomerase like 6

H2BC26	-0.96143	0.003048	0.012976	-2.96284	Down	H2B clustered histone 26
CP	-0.96137	0.000819	0.004595	-3.34633	Down	ceruloplasmin
ANKK1	-0.96132	0.000322	0.002219	-3.59714	Down	ankyrin repeat and kinase domain containing 1
GSTA3	-0.96087	0.015122	0.046014	-2.42944	Down	glutathione S-transferase alpha 3
GLB1L3	-0.95966	0.015105	0.045972	-2.42985	Down	galactosidase beta 1 like 3
CHST6	-0.95939	0.001305	0.006634	-3.21483	Down	carbohydrate sulfotransferase 6
TMEM184A	-0.95888	9.31E-06	0.000132	-4.4326	Down	transmembrane protein 184A
CLDN16	-0.95866	0.007814	0.02711	-2.66002	Down	claudin 16
IQCH	-0.95788	3.00E-05	0.000332	-4.17371	Down	IQ motif containing H
TMEM231	-0.9565	2.98E-05	0.000331	-4.17518	Down	transmembrane protein 231
PKP2	-0.95464	6.17E-06	9.53E-05	-4.52044	Down	plakophilin 2
CFAP119	-0.95261	7.98E-06	0.000117	-4.46562	Down	cilia and flagella associated protein 119
COL7A1	-0.95221	0.000335	0.002293	-3.58693	Down	collagen type VII alpha 1 chain
LHCGR	-0.95083	0.004024	0.016032	-2.8763	Down	luteinizing hormone/choriogonadotropin receptor
KCNRG	-0.94981	0.003161	0.013339	-2.95163	Down	potassium channel regulator
EPN3	-0.94674	0.007764	0.026974	-2.66218	Down	epsin 3
AQP5	-0.94586	0.001798	0.008599	-3.12164	Down	aquaporin 5
NME5	-0.94235	0.000124	0.001063	-3.83767	Down	NME/NM23 family member 5
HNF4G	-0.94189	0.009498	0.031527	-2.5936	Down	hepatocyte nuclear factor 4 gamma
GRM7	-0.94082	0.0043	0.016875	-2.85524	Down	glutamate metabotropic receptor 7
CYP2J2	-0.94054	0.000266	0.001907	-3.64628	Down	cytochrome P450 family 2 subfamily J member 2
ALG1L1P	-0.93963	0.00312	0.013205	-2.95569	Down	ALG1 like 1, pseudogene
RAB3B	-0.93872	0.001739	0.008373	-3.13159	Down	RAB3B, member RAS oncogene family
BAIAP3	-0.9376	0.000125	0.001066	-3.83686	Down	BAI1 associated protein 3
SYT13	-0.93707	0.000652	0.003834	-3.40911	Down	synaptotagmin 13
CRABP2	-0.93442	0.000256	0.001849	-3.65635	Down	cellular retinoic acid binding protein 2
LRRC23	-0.93392	0.000157	0.001264	-3.78015	Down	leucine rich repeat containing 23
CCDC153	-0.93317	3.78E-05	0.000403	-4.12052	Down	coiled-coil domain containing 153
DNAAF11	-0.93289	4.02E-06	6.81E-05	-4.61031	Down	dynein axonemal assembly factor 11
KLK13	-0.92961	0.009131	0.030602	-2.60712	Down	kallikrein related peptidase 13
SAMD15	-0.92506	0.000928	0.005053	-3.31154	Down	sterile alpha motif domain containing 15
OMG	-0.92423	0.000255	0.001848	-3.65684	Down	oligodendrocyte myelin glycoprotein
SPACA9	-0.92267	2.62E-05	0.000298	-4.20446	Down	sperm acrosome associated 9
CABCOCO1	-0.92177	0.001385	0.006976	-3.19767	Down	ciliary associated calcium binding coiled-coil 1
LRRC56	-0.9206	8.93E-06	0.000128	-4.44146	Down	leucine rich repeat containing 56
PPP1R36	-0.91998	8.66E-05	0.000795	-3.92549	Down	protein phosphatase 1 regulatory subunit 36
DEGS2	-0.91768	0.00084	0.004692	-3.33925	Down	delta 4-desaturase, sphingolipid 2
GIPR	-0.91692	4.01E-05	0.000421	-4.10684	Down	gastric inhibitory polypeptide receptor
KIAA0319	-0.91661	0.0003	0.002104	-3.61554	Down	KIAA0319
CCDC81	-0.9128	0.001112	0.005823	-3.26055	Down	coiled-coil domain containing 81
CAPS	-0.91114	0.000927	0.005052	-3.31175	Down	calcyphosine
ZCCHC12	-0.91021	0.000843	0.004698	-3.33838	Down	zinc finger CCHC-type containing 12
CCDC157	-0.90985	1.25E-06	2.63E-05	-4.84801	Down	coiled-coil domain containing 157

MORN3	-0.90908	0.000183	0.001415	-3.74199	Down	MORN repeat containing 3
INSYN2A	-0.90908	0.001007	0.005382	-3.28864	Down	inhibitory synaptic factor 2A
ELF3	-0.90836	0.000135	0.00113	-3.8178	Down	E74 like ETS transcription factor 3
RAB36	-0.90607	0.000118	0.001017	-3.85125	Down	RAB36, member RAS oncogene family
TSGA10	-0.90503	0.000263	0.00189	-3.64964	Down	testis specific 10
CSMD1	-0.90479	0.000627	0.003719	-3.41964	Down	CUB and Sushi multiple domains 1
ZDHHC11	-0.90465	8.97E-07	1.99E-05	-4.91309	Down	zinc finger DHHC-type containing 11
ARHGAP39	-0.90445	1.51E-05	0.000193	-4.32788	Down	Rho GTPase activating protein 39
TMEM232	-0.90113	0.003606	0.014768	-2.91068	Down	transmembrane protein 232
KLHL32	-0.89377	0.002938	0.012613	-2.97417	Down	kelch like family member 32
KLK14	-0.89277	0.000229	0.001691	-3.685	Down	kallikrein related peptidase 14
SRGAP3	-0.89163	3.70E-05	0.000398	-4.12545	Down	SLIT-ROBO Rho GTPase activating protein 3
CDH3	-0.89152	0.000211	0.001581	-3.70587	Down	cadherin 3
SLC28A3	-0.88983	0.000152	0.001235	-3.78784	Down	solute carrier family 28 member 3
GPR156	-0.88904	9.99E-05	0.000888	-3.8909	Down	G protein-coupled receptor 156
SPDEF	-0.88422	0.003353	0.013967	-2.93341	Down	SAM pointed domain containing ETS transcription factor
MAK	-0.88349	0.001961	0.009208	-3.096	Down	male germ cell associated kinase
WNK4	-0.8826	0.011778	0.03764	-2.51872	Down	WNK lysine deficient protein kinase 4
LEKR1	-0.88245	0.000468	0.00296	-3.49862	Down	leucine, glutamate and lysine rich 1
NHLRC4	-0.8819	0.000333	0.002287	-3.58798	Down	NHL repeat containing 4
RGS22	-0.88159	0.000513	0.00317	-3.47386	Down	regulator of G protein signaling 22
CCDC96	-0.88148	0.000237	0.001739	-3.67578	Down	coiled-coil domain containing 96
EFNB3	-0.88045	0.000195	0.001486	-3.72532	Down	ephrin B3
TTC21A	-0.87693	5.90E-07	1.38E-05	-4.99437	Down	tetratricopeptide repeat domain 21A
DEFB124	-0.87665	0.001071	0.005659	-3.27129	Down	defensin beta 124
SCG5	-0.87254	0.000219	0.001634	-3.69646	Down	secretogranin V
CYP27C1	-0.87203	0.007185	0.025388	-2.68817	Down	cytochrome P450 family 27 subfamily C member 1
CCDC30	-0.86754	1.87E-06	3.70E-05	-4.76658	Down	coiled-coil domain containing 30
TRPV6	-0.86561	0.000491	0.003074	-3.48574	Down	transient receptor potential cation channel subfamily V member 6
SLC4A11	-0.86245	0.000692	0.004017	-3.39255	Down	solute carrier family 4 member 11
LCA5L	-0.86201	0.000185	0.001427	-3.73905	Down	lebercilin LCA5 like
ALDH3A1	-0.8602	0.009274	0.030957	-2.60179	Down	aldehyde dehydrogenase 3 family member A1
CLDN10	-0.85879	0.000336	0.002304	-3.58549	Down	claudin 10
BAAT	-0.8557	0.0037	0.015053	-2.90265	Down	bile acid-CoA:amino acid N-acyltransferase
SPTBN2	-0.85483	2.32E-05	0.00027	-4.23181	Down	spectrin beta, non-erythrocytic 2
VSTM2L	-0.85454	9.70E-05	0.000869	-3.89794	Down	V-set and transmembrane domain containing 2 like
SALL4	-0.8545	0.000271	0.001934	-3.64191	Down	spalt like transcription factor 4
SLC22A4	-0.85099	0.000367	0.002457	-3.563	Down	solute carrier family 22 member 4
SYT12	-0.84986	0.004634	0.017964	-2.83145	Down	synaptotagmin 12
PPP1R32	-0.84947	1.59E-05	0.000201	-4.31571	Down	protein phosphatase 1 regulatory subunit 32
SERPINE1	-0.84896	0.004492	0.017481	-2.84138	Down	serpin family E member 1
COL28A1	-0.84895	0.000491	0.003074	-3.48587	Down	collagen type XXVIII alpha 1 chain
BCO2	-0.84871	3.90E-07	9.83E-06	-5.0737	Down	beta-carotene oxygenase 2

SLC51A	-0.84745	0.009252	0.030913	-2.60258	Down	solute carrier family 51 subunit alpha
OGDHL	-0.84526	0.015315	0.046478	-2.42485	Down	oxoglutarate dehydrogenase L
CES4A	-0.84525	6.81E-07	1.57E-05	-4.96684	Down	carboxylesterase 4A
COMP	-0.84509	0.003913	0.01571	-2.88512	Down	cartilage oligomeric matrix protein
SPATA18	-0.84457	0.001466	0.00731	-3.18131	Down	spermatogenesis associated 18
DPP10	-0.84416	0.001498	0.007431	-3.17507	Down	dipeptidyl peptidase like 10
BBOF1	-0.84411	7.73E-05	0.000725	-3.95251	Down	basal body orientation factor 1
DZIP1L	-0.84411	4.78E-08	1.73E-06	-5.45922	Down	DAZ interacting zinc finger protein 1 like
H2BC8	-0.84364	0.000157	0.001263	-3.78039	Down	H2B clustered histone 8
OSCP1	-0.84193	2.00E-05	0.00024	-4.2646	Down	organic solute carrier partner 1
CCDC180	-0.84176	2.18E-05	0.000257	-4.2453	Down	coiled-coil domain containing 180
TTC34	-0.84166	1.88E-05	0.000229	-4.27831	Down	tetratricopeptide repeat domain 34
MLIP	-0.84099	0.005087	0.019349	-2.80145	Down	muscular LMNA interacting protein
CLCNKA	-0.84099	2.63E-05	0.000298	-4.20358	Down	chloride voltage-gated channel Ka
HCN4	-0.84094	0.009377	0.031218	-2.598	Down	hyperpolarization activated cyclic nucleotide gated potassium channel 4
TRIM17	-0.83855	1.51E-06	3.10E-05	-4.80963	Down	tripartite motif containing 17
H2AC8	-0.83851	0.000477	0.003008	-3.49307	Down	H2A clustered histone 8
SMIM5	-0.83732	3.61E-05	0.00039	-4.13116	Down	small integral membrane protein 5
ZMYND12	-0.83364	9.52E-05	0.000858	-3.9025	Down	zinc finger MYND-type containing 12
LCNL1	-0.83337	9.16E-06	0.00013	-4.43599	Down	lipocalin like 1

Table S2 The enriched GO terms of the up and down regulated differentially expressed genes.

GO ID	CATEGORY	GO Name	adjusted_p_value	negative_log10_of_adjusted_p_value	Gene Count	Gene
			Up regulated genes			
GO:0050896	BP	response to stimulus	5.27E-18	17.27803883	288	OLFM4,DEFA4,RTKN2,CYP1A1,HHIP,AGTR2,MAP3K15,ORM2,PRTN3,WFDC12,DEFA3,RNASE3,SCN1A,PI3,CX3CR1,S100A12,TUBB1,PROK2,CSF2,CD177,ANKRD1,AGRP,SLC39A8,ADRA1A,MS4A3,MS4A15,ADCY8,TRIM58,FGG,AGER,LHX9,PF4,FFAR2,ORM1,GP1BA,MPO,CD5L,SH3GL2,ADGRE1,HMGCS2,RXFP1,ITGA2B,F11,S100A8,CHRM1,PGLYRP1,BRINP1,GPM6A,GRIA1,NPR3,VEGFD,CHRM2,MPIG6B,CXCL11,LAMP3,GPR15,SHISA2,KCTD16,IL1A,LILRA1,ND6,CBS,CLDN18,WNT7A,NPFFR1,NFE2,BPI,BTNL8,SSTR1,PRKG2,AQP4,USP44,SCD,LRP2,TRIM71,SLC6A4,MME,BDNF,ZNF385B,NRGN,GCOM1,CXCL10,ODAM,CPB2,ANGPTL7,CASP5,CCL8,PRODH,CLTRN,TMEM100,P2RY1,RNASE2,CSPG5,LRRN4,VEPH1,FCGR3B,S100A9,NPY1R,MARCKS,IL1B,CXCR1,PLPPR1,CXCR2,GPIHBP1,AFF3,TNNT1,WNT3A,SP5,APOH,FZD5,CHRM3,CTNND2,BMI1,PCARE,CYP1B1,FCN3,TTN,NIM1K,SHISA3,AZGP1,GPAM,STC1,ABCA3,GBP4,CD36,ED

						NRB,MYZAP,MACROD2,PLA2G3,BTNL9,CEBPA,TRIB3,GPR18,SLC46A2,CNTN6,FMO5,TNNC1,ANGPT4,SPX,MAP3K21,SYNPO2L,CCDC141,PCSK9,ACVR1C,SH2D1B,PRTG,P2RY13,CDKL2,KITLG,GPD1,CDH13,BATF2,FFAR4,PTPRQ,FIBIN,GPX3,RAPGEF6,LTB,LILRA2,FGF2,ACKR4,CSF3R,MMP25,NDUFC2,MAP1LC3C,FUT7,FASN,PIK3AP1,NKD1,KBTBD7,GPR160,FOXD1,SLC1A1,RXRG,LILRA5,SHH,DGAT2,DACH1,PNPLA3,FGF9,HMSD,SLC7A11,CLIC5,SGPP1,MGAT3,CCDC68,MTARC1,OLFML2A,HSPA6,TSPAN12,SCAI,VIP,IGSF6,GMFB,PLLP,SH3GL3,KLRF1,KCNJ2,TRHDE,ADGRE3,KL,UBE2D1,SMAD6,BMPR2,APOA1,CALCRL,TGFBR3,INSIG1,RASGRF1,LRRK2,ATP8A2,CD244,TLR3,CAV2,HLX,CLEC4D,KCNMB4,NLRP12,ADRB1,OCLN,TNFSF10,SPOCK2,SLC22A3,CA2,GCSAML,TMEM170B,PAPSS2,EMP2,SNX10,LIFR,HPN,MNDA,TLR8,CMPK2,GATA3,RNASE4,RSPO2,CCR2,IFIT3,NEK7,EPB41L5,DIPK2A,MARCHF1,CD274,SPRING1,DDIAS,GUCY1A2,TLR7,MFSD2A,RALA,DCC,CCRL2,PRAM1,BEX1,BMPER,ARHGAP29,DUSP26,ACP3,EFNB2,CCBE1,FZD8,CAV1,UBE2W,ARRB1,TRPC3,CR1,WWC2,GPR82,PEG10,ANOS1,PPP3R1,DLL4,MEFV,TMC2,ANXA3,TFPI,MAPT,ASGR2,CXADR,EPAS1,ADGRG2,TBX3,FADS1,DKK2,DNAJB14,GPER1,SUCNR1,PCLAF
GO:0065007	BP	biological regulation	3.86E-09	8.413646903	334	OLFM4,NECAB1,RTKN2,CYP1A1,HHIP,AGTR2,MAP3K15,ORM2,PRTN3,WFDC12,DEFA3,SCN1A,HSD17B6,PI3,CX3CR1,S100A12,TUBB1,PROK2,CSF2,CD177,ANKRD1,AGRP,SLC39A8,ADRA1A,MS4A3,MS4A15,ADCY8,TRIM58,FGG,AGER,LHX9,PF4,FFAR2,ORM1,GP1BA,MPO,CD5L,SH3GL2,ADGRE1,RXFP1,ITGA2B,F11,S100A8,CHRM1,PGLYRP1,BRINP1,ZBED2,GPM6A,GRIA1,NPR3,VEGFD,CHRM2,MPIG6B,CXCL11,LAMP3,GPR15,SHISA2,KCTD16,IL1A,LILRA1,CBS,CLDN18,WNT7A,NPFFR1,NFE2,BPI,BTNL8,SSTR1,PRKG2,AQP4,USP44,PLA2G4E,SCD,LRP2,TRIM71,SLC6A4,MME,BDNF,ZNF385B,MYRF,NRGN,GCOM1,GFI1B,LRRN3,CXCL10,ODAM,CPB2,RPH3A,KCNS1,ANGPTL7,TMEM97,PTCRA,CASP5,LGI3,CCL8,PRODH,CLTRN,TMEM100,P2RY1,CSPG5,LRRTM2,VEPH1,FCGR3B,S100A9,NPY1R,IL1B,CXCR1,PLPPR1,CXCR2,GPIHBP1,AFF3,TNNT1,WNT3A,PARD6B,SP5,APOH,FZD5,CHRM3,ACADL,CTNND2,BMI1,CYP1B1,FCN3,TTN,NIM1K,SHISA3,AZGP1,RIPPLY3,GPAM,STC1,ABCA3,ELOVL3,CD36,CACNA2D3,CACNA2D2,EDNRB,MYZAP,PLA2G3,BTNL9,CEBPA,T

GO:0071944	CC	cell periphery	2.59E-27	26.58672363	245	RIB3,GPR18,SLC46A2,CNTN6,FMO5,TNNC1,ANGPT4,SPX,MAP3K21,SYNPO2L,ATP8A1,PCSK9,ACVR1C,TDRD9,RNF128,SH2D1B,PRTG,P2RY13,IER3IP1,CDKL2,KITLG,GPD1,CDH13,BATF2,DPP6,FFAR4,FIBIN,RAPGEF6,LTB,LILRA2,FGF2,ACKR4,CSF3R,NDUFC2,TSPAN13,P3H2,FUT7,PLAG1,UPK3B,PIK3AP1,NKD1,ADH1B,STXBP6,KBTBD7,GPR160,FOXD1,SLC1A1,RXRG,LILRA5,SHH,DGAT2,DACH1,SRP9,FGF9,HMSD,SLC7A11,CLIC5,SGPP1,MGAT3,CCDC68,OLFML2A,PHGDH,TSPAN12,SCAI,KCNA4,HOXD1,PCDH17,VIP,IGSF6,GMFB,SCOC,SH3GL3,SDR16C5,KLRF1,KCNJ2,TRHDE,ADGRE3,KL,UBE2D1,ADH1A,SMAD6,ELF5,KHDRBS2,BMPR2,APOA1,QKI,NEGR1,CALCRL,TGFBR3,INSIG1,RASGRF1,LRRK2,ATP8A2,CD244,TLR3,CAV2,HLX,CLEC4D,KCNMB4,NLRP12,ADRB1,OCLN,TNFSF10,SPOCK2,SUSD2,SLC22A3,SPIN4,CA2,GCSAML,TMEM170B,PAPSS2,EMP2,GPM6B,LIFR,PHACTR1,HPN,RIMS4,MNDA,TLR8,GTF2A1,GATA3,DCXR,RSPO2,CCR2,IFIT3,NEK7,TMEM41B,NKAPL,EPB41L5,DIPK2A,VGLL3,CD274,SPRING1,DDIAS,STARD4,GUCY1A2,CHPT1,TLR7,MFSD2A,RALA,DCC,CCRL2,PRAM1,BEX1,BMPER,ARHGAP29,PDP2,DUSP26,KANK4,ACP3,EFNB2,CCBE1,ZBTB41,PKIA,HSD17B12,FZD8,CAV1,DHCR7,SP6,ANP32E,ID4,ARRB1,TRPC3,CR1,WWC2,GPR82,ZBTB6,HECW2,PEG10,ANOS1,PPP3R1,DLL4,CFL2,MEFV,TMC2,ANXA3,TFPI,MAPT,ASGR2,EDIL3,C21ORF91,SLITRK2,CXADR,IRX1,EPAS1,ADGRG2,RNF144B,TBX3,IRX2,FADS1,DKK2,GPER1,SUCNR1,PCLAF SLCO1A2,OLFM4,RTKN2,HHIP,AGTR2,ORM2,PRTN3,SCN1A,PI3,CX3CR1,S100A12,CSF2,ALPP,CD177,CADM2,SLC39A8,ADRA1A,MS4A3,MS4A15,ADCY8,FGG,AGER,PF4,FFAR2,ORM1,GP1BA,CD5L,SLCO4C1,SH3GL2,ADGRE1,SLC5A9,RXFP1,ITGA2B,F11,S100A8,CHRM1,GPM6A,GRIA1,NPR3,MUCL3,CHRM2,MPIG6B,LAMP3,GPR15,KCTD16,IL1A,LILRA1,CLDN18,WNT7A,NPFFR1,BTNL8,SSTR1,PRKG2,AQP4,PLA2G4E,FOLR3,SLC19A3,LRP2,SLC6A4,MME,NRGN,GFI1B,LRRN3,CXCL10,ODAM,RPH3A,KCNS1,ANGPTL7,TMEM97,CLTRN,TMEM100,P2RY1,CSPG5,LRRTM2,GPRIN2,LRRN4,VEPH1,SIGLEC11,FCGR3B,S100A9,NPY1R,MARCKS,MSMO1,CXCR1,PLPPR1,CXCR2,GPIHBP1,PCDH9,WNT3A,PARD6B,CA4,APOH,SGCG,FZD5,CHRM3,CTNND2,PRSS21,FCN3,TTN,AZGP1,GPAM,STC1,ABCA3,GBP4,CD36,CACNA2D3,CACNA2D2,VSIG10,EDNRB,MYZAP,MGAM,PLA2G

						3,BTNL9,C2ORF88,TRIB3,GPR18,SLC46A2,CNTN6,ANGPT4,ATP8A1,CCDC141,PCSK9,ACVR1C,PRTG,P2RY13,KITLG,CDH13,DPP6,FFAR4,PTPRQ,RAPGEF6,LTB,LILRA2,PDZD2,ACKR4,CSF3R,MMP25,NDUFC2,SLC26A8,TSPAN13,P3H2,FASN,UPK3B,PIK3AP1,NKD1,ADH1B,STXBP6,GPR160,SLC1A1,LILRA5,SHH,SLC7A11,EFR3B,CLIC5,SGPP1,OLFML2A,HSPA6,TSPAN12,KCNA4,PI4K2B,PCDH17,IGSF6,SDR16C5,KLRF1,KCNJ2,TRHDE,ADGRE3,KL,ADH1A,BMPR2,GCA,APOA1,NEGR1,CALCRL,TGFBR3,RASGRF1,LRRK2,ATP8A2,CD244,TLR3,CAV2,CLEC4D,KCNMB4,ADRB1,OCLN,VNN2,TNFSF10,SPOCK2,SUSD2,SLC22A3,CA2,TMEM170B,EMP2,GPM6B,LIFR,HPN,RIMS4,TLR8,DCXR,CCR2,EPB41L5,AP1S2,MARCHF1,CD274,TLR7,MFSD2A,RALA,DCC,CCRL2,PRAM1,TMEM47,SIGLEC14,MAL2,BMPER,SCAMP1,ACP3,EFNB2,CCBE1,HSD17B12,FZD8,CAV1,FREM2,ARRB1,CLCN4,TRPC3,CR1,GPR82,ERVMER34-1,ANOS1,PPP3R1,DLL4,TMC2,ANXA3,TFPI,MAPT,ASGR2,SLC33A1,EDIL3,MATN3,SLITRK2,CXADR,ADGRG2,PHACTR2,GPER1,SUCNR1
GO:0016020	CC	membrane	4.24E-20	19.37225431	306	SLCO1A2,OLFM4,RTKN2,CYP1A1,HHIP,AGTR2,PRTN3,GALNT13,SCN1A,HSD17B6,PI3,CX3CR1,S100A12,CSF2,ALPP,CD177,CADM2,SLC39A8,ADRA1A,MS4A3,MS4A15,ADCY8,FGG,ZDHHC19,AGER,FFAR2,SERTM1,MGAM2,GP1BA,CD5L,SLCO4C1,SH3GL2,ADGRE1,SLC5A9,RXFP1,ITGA2B,F11,S100A8,SFTPA2,CHRM1,SNX22,GPM6A,GRIA1,NPR3,MUCL3,VEGFD,CHRM2,MPIG6B,LAMP3,GPR15,SHISA2,KCTD16,KLRG2,IL1A,LILRA1,ND6,CLDN18,WNT7A,NPFFR1,SFTPA1,BPI,BTNL8,SSTR1,PRKG2,AQP4,PLA2G4E,FOLR3,SCD,SLC19A3,LRP2,SLC6A4,MME,RNF182,MYRF,NRGN,GFI1B,PEBP4,LRRN3,CXCL10,RPH3A,KCNS1,TMEM97,PTCRA,PRODH,CLTRN,TMEM100,P2RY1,CSPG5,LRRTM2,GPRIN2,LRRN4,VEPH1,SIGLEC11,FCGR3B,S100A9,NPY1R,MARCKS,MSMO1,CXCR1,PLPPR1,CXCR2,GPIHBP1,SAMD5,PCDH9,WNT3A,PARD6B,TMED7,CA4,SGCG,FZD5,CHRM3,ACADL,CTNND2,PRSS21,CYP1B1,FCN3,TTN,SHISA3,AZGP1,GPAM,STC1,ABCA3,ST8SIA6,ELOVL3,GBP4,CD36,CACNA2D3,CACNA2D2,VSIG10,EDNRB,MYZAP,MGAM,PLA2G3,BTNL9,C2ORF88,TRIB3,GPR18,SLC46A2,CNTN6,FMO5,ATP8A1,CCDC141,PCSK9,ACVR1C,RNF128,PRTG,P2RY13,IER3IP1,KITLG,CDH13,DPP6,FFAR4,PTPRQ,AATK,RAPGEF6,TLCD4,LTB,LILRA2

						,PDZD2,ACKR4,CSF3R,MMP25,NDUFC2,MAP1LC3C,SLC26A8,TSPAN13,FUT7,FASN,UPK3B,PIK3AP1,TMEM26,NKD1,ADH1B,STXBP6,GPR160,SLC1A1,LILRA5,SHH,DGAT2,PNPLA3,SRP9,SLC7A11,EFR3B,CLIC5,SGPP1,MGAT3,MTARC1,HSPA6,TSPAN12,SCAI,KCNA4,PI4K2B,PCDH17,IGSF6,PLLP,SCOC,SH3GL3,SDR16C5,KLRF1,KCNJ2,TRHDE,LRRC25,ADGRE3,KL,ADH1A,ANKRD29,BMPR2,GCA,APOA1,NEGR1,CALCRL,TGFBR3,INSIG1,RASGRF1,LRRK2,FAM241A,ATP8A2,CD244,TLR3,CAV2,CLEC4D,KCNMB4,ADRB1,OCLN,VNN2,TNFSF10,SUSD2,SLC22A3,CA2,TMEM170B,ST6GALNAC5,EMP2,GPM6B,C10ORF105,SNX10,LIFR,HPN,RIMS4,LILRB1,TLR8,DCXR,CCR2,TMEM41B,EPB41L5,AP1S2,DIPK2A,MARCHF1,CD274,SPRING1,B3GALT2,CHPT1,TLR7,MFSD2A,RALA,DCC,CCRL2,PRAM1,TMEM47,SIGLEC14,MAL2,SCAMP1,TMEM139,SMCO3,ACP3,EFNB2,HPCAL1,HSD17B12,FZD8,CAV1,DHCR7,CASD1,UBE2W,FREM2,SLC25A46,SC5D,ARRB1,CLCN4,TRPC3,CR1,B3GALNT1,GPR82,ERVMER34-1,PEG10,ANOS1,PPP3R1,DLL4,TMC2,ANXA3,TFPI,MYLK4,MAPT,C1GALT1,ASGR2,C19ORF38,SLC33A1,EBP,SLITRK2,CXADR,ADGRG2,RNF144B,FADS1,DNAJB14,PHACTR2,PXMP4,GPER1,SUCNR1
GO:0005102	MF	signaling receptor binding	0.000101538	3.993371364	68	AGTR2,PRTN3,CX3CR1,S100A12,PROK2,CSF2,CD177,AGRP,FGG,PF4,ITGA2B,S100A8,VEGFD,CXCL11,IL1A,WNT7A,BTNL8,BDNF,CXCL10,ANGPTL7,CCL8,P2RY1,CSPG5,LRRTM2,S100A9,IL1B,WNT3A,STC1,CD36,EDNRB,BTNL9,ANGPT4,SPX,PCSK9,KITLG,LTB,FGF2,PIK3AP1,SHH,FGF9,VIP,GMFB,KL,SMAD6,APOA1,TGFBR3,RASGRF1,CD244,CLEC4D,ADRB1,TNFSF10,EMP2,LIFR,GATA3,RSPO2,CCR2,MARCHF1,RALA,CCRL2,BEX1,EFNB2,FZD8,CAV1,ARRB1,DLL4,EDIL3,CXADR,DKK2
GO:0060089	MF	molecular transducer activity	0.001447579	2.839357788	60	AGTR2,CX3CR1,ADRA1A,AGER,FFAR2,GP1BA,ADGRE1,RXFP1,CHRM1,PGLYRP1,GRIA1,NPR3,CHRM2,GPR15,LILRA1,NPFFR1,SSTR1,FOLR3,P2RY1,FCGR3B,NPY1R,CXCR1,CXCR2,FZD5,CHRM3,CD36,EDNRB,GPR18,ACVR1C,PRTG,P2RY13,FFAR4,LILRA2,ACKR4,CSF3R,GPR160,RXRG,LILRA5,IGSF6,KLRF1,ADGRE3,BMPR2,CALCRL,TGFBR3,CD244,TLR3,CLEC4D,ADRB1,LIFR,TLR8,CCR2,TLR7,DCC,CCRL2,FZD8,CR1,GPR82,ADGRG2,GPER1,SUCNR1
			**Down regulated genes**			
GO:0007017	BP	microtubule-based process	5.51E-46	45.2586316	98	MCIDAS,DEUP1,DRC1,DNAAF1,DNAI1,DNAH3,DNAH10,CFAP100,CFAP157,CFAP52,HOATZ,DNAH9,HYDIN,CFAP74,TEKT2,TEKT1,CF

						AP46,CFAP65,SPAG17,DNAJB13,CFAP53,DNAH12,CCDC65,ODAD1,TTLL9,DNAH11,CFAP43,SPAG6,RSPH1,CFAP47,SPEF1,DRC7,DNAH2,RP1,DNAAF6,DNAI3,GAS2L2,TTC29,DNAI2,CCDC187,EFHC2,TEKT4,STMND1,ZBBX,NEK10,DNAAF8,DNAH7,CFAP45,AGBL4,CFAP70,DNAH6,CCDC78,CFAP61,ANKFN1,ZMYND10,ODAD2,TCTE1,CFAP251,ENO4,ODAD3,MAPK15,RSPH4A,DYNLT4,CATSPERD,DNAAF3,ENKUR,TOGARAM2,ROPN1L,CLXN,ODAD4,DYNLRB2,DAW1,RSPH9,MEIG1,CFAP58,CFAP73,TEKT3,KIF19,CCDC40,DNAH5,FOXJ1,DNAI4,CCDC170,PIERCE1,CFAP54,MNS1,MAP7D2,LRGUK,TTLL6,KIF6,KIF24,IQCG,TPPP3,NME5,DNAAF11,MAK,TTC21A,LCA5L
GO:0120036	BP	plasma membrane bounded cell projection organization	6.93E-25	24.15903131	93	MCIDAS,CCNO,DRC1,DNAAF1,DNAI1,CFAP100,CFAP157,CFAP52,HOATZ,HYDIN,CFAP74,TEKT2,TEKT1,CFAP46,CFAP65,SPAG17,DNAJB13,CFAP53,CCDC65,ODAD1,CFAP43,SPAG6,RSPH1,CFAP47,SPEF1,DRC7,DNAH2,RP1,CIBAR2,DNAAF6,DNAI3,TTC29,DNAI2,EFHC2,TEKT4,STMND1,DNAAF8,DNAH7,CFAP70,UBXN10,EDN2,CFAP61,CFAP126,ZMYND10,ODAD2,ENO4,ODAD3,MAPK15,RSPH4A,PIFO,DNAAF3,CLXN,ODAD4,DAW1,RSPH9,CATIP,MEIG1,CFAP58,CFAP73,TEKT3,CCDC113,IQUB,KIF19,DKK1,CCDC40,DNAH5,FOXJ1,DNAI4,SRCIN1,PIERCE1,CFAP54,ESPN,NGEF,MNS1,LRGUK,KIF24,IQCG,CFAP161,TMEM231,NME5,GRM7,CRABP2,DNAAF11,OMG,KIAA0319,MAK,CCDC96,EFNB3,TTC21A,LCA5L,VSTM2L,BBOF1,DZIP1L
GO:0042995	CC	cell projection	3.00E-45	44.52237057	154	DRC1,DNAAF1,DNAI1,DNAH3,DNAH10,CFAP100,CFAP157,CFAP52,HOATZ,NME9,DRD5,DNAH9,HYDIN,DRC3,CFAP74,AKAP14,TEKT2,TEKT1,CFAP46,CFAP65,SPAG17,DNAJB13,STOML3,CFAP53,DNAH12,CCDC65,ODAD1,TTLL9,CFAP107,DNAH11,CFAP43,AGBL2,DLEC1,ADGB,BDKRB1,SPAG6,RSPH1,GABRP,CFAP47,SPEF1,SPATA4,DRC7,DNAH2,RP1,CIBAR2,DNAI3,RIBC2,GAS2L2,TTC29,DNAI2,EFHC2,TEKT4,STMND1,FAM166B,ZBBX,CNGA4,CFAP141,DNAH7,CFAP45,SPAG8,AGBL4,CFAP70,DNAH6,UBXN10,CFAP61,CFAP126,DNAI7,ODAD2,TCTE1,CFAP251,SRD5A2,FAM183A,ENO4,ODAD3,MAPK15,RSPH4A,DYNLT4,CATSPERD,FANK1,UCN3,PIFO,C4ORF47,ENKUR,CFAP210,TOGARAM2,ROPN1L,IQCD,C5ORF49,CLXN,ODAD4,DYNLRB2,DAW1,RSPH9,SAXO2,CFAP58,CFAP276,CFAP73,DOC2A,AK8,CFAP77,TEKT3,SYT8,CCDC181,CCDC113,CERKL,IQUB,KIF19,PACRG,CNGA3,CCDC40,SNTN,E

						FHB,DNAH5,EFCAB6,DNAI4,CFAP95,CCDC170,SRCIN1,FLACC1,PROM1,PIERCE1,CFAP54,ESPN,AK7,NGEF,MNS1,LRGUK,OXTR,TTLL6,IQCG,CFAP161,ANKK1,TMEM231,CFAP119,AQP5,NME5,GRM7,SYT13,DNAAF11,SPACA9,CABCOCO1,LRRC56,TSGA10,SLC28A3,MAK,CCDC96,TTC21A,LCA5L,SPTBN2,VSTM2L,PPP1R32,BBOF1,DZIP1L,HCN4
GO:0005737	CC	cytoplasm	1.94E-15	14.71154083	308	MUC5AC,CYP2A6,SERPINB11,PCK1,BTG4,PITX1,ATP12A,SERPINB3,FRMPD2,CCNO,CYP2F1,KRT14,GSTA2,DEUP1,DRC1,FBXW10,DNAAF1,DNAI1,CFAP299,DNAH3,EPPIN,DNAH10,CFAP100,CFAP157,CRABP1,SEC14L3,MUC13,CFAP52,CYP2A13,HOATZ,NME9,ALOX15,LMNTD1,DNAH9,HYDIN,DRC3,CFAP74,AKAP14,TEKT2,MUC16,TEKT1,CFAP46,CFAP65,SPAG17,DNAJB13,CCNA1,SLC23A1,VWA3B,CAPSL,CCDC33,CFAP53,SIAH3,DNAH12,CCDC65,ODAD1,ABHD12B,TTLL9,CFAP107,DNAH11,EPGN,MB,LRRC43,CFAP43,AGBL2,DLEC1,ACSBG1,MROH9,ABCA13,BDKRB1,SPAG6,RSPH1,MYCBPAP,CFAP47,SPEF1,SPATA4,DRC7,DNAH2,KRT13,LRRC46,RP1,CIBAR2,DNAAF6,DNAI3,NWD1,RIBC2,GAS2L2,TTC29,DNAI2,TTLL10,EFHC2,FABP6,TEKT4,STMND1,FAM166B,FAM83A,CNGA4,CFAP141,DNAAF8,DCDC1,SCGB1A1,DNAH7,CFAP45,USP6,SPAG8,AGBL4,WDR93,CFAP70,DNAH6,CCDC78,UBXN10,GCNT3,CFAP61,CFAP126,ZMYND10,HHATL,TSPAN1,DNAI7,ODAD2,TSNAXIP1,TCTE1,CWH43,CFAP251,SRD5A2,ENO4,ODAD3,MAPK15,RSPH4A,DYNLT4,CHST9,B3GALT5,MMP3,FBXO15,FANK1,ERN2,PIFO,C4ORF47,SLC5A5,FUT6,DNAAF3,ENKUR,CFAP210,TOGARAM2,ROPN1L,IQCD,DIO1,CLXN,ODAD4,DYNLRB2,MAT1A,RSPH9,CATIP,MEIG1,C9ORF24,SPATA17,EYA1,SAXO2,SERPINB2,CFAP58,CFAP276,SPINK1,TP53AIP1,CFAP73,DOC2A,AK8,TEKT3,PADI1,TEX26,ADH6,SYT8,STOX1,CCDC181,CCDC113,B4GALNT2,CERKL,TMEM59L,IQUB,KIF19,DKK1,PACRG,TMPRSS4,GSTA1,CNGA3,TTC23L,CCDC40,ABCA12,CD164L2,CNTD1,TP73,EFHB,DNAH5,EFCAB6,DNAI4,BCAS1,HRK,KRT15,CFAP95,GDA,CCDC170,SRCIN1,FLACC1,CAPN13,PROM1,STK33,ST6GALNAC1,PIERCE1,ATG9B,CFAP54,PLCH2,CCDC146,ESPN,MS4A6E,AK7,TRIM29,SLC27A2,PIH1D2,MUC4,AKR1B10,NGEF,MNS1,WNK2,TFF3,FUT2,NEK11,GBP6,AWAT2,LGSN,LRGUK,BBOX1,CRLF1,PLPP2,FAM83B,TTLL6,KIF6,KIF24,KPNA7,IQCG,GJB3,CFAP161,B3GNT3,TPPP3,PPIL6,H2BC26,CP,ANKK1,GSTA3,

						GLB1L3,CHST6,TMEM184A,PKP2,CFAP119,COL7A1,LHCGR,KCNRG,EPN3,AQP5,HNF4G,GRM7,CYP2J2,RAB3B,BAIAP3,SYT13,CRABP2,LRRC23,DNAAF11,KLK13,SPACA9,CABCOCO1,DEGS2,KIAA0319,CCDC81,CAPS,ELF3,RAB36,TSGA10,ZDHHC11,ARHGAP39,KLK14,SRGAP3,CDH3,SLC28A3,MAK,WNK4,RGS22,CCDC96,SCG5,CYP27C1,LCA5L,ALDH3A1,CLDN10,BAAT,SPTBN2,VSTM2L,SALL4,SLC22A4,SYT12,PPP1R32,SERPINE1,COL28A1,BCO2,SLC51A,OGDHL,SPATA18,BBOF1,DZIP1L,H2BC8,OSCP1,HCN4,TRIM17
GO:0015631	MF	tubulin binding	5.74736E-06	5.240531874	27	CFAP157,TTLL9,DLEC1,SPAG6,SPEF1,SPATA4,RP1,GAS2L2,CCDC187,EFHC2,STMND1,DCDC1,SPAG8,AGBL4,DNAI7,PIFO,TOGARAM2,C9ORF24,SAXO2,CCDC181,KIF19,PACRG,CCDC170,TTLL6,KIF6,KIF24,TPPP3
GO:0005509	MF	calcium ion binding	0.147835087	0.830222478	26	CDHR4,CAPSL,EFCAB12,EFHC2,CDHR3,DNAH7,WDR49,CLXN,DOC2A,PADI1,SYT8,HABP2,EFCAB10,SNTN,EFHB,EFCAB6,CAPN13,PLCH2,GRM7,BAIAP3,SYT13,CABCOCO1,CAPS,CDH3,SYT12,COMP

Table S3 The enriched pathway terms of the up and down regulated differentially expressed genes.

Pathway ID	Pathway Name	adjusted_p_value	negative_log10_of_adjusted_p_value	Gene Count	Gene
		**Up regulated genes**			
REAC:R-HSA-500792	GPCR ligand binding	6.19E-07	6.208233279	38	AGTR2,CX3CR1,PROK2,ADRA1A,PF4,FFAR2,ADGRE1,RXFP1,CHRM1,CHRM2,CXCL11,WNT7A,NPFFR1,SSTR1,CXCL10,P2RY1,NPY1R,CXCR1,PLPPR1,CXCR2,WNT3A,FZD5,CHRM3,EDNRB,GPR18,P2RY13,FFAR4,ACKR4,SHH,VIP,ADGRE3,CALCRL,ADRB1,CCR2,CCRL2,FZD8,GPER1,SUCNR1
REAC:R-HSA-373076	Class A/1 (Rhodopsin-like receptors)	8.95445E-06	5.047960873	29	AGTR2,CX3CR1,PROK2,ADRA1A,PF4,FFAR2,RXFP1,CHRM1,CHRM2,CXCL11,NPFFR1,SSTR1,CXCL10,P2RY1,NPY1R,CXCR1,PLPPR1,CXCR2,CHRM3,EDNRB,GPR18,P2RY13,FFAR4,ACKR4,ADRB1,CCR2,CCRL2,GPER1,SUCNR1
REAC:R-HSA-6798695	Neutrophil degranulation	2.14395E-05	4.668784673	35	OLFM4,DEFA4,ORM2,PRTN3,RNASE3,S100A12,CD177,MS4A3,ORM1,MPO,SLCO4C1,S100A8,PGLYRP1,BPI,FOLR3,MME,RNASE2,FCGR3B,S100A9,CXCR1,CXCR2,CD36,MGAM,ATP8A1,MMP25,NDUFC2,HSPA6,ADGRE3,GCA,CLEC4D,MNDA,SIGLEC14,SCAMP1,ACP3,CR1

REAC:R-HSA-168256	Immune System	0.004963747	2.304190341	83	OLFM4,DEFA4,ORM2,PRTN3,DEFA3,RNASE3,PI3,S100A12,TUBB1,CSF2,CD177,MS4A3,FGG,AGER,ORM1,MPO,SLCO4C1,SH3GL2,S100A8,SFTPA2,PGLYRP1,IL1A,LILRA1,SFTPA1,BPI,BTNL8,FOLR3,TRIM71,MME,RNF182,CXCL10,CPB2,RNASE2,SIGLEC11,FCGR3B,S100A9,IL1B,CXCR1,CXCR2,FCN3,GBP4,CD36,MGAM,BTNL9,TRIB3,ATP8A1,SH2D1B,LTB,LILRA2,FGF2,CSF3R,MMP25,NDUFC2,PIK3AP1,KBTBD7,LILRA5,HSPA6,KLRF1,ADGRE3,UBE2D1,GCA,TLR3,CLEC4D,LIFR,MNDA,TLR8,GATA3,CCR2,IFIT3,AP1S2,CD274,TLR7,RALA,SIGLEC14,SCAMP1,ACP3,UBE2W,CR1,HECW2,PPP3R1,MEFV,CXADR,RNF144B
REAC:R-HSA-556833	Metabolism of lipids	0.008947205	2.048312629	37	SLCO1A2,CYP1A1,ANKRD1,HMGCS2,PLA2G4E,SCD,LRP2,MSMO1,ACADL,CYP1B1,GPAM,ELOVL3,CD36,HMGCS1,PLA2G3,TRIB3,GPD1,FASN,DGAT2,PNPLA3,ACOXL,SGPP1,PI4K2B,APOA1,INSIG1,STARD4,CHPT1,MFSD2A,HSD17B12,CAV1,DHCR7,SC5D,B3GALNT1,ACAT2,EBP,IDI1,FADS1
REAC:R-HSA-162582	Signal Transduction	0.034220611	1.465712234	92	HHIP,AGTR2,CX3CR1,TUBB1,PROK2,CSF2,ADRA1A,ADCY8,FGG,PF4,FFAR2,SH3GL2,ADGRE1,RXFP1,ITGA2B,S100A8,CHRM1,VEGFD,CHRM2,CXCL11,GPR15,WNT7A,NPFFR1,SSTR1,PRKG2,SCD,BDNF,CXCL10,PTCRA,P2RY1,S100A9,NPY1R,CXCR1,PLPPR1,CXCR2,WNT3A,PARD6B,FZD5,CHRM3,BMI1,GPAM,EDNRB,TRIB3,GPR18,ACVR1C,P2RY13,FFAR4,FGF2,ACKR4,FASN,PIK3AP1,KBTBD7,RXRG,SHH,FGF9,SCAI,VIP,SH3GL3,SDR16C5,ADGRE3,UBE2D1,ADH1A,SMAD6,KHDRBS2,BMPR2,CALCRL,RASGRF1,LRRK2,CAV2,ADRB1,TNFSF10,HPN,GTF2A1,GATA3,RSPO2,CCR2,CD274,RALA,CCRL2,ARHGAP29,FZD8,CAV1,ID4,ARRB1,TRPC3,ANOS1,PPP3R1,DLL4,EPAS1,DKK2,GPER1,SUCNR1
**Down regulated genes**					
REAC:R-HSA-5083625	Defective GALNT3 causes HFTC	0.014807171	1.829527921	4	MUC5AC,MUC13,MUC16,MUC4
REAC:R-HSA-9037629	Lewis blood group biosynthesis	0.014807171	1.829527921	4	B3GALT5,FUT6,B4GALNT2,FUT2
REAC:R-HSA-2672351	Stimuli-sensing channels	0.036572161	1.436849376	7	BEST4,STOML3,SLC9C2,WNK2,WNK4,TRPV6,CLCNKA
REAC:R-HSA-211859	Biological oxidations	0.0454341	1.342618075	10	CYP2A6,CYP2F1,GSTA2,CYP2A13,MAT1A,ADH6,GSTA1,GSTA3,CYP2J2,ALDH3A1
REAC:R-HSA-983712	Ion channel transport	0.13379853	0.873548658	8	ATP12A,BEST4,STOML3,SLC9C2,WNK2,WNK4,TRPV6,CLCNKA

REAC:R-HSA-9006931	Signaling by Nuclear Receptors	0.219572305	0.65842244	10	PCK1,CRABP1,EPGN,FABP6, MMP3,TFF3,H2BC26,CRABP2, H2BC8,H2AC8

Table S4 Topology table for up and down regulated genes

Regulation	Node	Degree	Betweenness	Stress	Closeness
Up	LRRK2	472	0.190674	1.06E+08	0.335042
Up	BMI1	218	0.071143	53875450	0.298069
Up	EBP	206	0.071703	29140262	0.287384
Up	MNDA	171	0.047777	41765340	0.276267
Up	KBTBD7	126	0.040553	11107264	0.280663
Up	SH3GL3	109	0.03146	7853178	0.292344
Up	FFAR2	108	0.022616	14923842	0.250609
Up	CAV1	104	0.035343	19955290	0.285318
Up	ARRB1	101	0.029933	15649370	0.279899
Up	MAPT	92	0.025833	13875412	0.273937
Up	MME	91	0.028261	18076366	0.26538
Up	NEK7	91	0.029626	6679066	0.279028
Up	TTN	84	0.023143	10691940	0.282317
Up	TRIB3	83	0.027033	8927188	0.29651
Up	TLCD4	80	0.016566	6890526	0.263755
Up	PARD6B	71	0.026644	5780562	0.289526
Up	UBE2W	68	0.022196	6224972	0.277814
Up	HSPA6	68	0.016356	11408814	0.264786
Up	TMEM97	67	0.019244	6783312	0.265102
Up	CHRM3	63	0.018624	7072284	0.27258
Up	LAMP3	63	0.020745	6535078	0.271461
Up	KHDRBS2	63	0.014929	8785332	0.254419
Up	CAV2	62	0.0132	13037948	0.257127
Up	ASGR2	61	0.012411	9569980	0.238486
Up	IFIT3	60	0.013739	5742008	0.274574
Up	VGLL3	59	0.006261	5614482	0.251244
Up	FAM167A	57	0.014141	8093696	0.268952
Up	SLC33A1	56	0.015855	7425746	0.271236
Up	BDNF	56	0.016474	6042434	0.270299
Up	AATK	52	0.012964	9398450	0.24777
Up	TUBB1	51	0.013668	5348290	0.261348
Up	NEBL	51	0.01065	8422544	0.276693
Up	APOA1	51	0.013963	7094756	0.257759
Up	MAP3K21	51	0.0124	8804010	0.234549
Up	PLLP	49	0.018813	6150918	0.268549
Up	FASN	49	0.011236	8103754	0.25936
Up	OLFM4	49	0.011917	7462782	0.242366
Up	CYP1A1	48	0.0126	6308186	0.249562
Up	HPCAL1	48	0.013521	7156920	0.26466
Up	EPB41L5	48	0.012672	7586464	0.263855
Up	KLRG2	47	0.012514	3790316	0.245363
Up	UBE2D1	47	0.009859	4929052	0.25566
Up	MAL2	47	0.009516	7300358	0.248591
Up	IER3IP1	45	0.010479	3871278	0.248791
Up	SCD	44	0.008916	4647562	0.252236
Up	VNN2	44	0.011702	5134620	0.248468
Up	SLC25A46	44	0.008476	5268336	0.245689
Up	CCR2	41	0.010649	4340172	0.261127
Up	TMEM139	41	0.006307	4308482	0.234124
Up	SCAMP1	40	0.008833	5807804	0.261311
Up	S100A8	38	0.006992	5069398	0.256545
Up	LRRC25	38	0.006222	3309428	0.226148
Up	SLC1A1	37	0.010948	4480156	0.253075
Up	MS4A3	37	0.003674	3888962	0.216608
Up	ALPP	36	0.008295	2222676	0.248368
Up	EFNB2	35	0.010959	2197494	0.265723
Up	MZT1	35	0.007766	3610258	0.235234

Up	BTNL8	35	0.009639	1996720	0.225817
Up	S100A9	34	0.005288	4106434	0.249003
Up	OCLN	34	0.00856	4035020	0.255872
Up	HSD17B12	33	0.006675	5796534	0.247439
Up	WNT7A	32	0.008898	4194240	0.260381
Up	SRP9	32	0.010153	1734654	0.281359
Up	TMEM100	32	0.008166	2540638	0.253931
Up	CHPT1	31	0.00693	1799108	0.243332
Up	SCOC	31	0.007589	2988652	0.251984
Up	PRAM1	29	0.008449	3539586	0.275145
Up	ANP32E	29	0.006114	3115816	0.249843
Up	CD274	29	0.006097	2975198	0.220853
Up	PHGDH	28	0.008161	2353820	0.275992
Up	ADRA1A	28	0.004997	1951692	0.242758
Up	AP1S2	28	0.007166	3086614	0.231009
Up	ZMYM1	28	0.007951	2099344	0.262757
Up	CDH13	27	0.005985	2659482	0.239948
Up	NECAB1	27	0.004831	2711672	0.232966
Up	SLC39A8	27	0.005109	2055994	0.234252
Up	GATA3	26	0.003939	2670070	0.242197
Up	LRP2	26	0.007175	1907922	0.244704
Up	PPP3R1	25	0.007379	2004758	0.238896
Up	GPM6A	25	0.006132	2357450	0.252902
Up	DCC	25	0.005345	4765410	0.221513
Up	SH2D1B	24	0.004062	2069942	0.258154
Up	RALA	24	0.006369	2662942	0.240114
Up	TNNT1	24	0.003339	2686226	0.229663
Up	CXCR2	24	0.004426	2347046	0.243684
Up	GCA	24	0.005351	3004102	0.238363
Up	SPRYD7	24	0.005051	2103140	0.24608
Up	QKI	24	0.004136	1912622	0.25378
Up	CFL2	24	0.003525	2115738	0.252706
Up	RBM27	24	0.00478	1955316	0.243066
Up	PLPPR1	23	0.00463	2162458	0.250892
Up	EDNRB	23	0.005338	1258118	0.241691
Up	SGPP1	23	0.005228	1293756	0.251961
Up	BEX1	23	0.004242	3422062	0.245017
Up	TSPAN12	22	0.001487	925910	0.220757
Up	CBS	22	0.005215	1234782	0.265685
Up	ARHGAP29	22	0.004421	1684386	0.251107
Up	PI4K2B	22	0.004614	1768178	0.242472
Up	MAP1LC3C	22	0.004982	1972584	0.229862
Up	B3GALNT1	21	0.005336	1126626	0.24
Up	ACP3	21	0.002576	1647834	0.228341
Up	HLX	21	0.006307	1362802	0.261594
Up	ANKRD1	21	0.00359	2198346	0.233347
Up	PEG10	21	0.004032	2364836	0.224539
Up	CD244	21	0.004061	2017350	0.222391
Up	DACH1	21	0.003655	804372	0.236859
Up	MSMO1	20	0.002071	1768042	0.209534
Up	FAM241A	20	0.003537	1219752	0.213249
Up	SEC14L4	20	0.002623	1149826	0.247153
Up	CRTAC1	20	0.003904	1466564	0.23377
Up	FADS1	19	0.002943	1095324	0.233151
Up	TPD52	19	0.003137	1804230	0.233544
Up	MTARC1	19	0.004099	1386362	0.236214
Up	FREM2	18	0.004422	1725406	0.232391
Up	KANK4	18	0.00341	1391402	0.217575
Up	EPAS1	18	0.00301	1633572	0.222284
Up	BRINP1	17	0.002968	1345528	0.223843
Up	BATF2	17	0.003047	556486	0.248602
Up	ABCA3	17	0.003037	1329398	0.214625
Up	SPRING1	17	0.003723	1590956	0.245222
Up	GPR18	2	1.51E-04	72740	0.202397
Up	INSIG1	2	1.05E-04	48156	0.212458
Up	BMPR2	2	0.001074	344232	0.234519
Up	ASPG	2	6.00E-06	8734	0.215977
Up	PIK3AP1	1	0	0	0.206597
Up	PNPLA3	1	0	0	0.199318
Up	DHCR7	1	0	0	0.189076

Up	RAPGEF6	1	0	0	0.195848
Up	AGER	1	0	0	0.212792
Up	GRIA1	1	0	0	0.195944
Up	ZNF385B	1	0	0	0.229634
Up	SH3GL2	1	0	0	0.250971
Up	EFR3B	1	0	0	0.197329
Up	CLTRN	1	0	0	0.195091
Up	CACNA2D2	1	0	0	0.184224
Up	PDP2	1	0	0	0.197259
Up	C2orf88	1	0	0	0.199111
Up	ORM1	1	0	0	0.198919
Up	PRODH	1	0	0	0.20936
Up	P2RY1	1	0	0	0.20186
Up	FCGR3B	1	0	0	0.205946
Up	HSD17B6	1	0	0	0.205946
Up	VIP	1	0	0	0.196603
Up	ANXA3	1	0	0	0.221991
Up	CACNB4	1	0	0	0.218362
Up	CXCL11	1	0	0	0.250971
Up	CA2	1	0	0	0.250971
Up	IDI1	1	0	0	0.250971
Up	FMO5	1	0	0	0.195841
Up	SP6	1	0	0	0.218165
Up	ANGPTL7	1	0	0	0.219163
Up	SGCG	1	0	0	0.22017
Up	AARD	1	0	0	0.210208
Down	KRT15	139	0.04143	17418098	0.301727
Down	OTX1	135	0.030588	21151220	0.266449
Down	TEKT4	112	0.032469	9352184	0.294482
Down	SPAG8	103	0.036366	18673084	0.30889
Down	EFHC2	102	0.023749	13739234	0.27935
Down	H2AC8	83	0.024611	6197630	0.281616
Down	PITX1	82	0.016303	6238340	0.280748
Down	ZMYND12	81	0.016851	5737646	0.275254
Down	TSGA10	79	0.014862	7614038	0.266143
Down	CCDC146	75	0.015646	10846316	0.271966
Down	IQUB	75	0.019032	14182898	0.288939
Down	PPP1R32	74	0.01733	8946190	0.293425
Down	PIH1D2	74	0.01504	6641412	0.276652
Down	TRIM29	73	0.021067	12200560	0.281131
Down	GPR156	69	0.0195	7611260	0.268704
Down	MORN3	68	0.014673	5553112	0.286969
Down	KRT13	68	0.009517	8500452	0.270945
Down	DEUP1	60	0.015169	5303104	0.284296
Down	PKP2	58	0.018858	8281268	0.293595
Down	ATP12A	55	0.016818	4886682	0.259347
Down	GJB7	51	0.013598	6713456	0.24583
Down	SERPINB2	46	0.01036	6281408	0.243535
Down	KRT14	45	0.006234	5565110	0.256415
Down	RSPH14	44	0.010927	4212324	0.278776
Down	FNDC11	44	0.008116	6241718	0.264798
Down	RIBC1	41	0.008105	3703944	0.268108
Down	GAS2L2	40	0.007397	4935930	0.260466
Down	ZG16B	39	0.010951	3230652	0.25027
Down	CCDC33	39	0.005982	3754990	0.260503
Down	TEKT3	38	0.005802	1893192	0.267695
Down	ODAD3	36	0.005999	4403200	0.255613
Down	MNS1	35	0.006885	2758774	0.274316
Down	LDLRAD1	34	0.006013	1330408	0.246167
Down	BAAT	34	0.006329	5346130	0.251653
Down	INSYN2A	33	0.007012	3379538	0.255307
Down	EPN3	32	0.006518	2340840	0.257771
Down	FAM83A	32	0.004534	3671638	0.26236
Down	DYDC1	32	0.003364	3009704	0.250451
Down	CCDC74B	32	0.005504	5667388	0.238999
Down	SLC27A2	31	0.007914	1713272	0.248925
Down	SERPINB3	31	0.00563	3936342	0.241723
Down	BDKRB1	31	0.007723	2242072	0.244607
Down	TTC23L	30	0.004402	3676066	0.244252
Down	ZNF474	29	0.004283	1420618	0.260466

Down	CDC20B	29	0.004849	4072152	0.260968
Down	SRGAP3	28	0.004753	3464156	0.262745
Down	MAP7D2	28	0.007073	2374110	0.256581
Down	EFNB3	28	0.006063	2698100	0.235025
Down	CCDC40	28	0.006637	3503538	0.226711
Down	LRRIQ1	28	0.005729	2532526	0.222365
Down	CFAP53	28	0.002528	2485698	0.241744
Down	CNGA3	27	0.007723	1571534	0.249249
Down	TMEM231	27	0.008263	3972348	0.240634
Down	TMEM184A	26	0.00679	1754794	0.245721
Down	TEKT1	26	0.003174	1610572	0.257103
Down	ZBBX	25	0.005702	1144532	0.233779
Down	ZCCHC12	25	0.005028	2186068	0.257103
Down	PRMT8	24	0.00457	1815782	0.24027
Down	LCA5L	24	0.002846	2152058	0.257115
Down	CLCNKA	24	0.003098	1512116	0.248925
Down	NGEF	24	0.004375	3394348	0.252729
Down	ERICH5	23	0.005562	1589752	0.243524
Down	AK8	23	0.003846	3112480	0.235803
Down	ARHGAP39	23	0.005476	2447044	0.242102
Down	TP73	22	0.004929	2030990	0.231838
Down	RIBC2	22	0.00551	1336290	0.262434
Down	CFAP100	22	0.002885	2339676	0.255648
Down	C20orf85	22	0.002468	2446246	0.232401
Down	SLC23A1	22	0.001624	1695110	0.244952
Down	FAM166B	21	0.001975	784560	0.244402
Down	FAM83B	21	0.003002	2319916	0.242176
Down	FOXJ1	21	0.004882	2455478	0.228849
Down	FLACC1	21	0.00364	1949842	0.238506
Down	CCDC180	21	0.001999	1615478	0.225533
Down	CLDN2	20	0.003773	2826512	0.233318
Down	CCDC153	20	0.002082	1105816	0.23825
Down	LRRC46	20	0.004517	1705482	0.220932
Down	DCDC2B	19	0.002655	1759754	0.251244
Down	SPDEF	19	0.004447	1360850	0.241052
Down	NHLRC4	19	0.002878	652636	0.259942
Down	HHATL	19	0.002887	876688	0.24028
Down	KIF24	19	0.002799	1332950	0.239153
Down	C1orf87	18	0.004359	890600	0.27001
Down	TTLL10	17	0.001559	1616018	0.24407
Down	CCDC17	17	0.001069	1242652	0.249933
Down	ODAD4	17	0.002318	1588464	0.247483
Down	SPTBN2	16	0.002395	1220708	0.232148
Down	SCGB2A1	16	0.003224	1253174	0.229076
Down	ZMYND10	16	0.003082	1689944	0.230501
Down	ALDH3A1	16	0.003372	1338176	0.215725
Down	ELF3	16	0.001865	988574	0.247307
Down	SPATA18	16	0.001743	574752	0.253733
Down	CATIP	16	0.001846	1647140	0.230645
Down	KLHDC8A	16	0.001582	1394580	0.226111
Down	BBOX1	15	0.003124	1107474	0.218568
Down	SERPINE1	15	0.002846	903206	0.248302
Down	OXTR	15	0.002718	884636	0.228567
Down	ECT2L	15	0.003524	658760	0.249271
Down	ENKUR	15	0.005566	1110698	0.286126
Down	LRRC56	15	0.001547	1302402	0.246331
Down	DNAAF6	15	0.003293	645306	0.241754
Down	HHLA2	15	0.001132	688642	0.211407
Down	RAB3B	14	0.002604	635154	0.234519
Down	SYT12	14	0.003878	846804	0.236808
Down	AKAP14	14	0.002154	875626	0.220625
Down	FANK1	14	0.002044	1609636	0.245092
Down	TMPRSS4	14	0.001922	1095720	0.221673
Down	GJB3	13	9.78E-04	383678	0.226692
Down	C21orf58	13	9.83E-04	559838	0.259347
Down	PLEKHS1	13	0.001282	1120706	0.218577
Down	DQX1	13	0.002291	682462	0.211665
Down	SPACA9	13	0.001535	1157462	0.225524
Down	CHST6	13	0.003695	1352488	0.219709
Down	MAK	12	0.002266	873078	0.220022

Down	CRABP2	12	0.001367	775692	0.218148
Down	DNAJB13	12	0.002065	958372	0.224403
Down	CLDN10	12	0.00183	734174	0.218706
Down	TSNAXIP1	12	0.002017	1063014	0.241094
Down	MORN5	12	0.001763	485166	0.23442
Down	CAPN13	12	0.001982	1168464	0.225368
Down	FUT2	2	2.74E-04	97780	0.233044
Down	DRC7	2	4.46E-04	125252	0.235743
Down	ROPN1L	2	1.75E-04	70850	0.223599
Down	ODAD1	1	0	0	0.203584
Down	ADGB	1	0	0	0.219744
Down	DNAH5	1	0	0	0.219744
Down	FAM83F	1	0	0	0.209732
Down	C22orf15	1	0	0	0.20936
Down	LCNL1	1	0	0	0.18605
Down	COMP	1	0	0	0.210399
Down	ATG9B	1	0	0	0.210399
Down	DNAH3	1	0	0	0.218697
Down	CDHR3	1	0	0	0.219587
Down	IGFL2	1	0	0	0.219215
Down	PACRG	1	0	0	0.184818
Down	ARMC3	1	0	0	0.198365
Down	UBXN10	1	0	0	0.250971
Down	OSCP1	1	0	0	0.178701
Down	RSPH1	1	0	0	0.222989
Down	ZDHHC11	1	0	0	0.175773
Down	CFAP45	1	0	0	0.22248
Down	SPATA17	1	0	0	0.218165
Down	TRIM17	1	0	0	0.217422
Down	SEC14L3	1	0	0	0.198181
Down	SERPINI2	1	0	0	0.209281
Down	SCG5	1	0	0	0.207066
Down	ADH6	1	0	0	0.218697
Down	PLPPR3	1	0	0	0.200578
Down	NME5	1	0	0	0.200296
Down	KLHL32	1	0	0	0.236004
Down	TTC29	1	0	0	0.215851
Down	CD164L2	1	0	0	0.211706
Category	Node	Degree	Betweenness	Stress	Closeness
Up	LRRK2	472	0.190674	1.06E+08	0.335042
Up	BMI1	218	0.071143	53875450	0.298069
Up	EBP	206	0.071703	29140262	0.287384
Up	MNDA	171	0.047777	41765340	0.276267
Up	KBTBD7	126	0.040553	11107264	0.280663
Up	SH3GL3	109	0.03146	7853178	0.292344
Up	FFAR2	108	0.022616	14923842	0.250609
Up	CAV1	104	0.035343	19955290	0.285318
Up	ARRB1	101	0.029933	15649370	0.279899
Up	MAPT	92	0.025833	13875412	0.273937
Up	MME	91	0.028261	18076366	0.26538
Up	NEK7	91	0.029626	6679066	0.279028
Up	TTN	84	0.023143	10691940	0.282317
Up	TRIB3	83	0.027033	8927188	0.29651
Up	TLCD4	80	0.016566	6890526	0.263755
Up	PARD6B	71	0.026644	5780562	0.289526
Up	UBE2W	68	0.022196	6224972	0.277814
Up	HSPA6	68	0.016356	11408814	0.264786
Up	TMEM97	67	0.019244	6783312	0.265102
Up	CHRM3	63	0.018624	7072284	0.27258
Up	LAMP3	63	0.020745	6535078	0.271461
Up	KHDRBS2	63	0.014929	8785332	0.254419
Up	CAV2	62	0.0132	13037948	0.257127
Up	ASGR2	61	0.012411	9569980	0.238486
Up	IFIT3	60	0.013739	5742008	0.274574
Up	VGLL3	59	0.006261	5614482	0.251244
Up	FAM167A	57	0.014141	8093696	0.268952
Up	SLC33A1	56	0.015855	7425746	0.271236
Up	BDNF	56	0.016474	6042434	0.270299
Up	AATK	52	0.012964	9398450	0.24777
Up	TUBB1	51	0.013668	5348290	0.261348

Up	NEBL	51	0.01065	8422544	0.276693
Up	APOA1	51	0.013963	7094756	0.257759
Up	MAP3K21	51	0.0124	8804010	0.234549
Up	PLLP	49	0.018813	6150918	0.268549
Up	FASN	49	0.011236	8103754	0.25936
Up	OLFM4	49	0.011917	7462782	0.242366
Up	CYP1A1	48	0.0126	6308186	0.249562
Up	HPCAL1	48	0.013521	7156920	0.26466
Up	EPB41L5	48	0.012672	7586464	0.263855
Up	KLRG2	47	0.012514	3790316	0.245363
Up	UBE2D1	47	0.009859	4929052	0.25566
Up	MAL2	47	0.009516	7300358	0.248591
Up	IER3IP1	45	0.010479	3871278	0.248791
Up	SCD	44	0.008916	4647562	0.252236
Up	VNN2	44	0.011702	5134620	0.248468
Up	SLC25A46	44	0.008476	5268336	0.245689
Up	CCR2	41	0.010649	4340172	0.261127
Up	TMEM139	41	0.006307	4308482	0.234124
Up	SCAMP1	40	0.008833	5807804	0.261311
Up	S100A8	38	0.006992	5069398	0.256545
Up	LRRC25	38	0.006222	3309428	0.226148
Up	SLC1A1	37	0.010948	4480156	0.253075
Up	MS4A3	37	0.003674	3888962	0.216608
Up	ALPP	36	0.008295	2222676	0.248368
Up	EFNB2	35	0.010959	2197494	0.265723
Up	MZT1	35	0.007766	3610258	0.235234
Up	BTNL8	35	0.009639	1996720	0.225817
Up	S100A9	34	0.005288	4106434	0.249003
Up	OCLN	34	0.00856	4035020	0.255872
Up	HSD17B12	33	0.006675	5796534	0.247439
Up	WNT7A	32	0.008898	4194240	0.260381
Up	SRP9	32	0.010153	1734654	0.281359
Up	TMEM100	32	0.008166	2540638	0.253931
Up	CHPT1	31	0.00693	1799108	0.243332
Up	SCOC	31	0.007589	2988652	0.251984
Up	PRAM1	29	0.008449	3539586	0.275145
Up	ANP32E	29	0.006114	3115816	0.249843
Up	CD274	29	0.006097	2975198	0.220853
Up	PHGDH	28	0.008161	2353820	0.275992
Up	ADRA1A	28	0.004997	1951692	0.242758
Up	AP1S2	28	0.007166	3086614	0.231009
Up	ZMYM1	28	0.007951	2099344	0.262757
Up	CDH13	27	0.005985	2659482	0.239948
Up	NECAB1	27	0.004831	2711672	0.232966
Up	SLC39A8	27	0.005109	2055994	0.234252
Up	GATA3	26	0.003939	2670070	0.242197
Up	LRP2	26	0.007175	1907922	0.244704
Up	PPP3R1	25	0.007379	2004758	0.238896
Up	GPM6A	25	0.006132	2357450	0.252902
Up	DCC	25	0.005345	4765410	0.221513
Up	SH2D1B	24	0.004062	2069942	0.258154
Up	RALA	24	0.006369	2662942	0.240114
Up	TNNT1	24	0.003339	2686226	0.229663
Up	CXCR2	24	0.004426	2347046	0.243684
Up	GCA	24	0.005351	3004102	0.238363
Up	SPRYD7	24	0.005051	2103140	0.24608
Up	QKI	24	0.004136	1912622	0.25378
Up	CFL2	24	0.003525	2115738	0.252706
Up	RBM27	24	0.00478	1955316	0.243066
Up	PLPPR1	23	0.00463	2162458	0.250892
Up	EDNRB	23	0.005338	1258118	0.241691
Up	SGPP1	23	0.005228	1293756	0.251961
Up	BEX1	23	0.004242	3422062	0.245017
Up	TSPAN12	22	0.001487	925910	0.220757
Up	CBS	22	0.005215	1234782	0.265685
Up	ARHGAP29	22	0.004421	1684386	0.251107
Up	PI4K2B	22	0.004614	1768178	0.242472
Up	MAP1LC3C	22	0.004982	1972584	0.229862
Up	B3GALNT1	21	0.005336	1126626	0.24
Up	ACP3	21	0.002576	1647834	0.228341

Up	HLX	21	0.006307	1362802	0.261594
Up	ANKRD1	21	0.00359	2198346	0.233347
Up	PEG10	21	0.004032	2364836	0.224539
Up	CD244	21	0.004061	2017350	0.222391
Up	DACH1	21	0.003655	804372	0.236859
Up	MSMO1	20	0.002071	1768042	0.209534
Up	FAM241A	20	0.003537	1219752	0.213249
Up	SEC14L4	20	0.002623	1149826	0.247153
Up	CRTAC1	20	0.003904	1466564	0.23377
Up	FADS1	19	0.002943	1095324	0.233151
Up	TPD52	19	0.003137	1804230	0.233544
Up	MTARC1	19	0.004099	1386362	0.236214
Up	FREM2	18	0.004422	1725406	0.232391
Up	KANK4	18	0.00341	1391402	0.217575
Up	EPAS1	18	0.00301	1633572	0.222284
Up	BRINP1	17	0.002968	1345528	0.223843
Up	BATF2	17	0.003047	556486	0.248602
Up	ABCA3	17	0.003037	1329398	0.214625
Up	SPRING1	17	0.003723	1590956	0.245222
Up	GPR18	2	1.51E-04	72740	0.202397
Up	INSIG1	2	1.05E-04	48156	0.212458
Up	BMPR2	2	0.001074	344232	0.234519
Up	ASPG	2	6.00E-06	8734	0.215977
Up	PIK3AP1	1	0	0	0.206597
Up	PNPLA3	1	0	0	0.199318
Up	DHCR7	1	0	0	0.189076
Up	RAPGEF6	1	0	0	0.195848
Up	AGER	1	0	0	0.212792
Up	GRIA1	1	0	0	0.195944
Up	ZNF385B	1	0	0	0.229634
Up	SH3GL2	1	0	0	0.250971
Up	EFR3B	1	0	0	0.197329
Up	CLTRN	1	0	0	0.195091
Up	CACNA2D2	1	0	0	0.184224
Up	PDP2	1	0	0	0.197259
Up	C2orf88	1	0	0	0.199111
Up	ORM1	1	0	0	0.198919
Up	PRODH	1	0	0	0.20936
Up	P2RY1	1	0	0	0.20186
Up	FCGR3B	1	0	0	0.205946
Up	HSD17B6	1	0	0	0.205946
Up	VIP	1	0	0	0.196603
Up	ANXA3	1	0	0	0.221991
Up	CACNB4	1	0	0	0.218362
Up	CXCL11	1	0	0	0.250971
Up	CA2	1	0	0	0.250971
Up	IDI1	1	0	0	0.250971
Up	FMO5	1	0	0	0.195841
Up	SP6	1	0	0	0.218165
Up	ANGPTL7	1	0	0	0.219163
Up	SGCG	1	0	0	0.22017
Up	AARD	1	0	0	0.210208
Down	KRT15	139	0.04143	17418098	0.301727
Down	OTX1	135	0.030588	21151220	0.266449
Down	TEKT4	112	0.032469	9352184	0.294482
Down	SPAG8	103	0.036366	18673084	0.30889
Down	EFHC2	102	0.023749	13739234	0.27935
Down	H2AC8	83	0.024611	6197630	0.281616
Down	PITX1	82	0.016303	6238340	0.280748
Down	ZMYND12	81	0.016851	5737646	0.275254
Down	TSGA10	79	0.014862	7614038	0.266143
Down	CCDC146	75	0.015646	10846316	0.271966
Down	IQUB	75	0.019032	14182898	0.288939
Down	PPP1R32	74	0.01733	8946190	0.293425
Down	PIH1D2	74	0.01504	6641412	0.276652
Down	TRIM29	73	0.021067	12200560	0.281131
Down	GPR156	69	0.0195	7611260	0.268704
Down	MORN3	68	0.014673	5553112	0.286969
Down	KRT13	68	0.009517	8500452	0.270945
Down	DEUP1	60	0.015169	5303104	0.284296

Down	PKP2	58	0.018858	8281268	0.293595
Down	ATP12A	55	0.016818	4886682	0.259347
Down	GJB7	51	0.013598	6713456	0.24583
Down	SERPINB2	46	0.01036	6281408	0.243535
Down	KRT14	45	0.006234	5565110	0.256415
Down	RSPH14	44	0.010927	4212324	0.278776
Down	FNDC11	44	0.008116	6241718	0.264798
Down	RIBC1	41	0.008105	3703944	0.268108
Down	GAS2L2	40	0.007397	4935930	0.260466
Down	ZG16B	39	0.010951	3230652	0.25027
Down	CCDC33	39	0.005982	3754990	0.260503
Down	TEKT3	38	0.005802	1893192	0.267695
Down	ODAD3	36	0.005999	4403200	0.255613
Down	MNS1	35	0.006885	2758774	0.274316
Down	LDLRAD1	34	0.006013	1330408	0.246167
Down	BAAT	34	0.006329	5346130	0.251653
Down	INSYN2A	33	0.007012	3379538	0.255307
Down	EPN3	32	0.006518	2340840	0.257771
Down	FAM83A	32	0.004534	3671638	0.26236
Down	DYDC1	32	0.003364	3009704	0.250451
Down	CCDC74B	32	0.005504	5667388	0.238999
Down	SLC27A2	31	0.007914	1713272	0.248925
Down	SERPINB3	31	0.00563	3936342	0.241723
Down	BDKRB1	31	0.007723	2242072	0.244607
Down	TTC23L	30	0.004402	3676066	0.244252
Down	ZNF474	29	0.004283	1420618	0.260466
Down	CDC20B	29	0.004849	4072152	0.260968
Down	SRGAP3	28	0.004753	3464156	0.262745
Down	MAP7D2	28	0.007073	2374110	0.256581
Down	EFNB3	28	0.006063	2698100	0.235025
Down	CCDC40	28	0.006637	3503538	0.226711
Down	LRRIQ1	28	0.005729	2532526	0.222365
Down	CFAP53	28	0.002528	2485698	0.241744
Down	CNGA3	27	0.007723	1571534	0.249249
Down	TMEM231	27	0.008263	3972348	0.240634
Down	TMEM184A	26	0.00679	1754794	0.245721
Down	TEKT1	26	0.003174	1610572	0.257103
Down	ZBBX	25	0.005702	1144532	0.233779
Down	ZCCHC12	25	0.005028	2186068	0.257103
Down	PRMT8	24	0.00457	1815782	0.24027
Down	LCA5L	24	0.002846	2152058	0.257115
Down	CLCNKA	24	0.003098	1512116	0.248925
Down	NGEF	24	0.004375	3394348	0.252729
Down	ERICH5	23	0.005562	1589752	0.243524
Down	AK8	23	0.003846	3112480	0.235803
Down	ARHGAP39	23	0.005476	2447044	0.242102
Down	TP73	22	0.004929	2030990	0.231838
Down	RIBC2	22	0.00551	1336290	0.262434
Down	CFAP100	22	0.002885	2339676	0.255648
Down	C20orf85	22	0.002468	2446246	0.232401
Down	SLC23A1	22	0.001624	1695110	0.244952
Down	FAM166B	21	0.001975	784560	0.244402
Down	FAM83B	21	0.003002	2319916	0.242176
Down	FOXJ1	21	0.004882	2455478	0.228849
Down	FLACC1	21	0.00364	1949842	0.238506
Down	CCDC180	21	0.001999	1615478	0.225533
Down	CLDN2	20	0.003773	2826512	0.233318
Down	CCDC153	20	0.002082	1105816	0.23825
Down	LRRC46	20	0.004517	1705482	0.220932
Down	DCDC2B	19	0.002655	1759754	0.251244
Down	SPDEF	19	0.004447	1360850	0.241052
Down	NHLRC4	19	0.002878	652636	0.259942
Down	HHATL	19	0.002887	876688	0.24028
Down	KIF24	19	0.002799	1332950	0.239153
Down	C1orf87	18	0.004359	890600	0.27001
Down	TTLL10	17	0.001559	1616018	0.24407
Down	CCDC17	17	0.001069	1242652	0.249933
Down	ODAD4	17	0.002318	1588464	0.247483
Down	SPTBN2	16	0.002395	1220708	0.232148
Down	SCGB2A1	16	0.003224	1253174	0.229076

Down	ZMYND10	16	0.003082	1689944	0.230501
Down	ALDH3A1	16	0.003372	1338176	0.215725
Down	ELF3	16	0.001865	988574	0.247307
Down	SPATA18	16	0.001743	574752	0.253733
Down	CATIP	16	0.001846	1647140	0.230645
Down	KLHDC8A	16	0.001582	1394580	0.226111
Down	BBOX1	15	0.003124	1107474	0.218568
Down	SERPINE1	15	0.002846	903206	0.248302
Down	OXTR	15	0.002718	884636	0.228567
Down	ECT2L	15	0.003524	658760	0.249271
Down	ENKUR	15	0.005566	1110698	0.286126
Down	LRRC56	15	0.001547	1302402	0.246331
Down	DNAAF6	15	0.003293	645306	0.241754
Down	HHLA2	15	0.001132	688642	0.211407
Down	RAB3B	14	0.002604	635154	0.234519
Down	SYT12	14	0.003878	846804	0.236808
Down	AKAP14	14	0.002154	875626	0.220625
Down	FANK1	14	0.002044	1609636	0.245092
Down	TMPRSS4	14	0.001922	1095720	0.221673
Down	GJB3	13	9.78E-04	383678	0.226692
Down	C21orf58	13	9.83E-04	559838	0.259347
Down	PLEKHS1	13	0.001282	1120706	0.218577
Down	DQX1	13	0.002291	682462	0.211665
Down	SPACA9	13	0.001535	1157462	0.225524
Down	CHST6	13	0.003695	1352488	0.219709
Down	MAK	12	0.002266	873078	0.220022
Down	CRABP2	12	0.001367	775692	0.218148
Down	DNAJB13	12	0.002065	958372	0.224403
Down	CLDN10	12	0.00183	734174	0.218706
Down	TSNAXIP1	12	0.002017	1063014	0.241094
Down	MORN5	12	0.001763	485166	0.23442
Down	CAPN13	12	0.001982	1168464	0.225368
Down	FUT2	2	2.74E-04	97780	0.233044
Down	DRC7	2	4.46E-04	125252	0.235743
Down	ROPN1L	2	1.75E-04	70850	0.223599
Down	ODAD1	1	0	0	0.203584
Down	ADGB	1	0	0	0.219744
Down	DNAH5	1	0	0	0.219744
Down	FAM83F	1	0	0	0.209732
Down	C22orf15	1	0	0	0.20936
Down	LCNL1	1	0	0	0.18605
Down	COMP	1	0	0	0.210399
Down	ATG9B	1	0	0	0.210399
Down	DNAH3	1	0	0	0.218697
Down	CDHR3	1	0	0	0.219587
Down	IGFL2	1	0	0	0.219215
Down	PACRG	1	0	0	0.184818
Down	ARMC3	1	0	0	0.198365
Down	UBXN10	1	0	0	0.250971
Down	OSCP1	1	0	0	0.178701
Down	RSPH1	1	0	0	0.222989
Down	ZDHHC11	1	0	0	0.175773
Down	CFAP45	1	0	0	0.22248
Down	SPATA17	1	0	0	0.218165
Down	TRIM17	1	0	0	0.217422
Down	SEC14L3	1	0	0	0.198181
Down	SERPINI2	1	0	0	0.209281
Down	SCG5	1	0	0	0.207066
Down	ADH6	1	0	0	0.218697
Down	PLPPR3	1	0	0	0.200578
Down	NME5	1	0	0	0.200296
Down	KLHL32	1	0	0	0.236004
Down	TTC29	1	0	0	0.215851
Down	CD164L2	1	0	0	0.211706

Table S5 Candidate miRNA and TFs targeted by the hub genes.

Regulation	Hub Genes	Degree	MicroRNA	Regulation	Hub Genes	Degree	TF
Up	PARD6B	217	hsa-mir-6830-5p	Up	CAV1	16	NFYA
Up	NEK7	156	hsa-mir-362-3p	Up	TTN	11	SRF
Up	BMI1	115	hsa-mir-15b	Up	MAPT	11	TFAP2A
Up	CAV1	115	hsa-mir-766-5p	Up	TRIB3	10	EN1
Up	TRIB3	80	hsa-mir-8057	Up	NEK7	9	ELK4
Up	MME	54	hsa-mir-526a	Up	MME	9	BRCA1
Up	ARRB1	52	hsa-mir-6804-5p	Up	KBTBD7	9	ARID3A
Up	MAPT	45	hsa-mir-2276-3p	Up	PARD6B	9	GATA2
Up	KBTBD7	40	hsa-mir-4743-3p	Up	EBP	8	YY1
Up	TTN	31	hsa-mir-641	Up	LRRK2	8	CEBPB
Up	FFAR2	24	hsa-mir-6787-3p	Up	ARRB1	8	FOXA1
Up	EBP	17	hsa-mir-16-5p	Up	FFAR2	7	PRRX2
Up	LRRK2	14	hsa-mir-10b-5p	Up	MNDA	6	STAT3
Up	MNDA	7	hsa-mir-181d-5p	Up	BMI1	6	EGR1
Up	SH3GL3	7	hsa-mir-374a-5p	Up	SH3GL3	5	IRF2
Down	GPR156	42	hsa-mir-302a-3p	Down	TSGA10	12	JUN
Down	PITX1	39	hsa-mir-19b-3p	Down	IQUB	9	STAT1
Down	TRIM29	27	hsa-mir-4524a-3p	Down	MORN3	9	MEF2A
Down	OTX1	24	hsa-mir-1537-5p	Down	OTX1	9	TP53
Down	CCDC146	11	hsa-mir-941	Down	CCDC146	8	NFKB1
Down	EFHC2	11	hsa-mir-4443	Down	EFHC2	7	HINFP
Down	TSGA10	10	hsa-mir-210-3p	Down	GPR156	7	JUND
Down	KRT15	10	hsa-mir-124-3p	Down	SPAG8	7	SREBF1
Down	PIH1D2	7	hsa-mir-194-5p	Down	PITX1	6	POU2F2
Down	ZMYND12	6	hsa-mir-147a	Down	TRIM29	5	RELA
Down	IQUB	5	hsa-mir-34a-5p	Down	ZMYND12	5	NRF1
Down	MORN3	3	hsa-mir-4747-3p	Down	TEKT4	5	TFAP2C
Down	TEKT4	2	hsa-mir-31-5p	Down	PIH1D2	4	FOS
Down	SPAG8	2	hsa-mir-373-3p	Down	PPP1R32	3	FOXC1
Down	PPP1R32	1	hsa-mir-1-3p	Down	KRT15	3	SOX5

Table S6 Candidate drugs targeted by the hub genes

Categeory	DEGs	Degree	Drug
Up	CA2	95	Cyclothiazide
Up	ADRA1A	89	Tetryzoline
Up	CHRM1	86	Fesoterodine
Up	CHRM2	72	Triflupromazine
Up	CHRM3	67	Methacholine
Up	ADRB1	57	Dobutamine
Up	SLC6A4	56	Protriptyli
Up	CA4	17	Methazolamide
Up	GRIA1	10	Enflurane
Up	SCN1A	10	Phenazopyridine
Up	TNNC1	8	Bepridil
Up	SLC7A11	5	L-Cystine
Up	ADH1A	4	N-Cyclopentyl-N-Cyclobutylformamide
Up	ORM1	3	Chlorpromazine
Up	ORM2	3	Thalidomide
Up	SLC1A1	2	L-Aspartic Acid

Up	NDUFC2	2	Carvedilol
Up	GPR18	1	Glycine
Up	DHCR7	1	NADH
Up	PHGDH	1	NADH
Up	ALDH6A1	1	NADH
Up	GPD1	1	NADH
Up	MSMO1	1	NADH
Up	BDNF	1	CX717
Down	DRD5	26	Rotigotine
Down	GRIK2	21	Hexobarbital
Down	GRIN3B	15	Atomoxetine
Down	MB	12	Methylethylamine
Down	LGSN	1	L-Glutamic Acid
Down	GRM7	1	L-Glutamic Acid
Down	BAAT	1	Glycine
Down	COMP	1	Calcium
